# Fully protected pyrophosphates *via* phosphorobromidates for the synthesis of biopolymers

**DOI:** 10.1039/d6sc01119e

**Published:** 2026-03-09

**Authors:** Sven Wijngaarden, Femke L. A. M. van der Heijden, Koen J. Rijpkema, Bob van Puffelen, Nico J. Meeuwenoord, Danicia Ramcharan, Gijsbert A. van der Marel, Jeroen D. C. Codée, Herman S. Overkleeft, Dmitri V. Filippov

**Affiliations:** a Leiden Institute of Chemistry, Leiden University Einsteinweg 55, 2333 CC Leiden The Netherlands filippov@chem.leidenuniv.nl

## Abstract

Natural oligomers containing phosphodiester linkages, such as DNA and RNA oligomers, are readily accessible through the phosphoramidite P(iii) approach. However, the repetitive introduction of pyrophosphates into an oligomeric backbone, as found in poly-ADP-ribose, remains challenging. The most effective method to date relies on P(iii)–P(v) coupling, in which a phosphate monoester is linked with a phosphoramidite, facilitating chain elongation of the biopolymer following the oxidation of the P(iii)–P(v) intermediate. This anionic and nucleophilic intermediate limits the chain length that can be achieved. To overcome this limitation, we designed a methodology based on P(v)–P(v) chemistry, where a phosphodiester is coupled to a protected and activated P(v) donor. To this end, the Atherton–Todd oxidation of H-phosphonates is utilised to generate an electrophilic halophosphate, which is then coupled with a phosphodiester to yield a fully protected pyrophosphate species. The chemistry was optimised to balance the reactivity and stability of the coupling partners and the product by introducing the *o*-chloro-*p*-nitrophenyl ethyl protecting group for the phosphodiester and pyrophosphates. The full protection of the pyrophosphate moiety during oligomer synthesis eliminates the issues associated with the nucleophilicity and charge of the deprotected pyrophosphate and enables the solid-phase synthesis of pyrophosphate-based ADP-ribose oligomers of unprecedented length.

## Abstract 2

A P(v)–P(v) coupling strategy using phosphorbromidate electrophiles, enables the solid-phase synthesis of fully protected pyrophosphates. This methodology provides synthetic access to defined ADPr fragments of unprecedented length.

## Graphical abstract

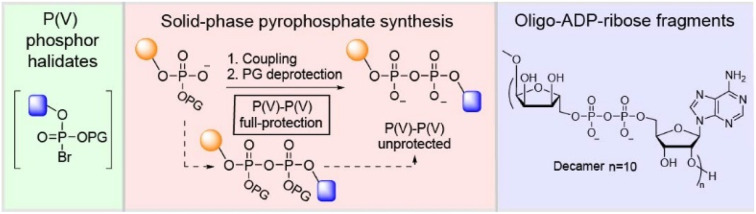

## Introduction

To date, phosphodiester-based biopolymers such as DNA and RNA are readily available due to advancements in oligonucleotide chemistry over the past few decades.^1^ The chemical synthesis of oligonucleotides has provided powerful molecular tools, such as guide strands for CRISPR/Cas9 gene editing^2^ and has enabled many new technologies like gene synthesis^3^ and antisense mRNA suppression.^4^ The use of phosphoramidites, which are P(iii) reagents, form the basis of the remarkably efficient synthetic methods employed in DNA and RNA fragment synthesis.

Adenosine-di-phosphate ribose (ADPr) is a polymer composed of ribosyl adenosines linked through pyrophosphate bonds, and is installed as a post-translational modification on many proteins to fulfill a myriad of biological functions.^5–9^ Well-defined ADPr fragments and synthetic ADPr peptides/proteins have been instrumental in unravelling ADPr biology.^10,11^ However, the generation of longer ADPr chains has been a major challenge.^12,13^ Pyrophosphates are intrinsically labile, as the P(v)–O–P(v) linkage is very susceptible to cleavage. As a result, the synthesis of pyrophosphate-containing biomolecules is far less developed as compared to their phosphodiester-based counterparts. The longest ADPr chain synthesized to date, was assembled using a solid-phase synthesis strategy in which resin-bound phosphate monoesters were reacted with a P(iii) phosphoramidite.^14^ This P(iii)–P(v) methodology has enabled the synthesis of various biomolecules and their derivatives as well as an oligo-ADPr fragment of five repeating units in length.^15–18^ However, the latter synthesis produced substantial amounts of truncated fragments, even when excess coupling reagents and multiple coupling cycles were employed. These shorter fragments likely originate from undesired reactivity associated with the unprotected pyrophosphate during chain elongation.^19–21^ This reduction in synthesis quality for longer ADPr oligomers, together with the nucleophilic and anionic character of the unprotected pyrophosphate during chain elongation, motivated the development of an alternative to the P(iii)–P(v) coupling strategy.

**Fig. 1:**
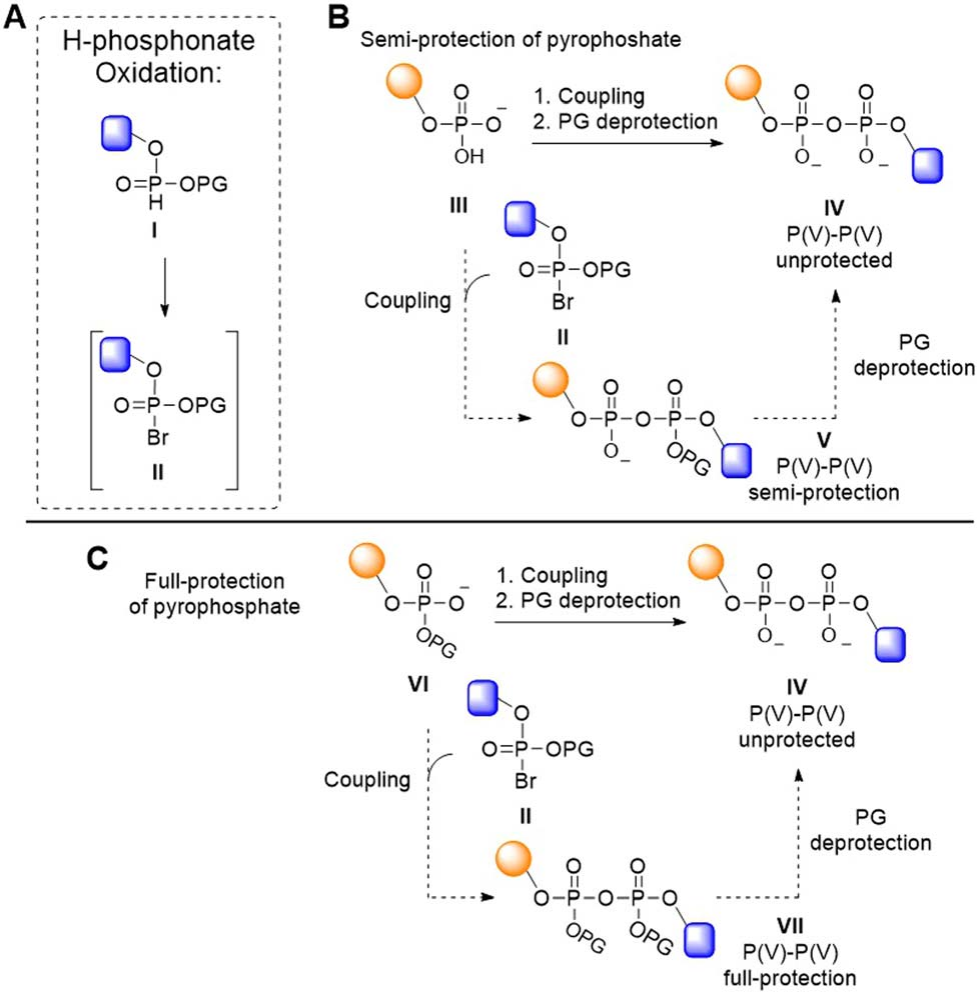
Synthetic strategies for the construction of the pyrophosphate bond *via* P(v) phosphorohalidates. (A) Generation of phosphorobromidate *via* Atherton–Todd oxidation of H-phosphonate. (B and C) Synthesis of pyrophosphate using P(v)–P(v) coupling with semi-protected (B) and fully protected (C) pyrophosphate intermediates.

Pyrophosphates can be introduced through the coupling of a phosphomonoester with an activated P(v) species, such as a halophosphate (Fig. 1). Halophosphates are readily obtained by the Atherton–Todd oxidation of the corresponding H-phosphonate precursors (Fig. 1A).^22^ They are commonly used *in situ*, while they are generally too unstable for standard isolation procedures.^23^ The Atherton–Todd reaction entails oxidation of an H-phosphonate (I) by reaction with a halogenating agent in the presence of a base to provide the electrophilic halogenated P(v) species (II).^24^ This reaction was originally developed to produce phosphoramides, but changing the nucleophilic reaction partner from an amine to a phosphate has delivered methodology to construct diphosphate bonds.^22^ Using this approach, the group of Meier has created di- and triphosphate linkages.^25–27^ Likewise, Hergenrother and colleagues employed an *in situ* generated halophosphate (II) to couple a phosphomonoester (III) in their solution-phase synthesis of di-ADPr, yielding a partially protected pyrophosphotriester (V) that, upon deprotection, furnished the desired diphosphate (Fig. 1B, IV).^28,29^ These partially protected intermediates are difficult to handle in solution, and no method based on this approach has been able to access constructs longer than a dimer.

We reasoned that full protection of the pyrophosphate species as a tetraester during the chain elongation would negate its nucleophilicity (Fig. 1C, VII), which could potentially benefit the synthesis of longer oligo-ADPr and related biopolymers, in line with contemporary oligonucleotide synthesis, in which the phosphates are protected as phosphotriesters. Because phosphodiesters do not react productively with phosphoramidites^30^ we set out to develop a P(v)–P(v) method for repetitive pyrophosphate formation where the coupling proceeds *via* attack of a phosphate diester (VI) on an electrophilic P(v) phosphorohalidate (II), obtained *via* an Atherton–Todd (AT) reaction (Fig. 1).^22^ This approach requires careful handling of the anhydride-type intermediates formed during chain elongation, as these species are highly susceptible to nucleophilic substitution leading to monophosphate byproducts instead of the desired pyrophosphate.^31–34^ Consequently, stringent demands are placed on the protecting groups used to mask both the phosphate that is to be elongated as well as the intermediate pyrophosphate that is formed. Here, we describe the successful development of such an approach, which was enabled by the use of the little-used *o*-chloro-*p*-nitrophenyl ethyl protecting group^35^ for the phosphodiester and pyrophosphotetraester. This protecting group strikes the right balance between pyrophosphate stability, ease of halophosphate formation and removal at the end of the synthesis. We demonstrate the applicability of the methodology in the solid phase assembly of ADPr oligomers up to the decamer level, representing the longest well-defined ADPr chains synthesized to date.

## Results and discussion

### Development of P(v)–P(v) method using oligo-TDP as the model system

To develop the P(v)–P(v) pyrophosphate coupling methodology and protecting group strategy, we first explored the usability of halophosphates (II, Fig. 1) for the synthesis of pyrophosphate-linked oligonucleotides (oligo-TDP), that can be assembled from relatively simple H-phosphonates.

**Fig. 2:**
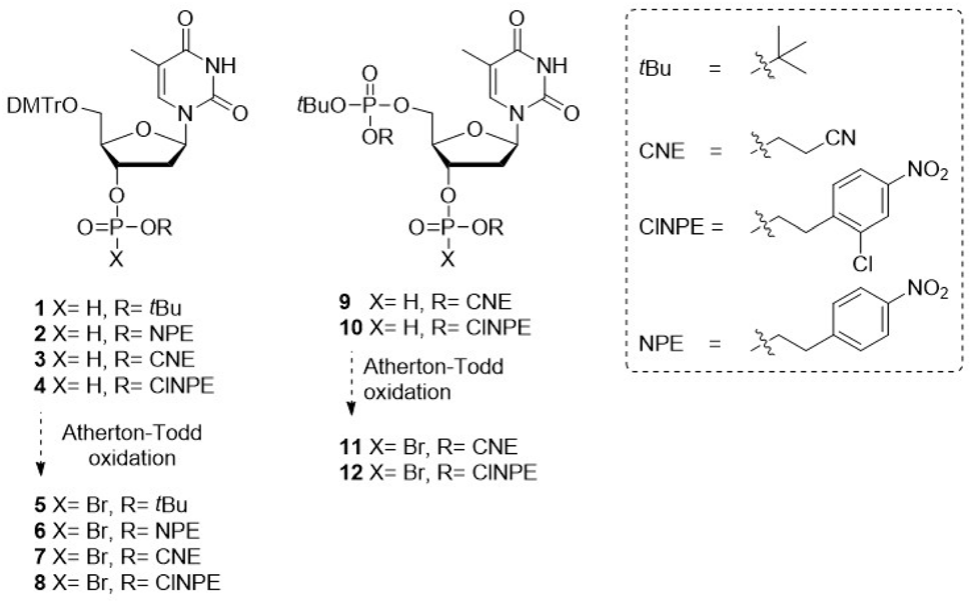
H-phosphonate building blocks required for solid-phase synthesis of oligo-TDP fragments *via* the P(v)–P(v) strategy.

Four 4,4-di-methoxytrityl (DMTr) protected thymidine H-phosphonates were synthesized, each featuring a distinct protecting group on the H-phosphonate functionality: an acid-labile *tert*-butyl (*t*Bu) ester or one of the three increasingly base-labile esters *para*-nitrophenylethyl (NPE), 2-chloro-4-nitrophenylethyl (ClNPE) or 2-cyanoethyl (CNE) (1–4, Fig. 2). Base labile pyrophosphate protecting groups that can be cleaved *via* an elimination reaction have previously been shown to be effective in the P(iii)–P(v) methodology.^14^ The NPE- and ClNPE groups, which can be cleaved by a strong base such as 1,8-diazabicyclo(5.4.0)undec-7-ene (DBU),^35,36^ were envisioned to be compatible with triethylamine (TEA), which is the base commonly used for the Atherton–Todd reaction. While CNE-protected phosphates are susceptible to cleavage by TEA, the milder base *N*-methylmorpholine (NMM) was employed instead.^37^

First, H-phosphonates 1–4 were assessed for their reactivity in the Atherton–Todd reaction using trichlorobromomethane (CBrCl_3_) as the halogenating agent, generating the phosphorobromidates (5–8), the formation of which was monitored by ^31^P-NMR.^38^ We explored two different bases in the reaction: TEA (p*K*_a_H 10.8) and the milder base NMM (p*K*_a_H 7.4).

**Fig. 3:**
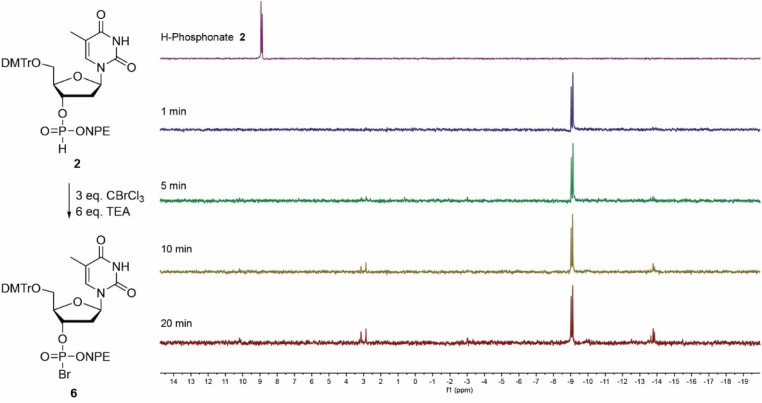
H-phosphonate activation of NPE protected H-phosphonate 2 by CBrCl_3_ and a TEA, measured over time by ^31^P-NMR. Chemical shifts: H-phosphonate 2 (9 ppm), phosphorobromidate 6 (−9 ppm), symmetric pyrophosphotetraester of type VII (−14 ppm).

The oxidation of NPE-protected H-phosphonate 2 (9 ppm) into phosphorobromidate 6 (−9 ppm) using TEA as base, proceeded uneventfully and the conversion was finished within one minute (Fig. 3). After twenty minutes, some hydrolysis to the phosphodiester (3 ppm) and formation of the symmetrical pyrophosphate (−13 ppm) could be detected.^39^ Oxidation of the more base labile ClNPE-protected H-phosphonate 4 was explored using either NMM or TEA as base. In an experiment with NMM no conversion to halogenated phosphate 8 (−9 ppm) could be detected after 2 minutes and the symmetrical pyrophosphotetraester (−14 ppm) was observed as the sole product after twenty minutes (Fig. S1). In contrast, the Atherton–Todd oxidation using TEA showed rapid conversion (<1 minute) into the bromidate 8 (−9 ppm), which was fully hydrolyzed and converted into the symmetrical pyrophosphate (−14 ppm) within three minutes (Fig. S2). No cleavage of the ClNPE-group could be detected under these conditions. The NMM-assisted oxidation of CNE-protected H-phosphonate 3 was significantly slower and complete conversion into the corresponding phosphorobromidate 7 was observed after approximately twenty minutes (Fig. S3). Here, slow hydrolysis, accompanied by the formation of the protected symmetrical pyrophosphate (−14 ppm) was also observed. The conversion of the *t*Bu-protected H-phosphonate 1 into the corresponding phosphorobromidate 5 (−15 ppm) proceeded the slowest and took approximately 30 minutes (Fig. S4).

The halophosphates (5–8) featuring the four different protective groups were next evaluated as electrophilic agents in the preparation of fully protected pyrophosphates. To this end, the solid-phase syntheses of oligo-TDP dimer 21 and trimer 26 was undertaken (Table 1). After the manual construction of the pyrophosphate linkages on resin, they were deprotected, the product was cleaved from the solid support and the coupling quality assessed *via* LCMS analysis.

**Table 1:** Exploratory solid-phase syntheses towards oligo-TDP compounds 21 and 26 and optimization of the protecting group strategy^a^

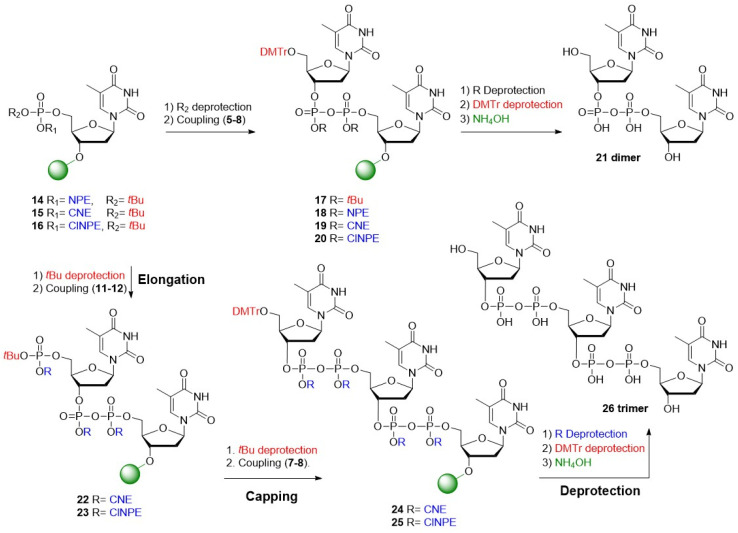								
#	Pyrophosphate protecting group R	Product	Base	Halogenating agent	Coupling time	R cleavage conditions	Notes	Yield^c^
1	*t*Bu	Dimer 21	TEA	CBrCl_3_	b30 min	20% TFA	—	77%
2	NPE	Dimer 21	TEA	CBrCl_3_	10 min	15% DBU	Incomplete deprotection of NPE	53%
3	CNE	Dimer 21	NMM	CBrCl_3_	10 min	5% DBU	—	89%
4	CNE	Trimer 26	NMM	CBrCl_3_	b10 min	5% DBU	19% dimer 21^c^	66%
5	ClNPE	Dimer 21	NMM	CBrCl_3_	b10 min	20% DBU	—	98%
6	ClNPE	Trimer 26	NMM	CBrCl_3_	b10 min	20% DBU	25% dimer 21^c^ + other side products	68%
7	ClNPE	Dimer 21	TEA	CBrCl_3_	1 min	20% DBU	—	93%
8	ClNPE	Trimer 26	TEA	CBrCl_3_	b1 min	20% DBU	10% dimer 21^c^	83%
9	ClNPE	Trimer 26	TEA	NCS	b1 min	20% DBU	27% dimer 21^c^	65%

a The scale of test syntheses was 2 µmol. General conditions during coupling reactions: 3 eq. of H-phosphonate 1–4, 6 eq. CBrCl_3_ and 9 eq. of base. The concentration of immobilized phosphodiester during coupling was 11 mM. *t*Bu was deprotected using 2 cycles of 10% v/v TFA in DCM. After *t*Bu deprotection, a washing step with the base was performed. Protecting groups depicted in blue are removed under basic conditions and those in red under acidic conditions. Cleavage from the solid support depicted in green was achieved under alkaline conditions. Solid-phase syntheses towards oligo-TDP compounds 21 and 26 and optimization of the protecting group strategy.

b Double coupling procedure.

c Estimated yield based on integration of UV-signal at 268 nm in the LC-MS traces.

The first approach explored the use of base-labile temporary phosphate protection in combination with acid-labile “permanent” pyrophosphate protection. Solid-phase synthesis commenced with the preparation of immobilized phosphotriester 16. The corresponding resin-bound *t*Bu-protected phosphodiester was obtained after cleavage of the ClNPE ester, which was condensed with *t*Bu-protected P(v) bromidate 5 to yield the acid-sensitive pyrophosphotetraester 17. Subsequent acidolysis of the *t*Bu pyrophosphate esters and cleavage from the solid support resulted in dimer 21 in suboptimal yield (Table 1, entry 1 and Fig. S5A).

The second approach employed acid-labile temporary protection together with base-labile “permanent” pyrophosphate protection. Acidolysis of the *t*Bu ester in immobilized phosphotriester 14, was followed by a wash with base to provide the phosphodiester as an organic salt, enabling coupling to phosphorobromidate 6 using TEA as a base. Deprotection of the pyrophosphotetraester with DBU, followed by cleavage of the oligomer from the solid support afforded dimer 21 (Table 1, entry 2 and Fig. S5B). Unfortunately, LCMS analysis of the reaction mixture revealed incomplete removal of the NPE-ester in pyrophosphotetraester 18. Repetition of the NPE-cleavage procedure or increasing the concentration of DBU did not improve the yield of the synthesis, and the NPE protecting group was therefore not pursued further.

Next, the CNE-group was evaluated in the construction of dimer 21. Using the coupling cycle described above, the oligo-TDP dimer 21 was obtained in good purity (Table 1, entry 3 and Fig. S5C). Encouraged by these results, the synthesis of trimer 26 was undertaken. Cleavage of the *t*Bu-ester in 15 generated the corresponding phosphodiester, which was coupled with phosphorobromidate 11 to afford the immobilized pyrophosphate 22, featuring a protected phosphate for the next elongation. Subsequent removal of the *t*Bu ester in 22 and coupling with the chain terminating bromidate 7 yielded the fully protected trimer 24. Next, deprotection of the CNE-phosphate esters, removal of the primary DMT and cleavage of the oligomer from the solid support yielded trimer 26. LCMS analysis revealed formation of the trimer in 68%, but also substantial amount of dimer 21 (∼20%, Table 1, entry 4 and Fig. S5D).

Lastly, the ClNPE ester was investigated following the same strategy. Elimination of the ClNPE pyrophosphate esters using DBU and subsequent cleavage from the solid support afforded dimer 21, again in good purity with minimal side products (Table 1, entry 5 and Fig. S5E). Using these coupling and deprotection conditions, trimer 26 was produced as a mixture with dimer 21 along with other byproducts originating from incomplete elongation and termination (Table 1, entry 6 and Fig. S5F). Based on the Atherton–Todd activation studies described above, we hypothesized that switching the base from NMM to TEA would accelerate halophosphate formation and thereby improve the overall efficiency of the synthesis.

**Fig. 4:**
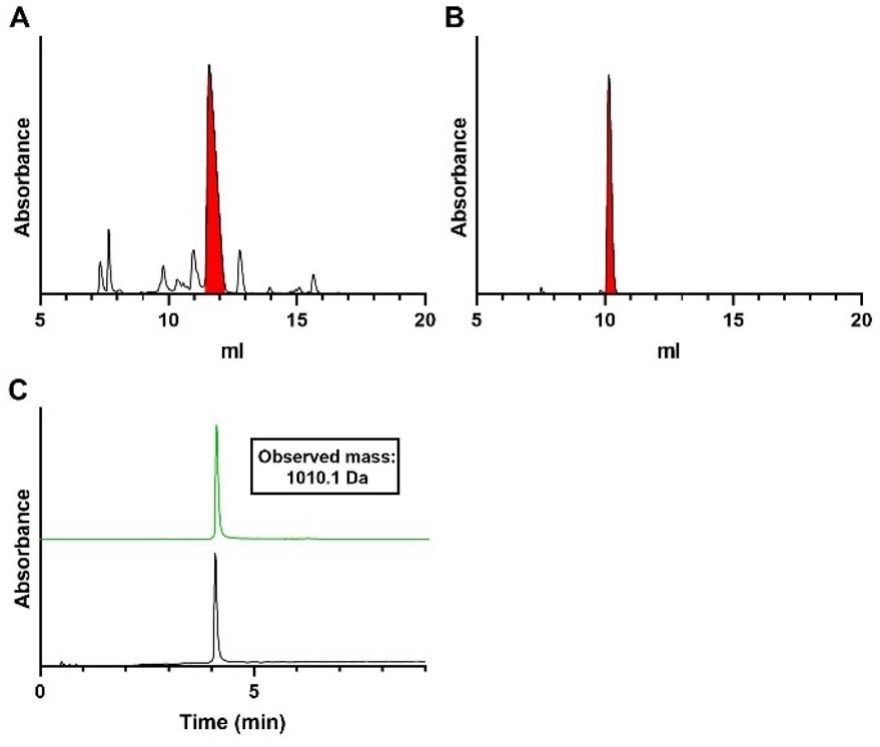
Analysis of oligo-TDP trimer 26. (A) Crude anion exchange chromatogram after solid-phase synthesis. Characterization of purified trimer 26 by (B) anion exchange chromatography (C) LCMS analysis. For IEX: gradient is 0–50% buffer B, absorbance is measured at 260 nm. For LCMS: gradient is 0–20% buffer D + 1% buffer 1 E, graph in black depicts the total absorbance measured from 220–680 nm and graph in green the absorbance of thymine (268 nm). Decon. = deconvoluted mass. Calc. = calculated mass.

Indeed, the synthesis of dimer 21 using H-phosphonate 4, CBrCl_3_ and TEA for the coupling reaction, delivered 21 in excellent purity (Table 1, entry 7 and Fig. S5G). The ensuing solid-phase synthesis of trimer 26*via* sequential coupling of phosphorobromidates 12 and 8 delivered the target trimer 26 in 83% with significantly less dimer being formed (∼10%, Table 1, entry 8 and Fig. S5H), representing a significant improvement over the previous attempts. Substitution of CBrCl_3_ with the commonly used halogenating reagent *N*-chlorosuccinimide (NCS) did not enhance the outcome and instead gave a lower yield of trimer 26 (Table 1, entry 9 and Fig. S5I). Using the optimized conditions of Table 1, entry 8, a preparative synthesis of the trimer 26 was undertaken (Fig. 4A). The pure oligo-TDP trimer could be isolated by ion exchange chromatography (IEX), followed by size exclusion chromatography, in 30% yield (Fig. 4B and C).

### Utilizing the P(v)–P(v) methodology for synthesis of ADPr-oligomers

**Scheme 1:**
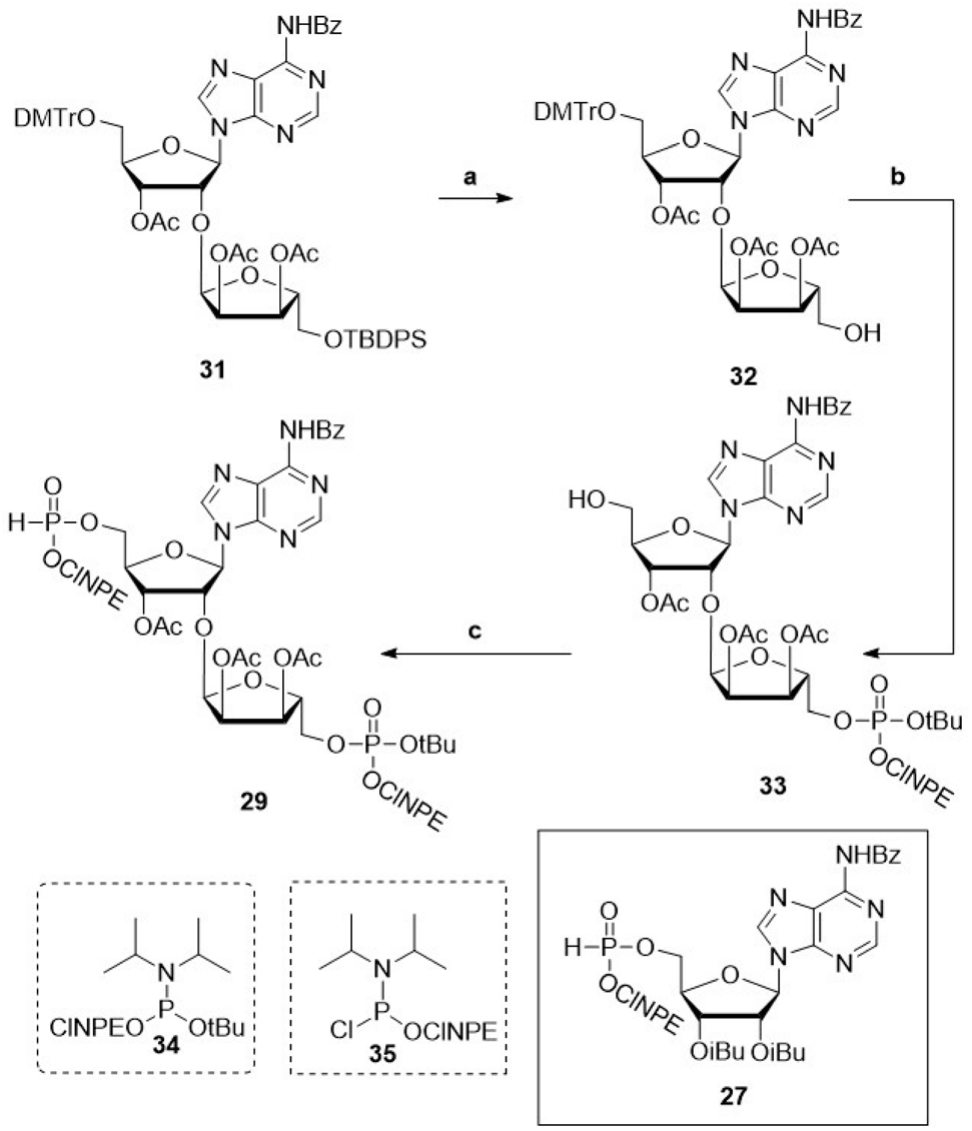
Synthesis of building block 29 required for solid-phase synthesis of ADPr oligomers. Reagents and conditions: (a) TBAF, THF, RT, ON, 80% (32). (b) (i) 34, NMI, NMI·HCl, 3 Å molecular sieves, RT, 15 min, (ii) *t*BuOOH, 0 °C, 30 min, (iii) TFA, DCM, RT, 30 min, 90% (33). (c) (i) 35, NMM, DCM, RT, 30 min, (ii) pyridine·HCl, H_2_O, ACN, RT, 1 h, 74% (29).

With conditions in hand for the solid-phase construction of the pyrophosphate linkages *via* fully protected pyrophosphates, we next set out to explore this chemistry for the synthesis of oligo-ADPr fragments. To this end, appropriately protected elongating and terminating H-phosphonate building blocks were designed based on the protecting group strategy developed above (27 and 29, Scheme 1). The central elongating building block 29 (Scheme 1) was prepared starting from orthogonally protected, ribosylated adenosine 31.^15^ Cleavage of the silyl ether using tetra-*n*-butylammoniumfluoride (TBAF), produced primary alcohol 32 in 80% yield, which was phosphitylated using the asymmetric *t*Bu/ClNPE protected phosphoramidite 34 giving the phosphite triester. This P(iii) species was oxidized by *t*BuOOH into the corresponding phosphotriester, after which acidolysis of the DMTr ether gave alcohol 33 in 90% yield over three steps. The H-phosphonate was then installed on the 5′-hydroxyl of compound 33*via* phosphitylation with chlorophosphoramidite 35 followed by pyridine·HCl mediated hydrolysis of the resulting phosphoramidite, to furnish the advanced phosphoryl H-phosphonate adenosine diriboside 29 in 74% yield over two steps.

Atherton–Todd oxidation of the synthesized H-phosphonates showed comparable reaction kinetics as compared to the oxidation experiments described for oligo-TDP building block 4 (Fig. S6 and S7). A preparative solution-phase synthesis of mono-ADPr 66 afforded the product in 11% yield after purification, demonstrating the applicability of this approach in solution-phase settings (Scheme S7A). Attempts to isolate the corresponding pyrophosphotetraester 48 were unsuccessful, consistent with the pronounced lability of this species. Likewise, an attempted synthesis of dimer 42 proved abortive as no full-length product was detected (Scheme S7B).

**Scheme 2:**
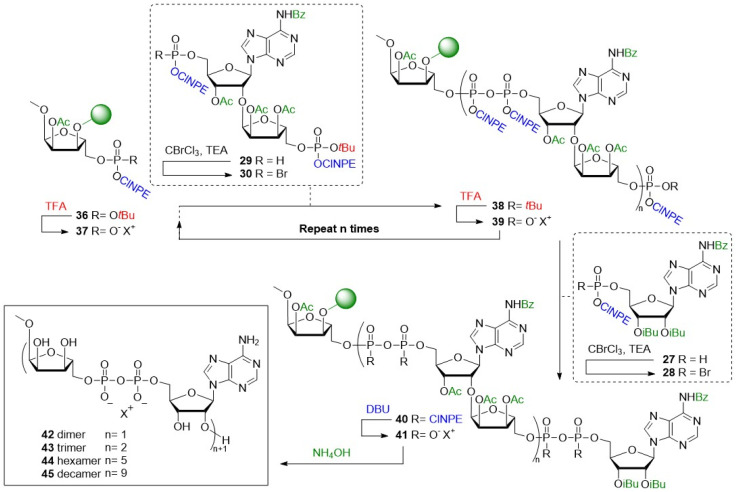
Solid-phase synthetic strategy for poly-ADPr fragments 42–45. Protecting groups depicted in blue are deprotected using basic conditions, in red are deprotected using acidic conditions and in green are deprotected using alkaline conditions.

With the required building blocks in hand, the solid phase assembly of the ADPr oligomers was undertaken (Scheme 2). Starting from resin bound triester 36, the elongation cycle began with acidolysis of the *t*Bu ester, followed by a wash with NMM to yield phosphodiester 37, which was then coupled with phosphorobromidate 30, generated *in situ via* the Atherton–Todd oxidation of H-phosphonate 29. This coupling procedure was repeated once to improve conversion, delivering immobilized pyrophosphotetraester 40 (*n* = 1). Repeating this cycle 2 times allowed for the synthesis of an oligo-ADPr trimer (38, *n* = 2). Termination of the oligomers involved removal of the *t*Bu ester and coupling with phosphorobromidate 28, to produce the protected full-length ADPr-oligomers 40.

**Fig. 5:**
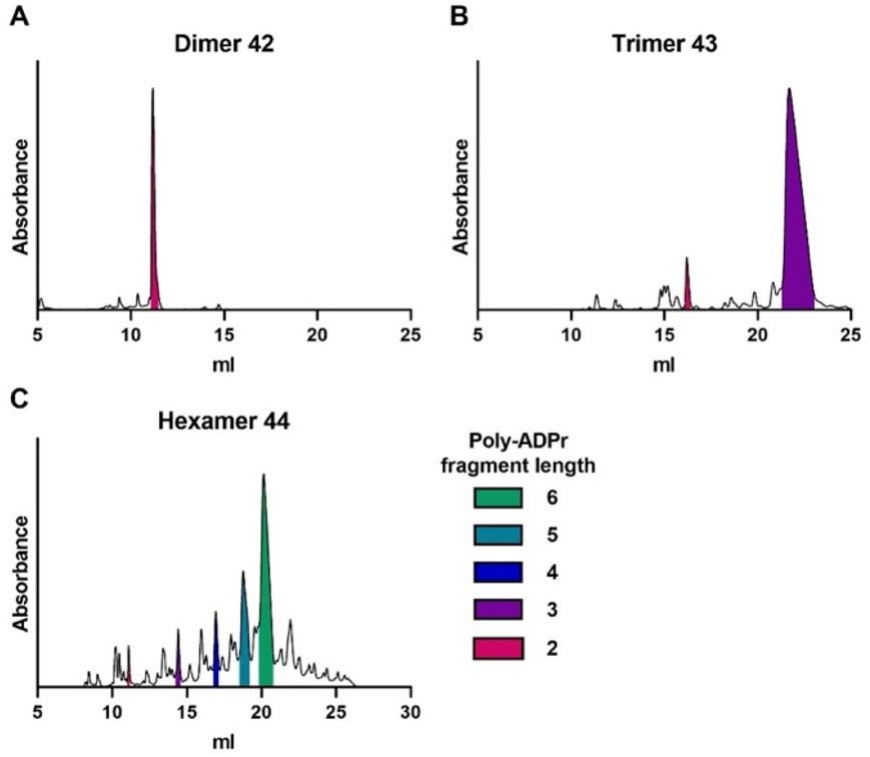
Analytical anion exchange chromatograms of crude poly-ADPr (A) dimer 42, (B) trimer 43 and (C) hexamer 44, obtained *via* the solid-phase synthetic strategy depicted in Scheme 2. The area under the curve of the individual poly-ADPr fragments are colored as depicted in the legend. UV absorbance is measured at 260 nm.

The pyrophosphates in the oligo-ADPr chain were then deprotected by first removal of the ClNPE esters using DBU, giving the pyrophosphodiesters 41, followed by global deprotection with concomitant cleavage from the solid support using aqueous ammonia to deliver the desired poly-ADPr fragments. The so-generated oligomers were analyzed by LCMS and purified by ion exchange chromatography (IEX) and size-exclusion chromatography. As shown in Fig. 5A the assembly of dimer proceeded uneventfully to deliver 42, which was isolated in a 27% yield based on resin loading. Analysis of the product mixture, generated in the synthesis of trimer 43, revealed the presence of significant amounts of dimer 42, which could originate from incomplete deprotection of the *t*Bu phosphate ester. We therefore adapted the synthetic protocol by using an increased concentration of TFA for the elimination of the *t*Bu ester, resulting in trimer 43 in a significantly improved yield (30% isolated yield, Fig. 5B).

With the successful preparation of trimer 43, the synthesis of fragments longer than a pentamer were attempted, which were previously inaccessible.^16^ The synthesis of ADP-ribose hexamer 44 proceeded well, delivering the target compound as the main product (Fig. 5C), although deletion sequences were also present in the mixture. Nonetheless, the hexamer could be effectively isolated to give the poly-ADPr fragment in 8.8% overall yield.

**Fig. 6:**
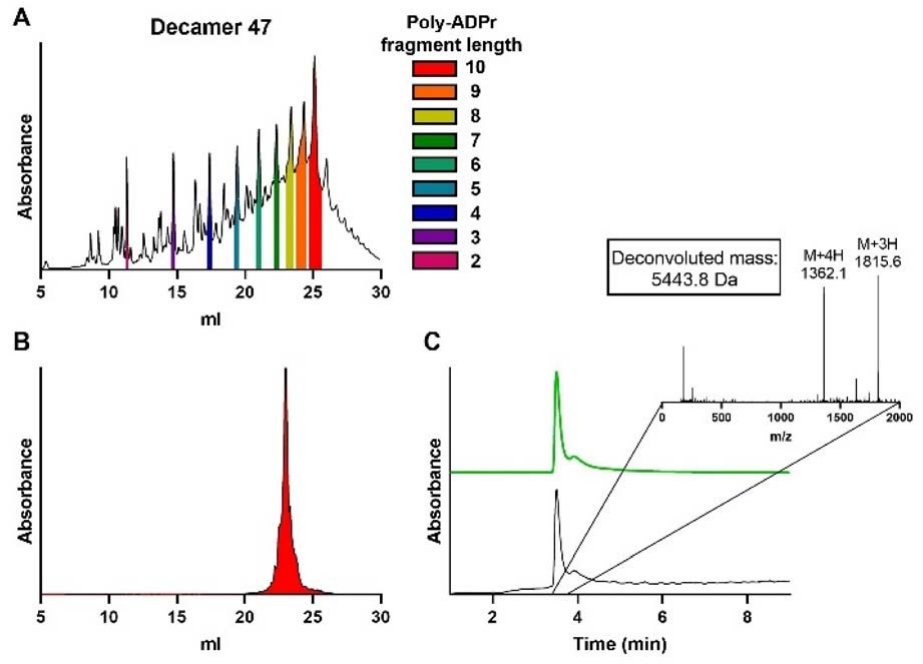
Synthesis and analysis of poly-ADPr decamer 45. (A) Crude anion exchange chromatogram after solid-phase synthesis. The area under the curve of the individual poly-ADPr fragments are colored as depicted in the legend. Characterization of purified decamer by (B) anion exchange chromatography (C) LCMS analysis. Expected mass for decamer 45 is 5442.6 Da. For IEX: gradient is 0–50% buffer B. Absorbance is measured at 260 nm. For LCMS: gradient is 0–20% buffer D + 1% buffer 1 E, graph in black depicts the total absorbance measured from 220–680 nm and graph in green the absorbance of adenine (260 nm).

Finally, the synthesis of decamer 45 was undertaken. Repetition of the elongation cycle nine times, followed by the termination cycle, furnished ADPr decamer 45. Analysis of the crude reaction mixture (Fig. 6A) showed the successful synthesis of the target decamer 45. Isolation by ion exchange chromatography afforded decamer 45 in 4.8% yield (Fig. 6A and B), completing the synthesis of the longest ADPr oligomer to date.

During the synthesis of decamer 45, truncated fragments ranging from the nonamer to the dimer were detected. Several processes could account for these species, including incomplete coupling, insufficient removal of the temporary protecting group, or phosphoanhydride scission during chain elongation. Trace amounts of water during coupling can consume phosphorobromidate, leading to incomplete coupling. Indeed, performing the solid-phase synthesis without rigorously dried reagents resulted in no detectable full-length product (Fig. S8, top *vs.* middle). Incomplete deprotection of the *t*Bu group could provide another possible mechanism for the observed deletion products. If the *t*Bu group is not fully removed at the end of an elongating cycle, the resulting protected intermediate cannot undergo the subsequent coupling step. In the next cycle, however, this unreacted intermediate, now lacking the monomer that should have been added, can re-enter the coupling chemistry and propagate through the remaining cycles, ultimately generating the truncation products. This was already observed during an exploratory synthesis of hexamer 44, in which 10% TFA yielded small amounts of full-length product. Increasing the TFA concentration to 20% improved deprotection efficiency (Fig. S8, middle *vs.* bottom). Phosphoanhydride scission during synthesis is also conceivable, given the intrinsic sensitivity of the pyrophosphotetraester linkage. Although such fragments were less apparent in shorter oligomers, cumulative low-frequency side reactions over multiple coupling cycles could reasonably cause the pronounced truncation pattern seen in the decamer.

## Conclusions

In this work, a solid-phase synthesis protocol to generate well-defined ADPr oligomers was developed, based on halophosphate P(v)–P(v) coupling methodology. The approach relies on the generation of fully protected pyrophosphotetraesters as intermediates during chain elongation, obtained by condensation of a phosphodiester with a phosphorobromidate, generated *in situ via* Atherton–Todd oxidation of the corresponding H-phosphonate. Crucial to the success of the approach has been the development of a protecting group scheme that hinges on the protection of the pyrophosphate with the ClNPE group, that balances the right stability and reactivity during the phosphorobromidate generation, the condensation reaction and can be cleaved under mild conditions that do not jeopardize the integrity of the labile (protected) pyrophosphate moieties. The presented solid-phase method successfully produced ADPr oligomers of unprecedented length, delivering the longest ADPr oligomer, assembled to date. The developed methodology is a valuable addition to the current toolbox for the synthesis of well defined poly-ADP-ribose chains and poly-ADPr proteins and peptides, as well as other biopolymers containing sugar-pyrophosphate chains. The generated ADPr oligomers will be valuable tools to probe interactions with ADPr processing enzymes and receptors.

## Author contributions

S. W.: investigation, methodology, conceptualisation, writing – original draft; F. L. A. M. v. d. H.: investigation, resources, writing – review & editing; K. J. R.: investigation, resources, writing – review & editing; B. v. P.: investigation, resources, writing – review & editing; N. J. M.: investigation, resources; D. R.: investigation, resources; G. A. v. d. M.: conceptualisation, writing – original draft, writing – review & editing; J. D. C. C.: supervision, writing – review & editing; H. S. O.: supervision, funding acquisition, writing – review & editing: D. V. F.: conceptualisation, supervision, funding acquisition, writing – original draft, writing – review & editing.

## Conflicts of interest

There are no conflicts to declare.

## Supplementary Material

SC-OLF-D6SC01119E-s001

SC-OLF-D6SC01119E-s002

## Data Availability

The data supporting this article have been included as part of the supplementary information (SI). The authors have cited two additional references within the SI.^40,41^ Supplementary information: figures, synthetic procedures and raw NMR spectra. See DOI: https://doi.org/10.1039/d6sc01119e.

## References

[cit1] Reese C. B. (2005). Org. Biomol. Chem..

[cit2] Chen Q., Zhang Y., Yin H. (2021). Adv. Drug Delivery Rev..

[cit3] Czar M. J., Anderson J. C., Bader J. S., Peccoud J. (2009). Trends Biotechnol..

[cit4] Lennox K. A., Behlke M. A. (2011). Gene Ther..

[cit5] Fehr A. R., Singh S. A., Kerr C. M., Mukai S., Higashi H., Aikawa M. (2020). Genes Dev..

[cit6] Suskiewicz M. J., Prokhorova E., Rack J. G. M., Ahel I. (2023). Cell.

[cit7] Dantzer F., Santoro R. (2013). FEBS J..

[cit8] Gupte R., Liu Z., Kraus W. L. (2017). Genes Dev..

[cit9] Rosado M. M., Bennici E., Novelli F., Pioli C. (2013). Immunology.

[cit10] Leung A. K. L. (2020). Trends Cell Biol..

[cit11] Minnee H., Codee J. D. C., Filippov D. V. (2024). ChemBioChem.

[cit12] Liu Q., van der Marel G. A., Filippov D. V. (2019). Org. Biomol. Chem..

[cit13] van der Heden van Noort G. J. (2020). ACS Omega.

[cit14] Gold H., van Delft P., Meeuwenoord N., Codee J. D. C., Filippov D. V., Eggink G., Overkleeft H. S., van der Marel G. A. (2008). J. Org. Chem..

[cit15] Kistemaker H. A. V., Lameijer L. N., Meeuwenoord N. J., Overkleeft H. S., van der Marel G. A., Filippov D. V. (2015). Angew. Chem., Int. Ed..

[cit16] Liu Q., Knobloch G., Voorneveld J., Meeuwenoord N. J., Overkleeft H. S., van der Marel G. A., Ladurner A. G., Filippov D. V. (2021). Chem. Sci..

[cit17] Kliza K. W., Liu Q., Roosenboom L. W. M., Jansen P. W. T. C., Filippov D. V., Vermeulen M. (2021). Mol. Cell.

[cit18] Kistemaker H. A. V., Meeuwenoord N. J., Overkleeft H. S., Van Der Marel G. A., Filippov D. V. (2015). Eur. J. Org Chem..

[cit19] Weimann G., Khorana H. G. (1962). J. Am. Chem. Soc..

[cit20] Sun Q., Liu S., Sun J., Gong S., Xiao Q., Shen L. (2013). Tetrahedron Lett..

[cit21] Knorre D. G., V Lebedev A., Levina A. S., Rezvukhin A. I., Zarytova V. F. (1974). Tetrahedron.

[cit22] Atherton F. R., Openshaw H. T., Todd A. R. (1945). J. Chem. Soc..

[cit23] Kraszewski A., Stawinski J. (2007). Pure Appl. Chem..

[cit24] Le Corre S. S., Berchel M., Couthon-Gourvès H., Haelters J. P., Jaffrès P. A. (2014). Beilstein J. Org. Chem..

[cit25] Jeschik N., Schneider T., Meier C. (2020). Eur. J. Org Chem..

[cit26] Jia X., Schols D., Meier C. (2023). J. Med. Chem..

[cit27] Weising S., Weber S., Schols D., Meier C. (2022). J. Med. Chem..

[cit28] Lambrecht M. J., Brichacek M., Barkauskaite E., Ariza A., Ahel I., Hergenrother P. J. (2015). J. Am. Chem. Soc..

[cit29] Drown B. S., Shirai T., Rack J. G. M., Ahel I., Hergenrother P. J. (2018). Cell Chem. Biol..

[cit30] Guzaev A. P., Manoharan M. (2001). J. Org. Chem..

[cit31] Sonveaux E. (1986). Bioorg. Chem..

[cit32] Ivanova E. M., Khalimskaya L. . M., Romanenko V. P., Zarytova V. F. (1982). Tetrahedron Lett..

[cit33] Chandrasegaran S., Murakami A., Kan L. (1984). J. Org. Chem..

[cit34] Kenner G. W., Todd A. R., Weymouth F. J. (1952). J. Chem. Soc..

[cit35] Uhlmann E., Pfleiderer W. (1981). Helv. Chim. Acta.

[cit36] Pfleiderer W., Himmelsbach F., Charubala R., Schirmeister H., Beiter A., Schulz B., Trichtinger T. (1985). Nucleosides Nucleotides.

[cit37] Hsiung H. M. (1982). Tetrahedron Lett..

[cit38] Atherton F. R., Todd A. R. (1947). J. Chem. Soc..

[cit39] Nilsson J., Kraszewski A., Stawinski J. (2001). J. Chem. Soc., Perkin Trans. 2.

[cit40] Claesen C. A. A., Segers R. P. A. M., Tesser G. I. (1985). Recl. Trav. Chim. Pays-Bas.

[cit41] Van Der Heden Van Noort G. J., Overkleeft H. S., Van Der Marel G. A., Filippov D. V. (2010). J. Org. Chem..

