# Supplementary material for: Fully protected pyrophosphates via phosphorobromidates for the synthesis of biopolymers
Source: Chem Sci. 2026 Mar 9;17(17):8630–7. doi: 10.1039/d6sc01119e (PMC12970391; doi:10.1039/d6sc01119e)
Supplement: SC-017-D6SC01119E-s001 [file SC-017-D6SC01119E-s001.pdf]

Supplementary Information (SI) for Chemical Science.
This journal is © The Royal Society of Chemistry 2026

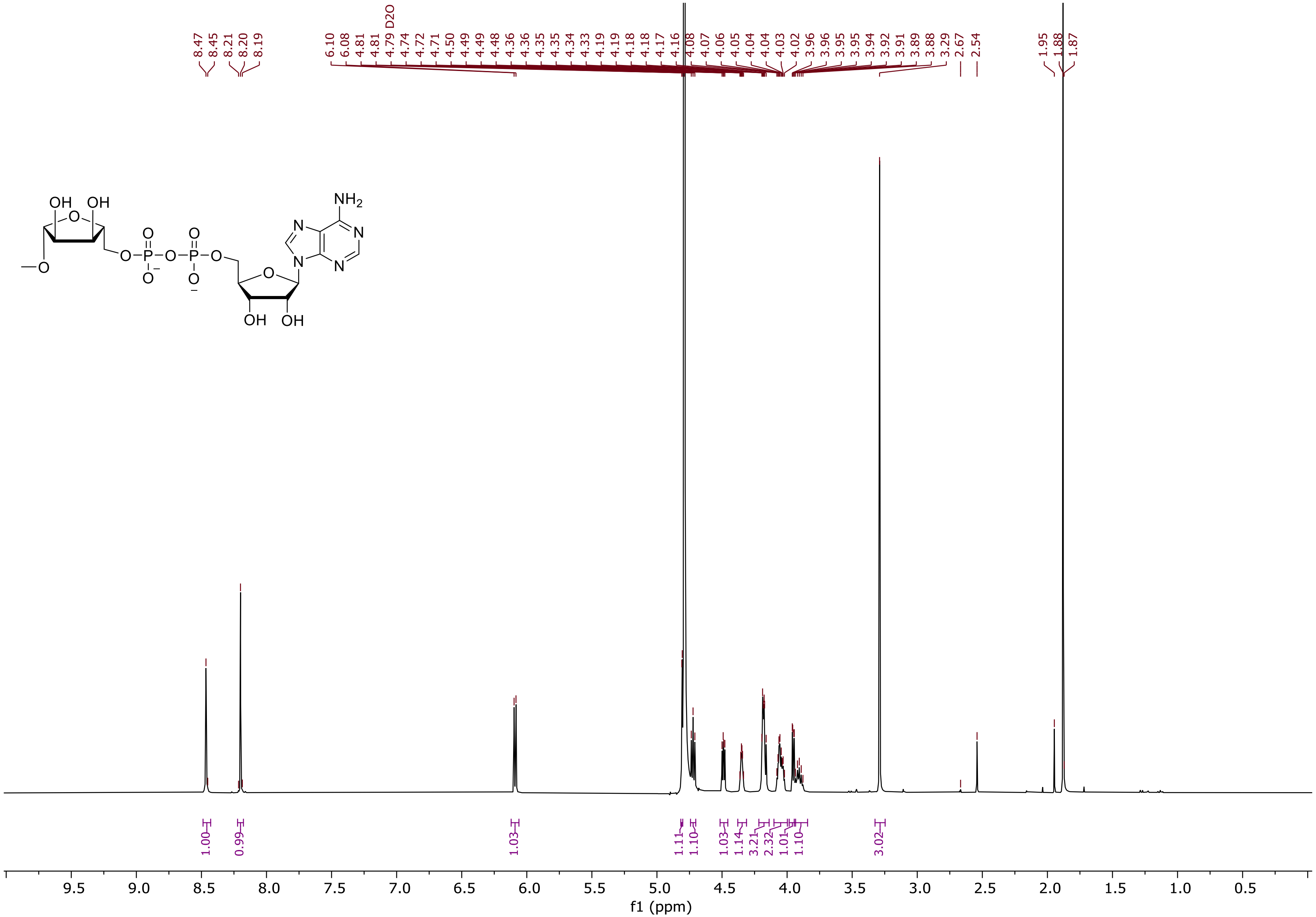

^{1}H-NMR (400 MHz, D_2O) of compound **66**. Solvent peak at 4.79 ppm.

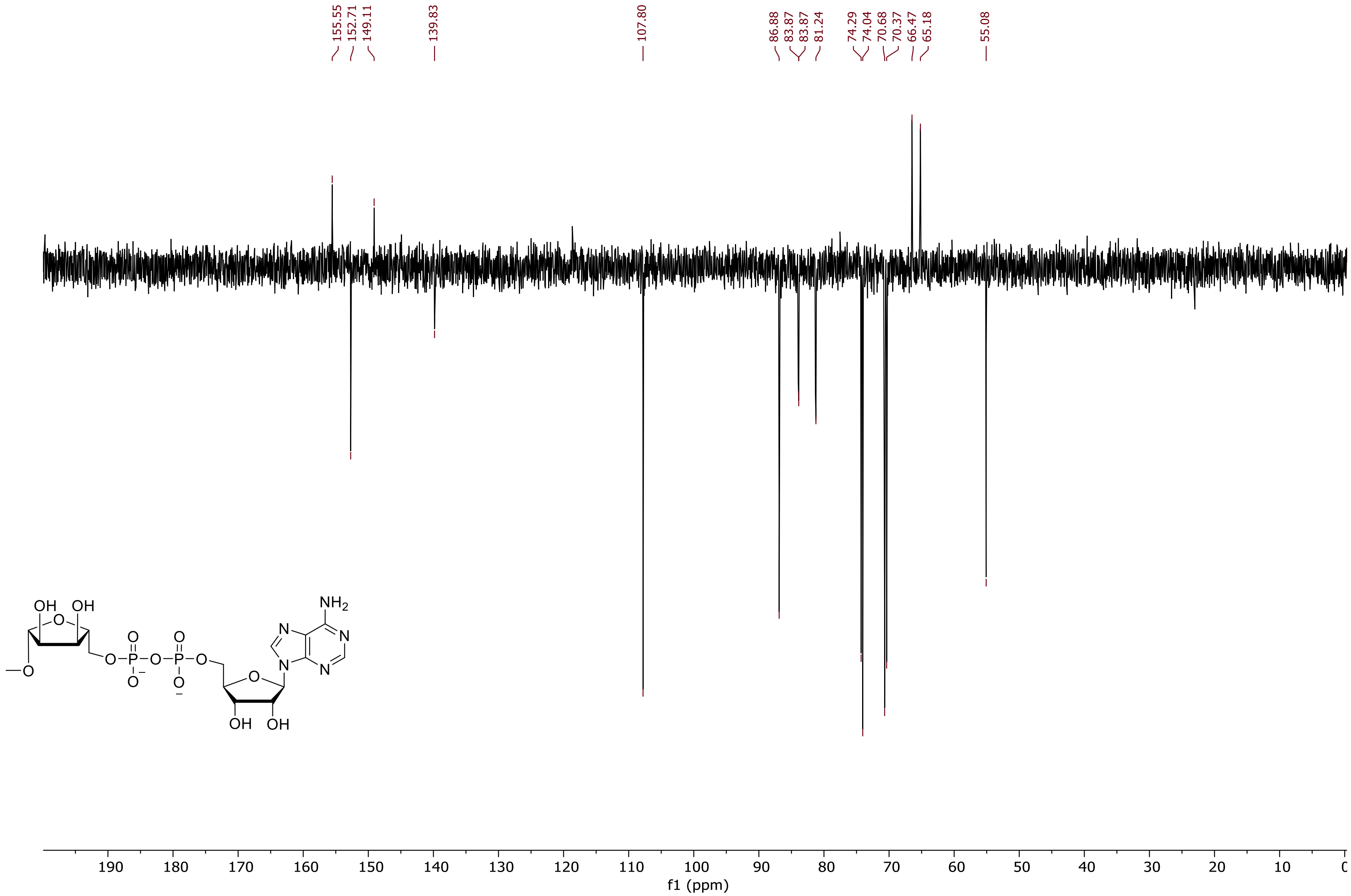

^{31}C-NMR (101 MHz, D_2O) of compound **66**.

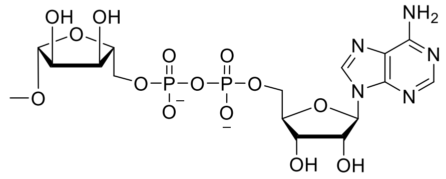

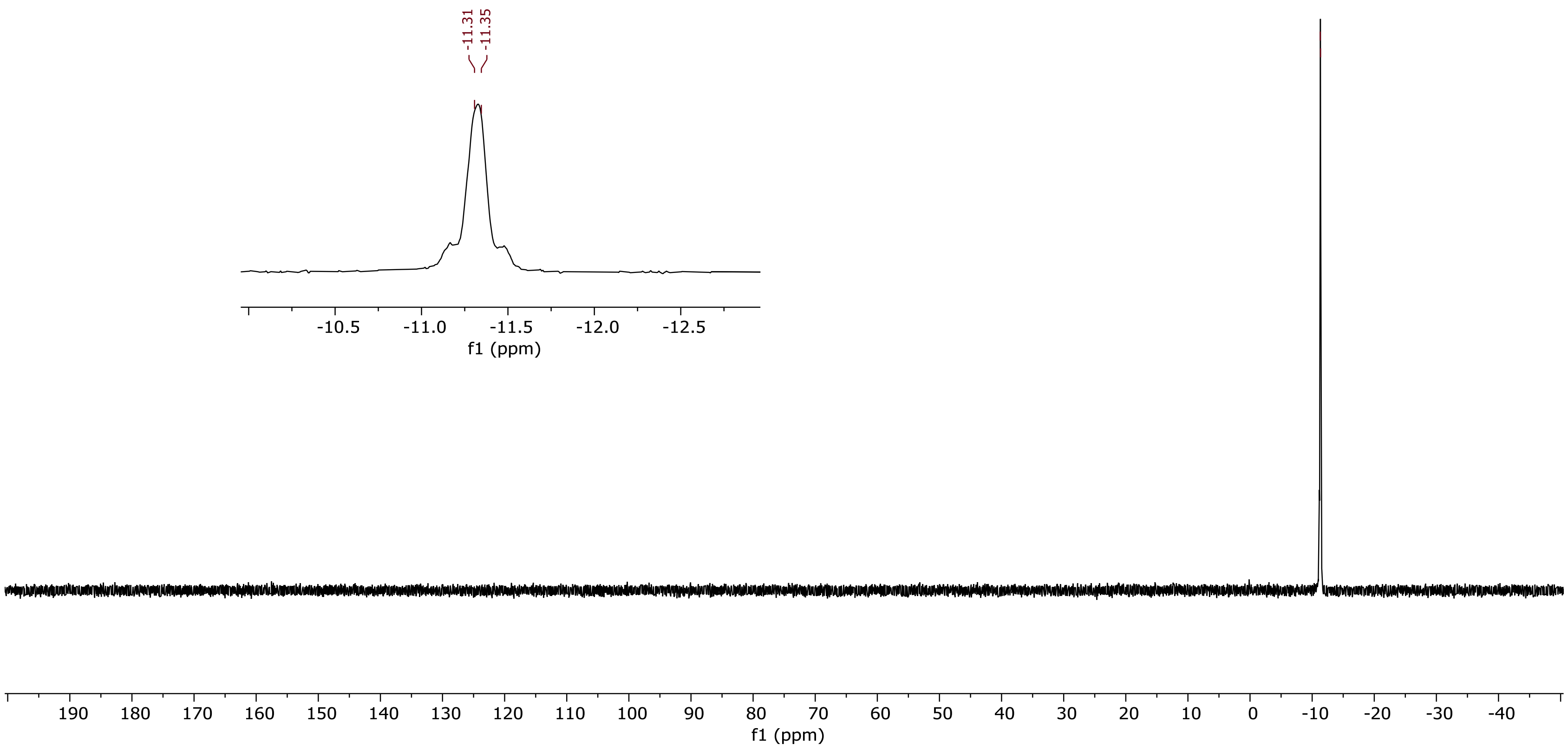

^{31}**P-NMR** (162 MHz, D_2O) of compound **66**.

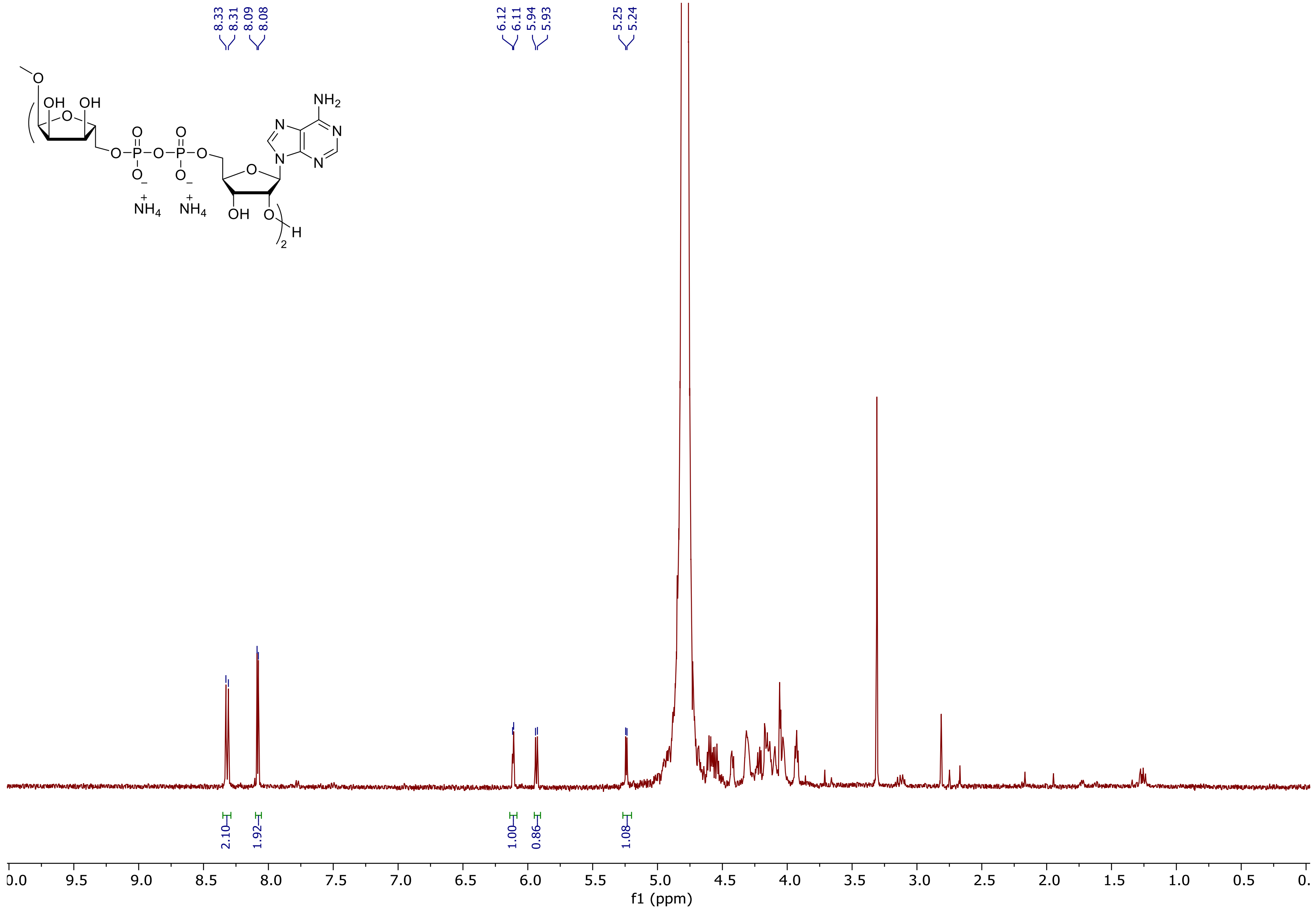

^{1}H-NMR (400 MHz, D_2O) of compound **42**. Solvent peak at 4.79 ppm.

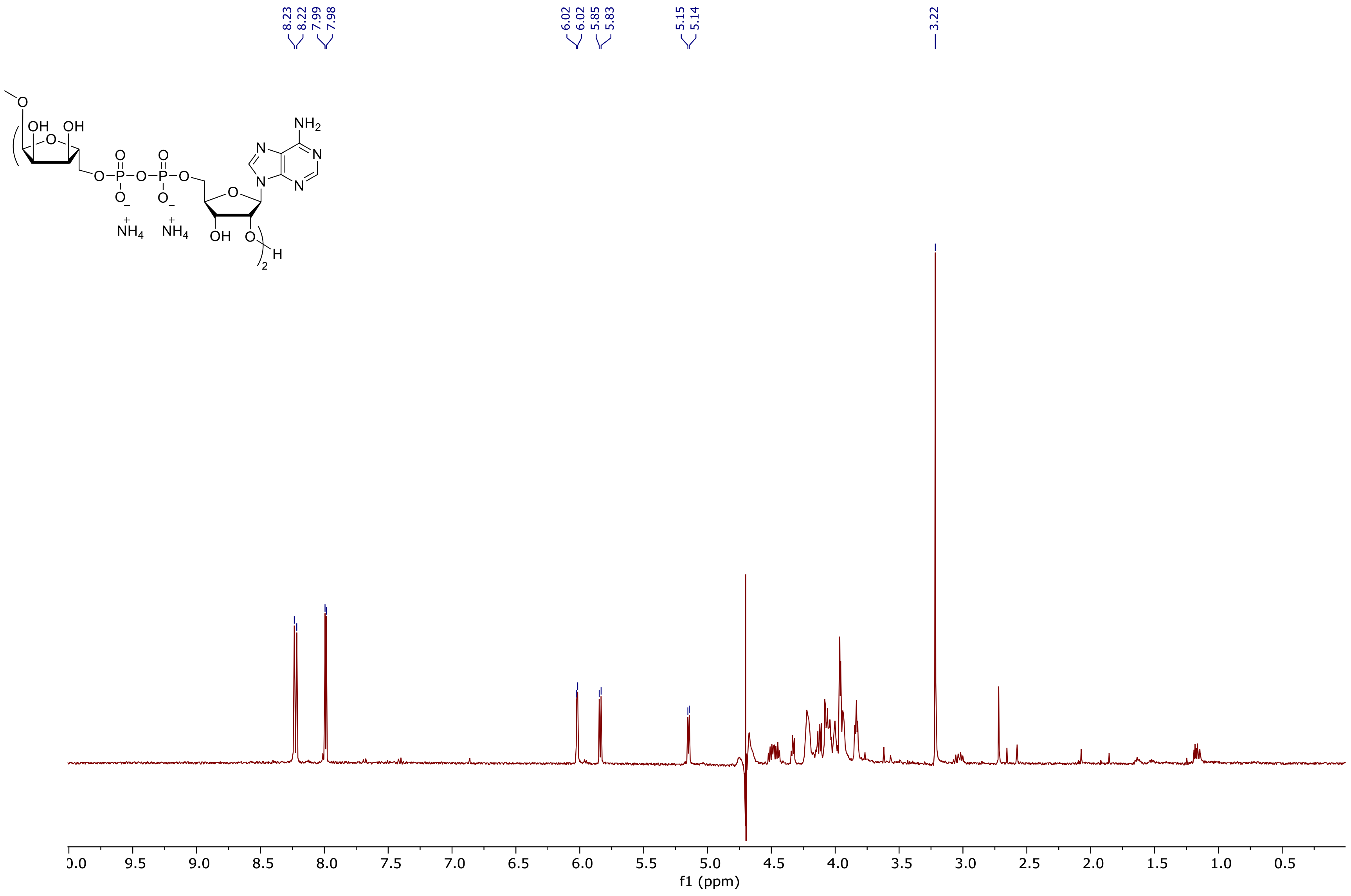

[1]**H-NMR** (400 MHz, D_2O, pre-saturated) of compound **42**. Solvent peak at 4.79 ppm.

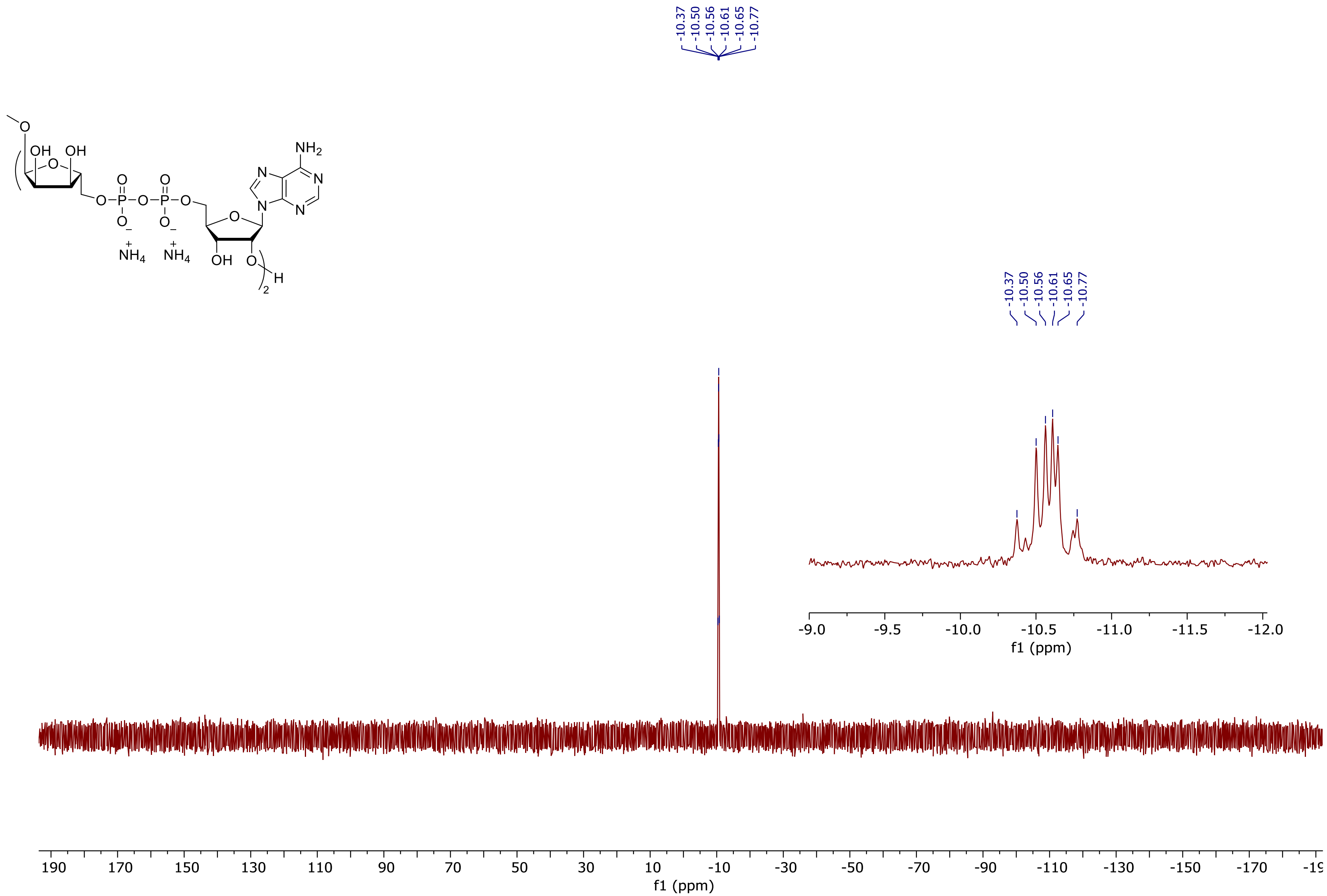

^{31}**P-NMR** (162 MHz, D_2O) of compound **42**.

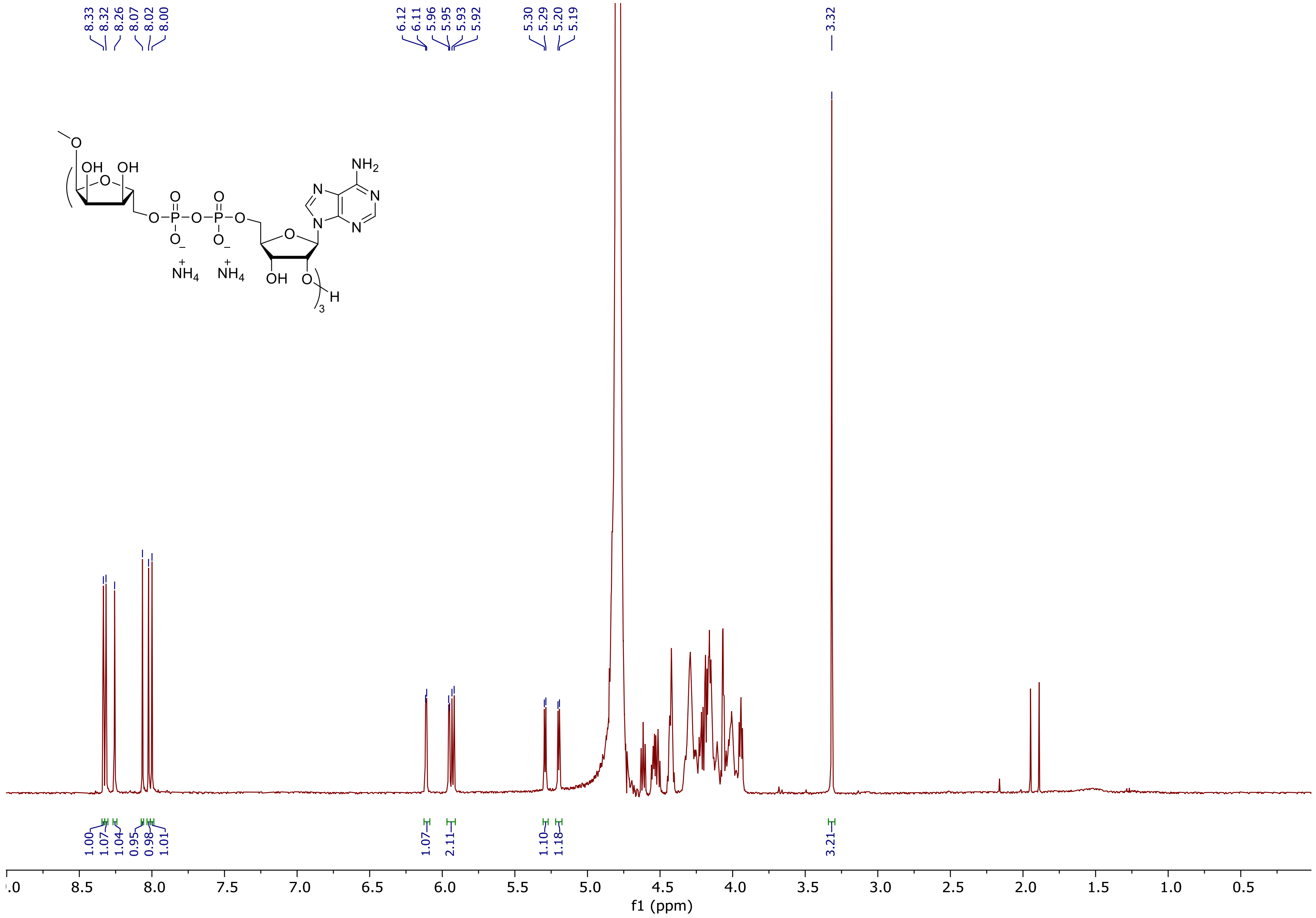

^{1}**H-NMR** (400 MHz, D_2O) of compound **43**. Solvent peak at 4.79 ppm.

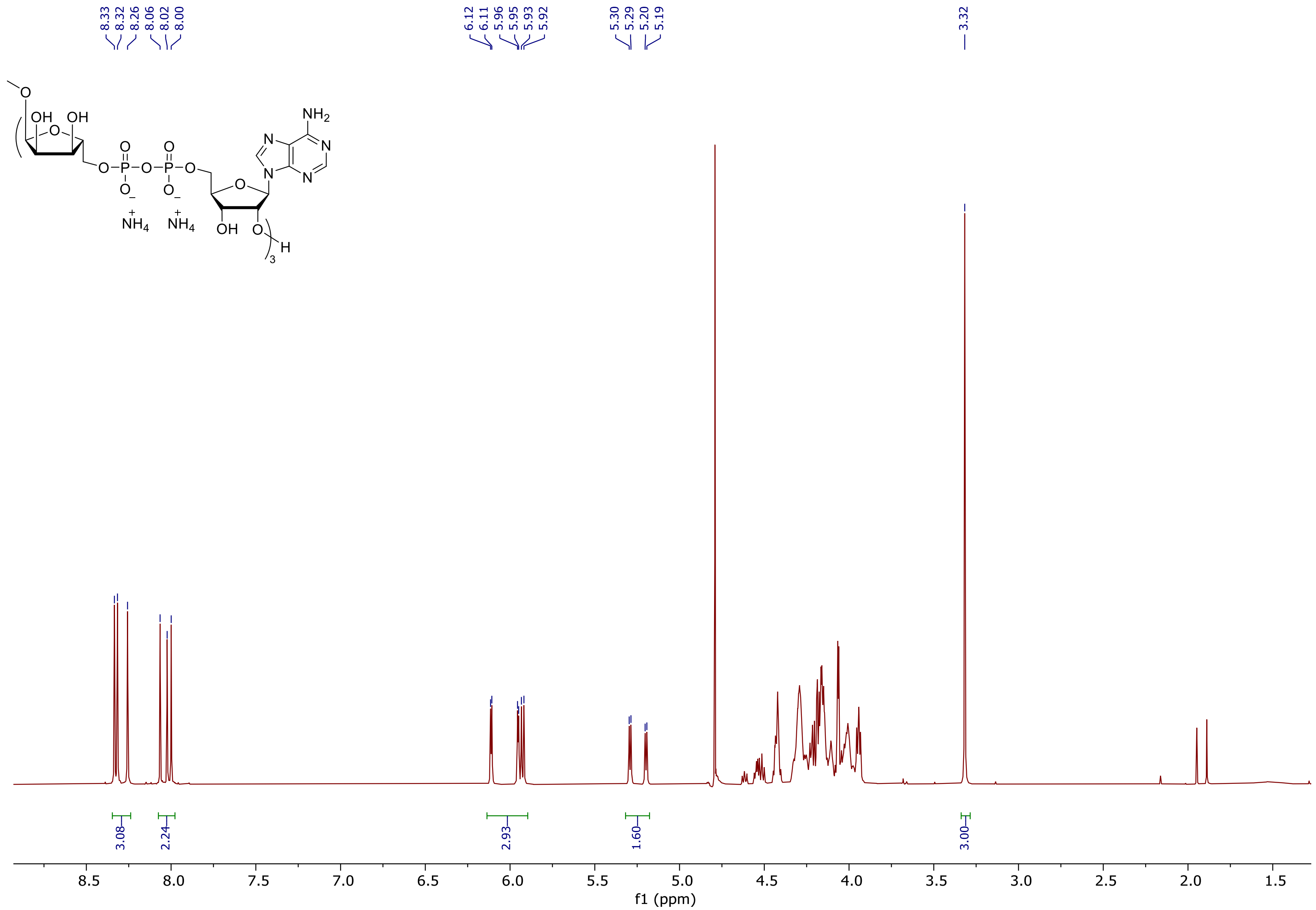

^{1}H-NMR (400 MHz, D_2O, pre-saturated) of compound **43**. Solvent peak at 4.79 ppm.

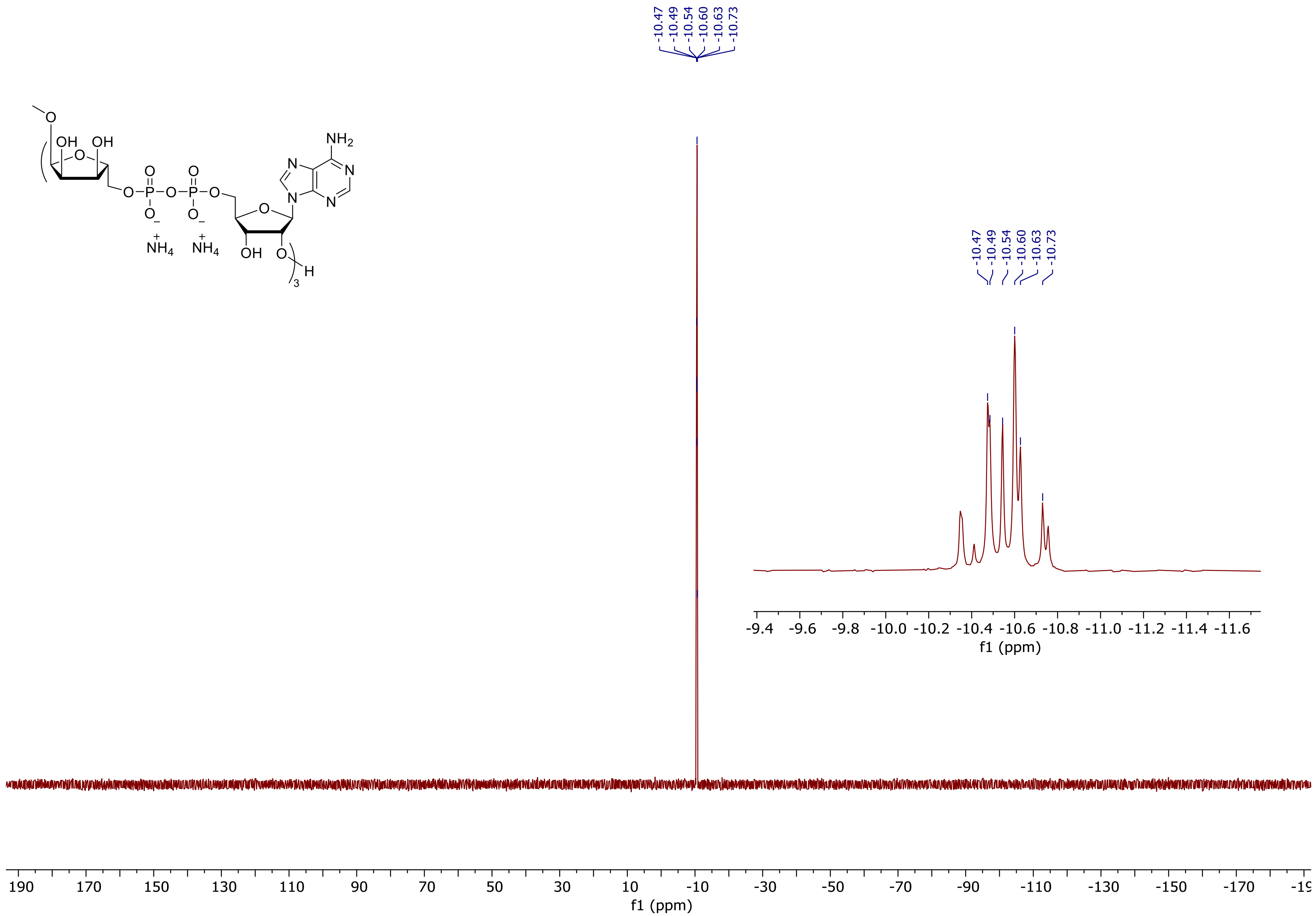

^{31}P-NMR (162 MHz, D_2O) of compound **43**.

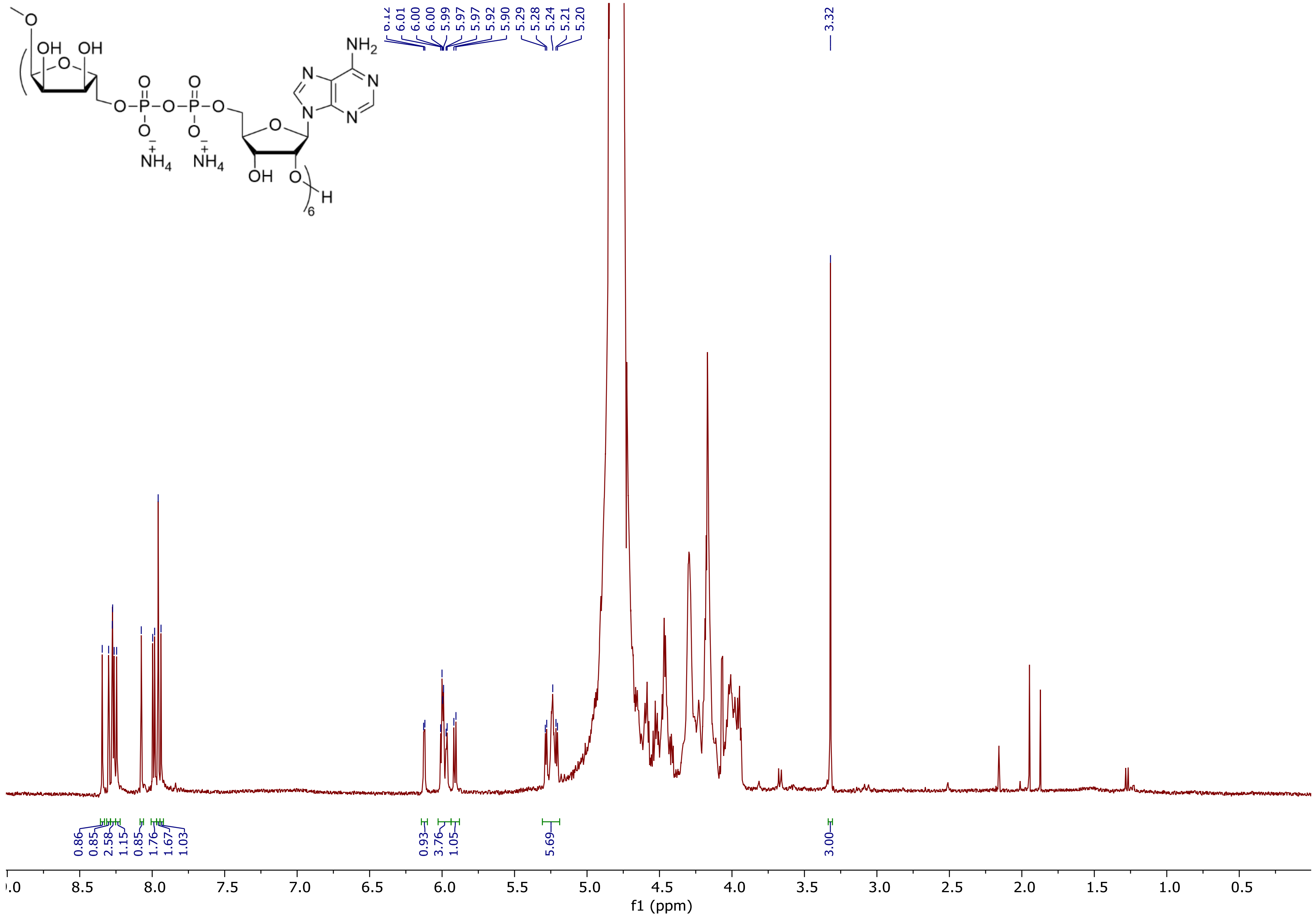

[1]**H-NMR** (400 MHz, D_2O) of compound **44**. Solvent peak at 4.79 ppm.

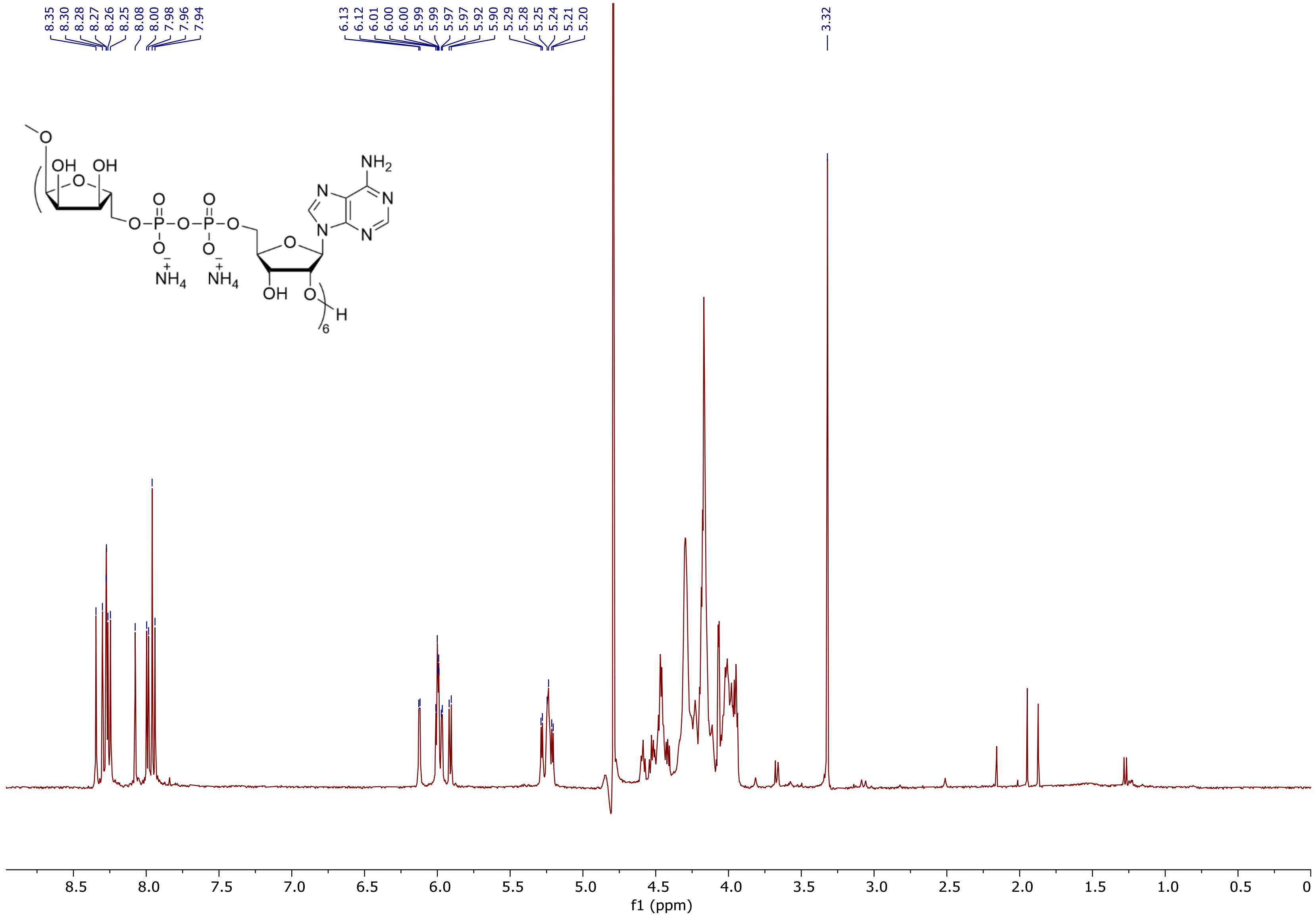

^{1}H-NMR (400 MHz, D_2O, pre-saturated) of compound **44**. Solvent peak at 4.79 ppm.

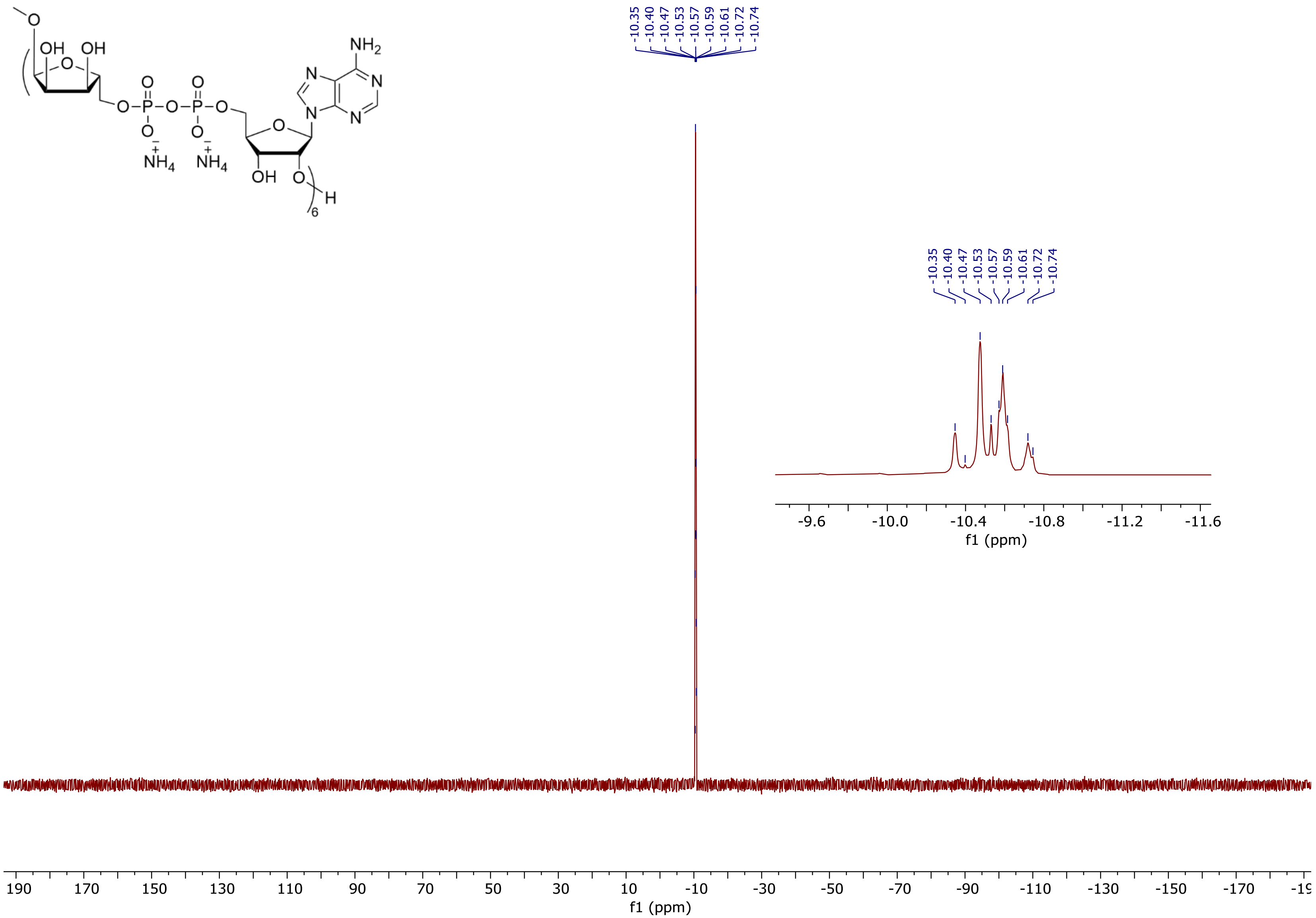

^{31}**P-NMR** (162 MHz, D_2O) of compound **44**.

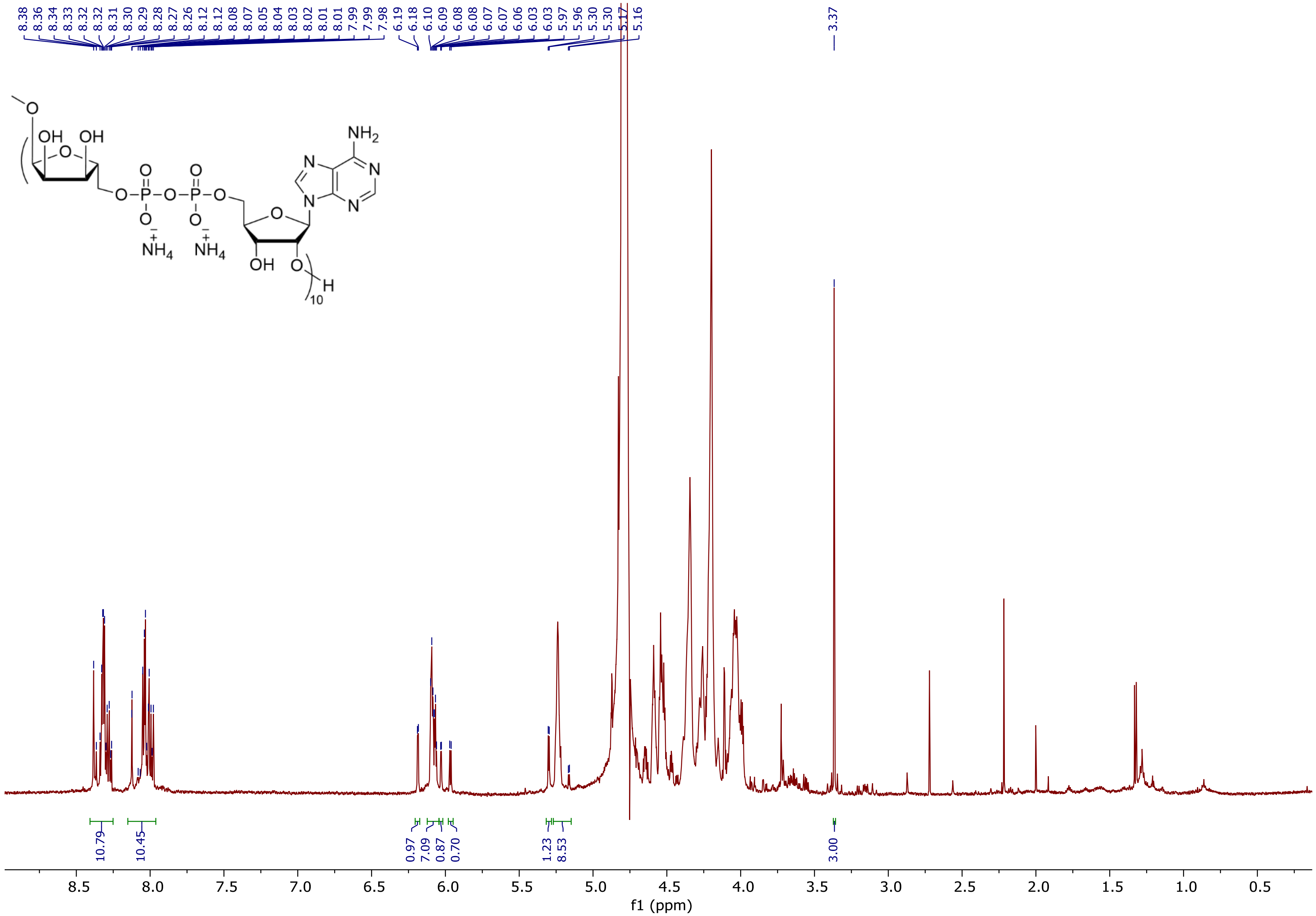

^{1}H-NMR (400 MHz, D_2O) of compound **45**. Solvent peak at 4.79 ppm.

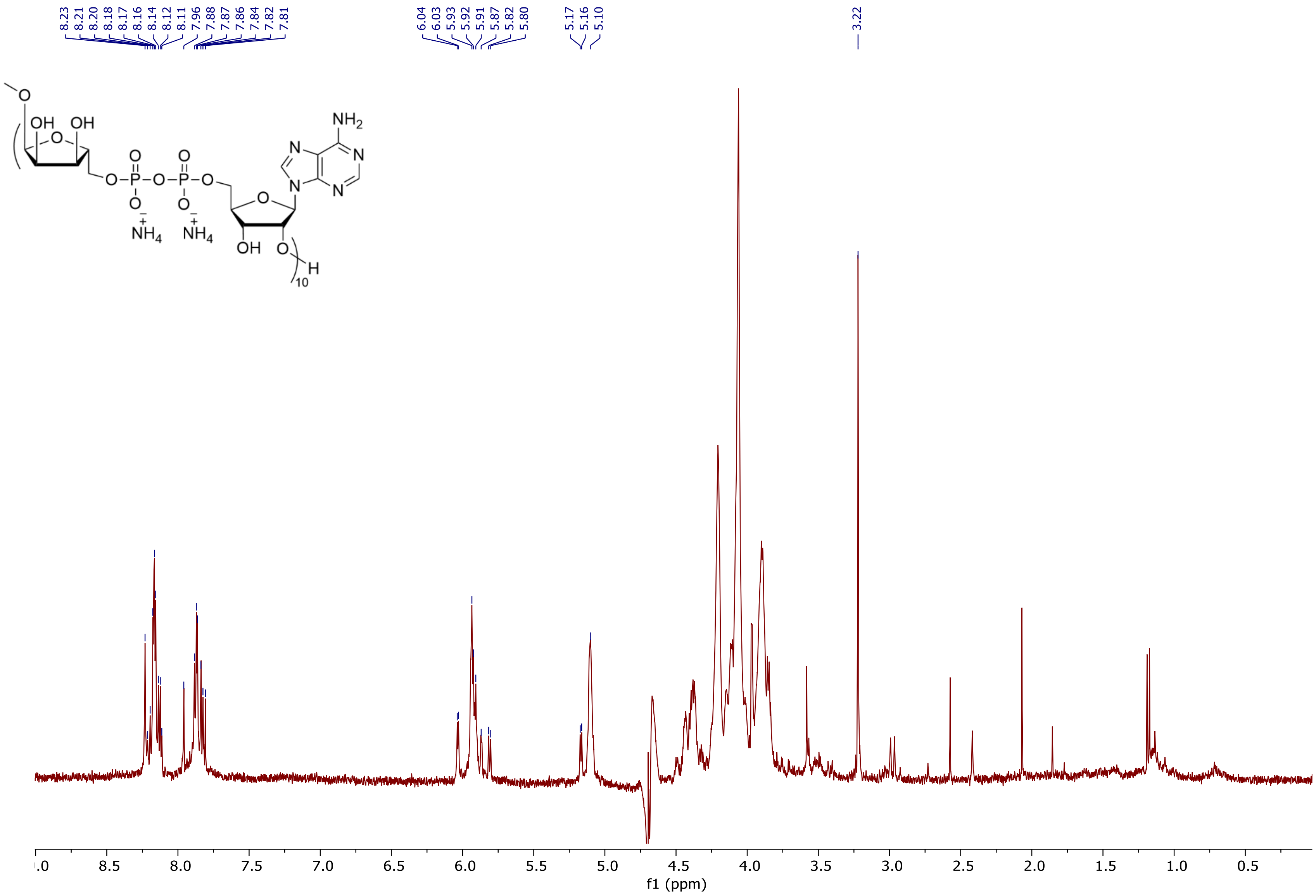

^{1}H-NMR (400 MHz, D_2O, pre-saturated) of compound **45**. Solvent peak at 4.79 ppm.

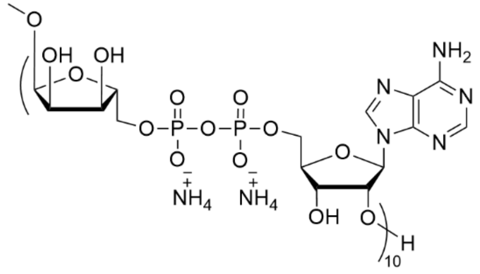

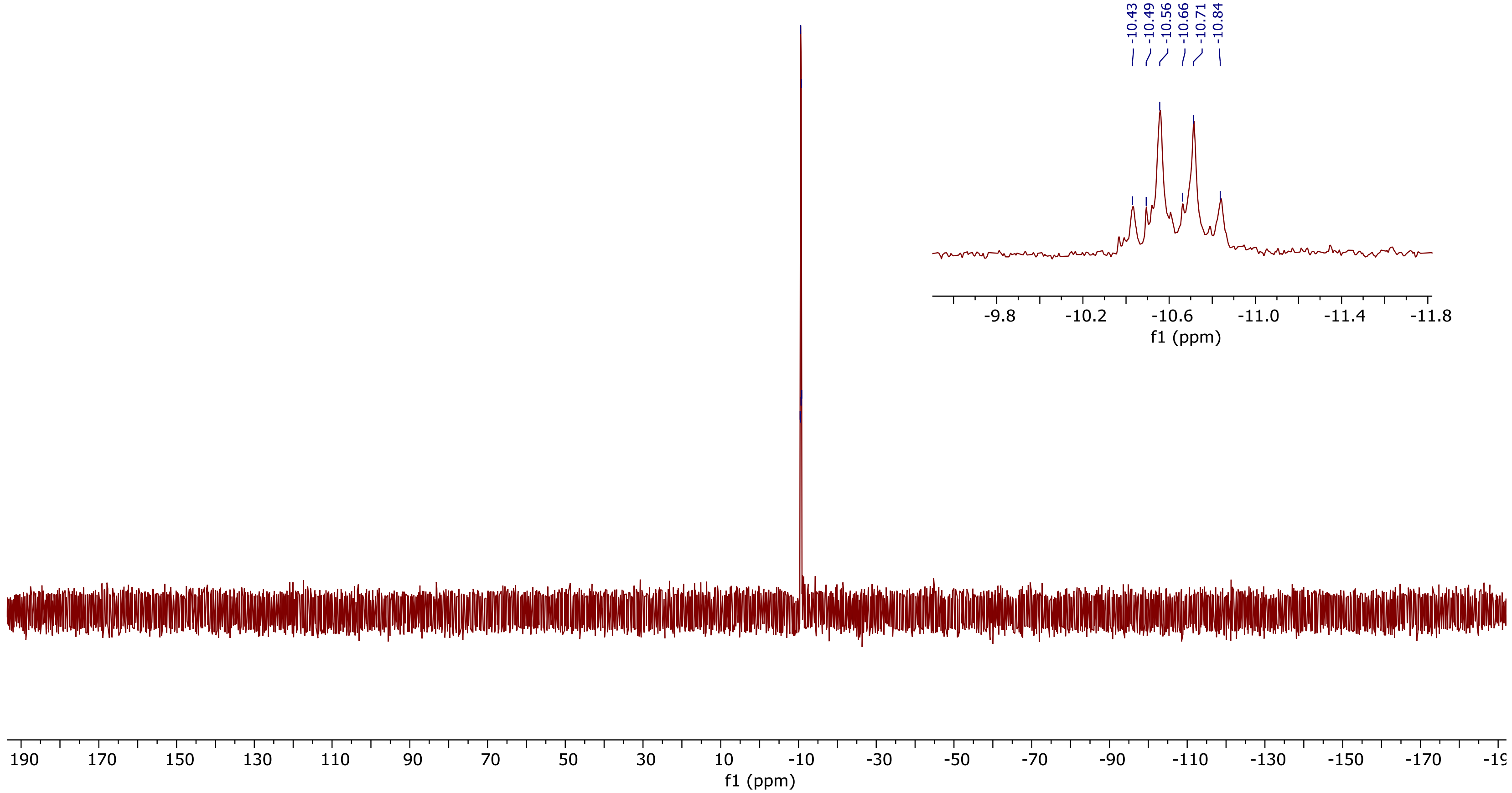

^{31}P-NMR (162 MHz, D_2O) of compound **45**.

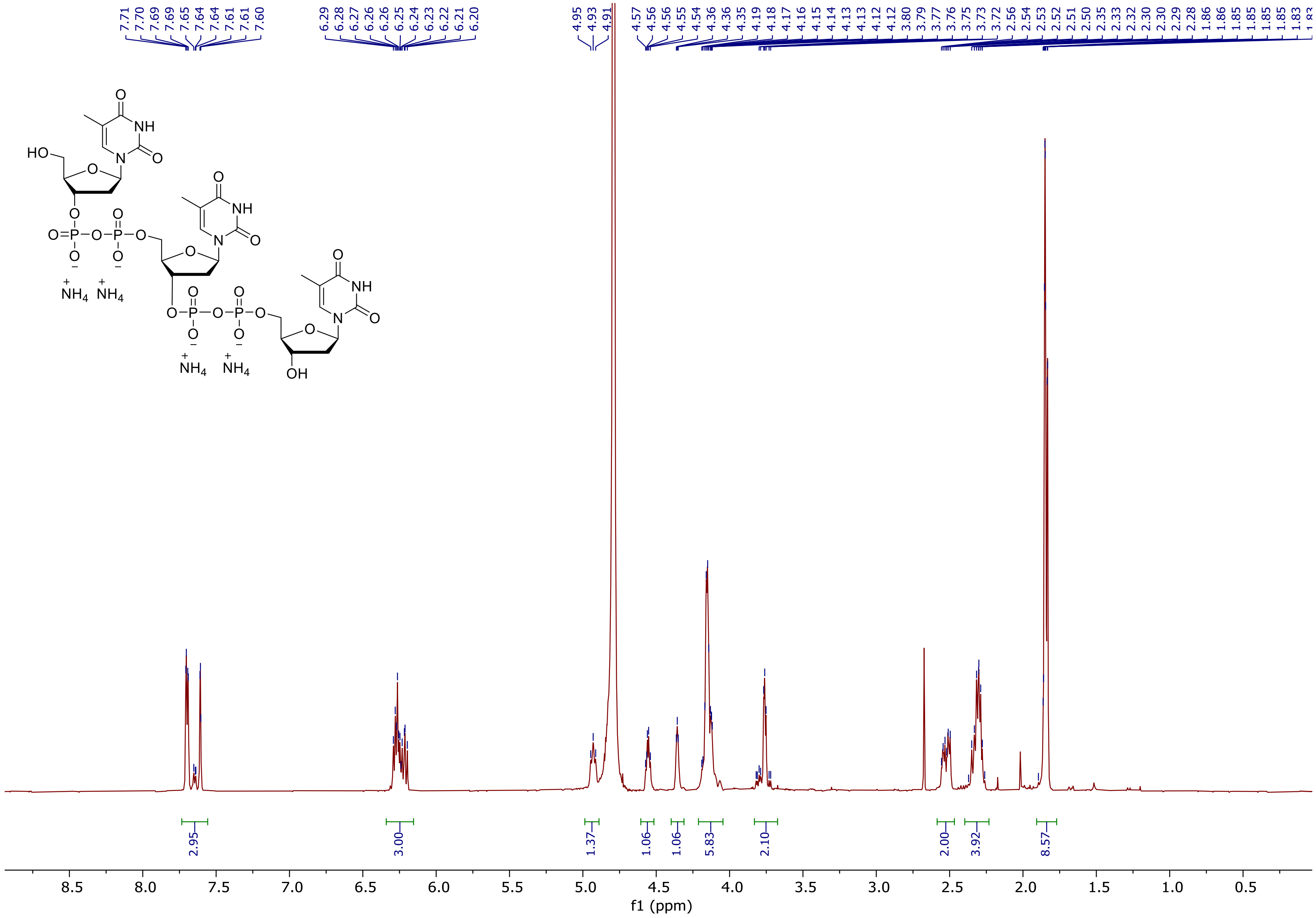

[1]**H-NMR** (400 MHz, D_2O) of compound **26**. Solvent peak at 4.79 ppm.

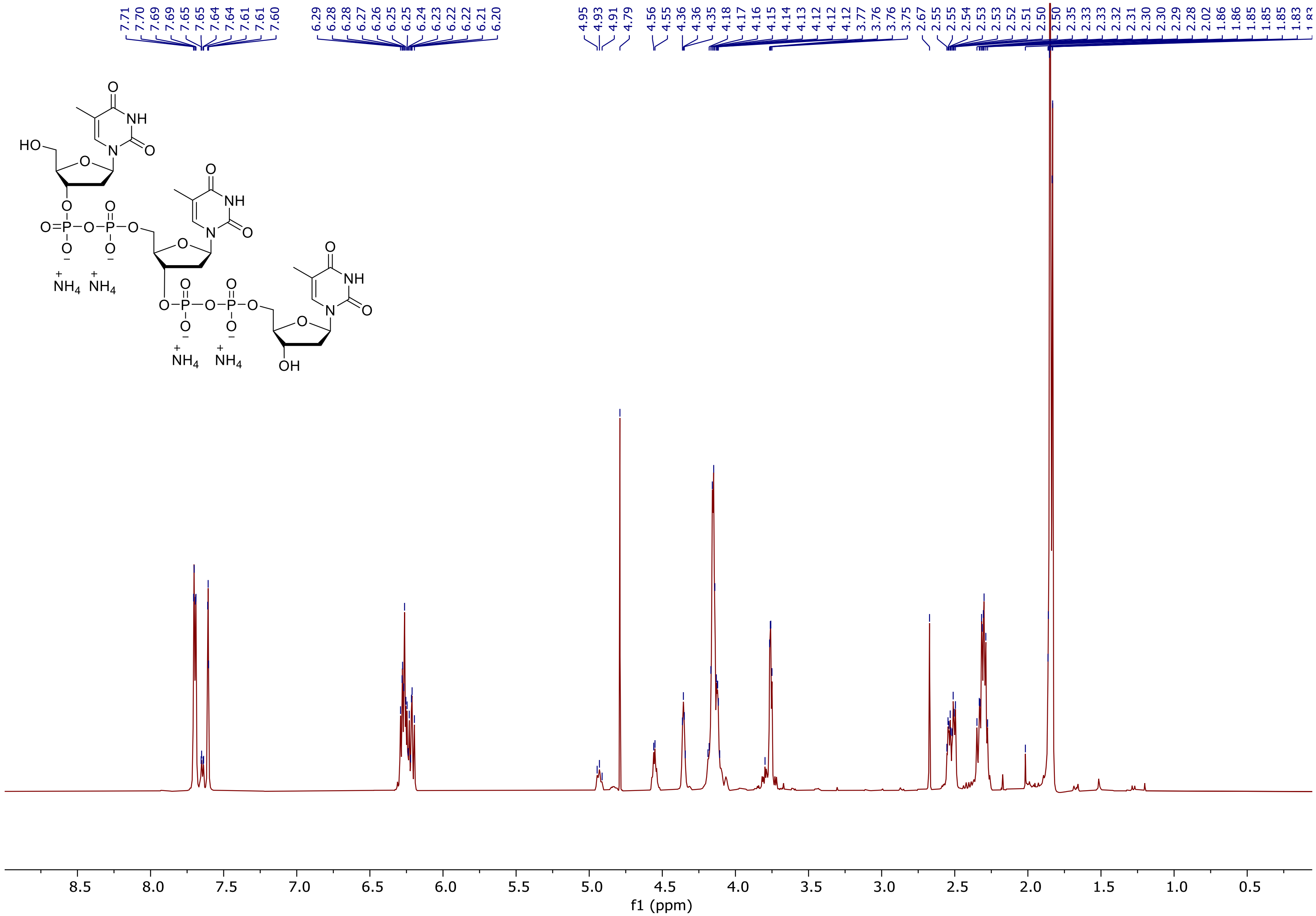

^{1}H-NMR (400 MHz, D_2O, pre-saturated) of compound **46**. Solvent peak at 4.79 ppm.

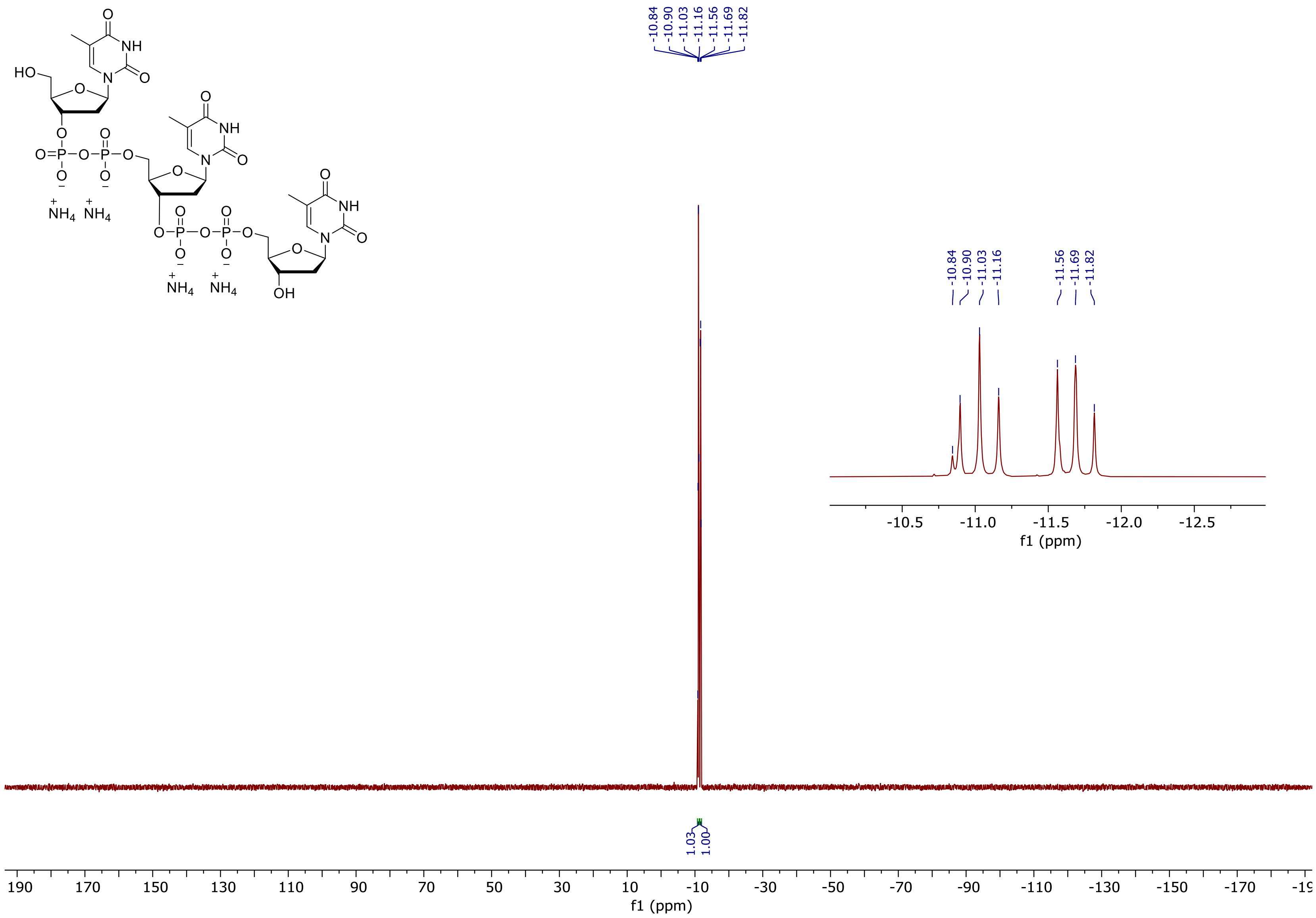

^{31}**P-NMR** (162 MHz, D_2O) of compound **45**.

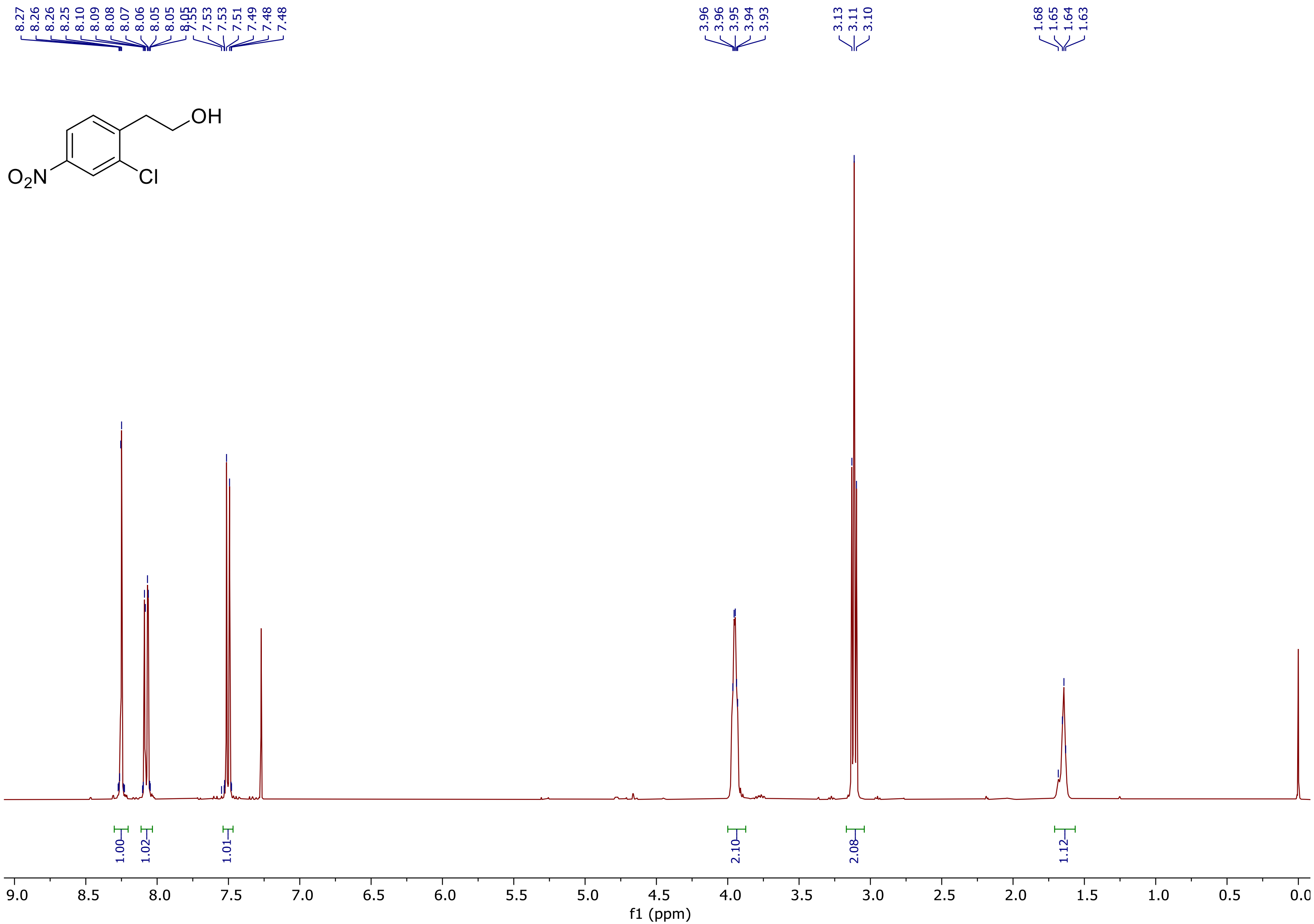

^{1}H-NMR (400 MHz, $CDCl_3$) of compound **50**. Solvent peak at 7.26 ppm.

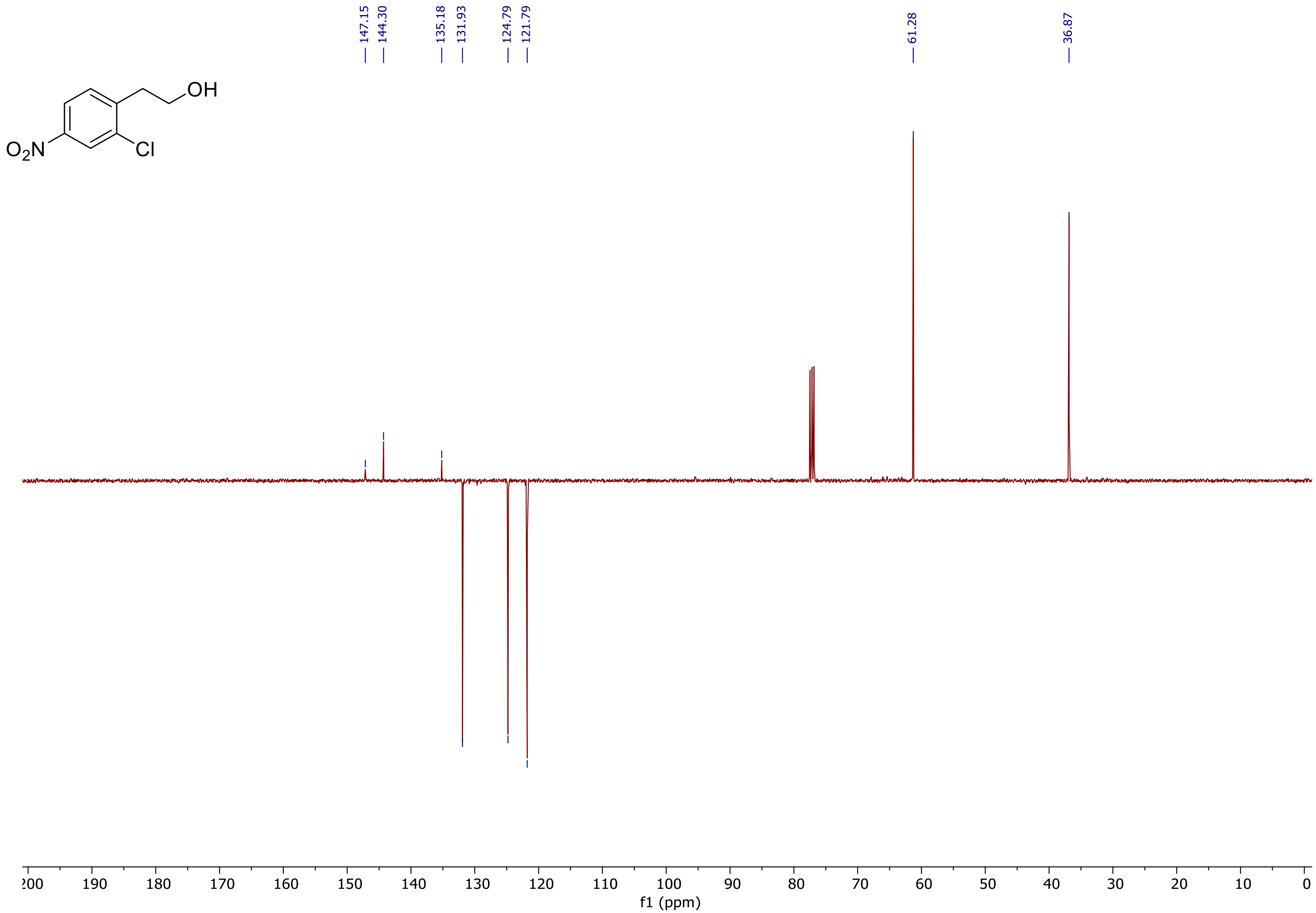

^{13}C-NMR (101 MHz, $CDCl_3$) of compound **50**. Solvent peak at 77.16 ppm.

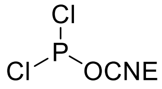

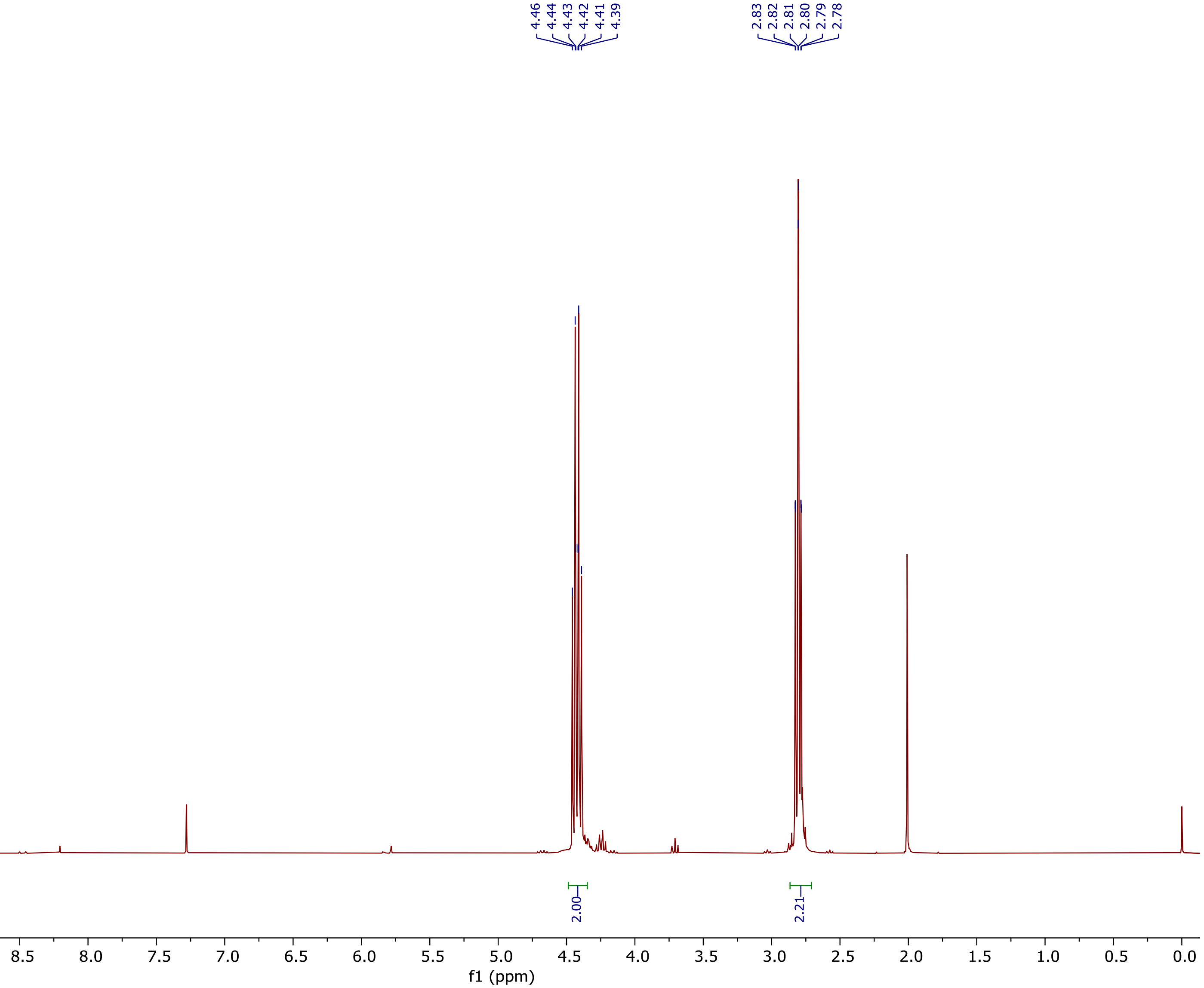

^{1}H-NMR (300 MHz, $CDCl_3$) of compound **51**. Solvent peak at 7.26 ppm.

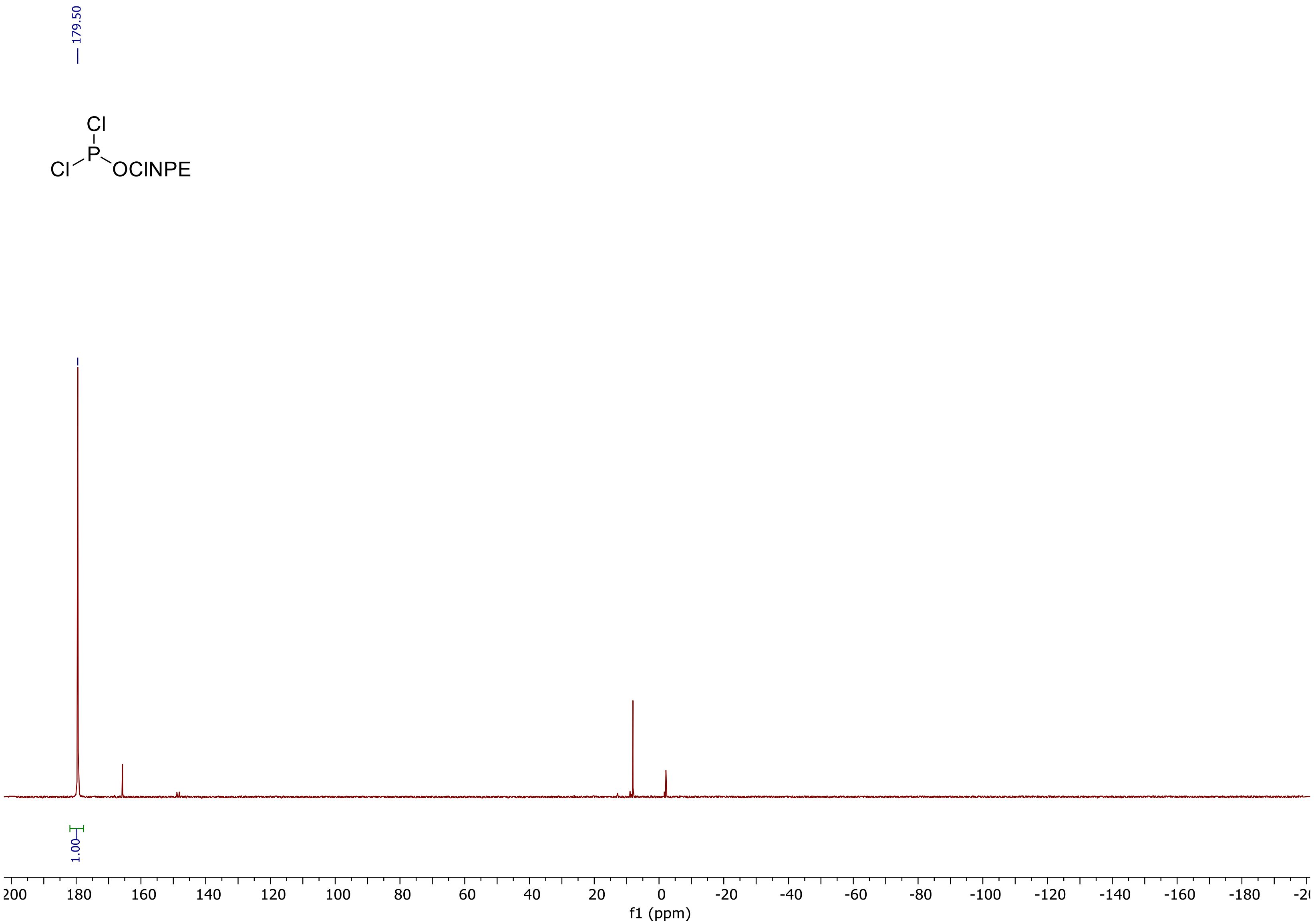

^{31}**P-NMR** (121 MHz, $CDCl_3$) of compound **51**.

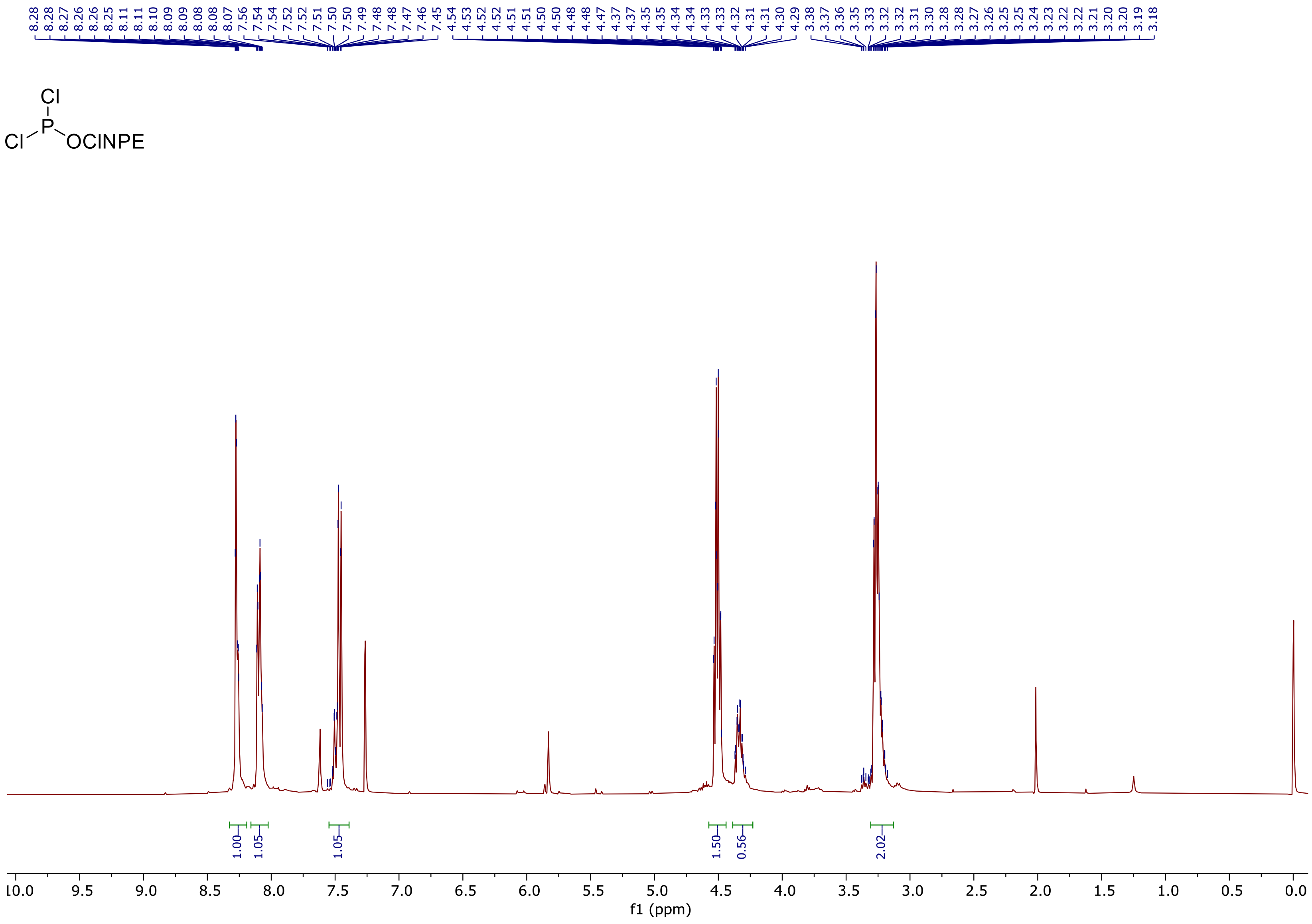

[1]**H-NMR** (400 MHz, $CDCl_3$) of compound **52**. Solvent peak at 7.26 ppm.

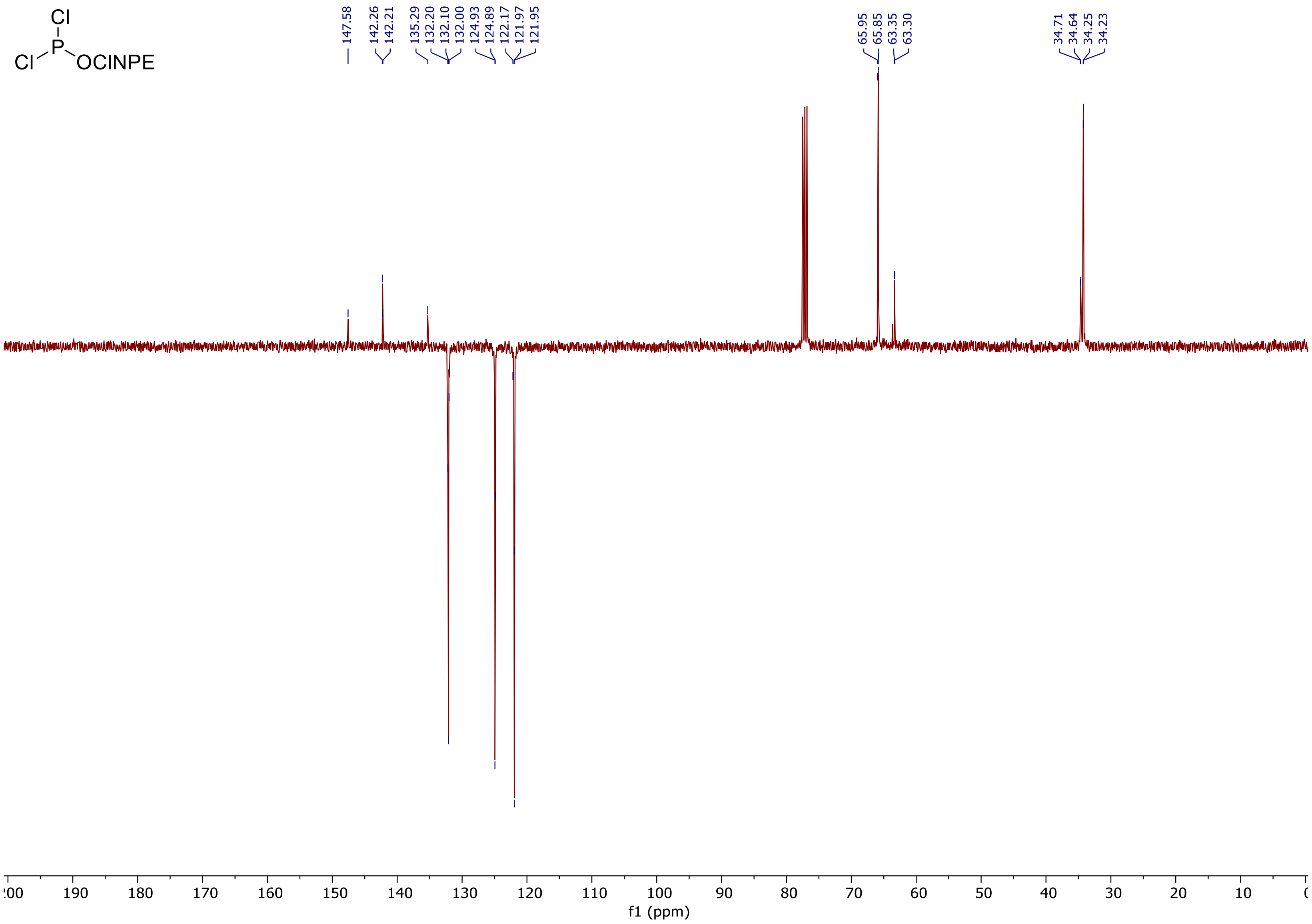

^{13}**C-NMR** (101 MHz, $CDCl_3$) of compound **52**. Solvent peak at 77.16 ppm.

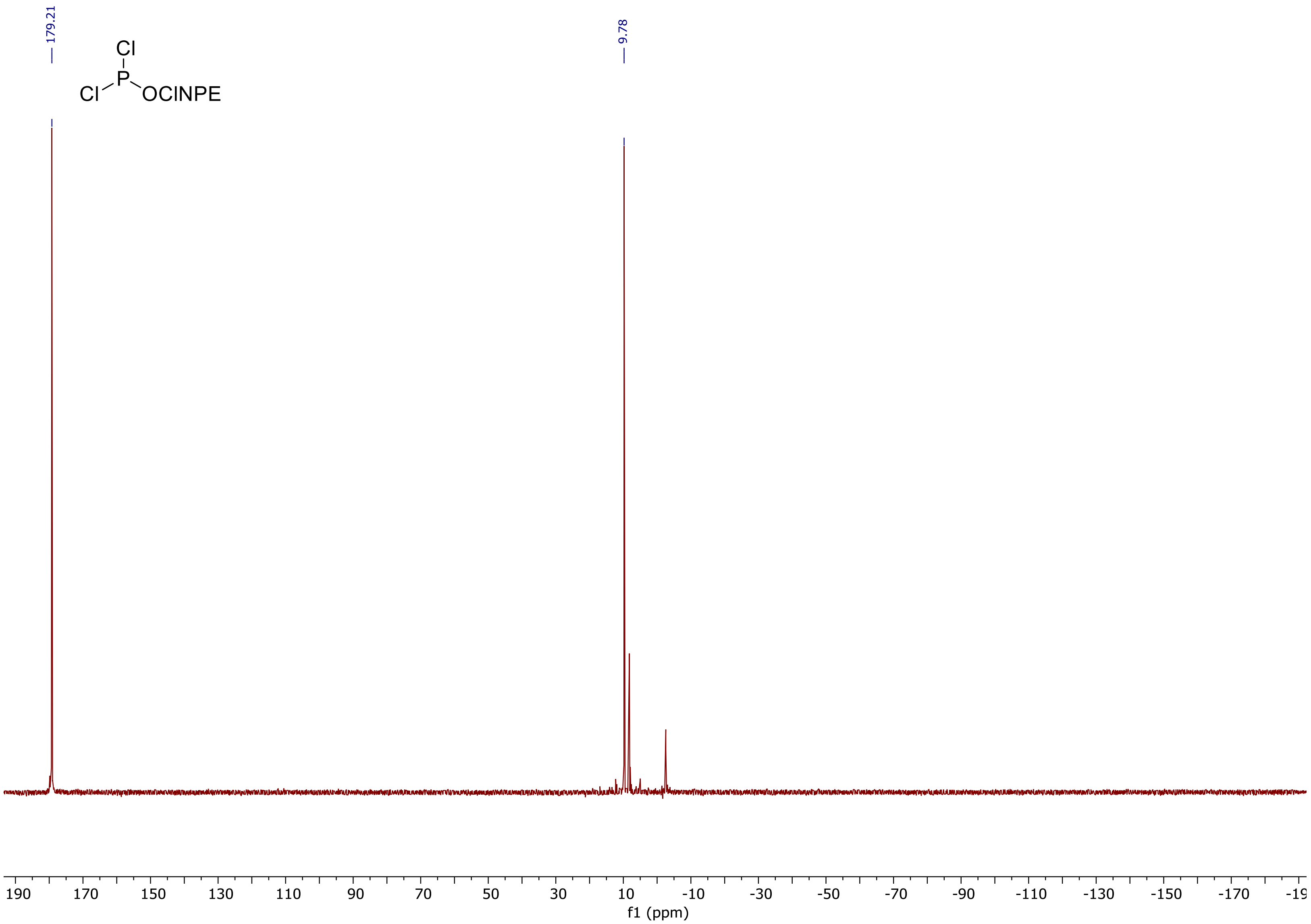

³¹P-NMR (162 MHz, $CDCl_3$) of compound **52**.

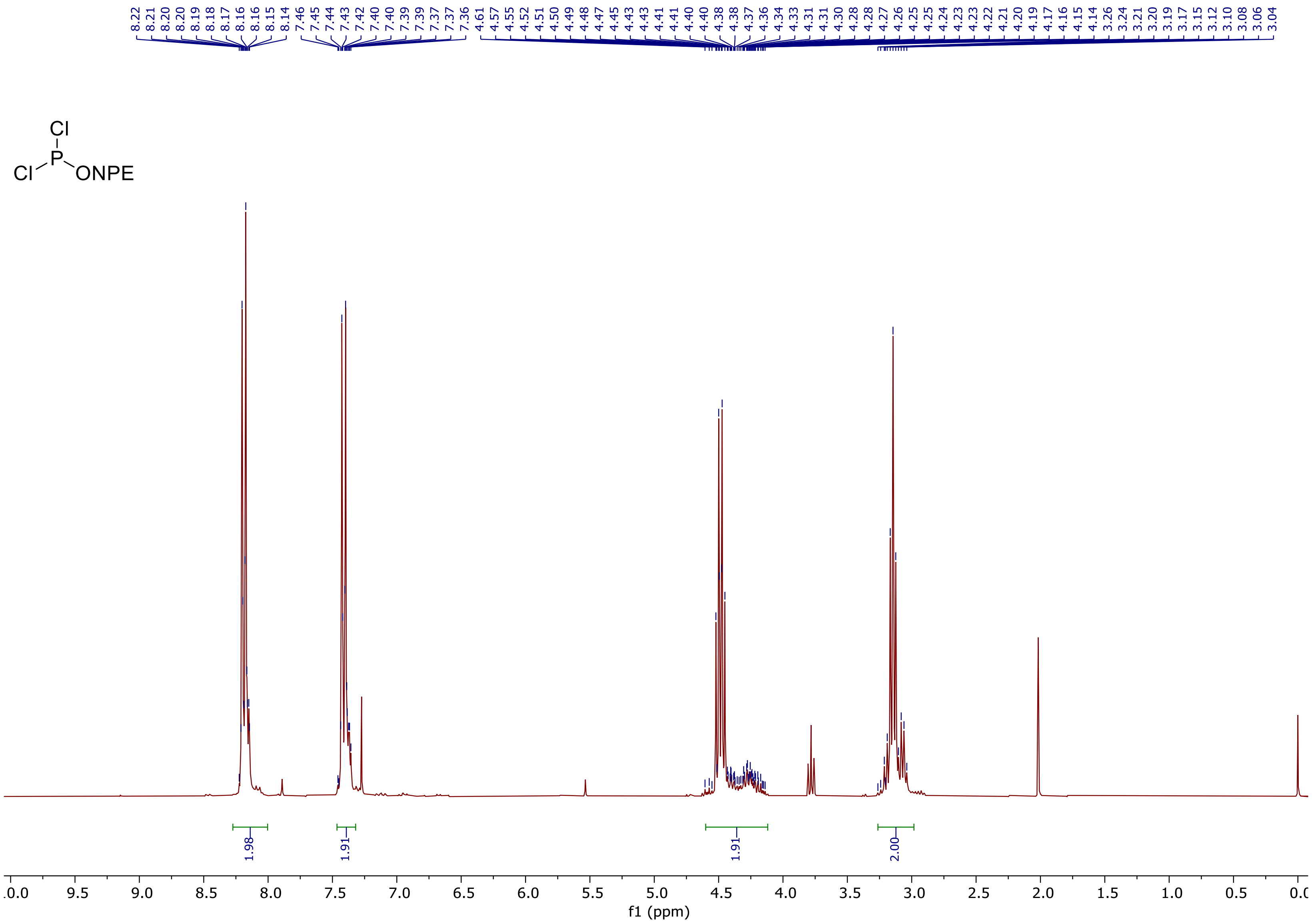

[1]**H-NMR** (300 MHz, $CDCl_3$) of compound **53**. Solvent peak at 7.26 ppm.

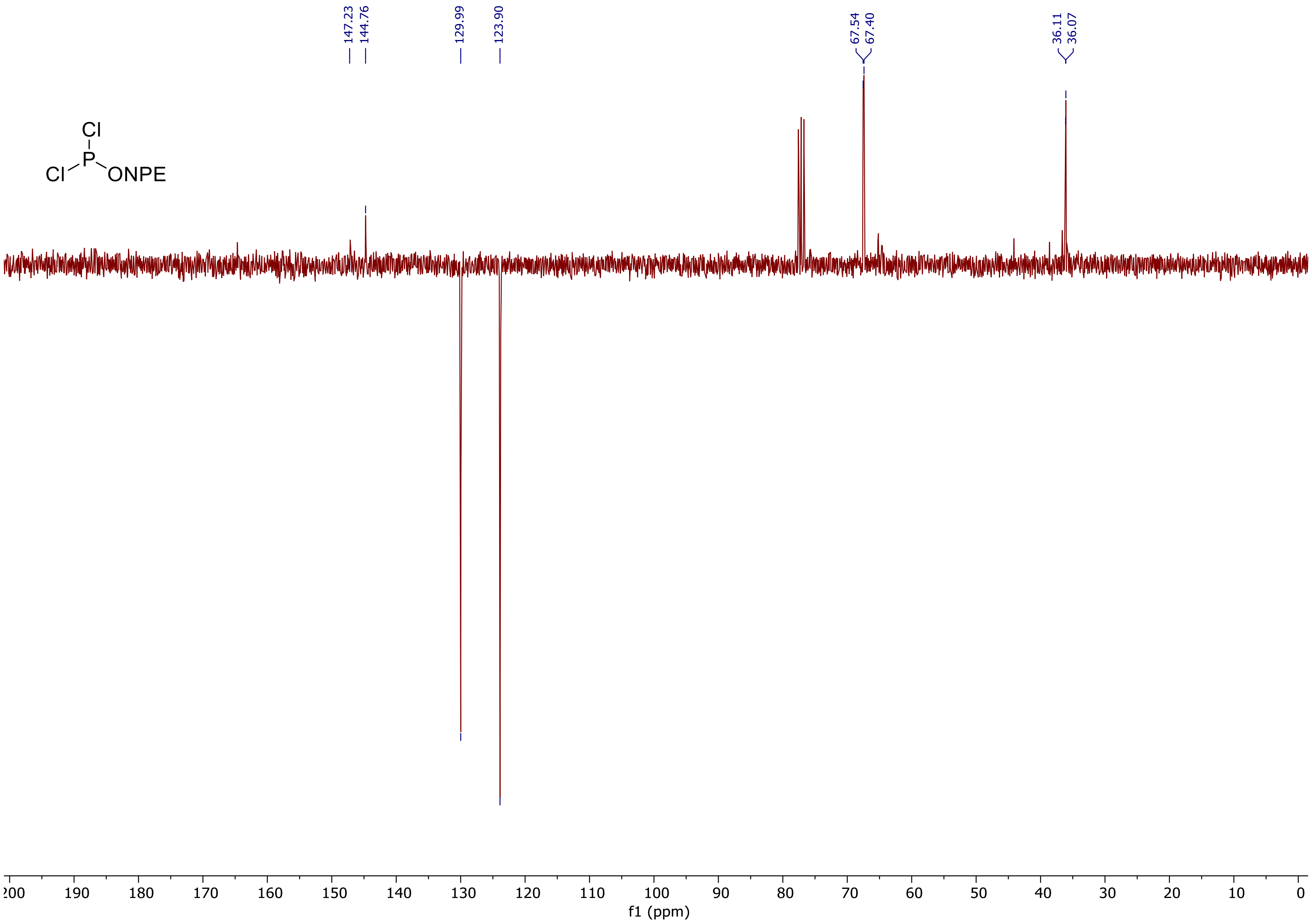

^{13}C-NMR (75 MHz, $CDCl_3$) of compound **53**. Solvent peak at 77.16 ppm.

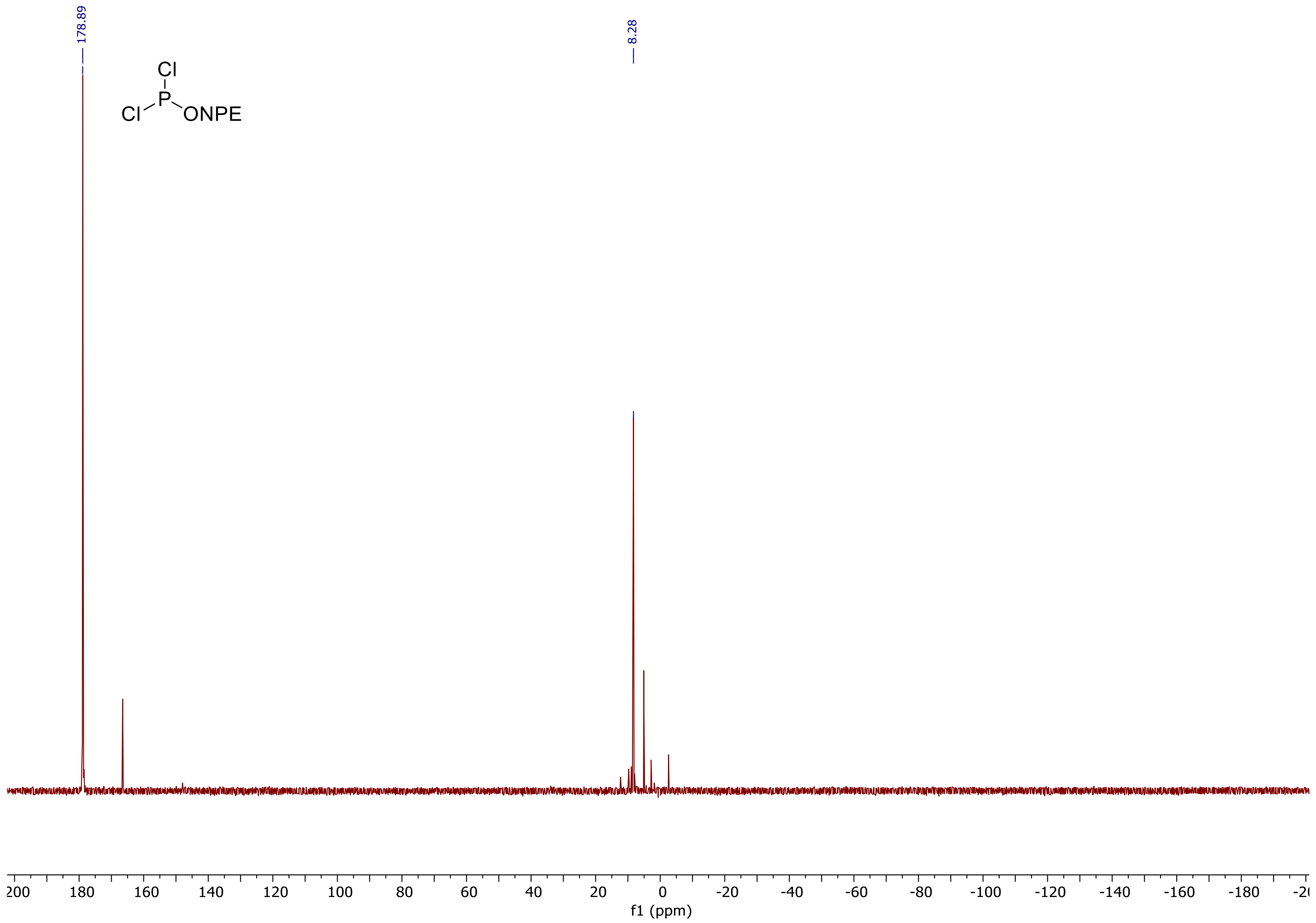

^{31}**P-NMR** (121 MHz, $CDCl_3$) of compound **53**.

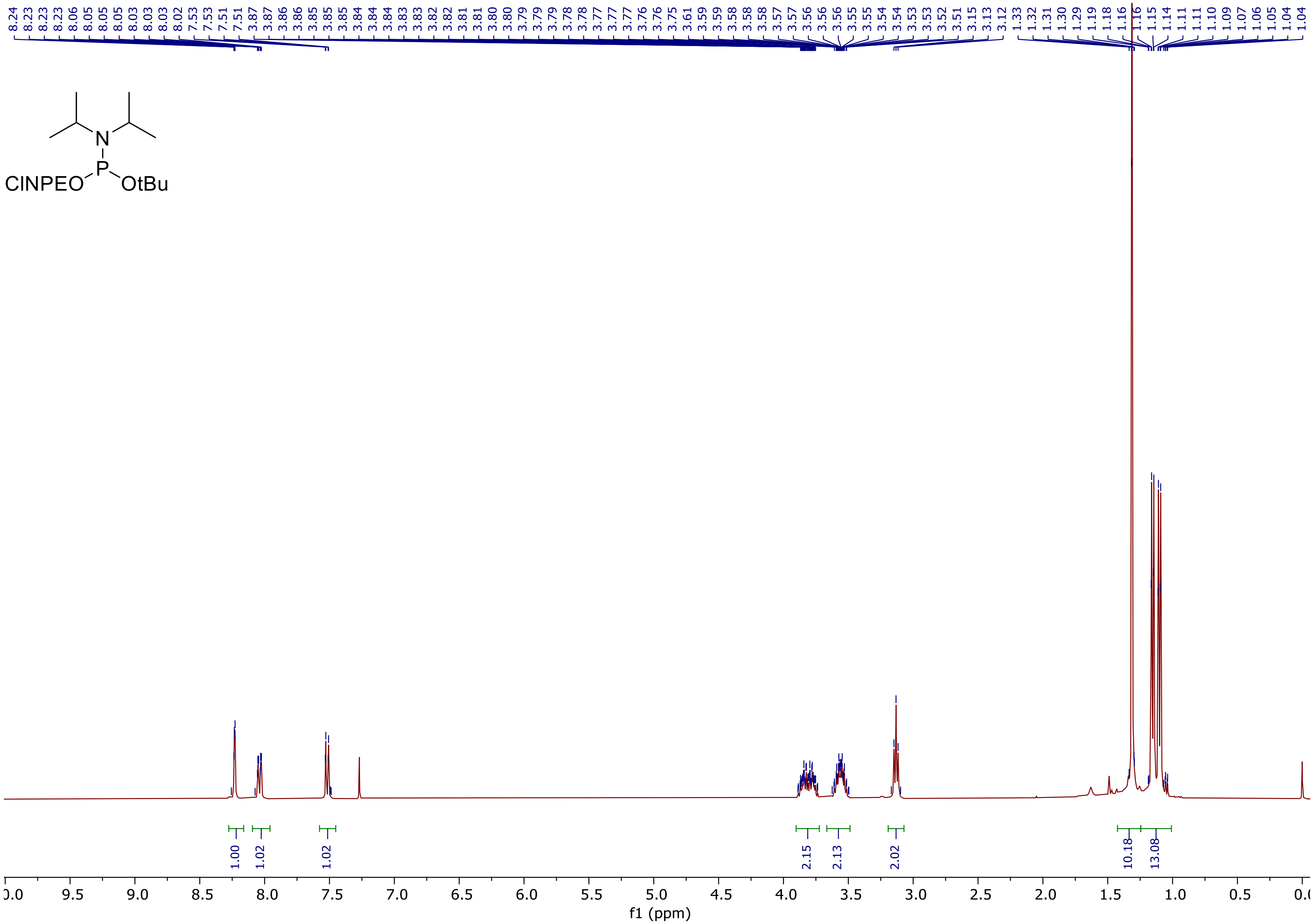

¹H-NMR (400 MHz, $CDCl_3$) of compound **34**. Solvent peak at 7.26 ppm.

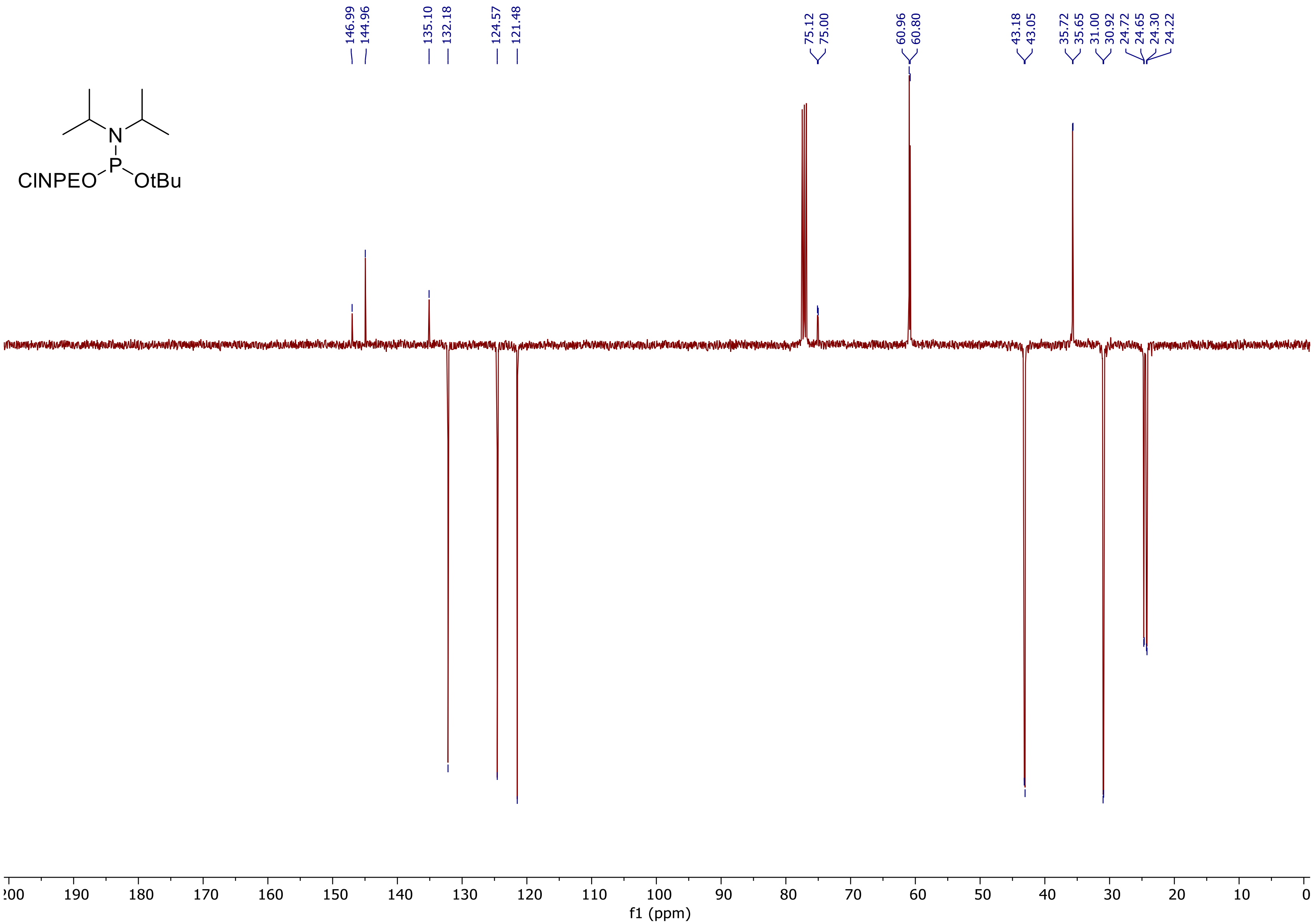

^{13}C-NMR (101 MHz, $CDCl_3$) of compound **34**. Solvent peak at 77.16 ppm.

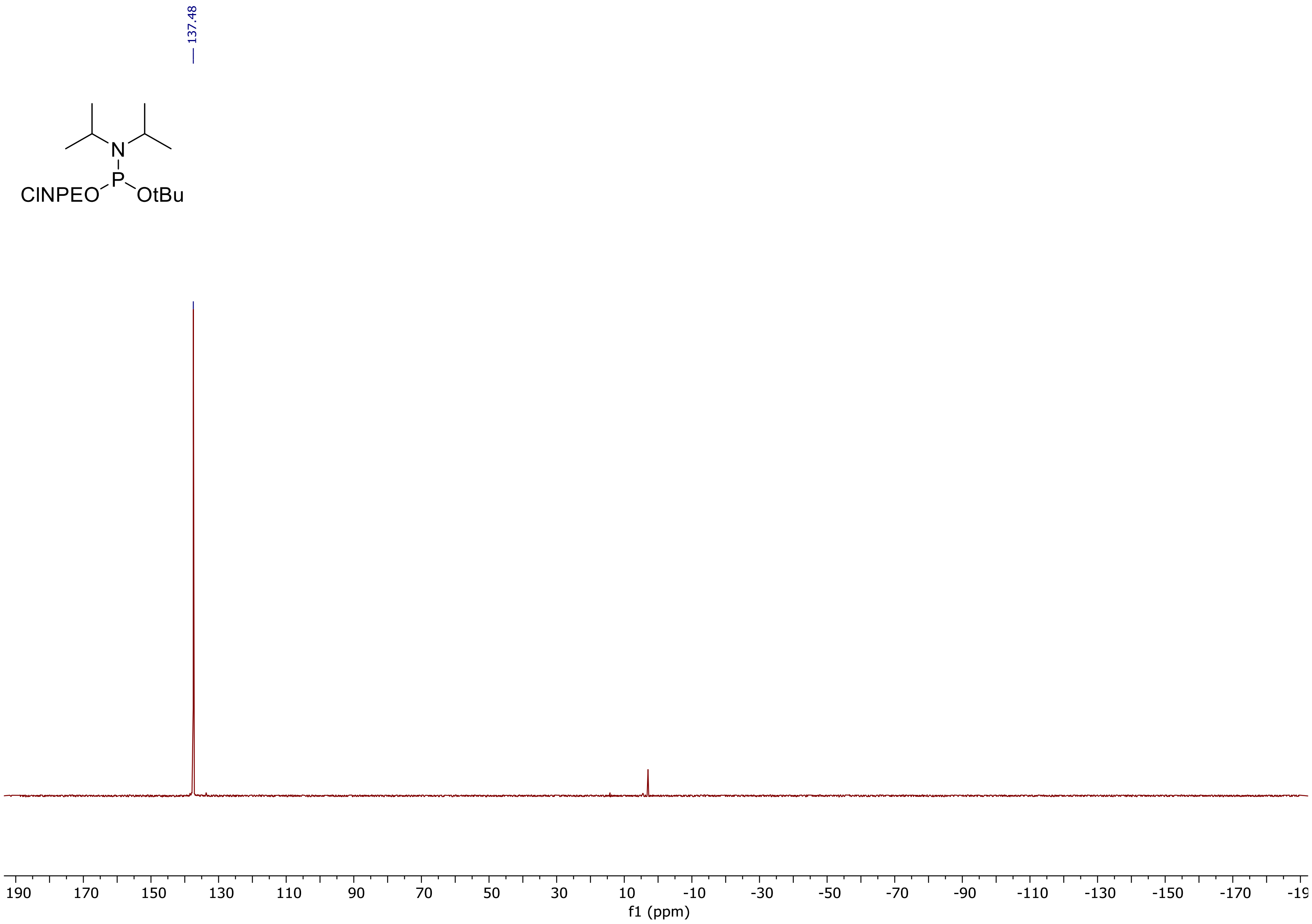

^{31}**P-NMR** (162 MHz, $CDCl_3$) of compound **34**.

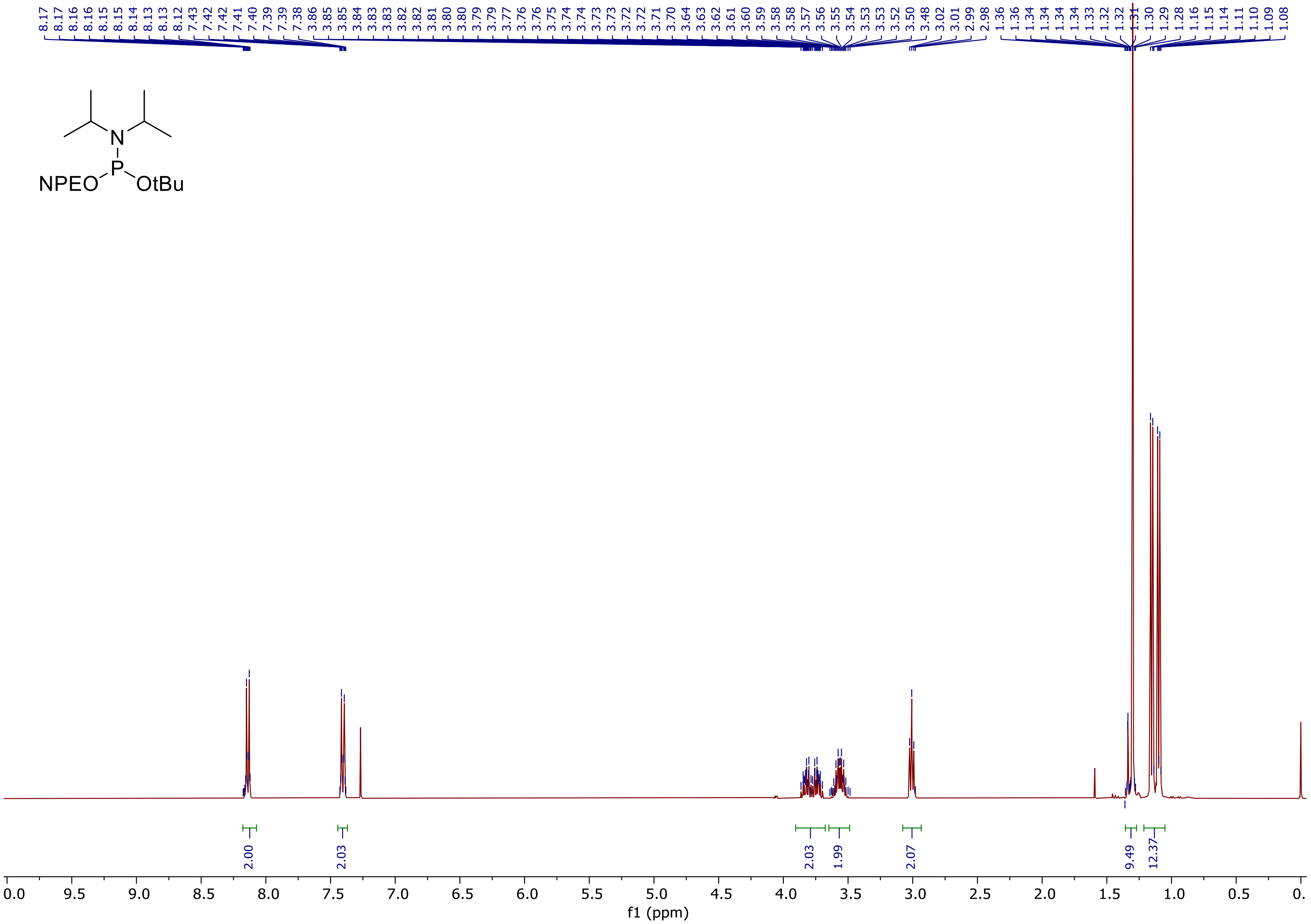

^{1}H-NMR (400 MHz, $CDCl_3$) of compound **55**. Solvent peak at 7.26 ppm.

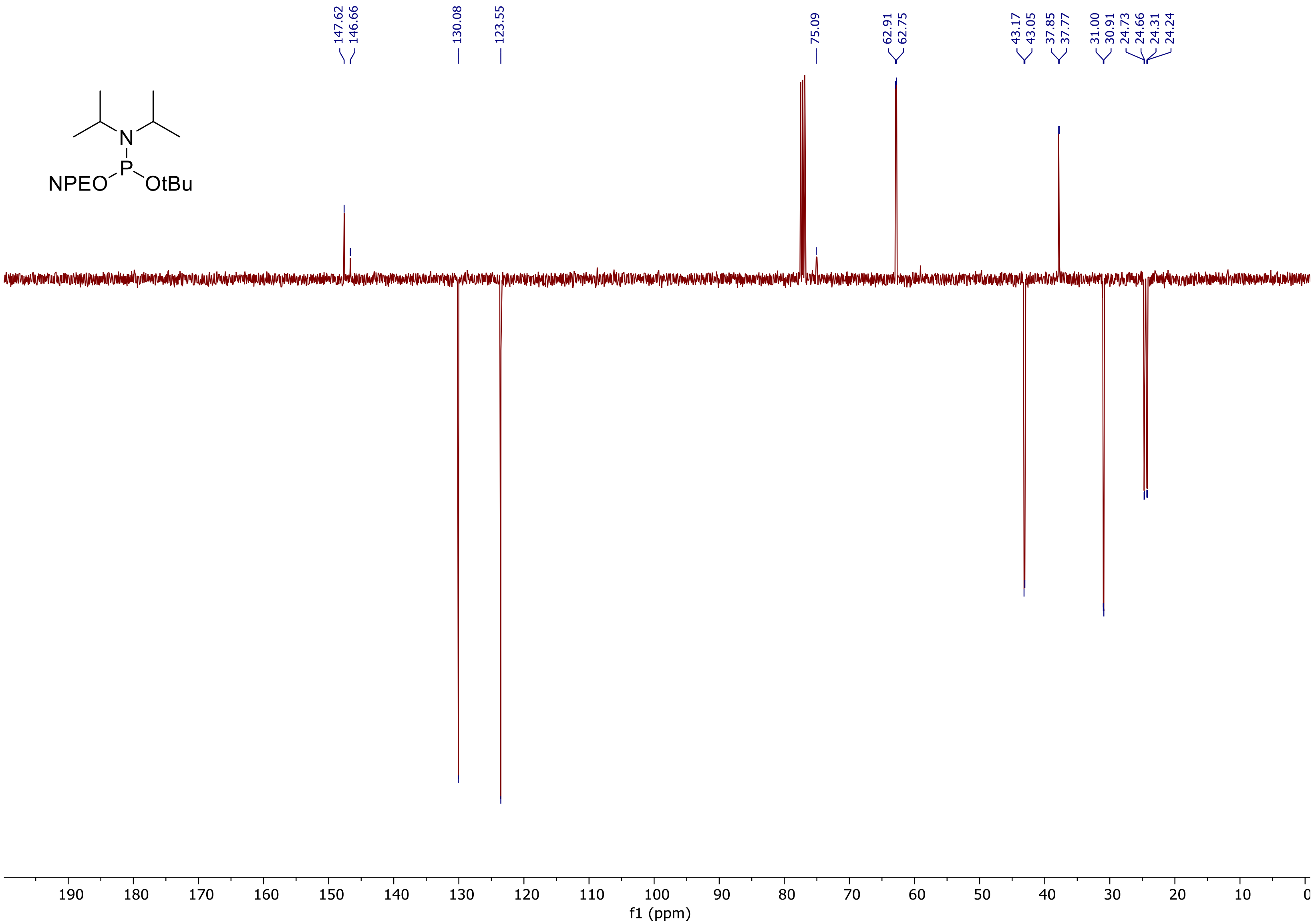

^{13}C-NMR (101 MHz, $CDCl_3$) of compound **55**. Solvent peak at 77.16 ppm.

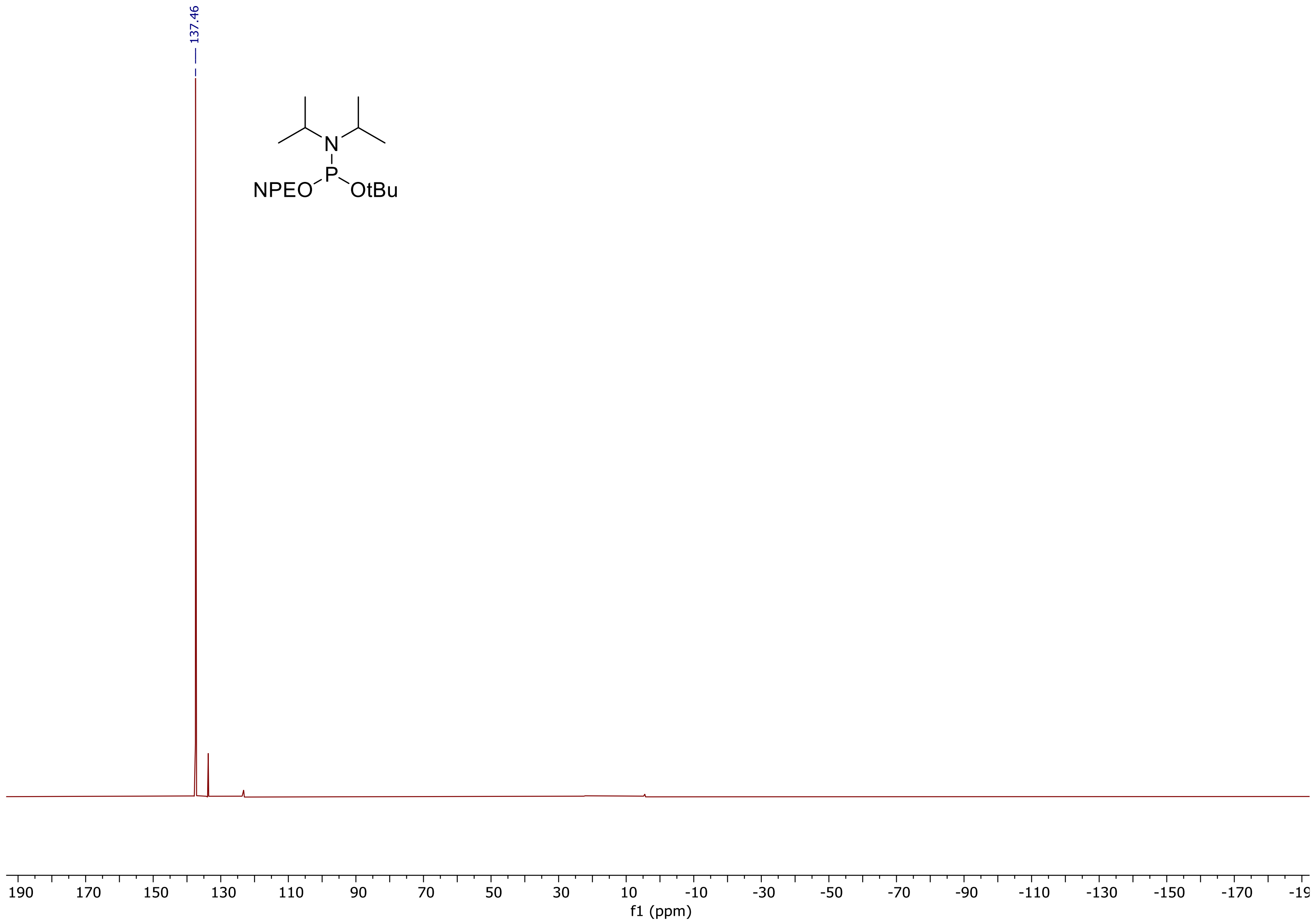

³¹P-NMR (162 MHz, $CDCl_3$) of compound **55**.

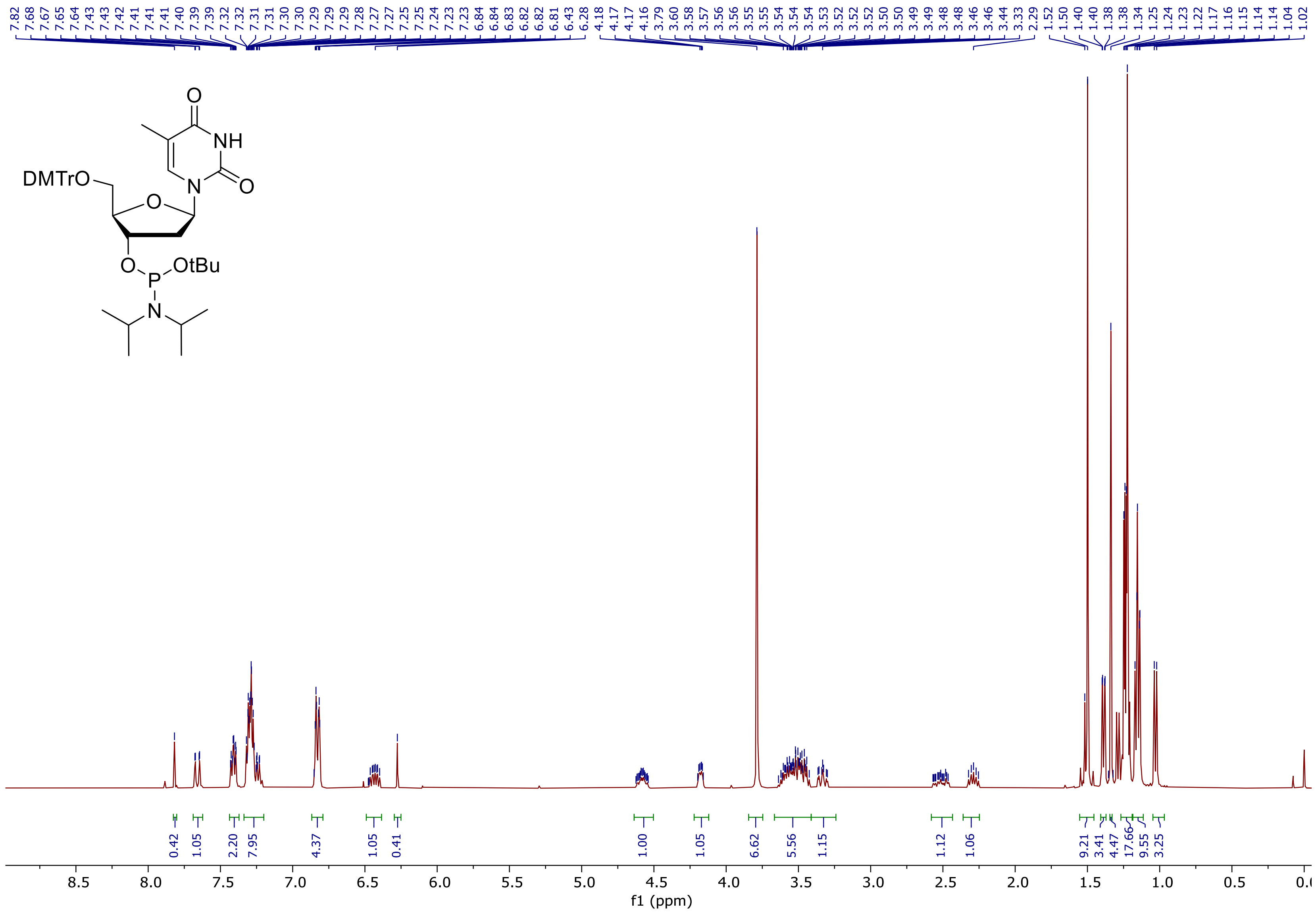

[1]**H-NMR** (400 MHz, $CDCl_3$) of compound **57**. Solvent peak at 7.26 ppm.

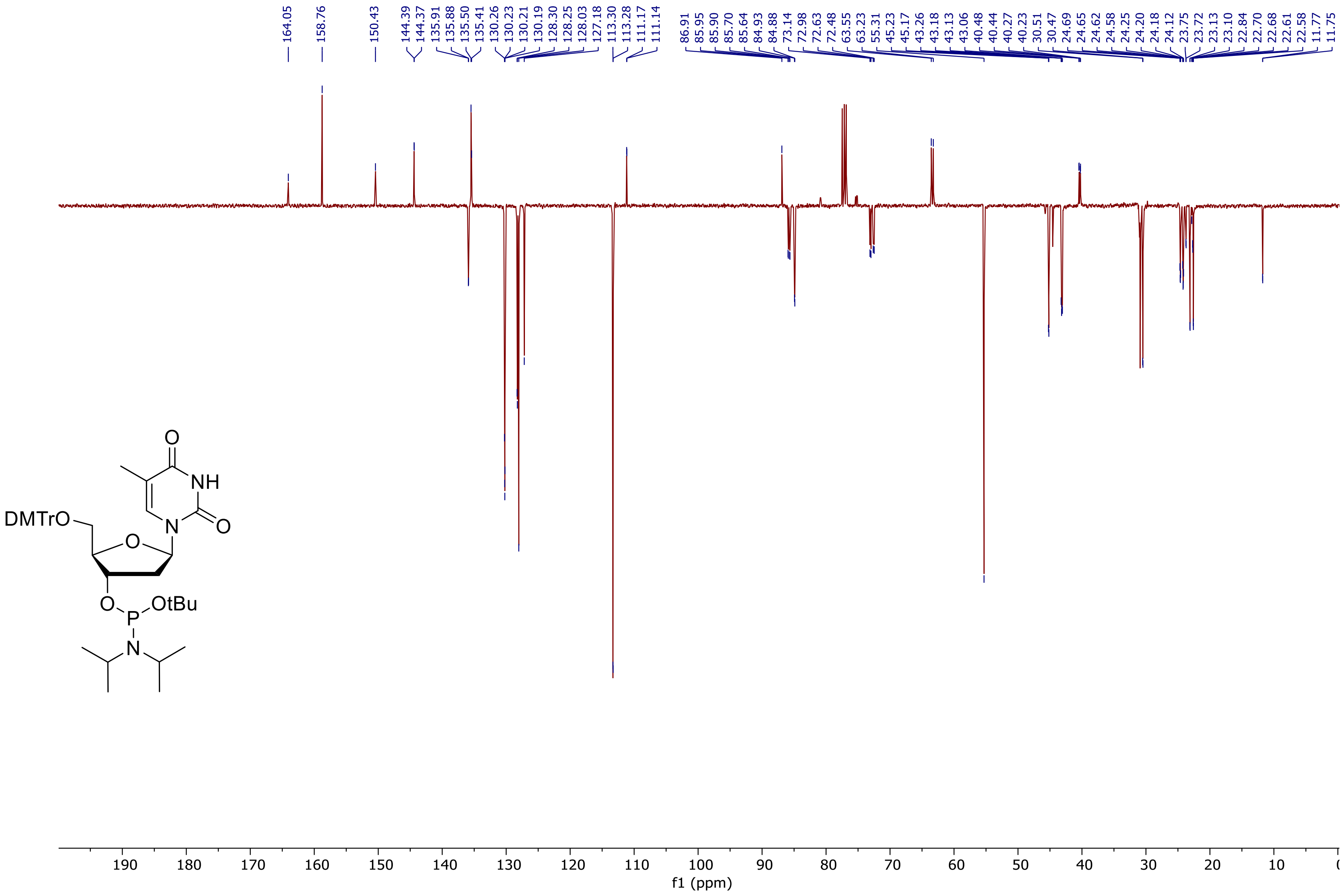

^{13}**C-NMR** (101 MHz, $CDCl_3$) of compound **57**. Solvent peak at 77.16 ppm.

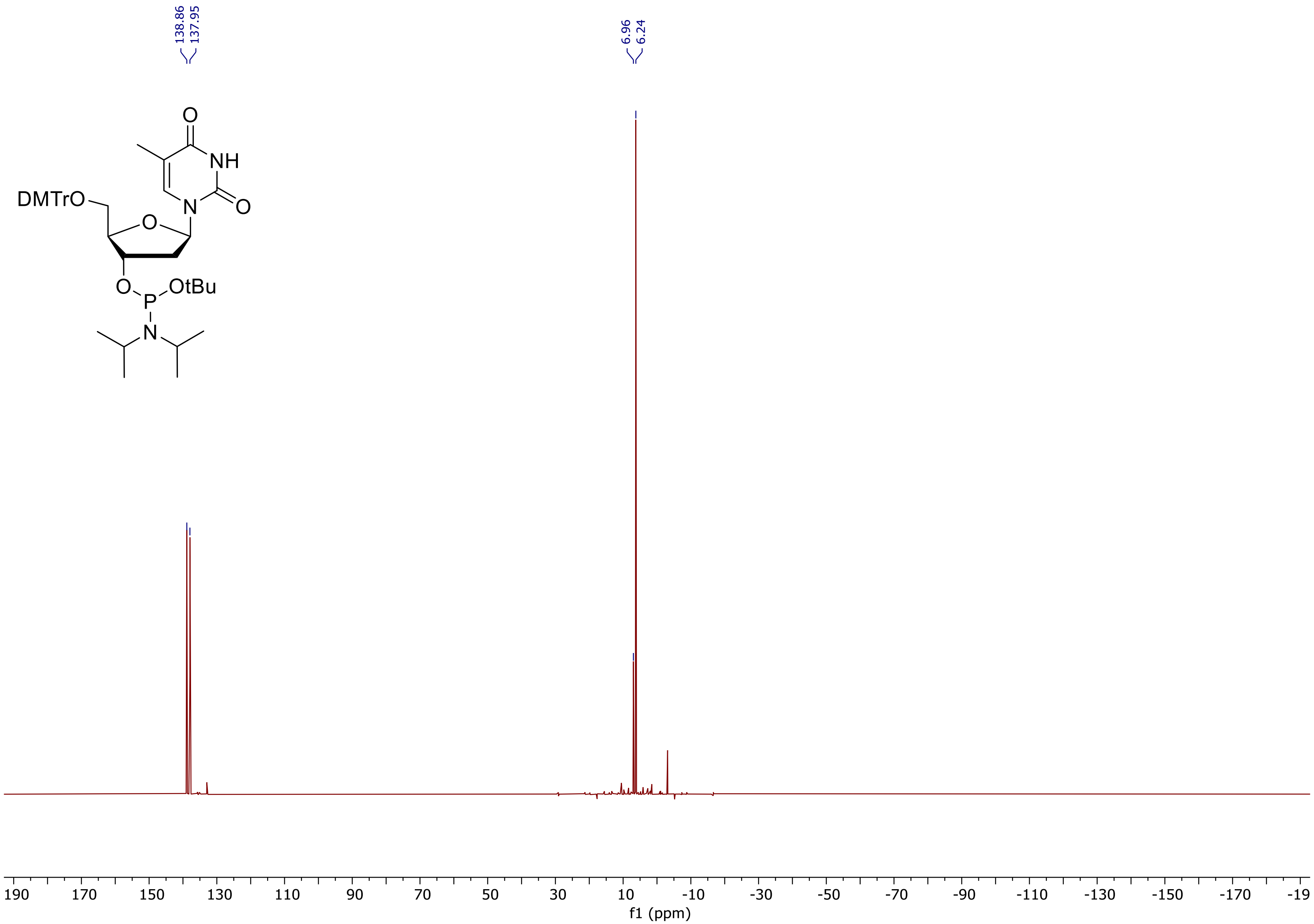

^{31}P-NMR (162 MHz, $CDCl_3$) of compound **57**.

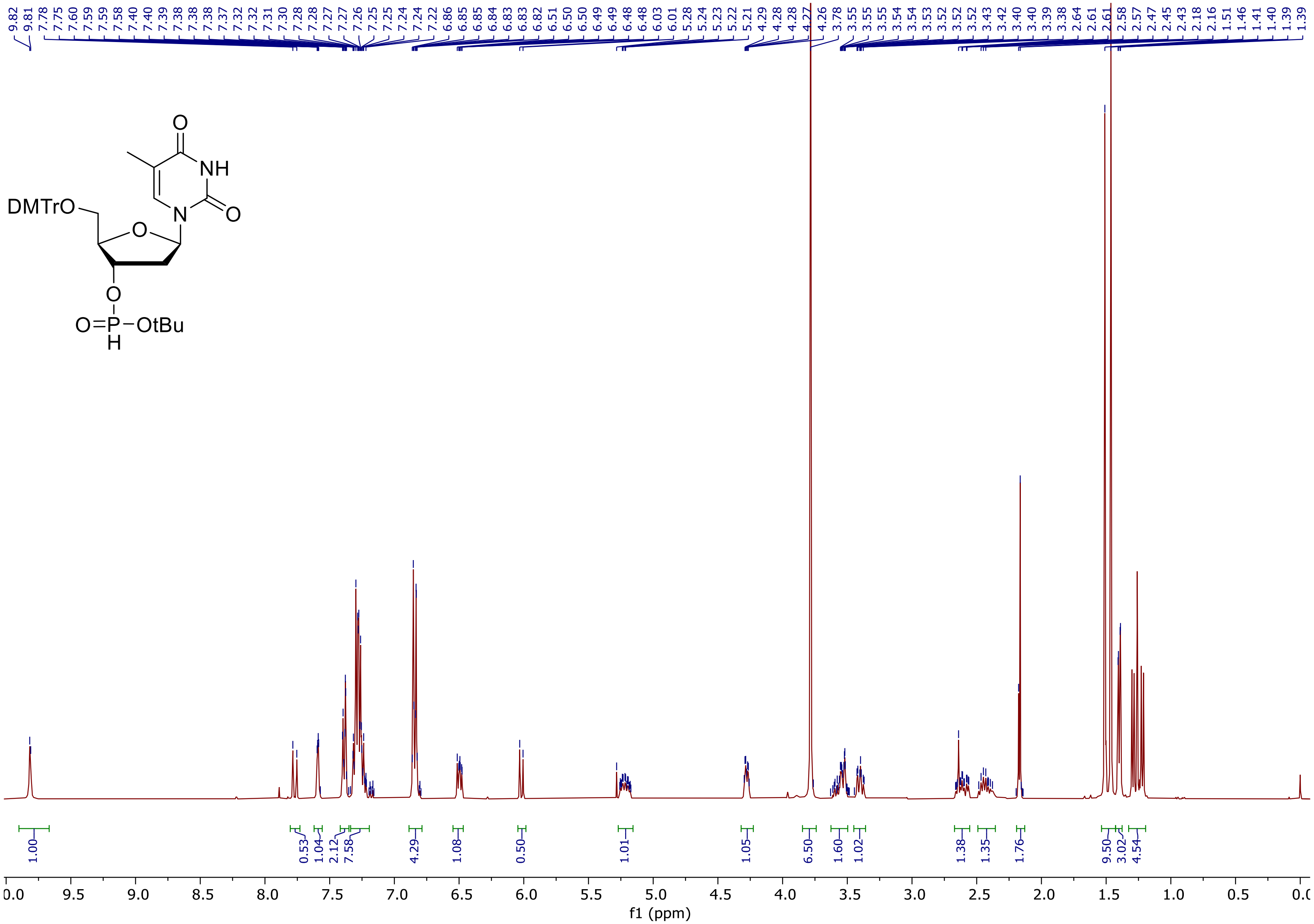

[1]**H-NMR** (400 MHz, $CDCl_3$) of compound **1**. Solvent peak at 7.26 ppm.

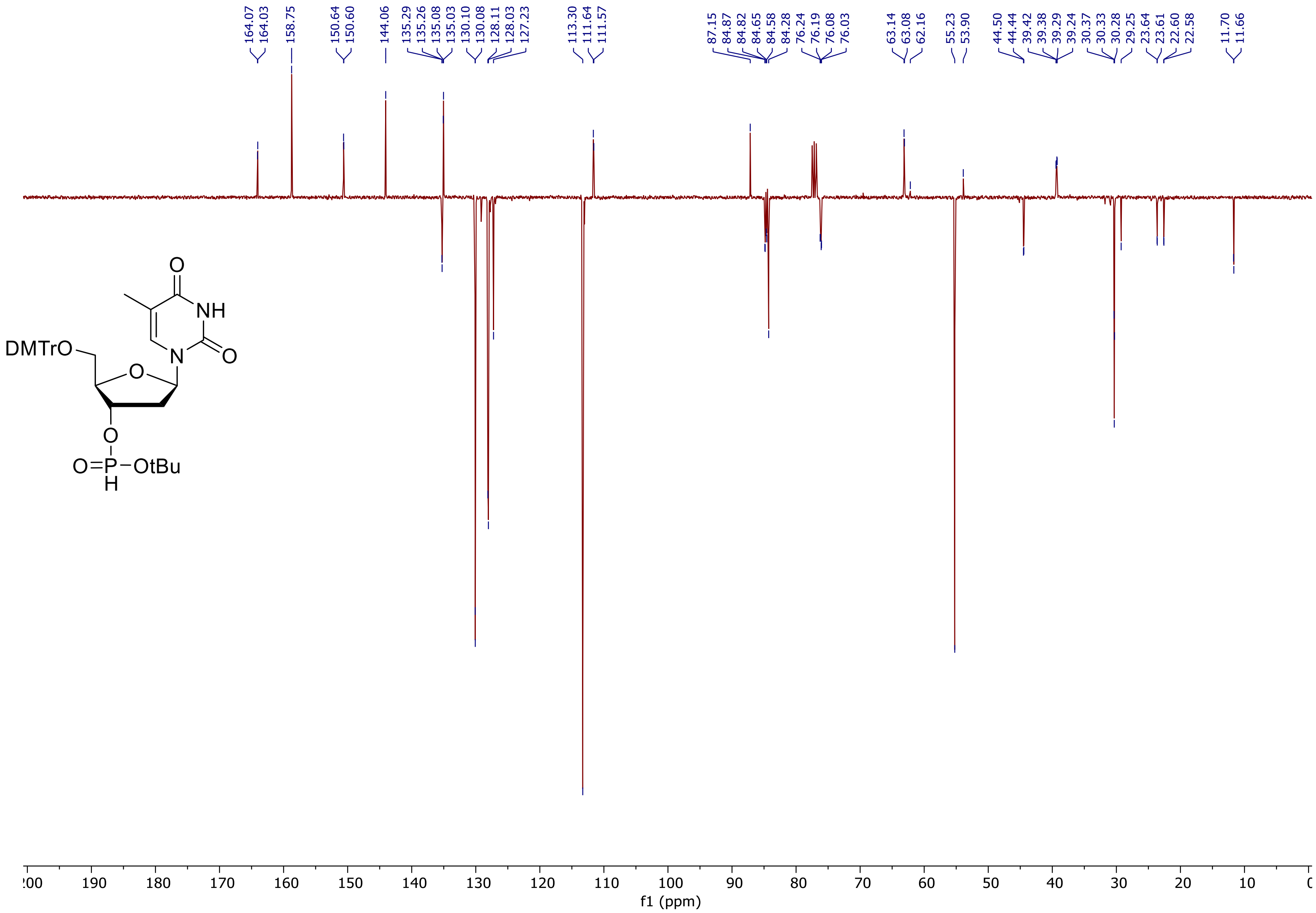

^{13}C-NMR (101 MHz, $CDCl_3$) of compound **1**. Solvent peak at 77.16 ppm.

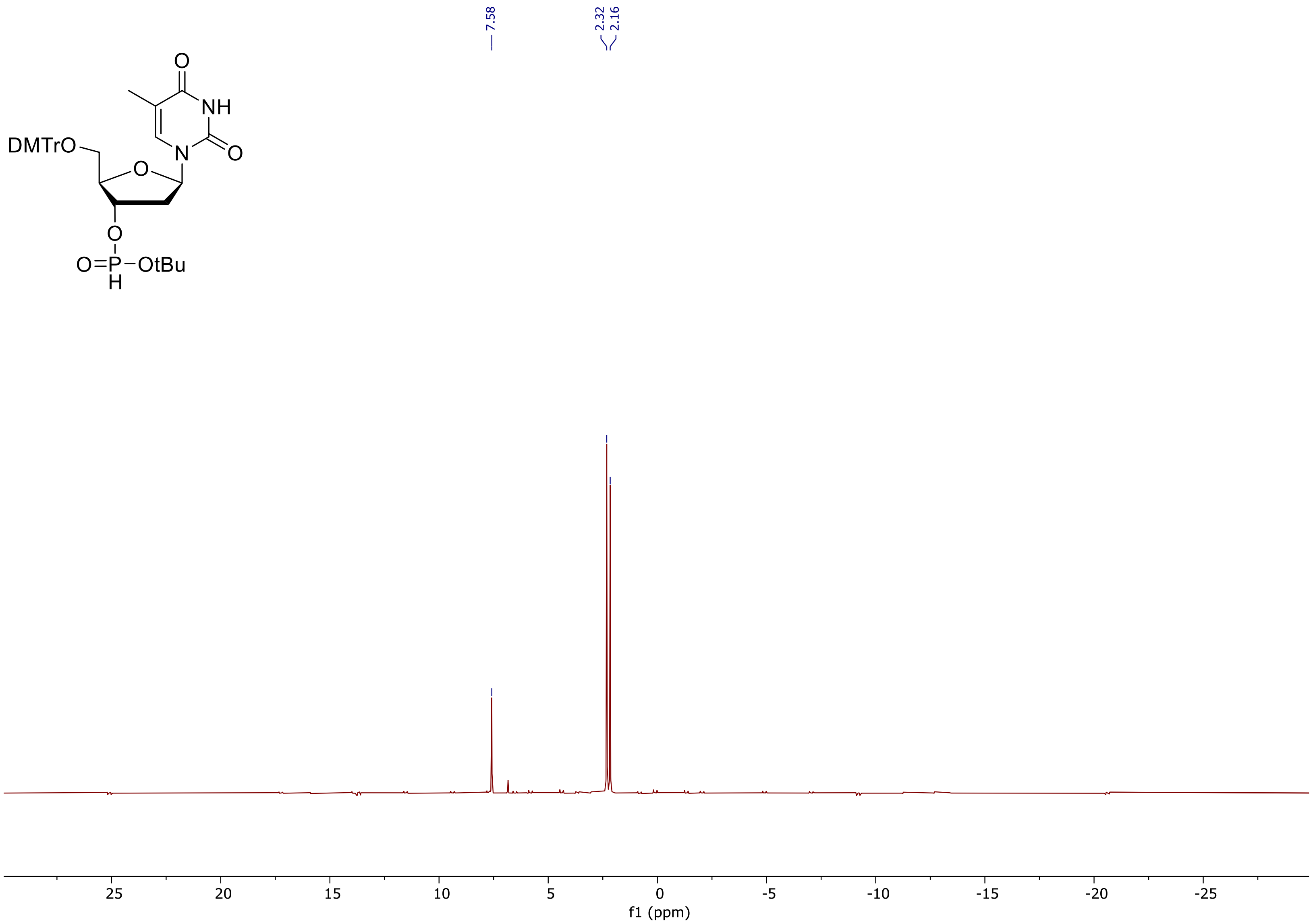

^{31}P-NMR (162 MHz, $CDCl_3$) of compound **1**.

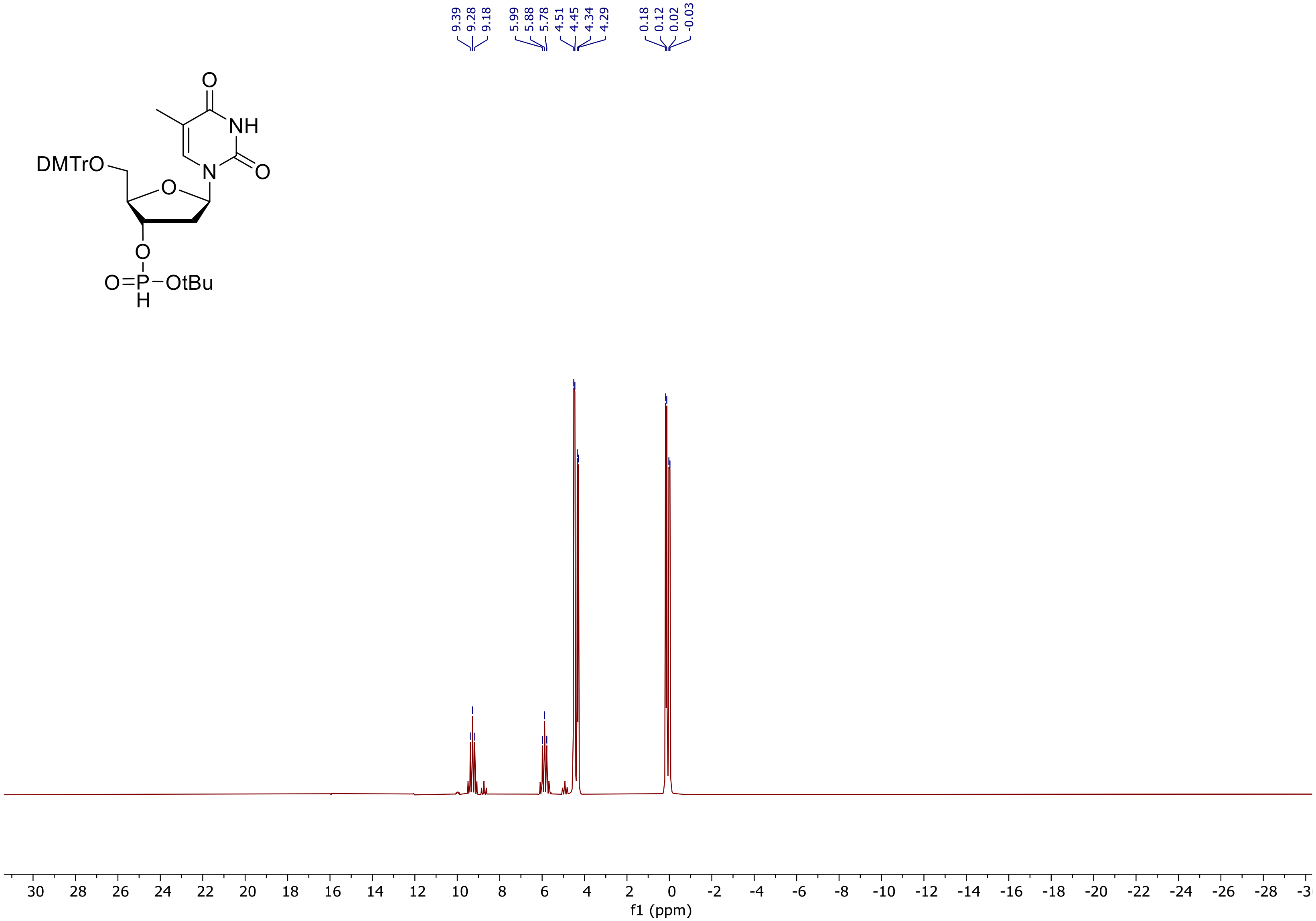

^{31}**P-NMR** (162 MHz, $CDCl_3$, proton coupled) of compound **1**.

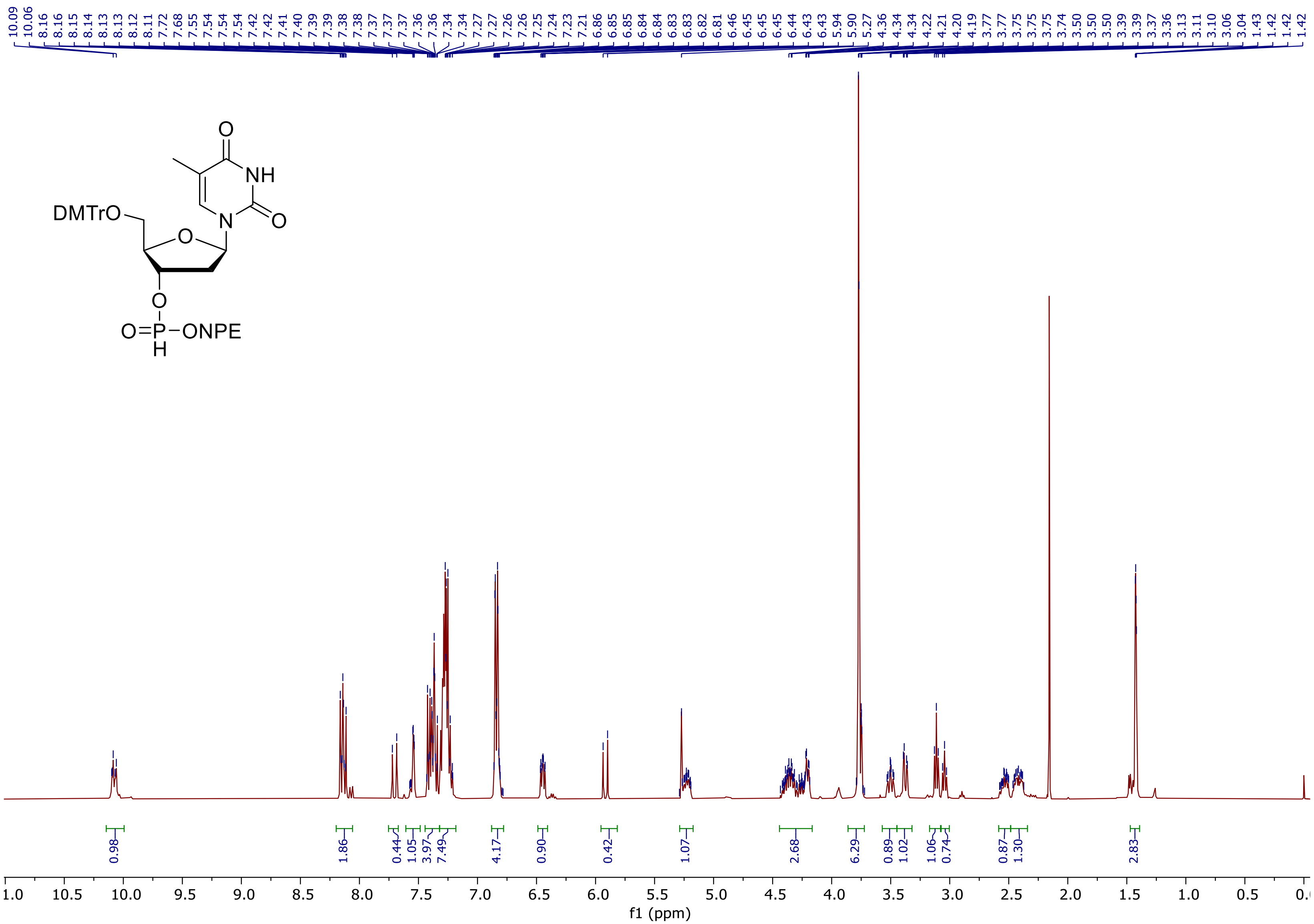

[1]**H-NMR** (400 MHz, $CDCl_3$) of compound **2**. Solvent peak at 7.26 ppm.

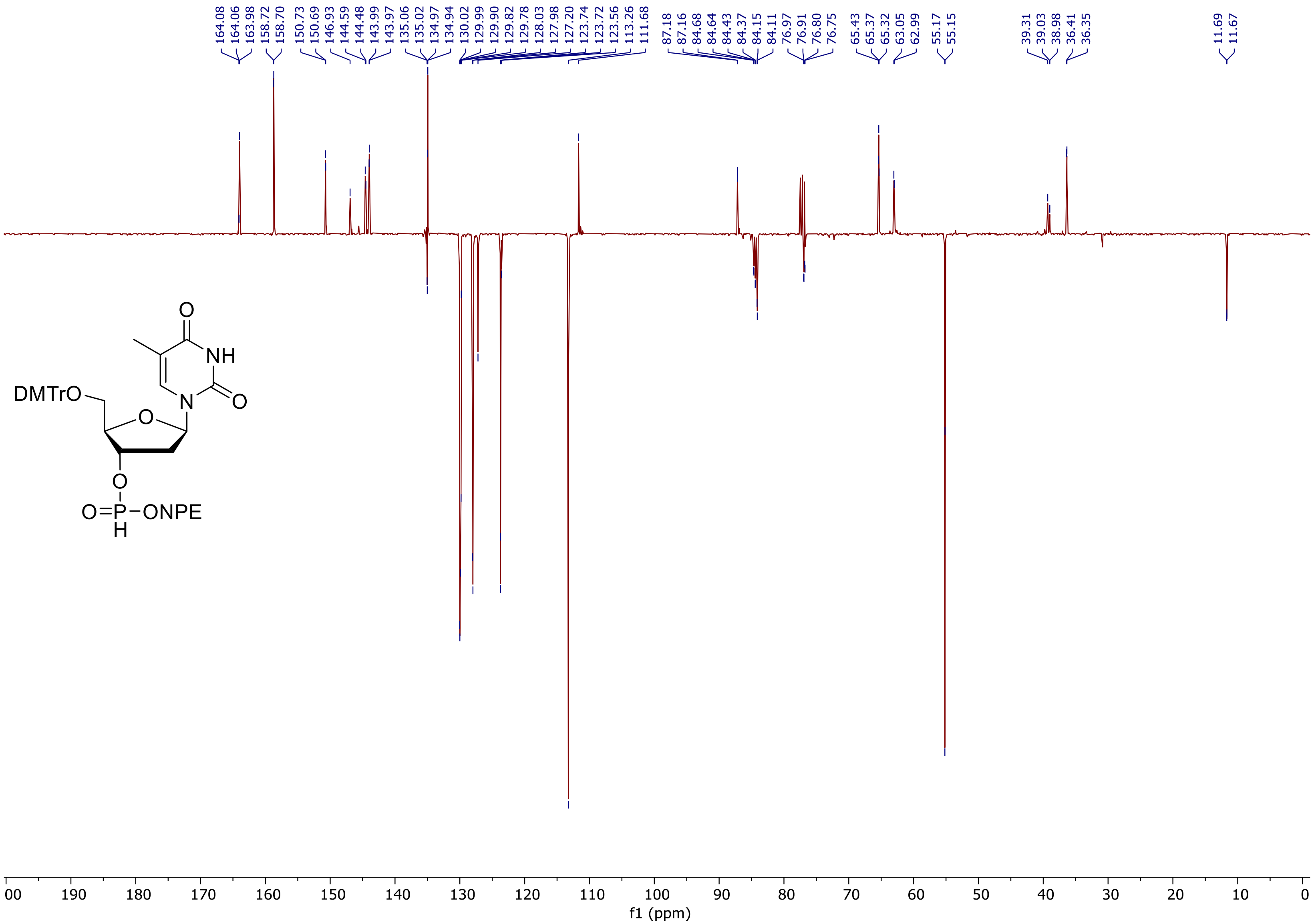

^{13}C-NMR (101 MHz, $CDCl_3$) of compound **2**. Solvent peak at 77.16 ppm.

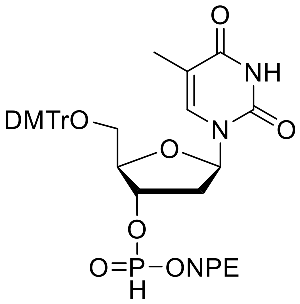

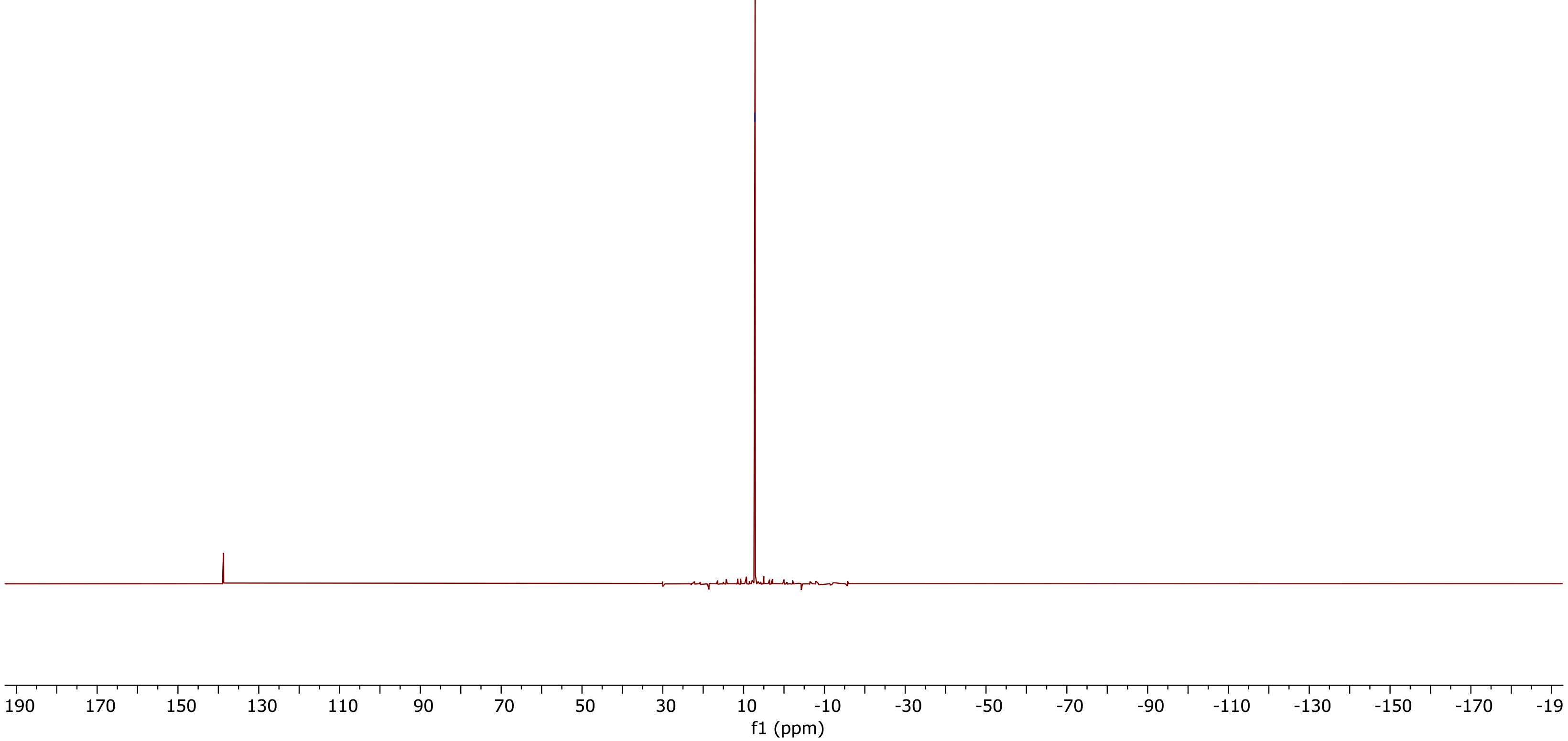

^{31}P-NMR (162 MHz, $CDCl_3$) of compound **2**.

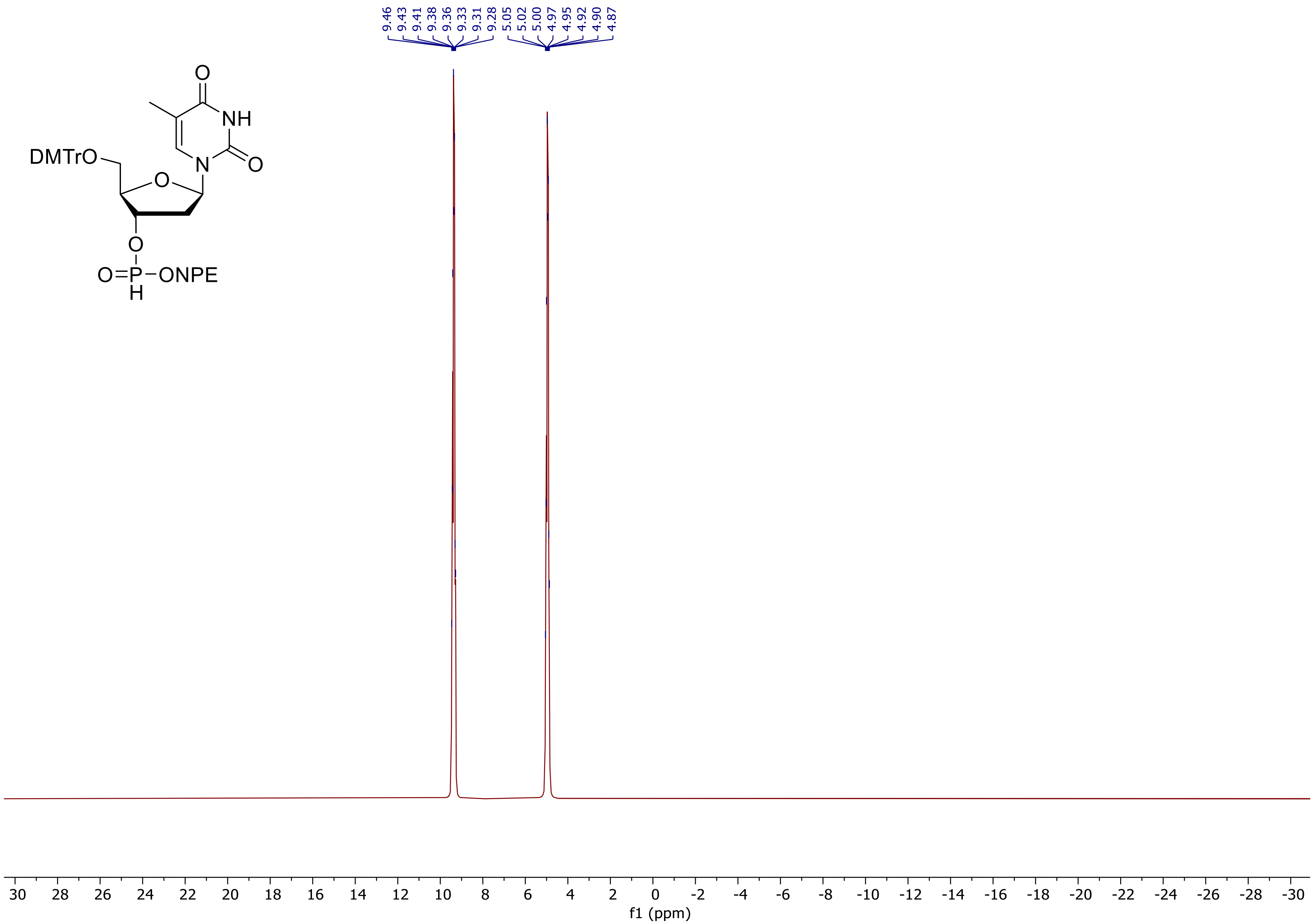

^{31}**P-NMR** (162 MHz, $CDCl_3$, proton coupled) of compound **2**.

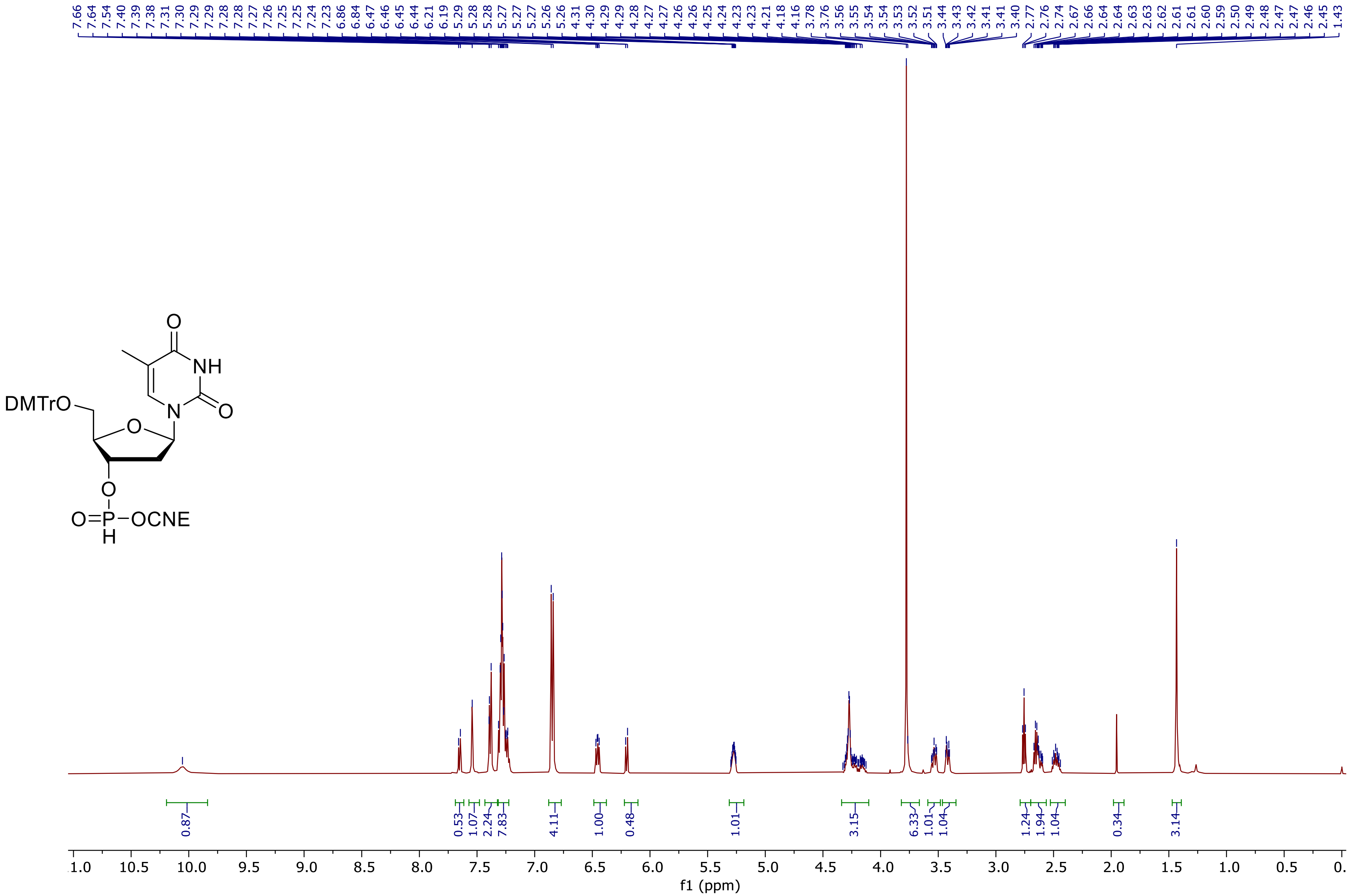

^{1}H-NMR (500 MHz, $CDCl_3$) of compound **3**. Solvent peak at 7.26 ppm.

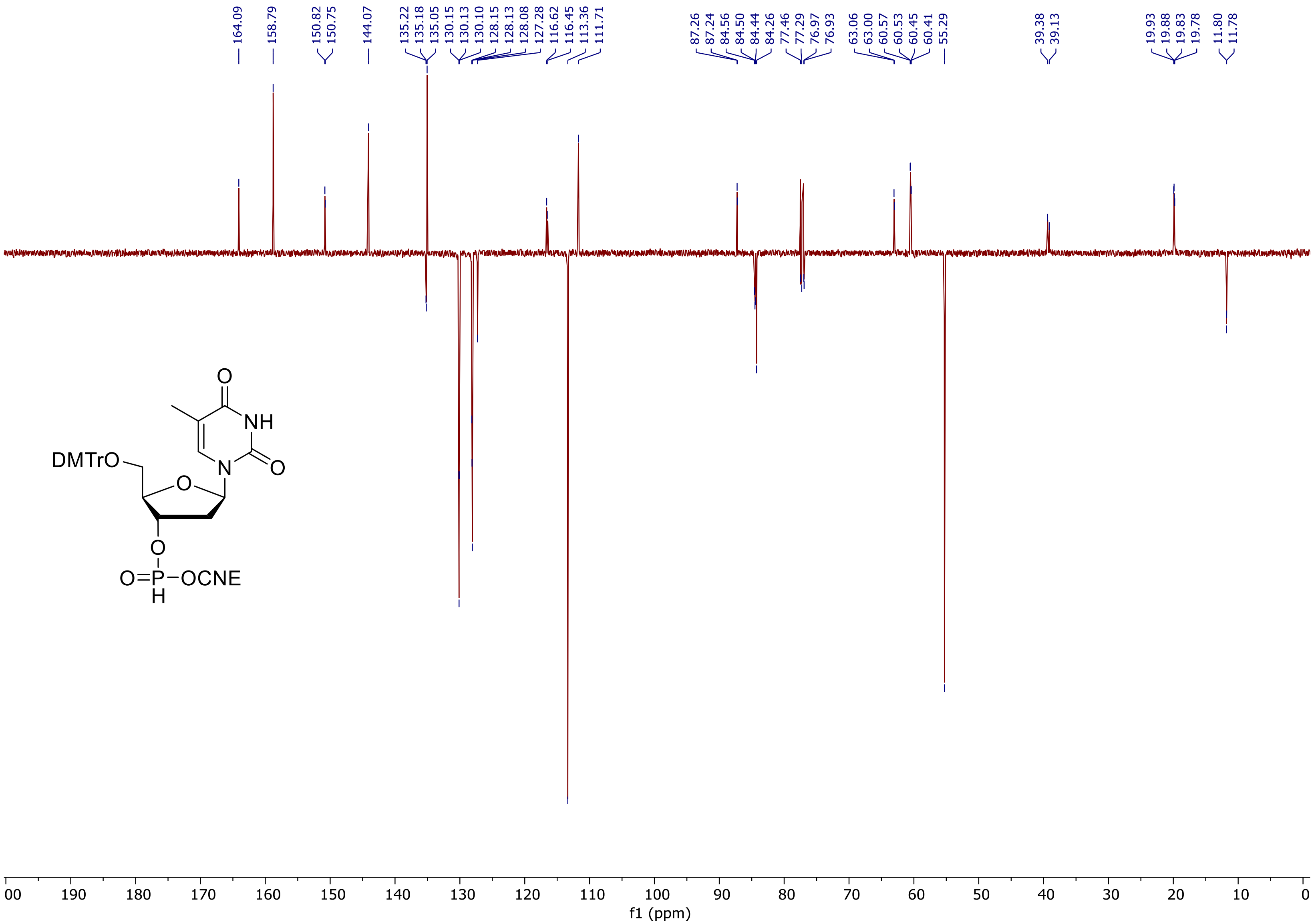

^{13}**C-NMR** (126 MHz, $CDCl_3$) of compound **3**. Solvent peak at 77.16 ppm.

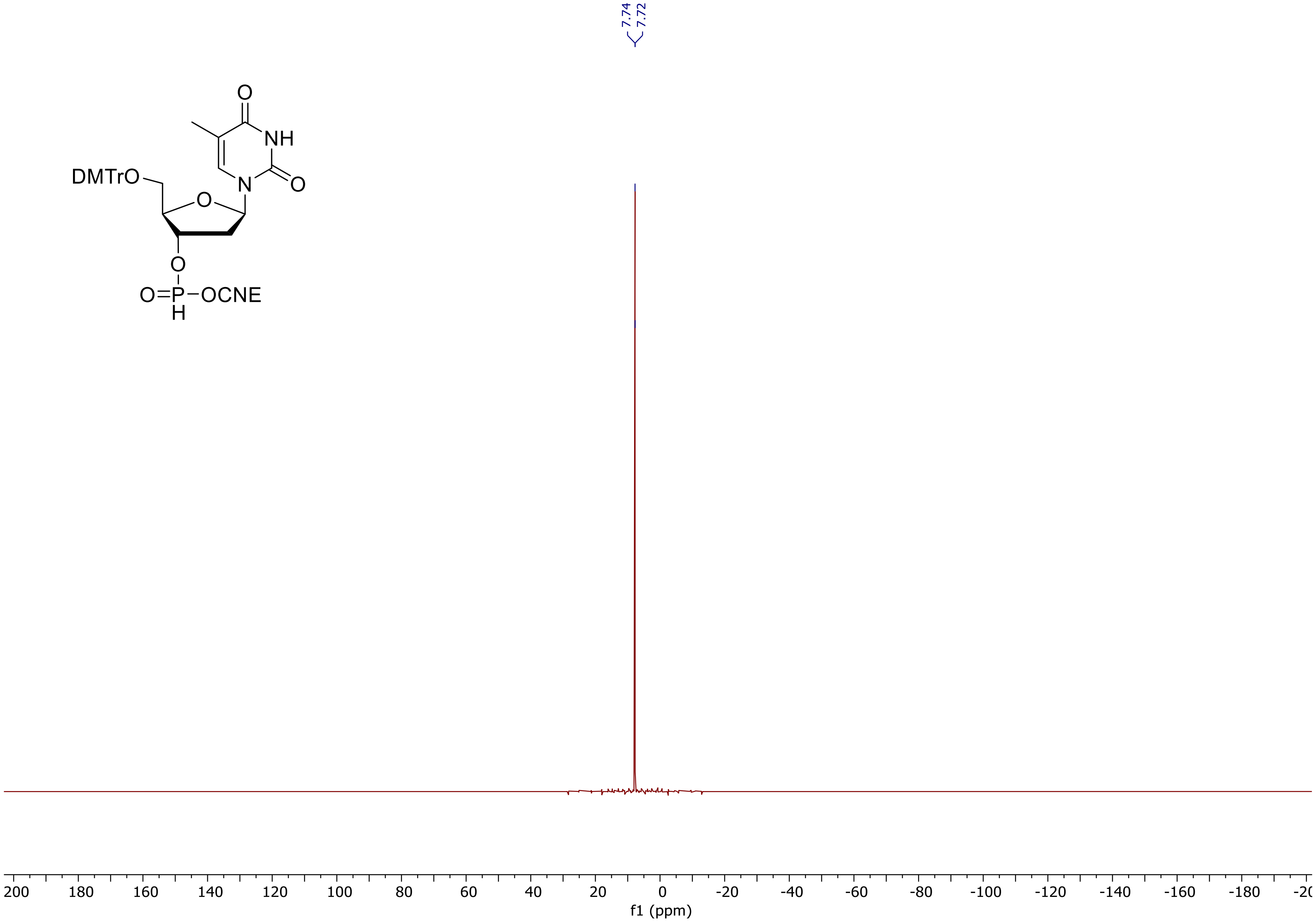

[31]**P-NMR** (202 MHz, $CDCl_3$) of compound **3**.

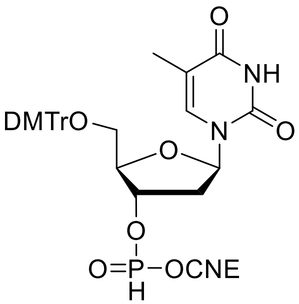

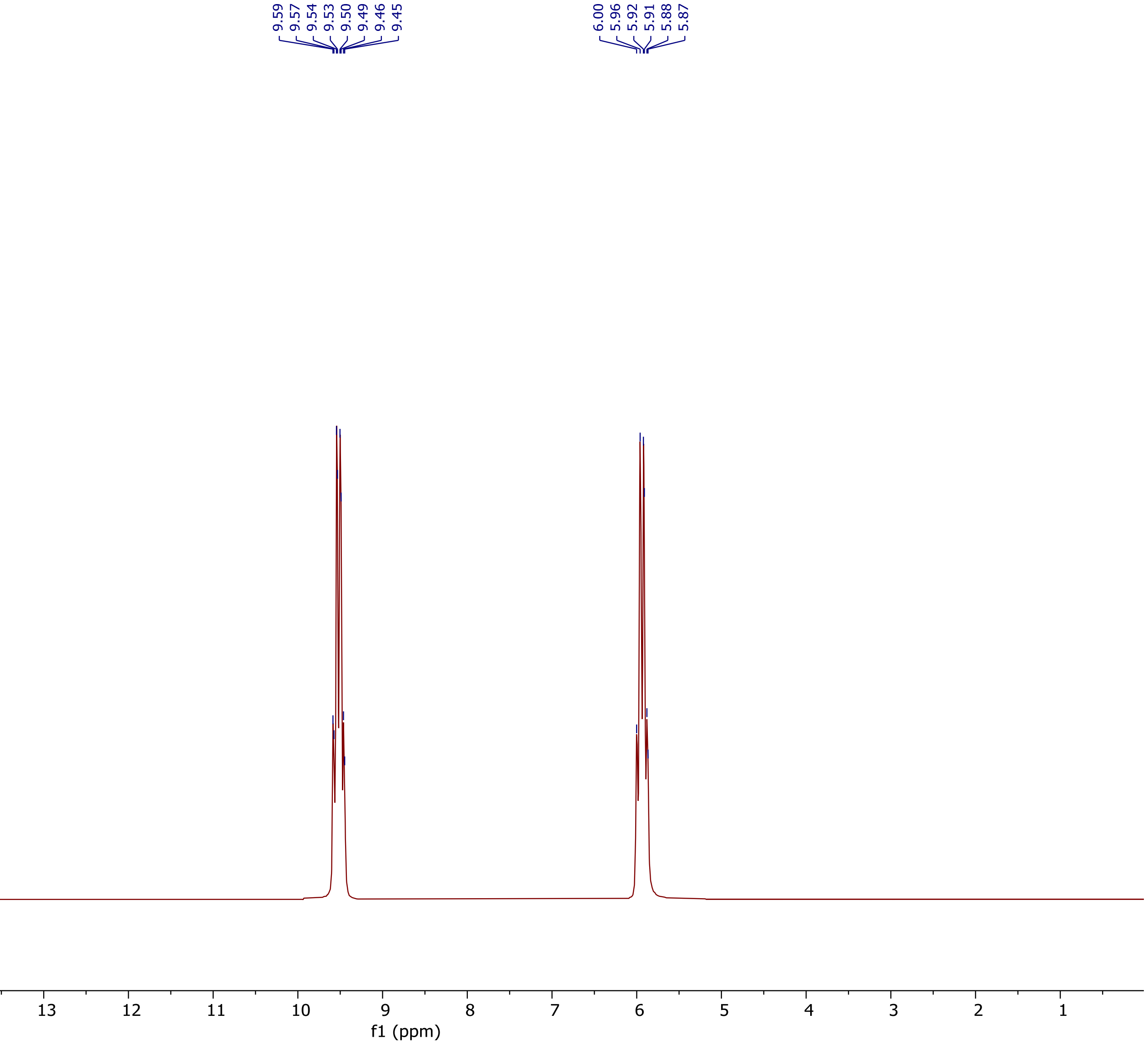

^{31}**P-NMR** (202 MHz, $CDCl_3$, proton coupled) of compound **3**.

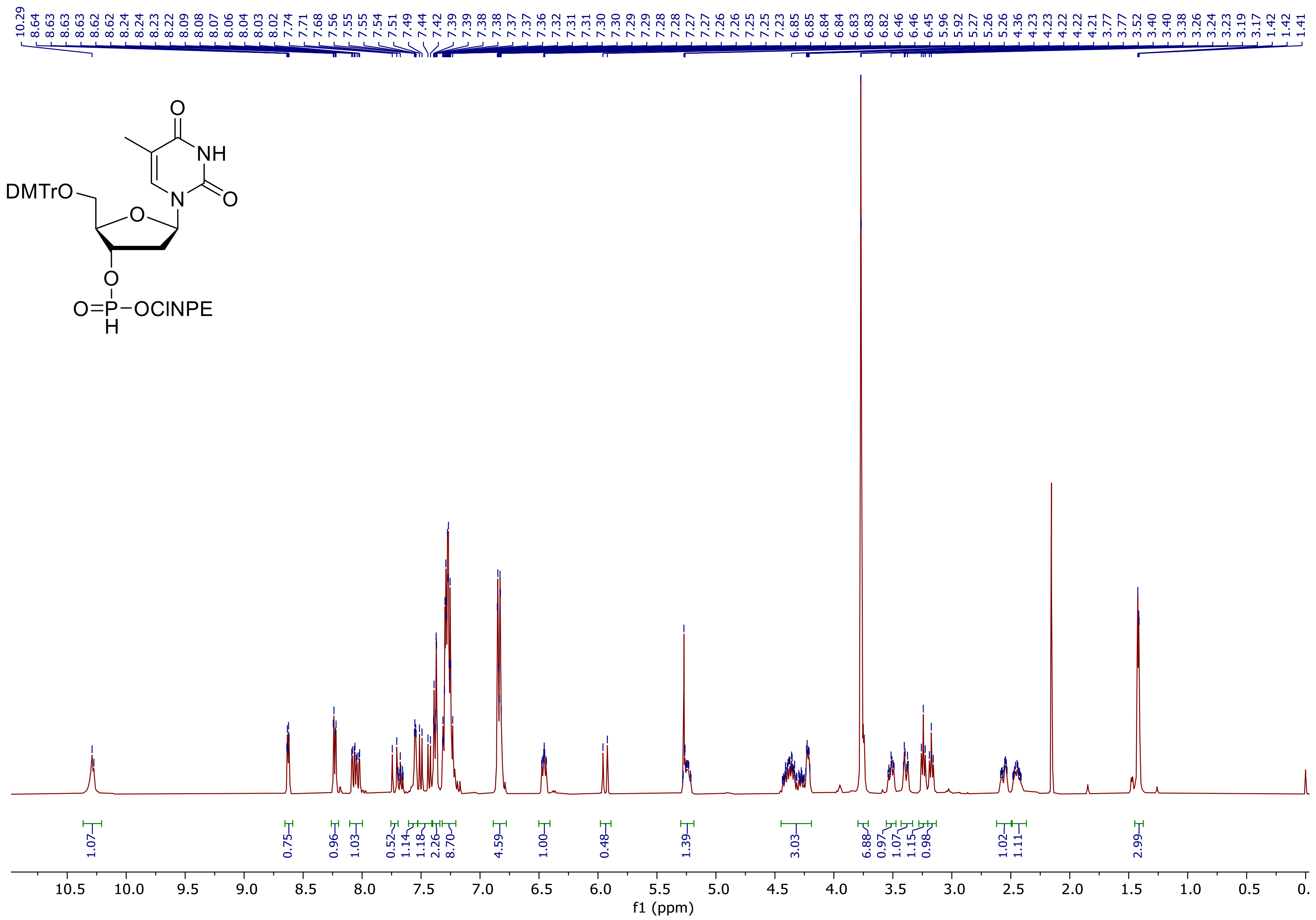

^{1}H-NMR (400 MHz, $CDCl_3$) of compound **4**. Solvent peak at 7.26 ppm.

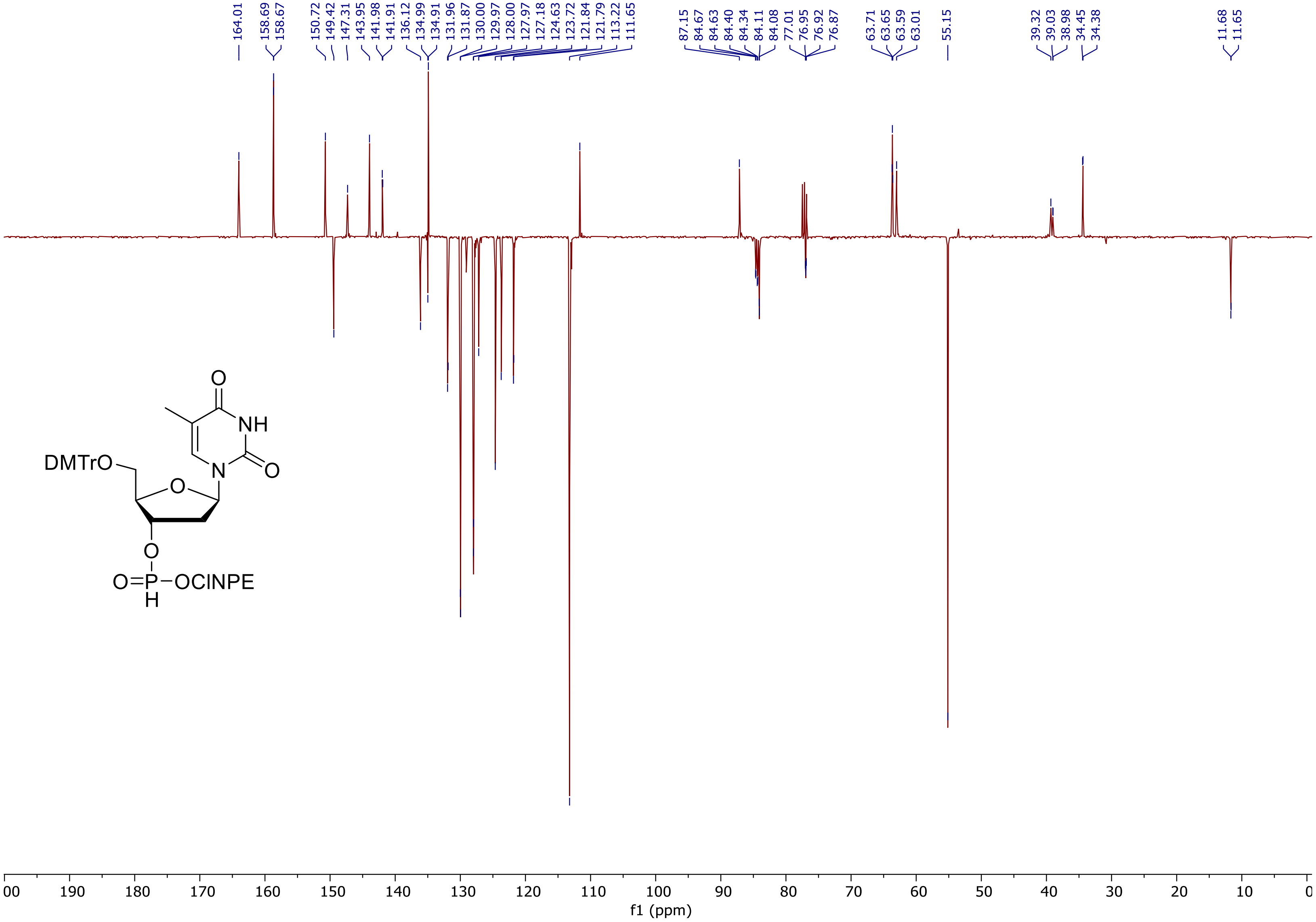

^{13}C-NMR (101 MHz, $CDCl_3$) of compound **4**. Solvent peak at 77.16 ppm.

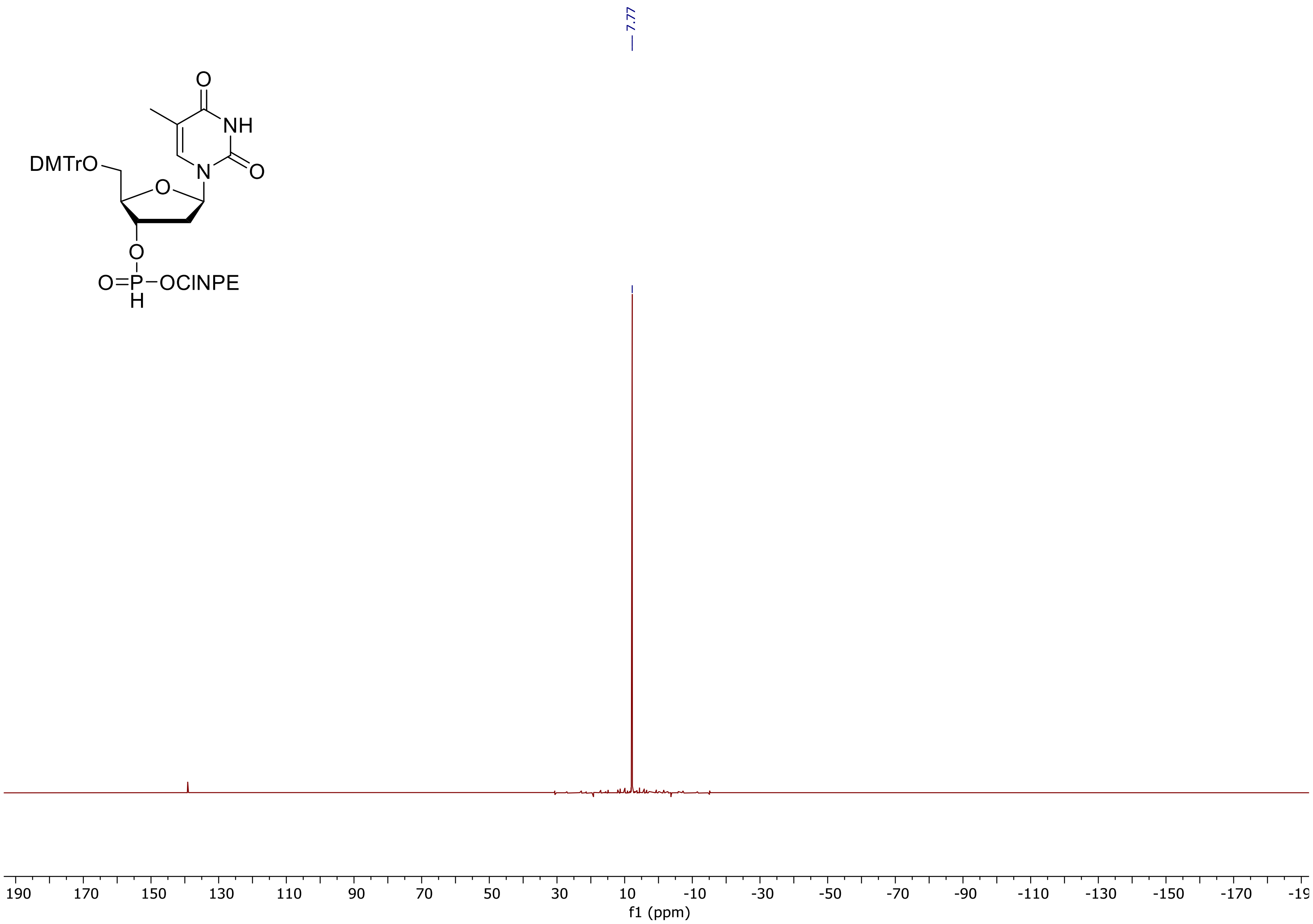

^{31}P-NMR (162 MHz, $CDCl_3$) of compound **4.**

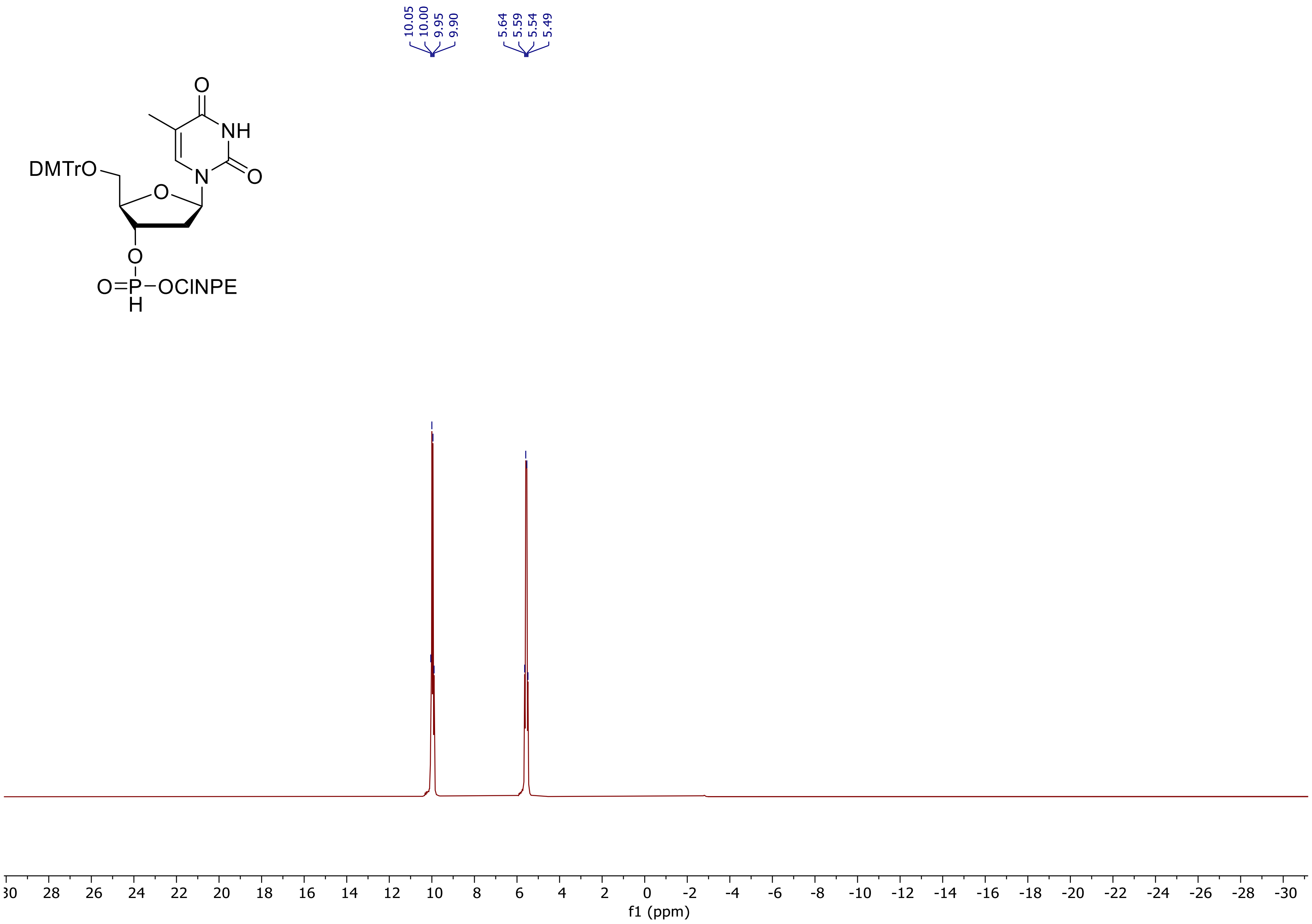

^{31}P-NMR (162 MHz, $CDCl_3$, proton coupled) of compound 4.

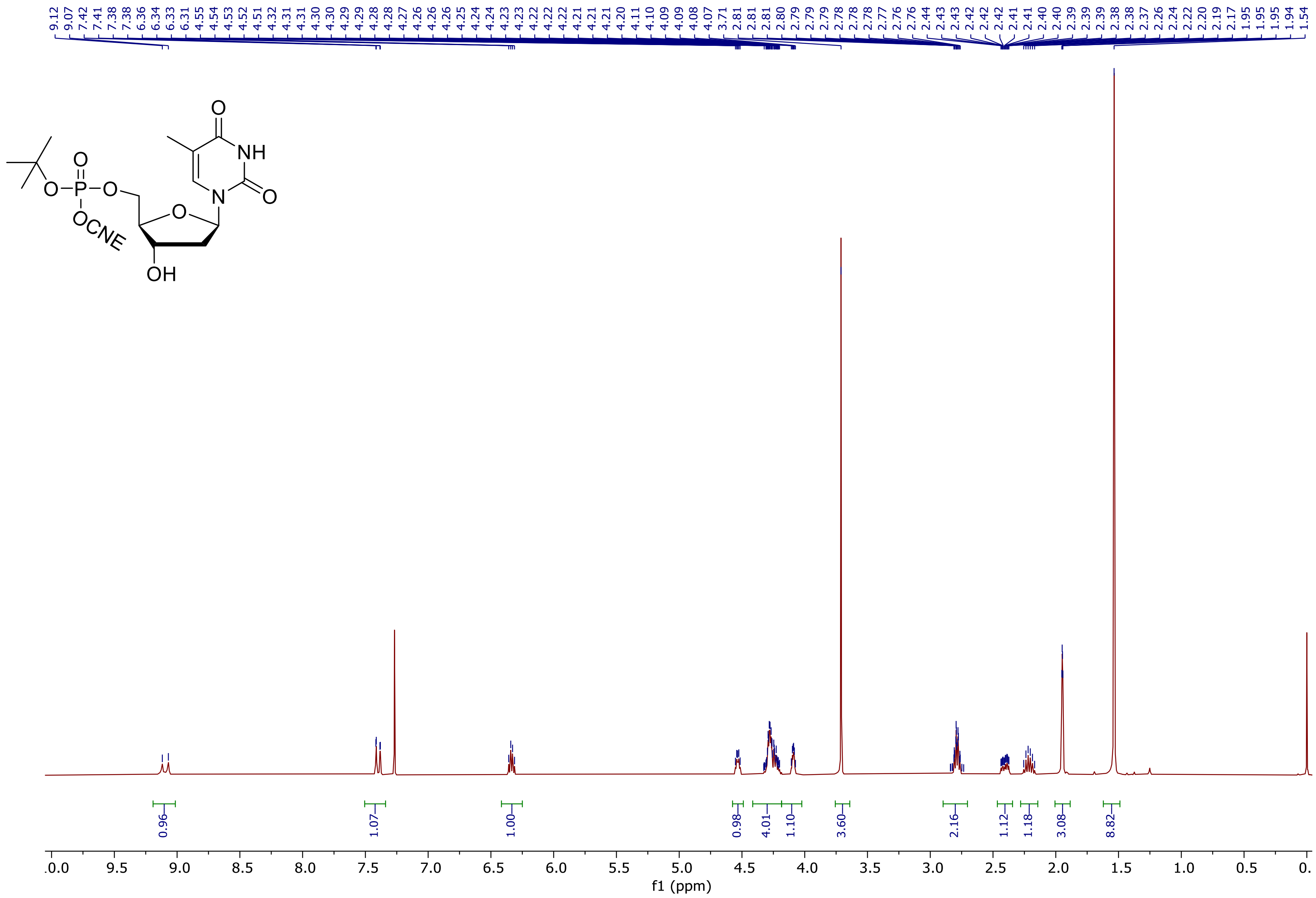

^{1}H-NMR (400 MHz, $CDCl_3$) of compound **60**. Solvent peak at 7.26 ppm.

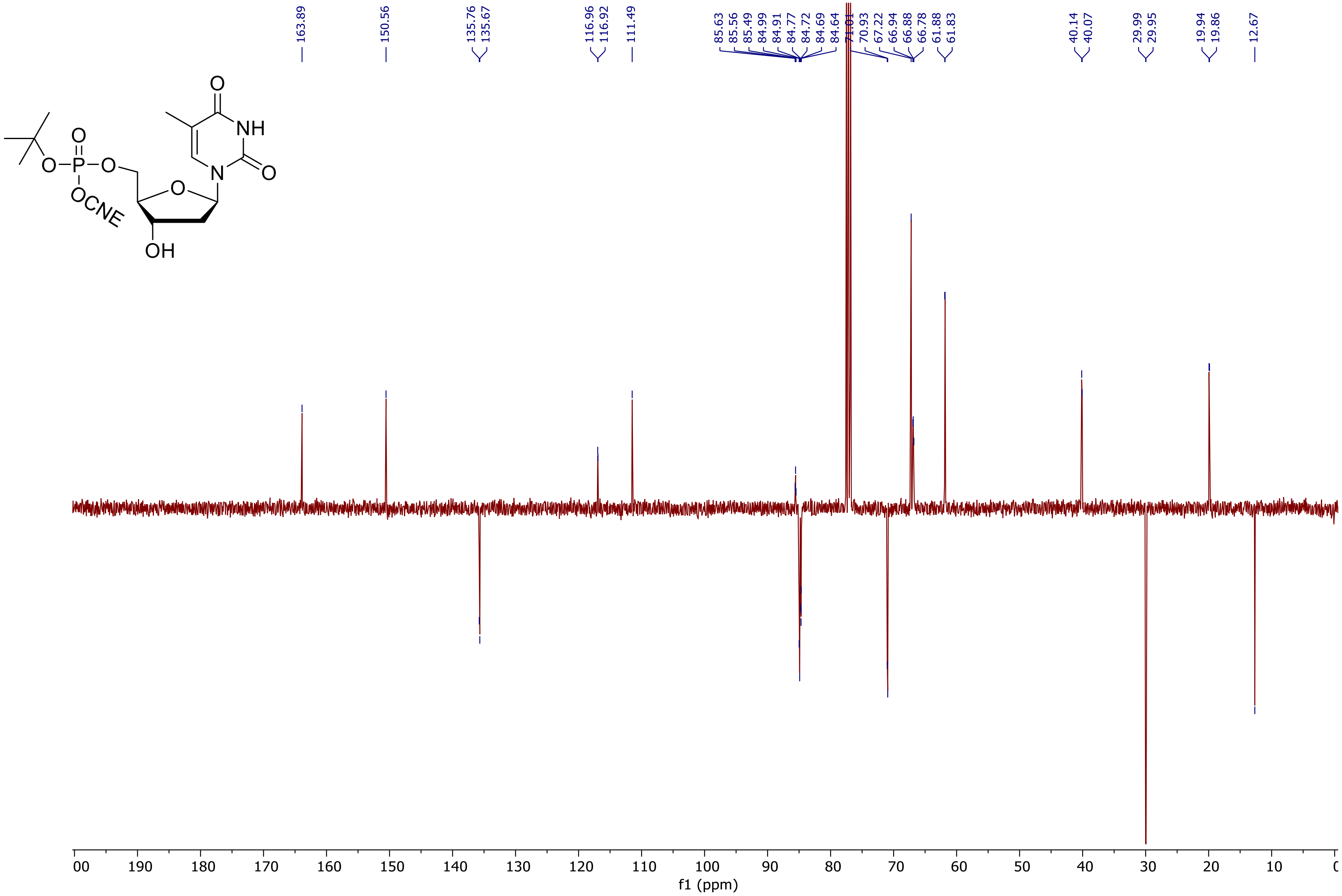

^{13}C-NMR (101 MHz, $CDCl_3$) of compound **60**. Solvent peak at 77.16 ppm.

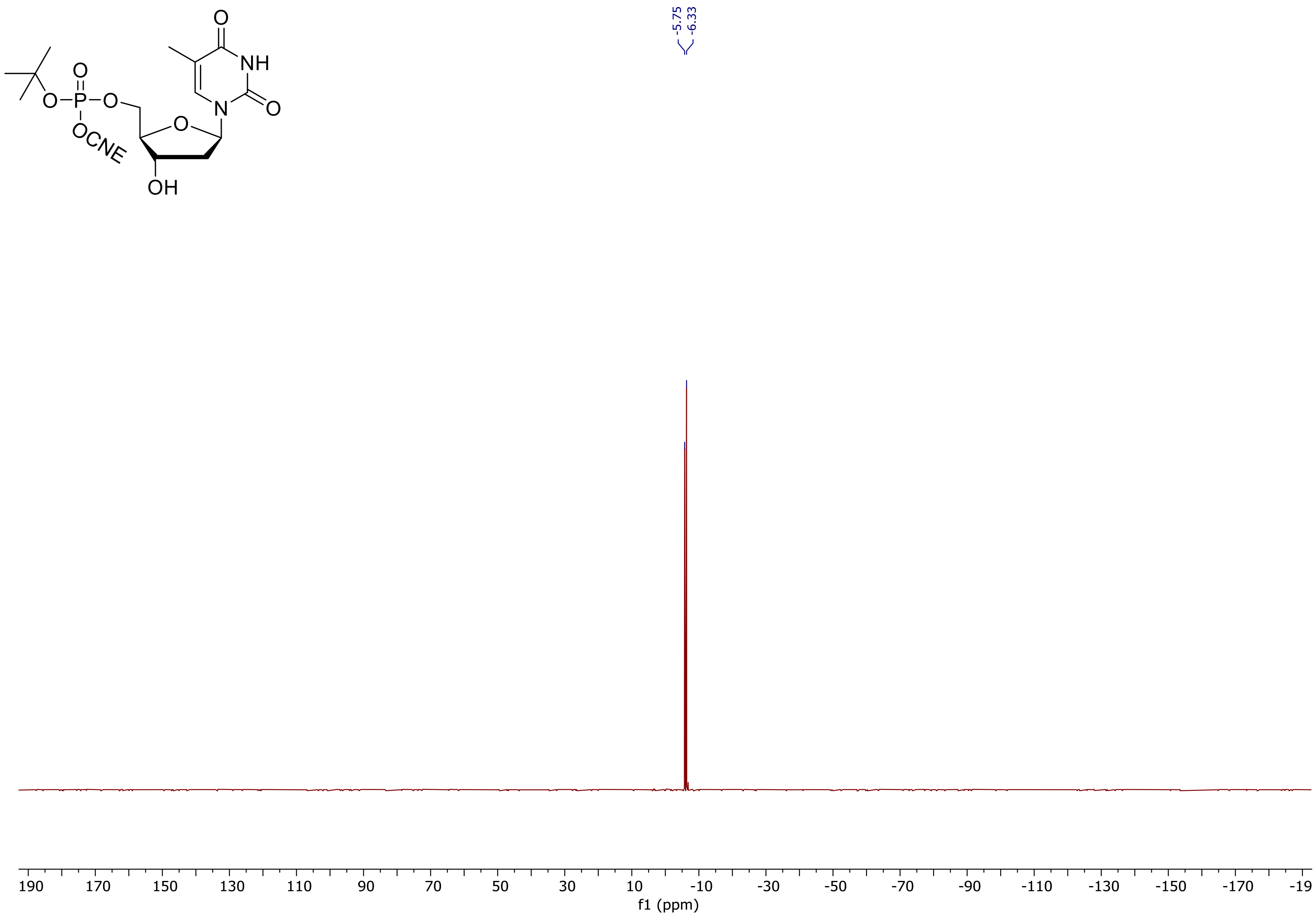

^{31}**P-NMR** (162 MHz, $CDCl_3$) of compound **60**.

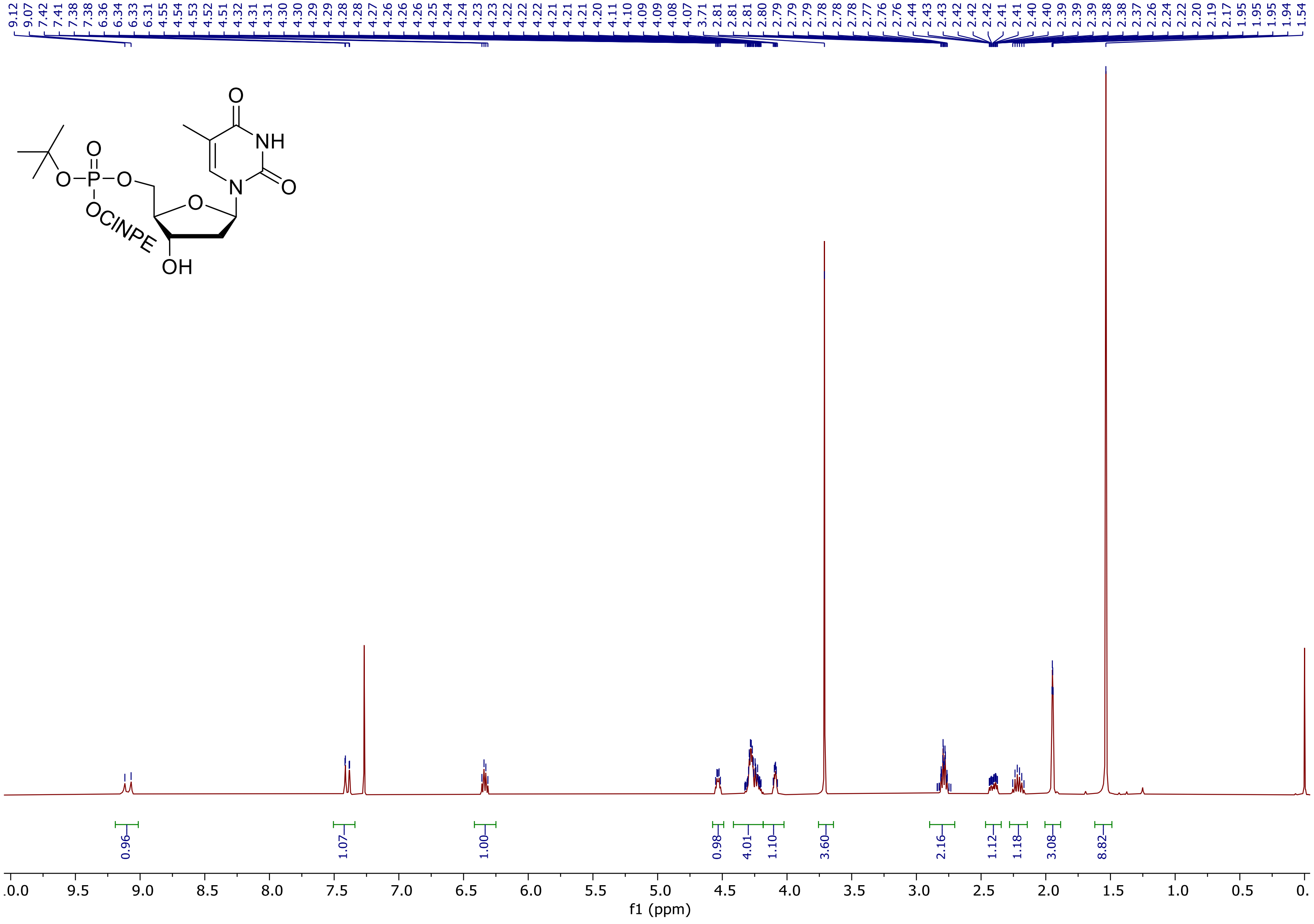

^{1}H-NMR (400 MHz, $CDCl_3$) of compound **61**. Solvent peak at 7.26 ppm.

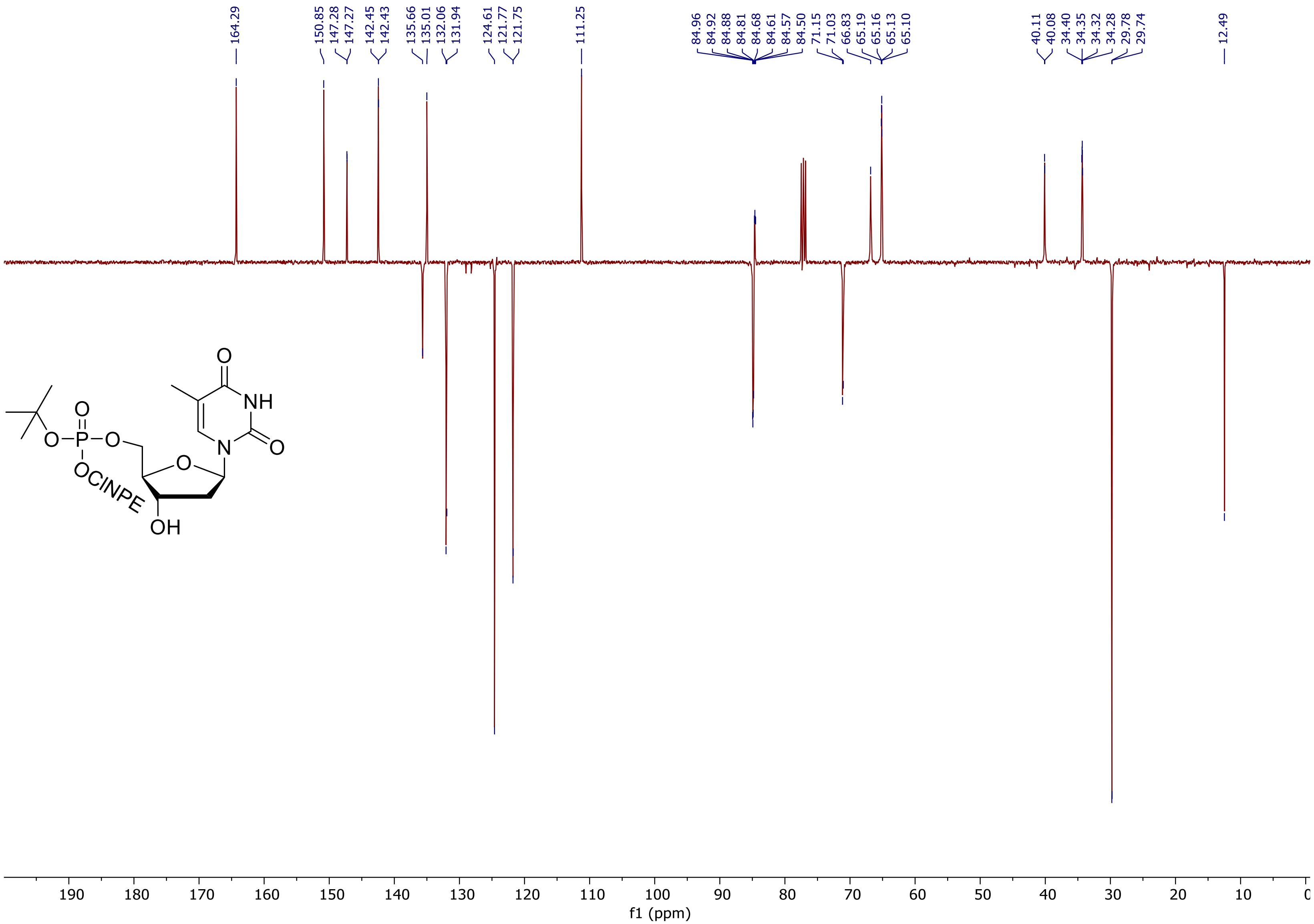

^{13}C-NMR (101 MHz, $CDCl_3$) of compound **61**. Solvent peak at 77.16 ppm.

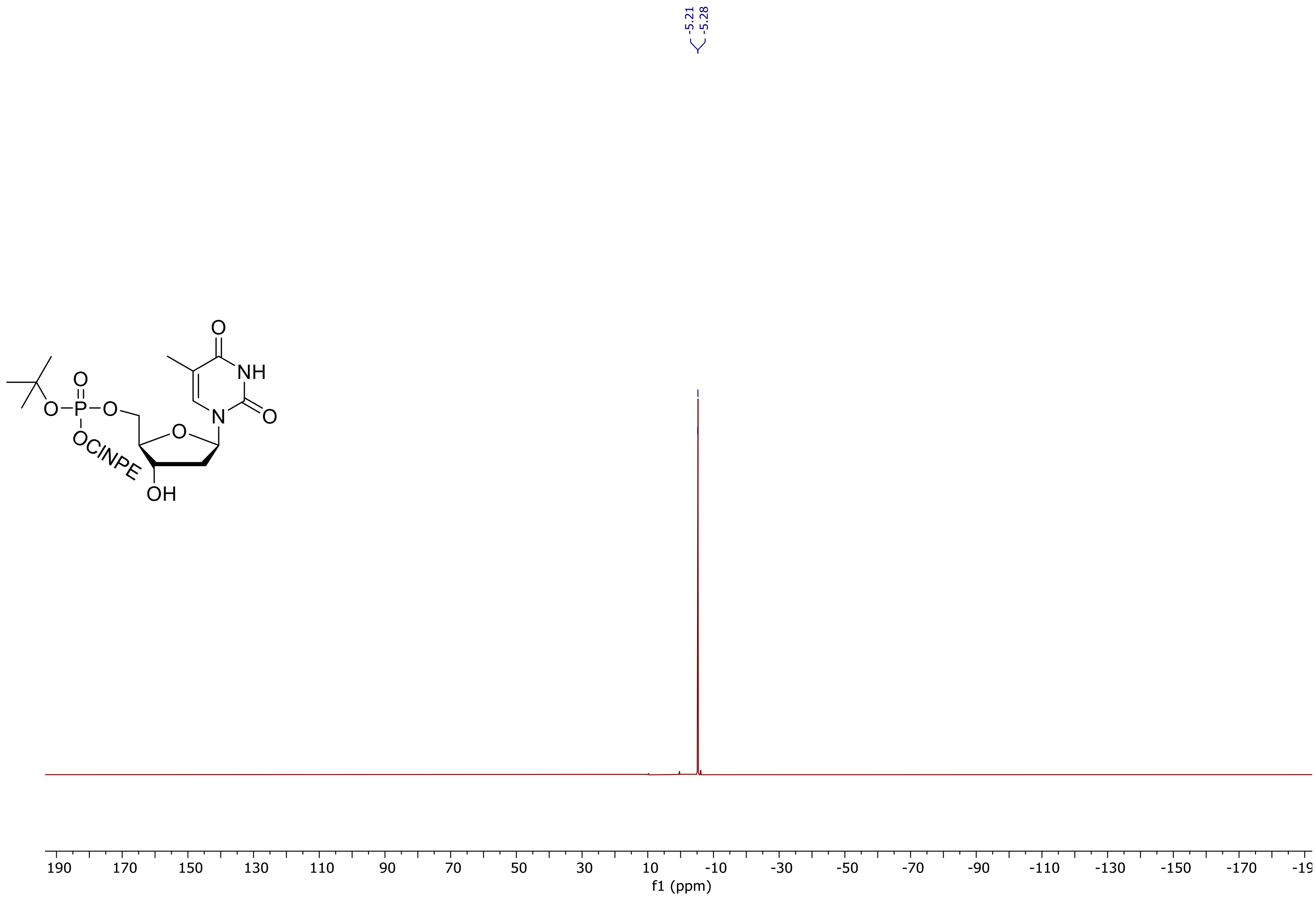

^{31}**P-NMR** (162 MHz, $CDCl_3$) of compound **61**.

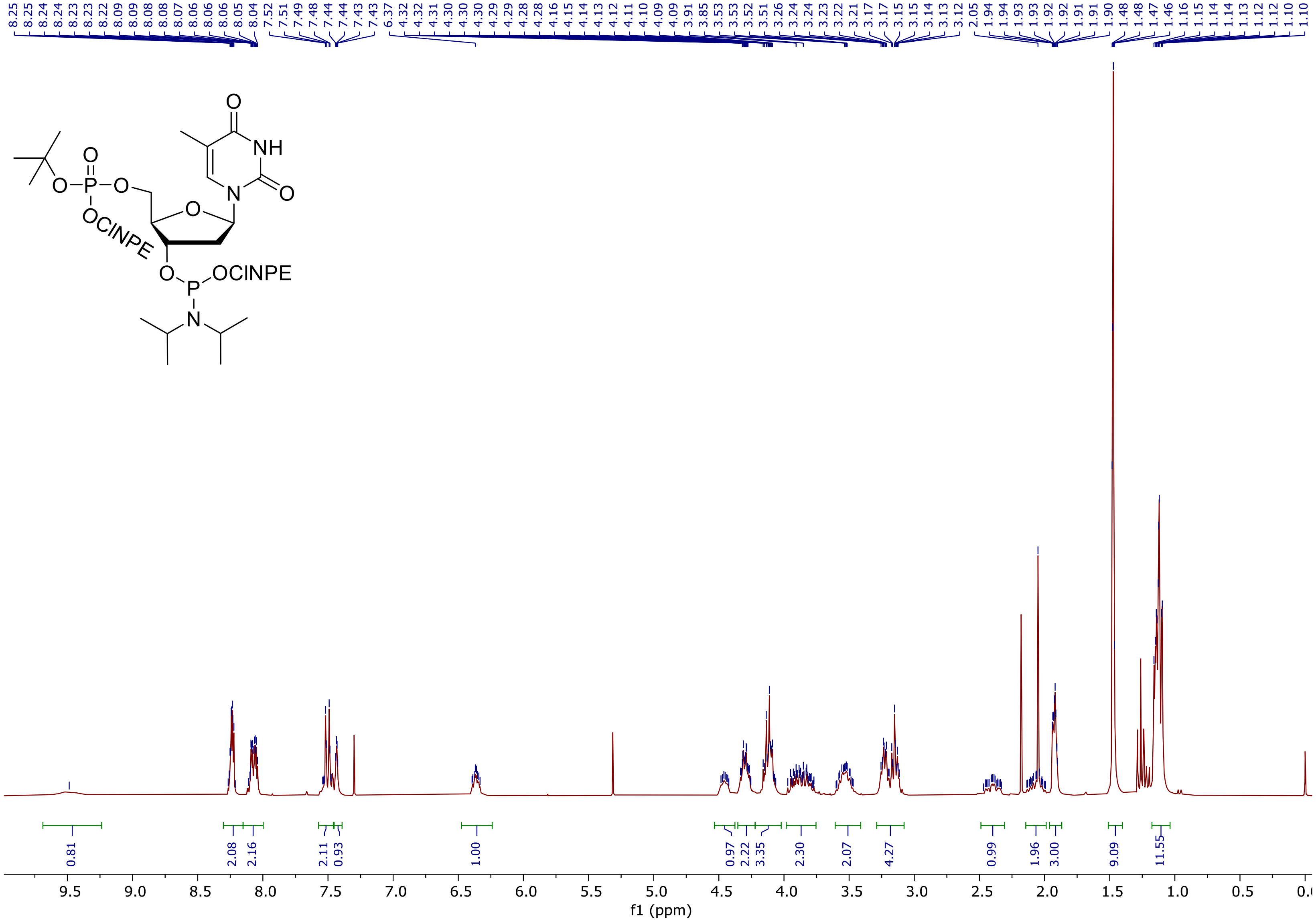

^{1}**H-NMR** (300 MHz, $CDCl_3$) of compound **62**. Solvent peak at 7.26 ppm.

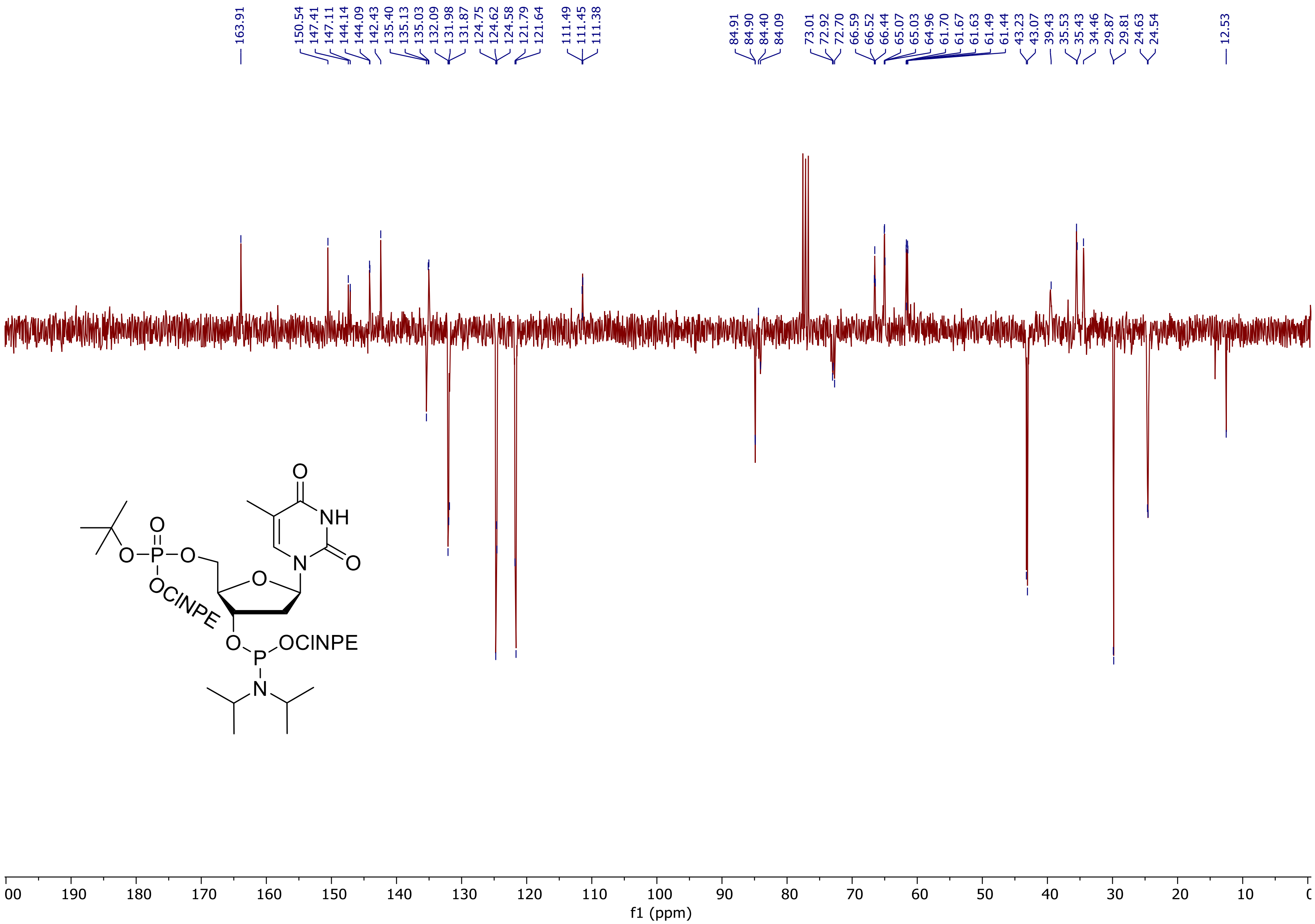

^{13}**C-NMR** (75 MHz, $CDCl_3$) of compound **62**. Solvent peak at 77.16 ppm.

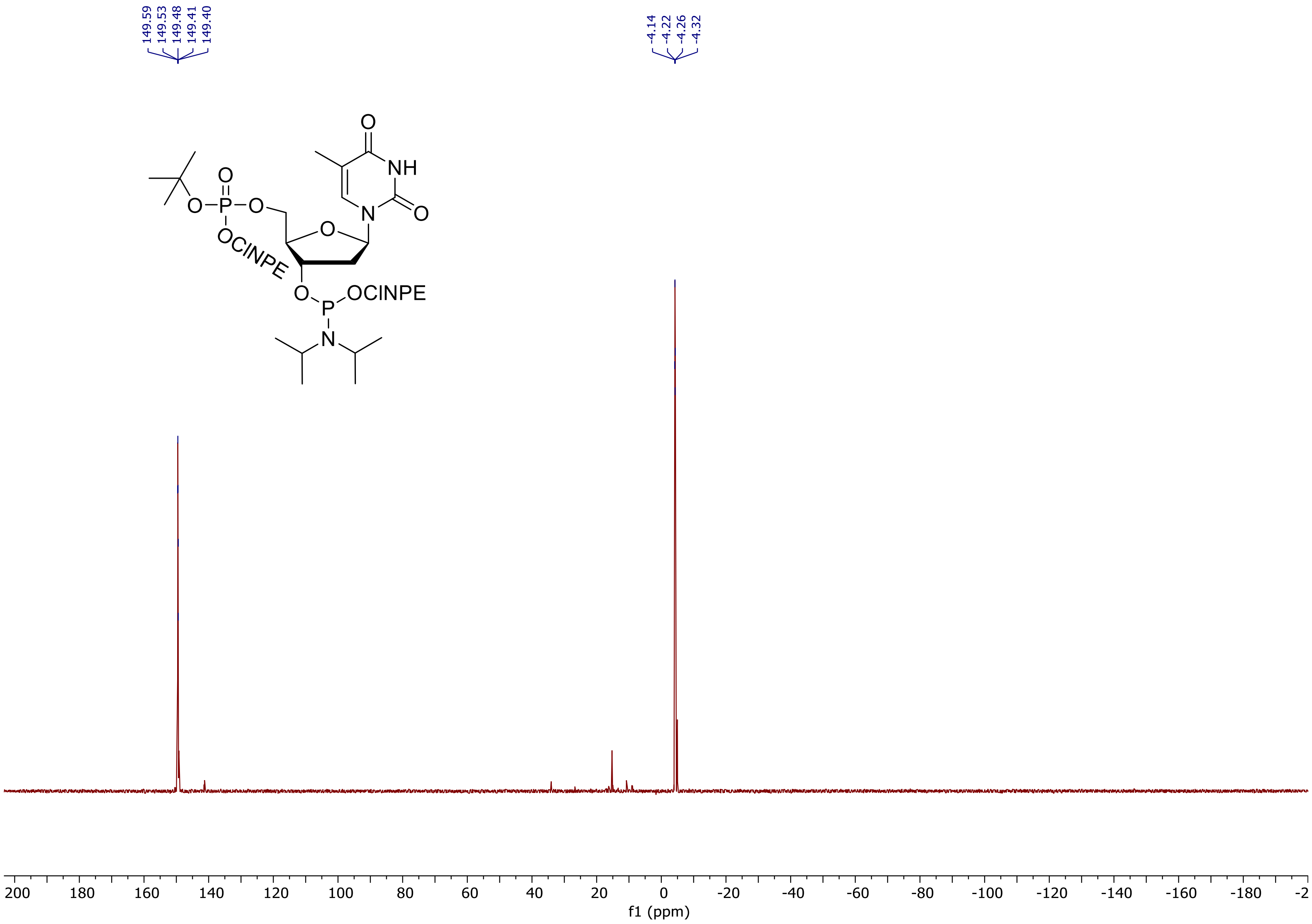

^{31}**P-NMR** (121 MHz, $CDCl_3$) of compound **62**.

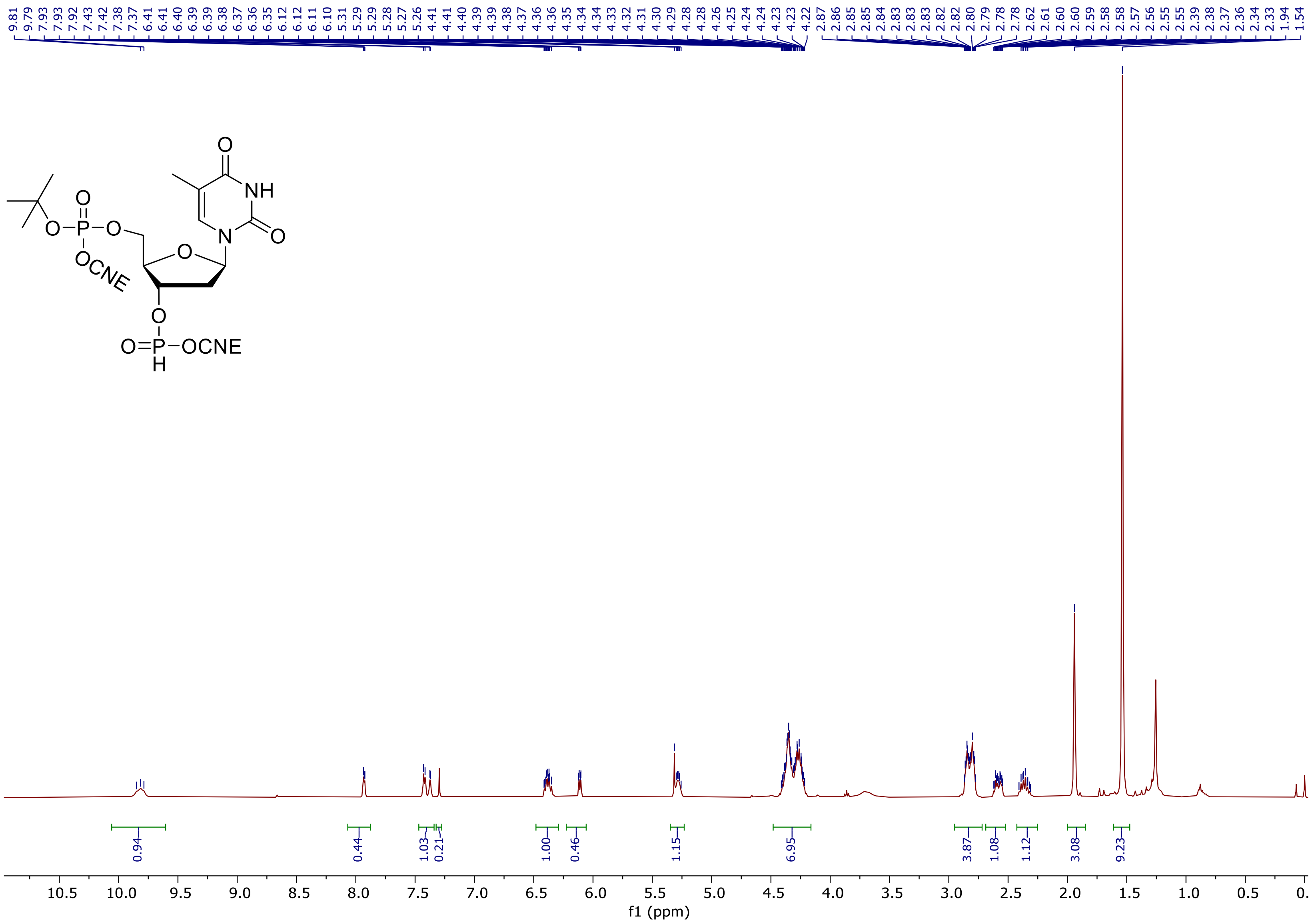

[1]**H-NMR** (400 MHz, $CDCl_3$) of compound **9**. Solvent peak at 7.26 ppm.

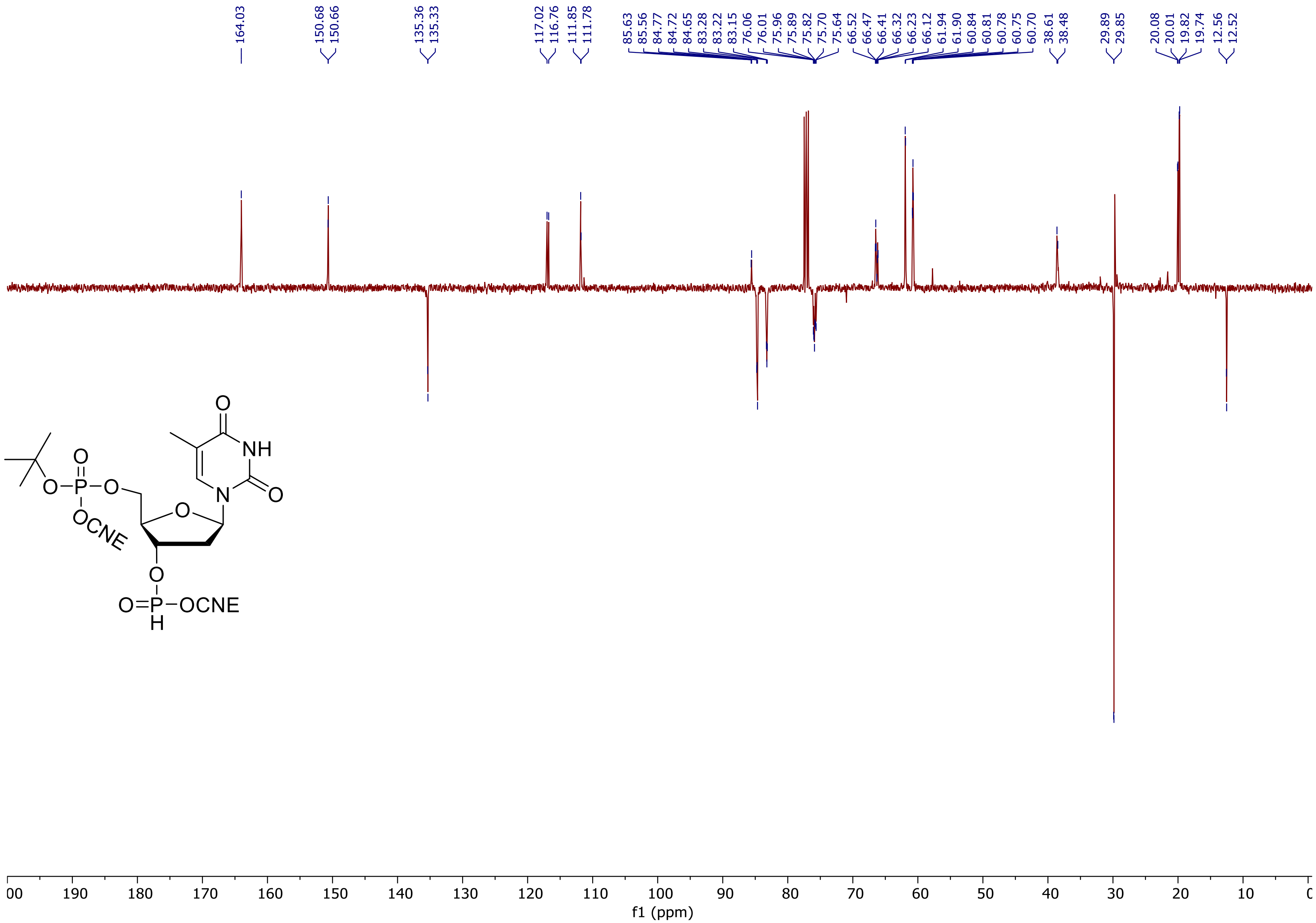

[13]**C-NMR** (101 MHz, $CDCl_3$) of compound **9**. Solvent peak at 77.16 ppm.

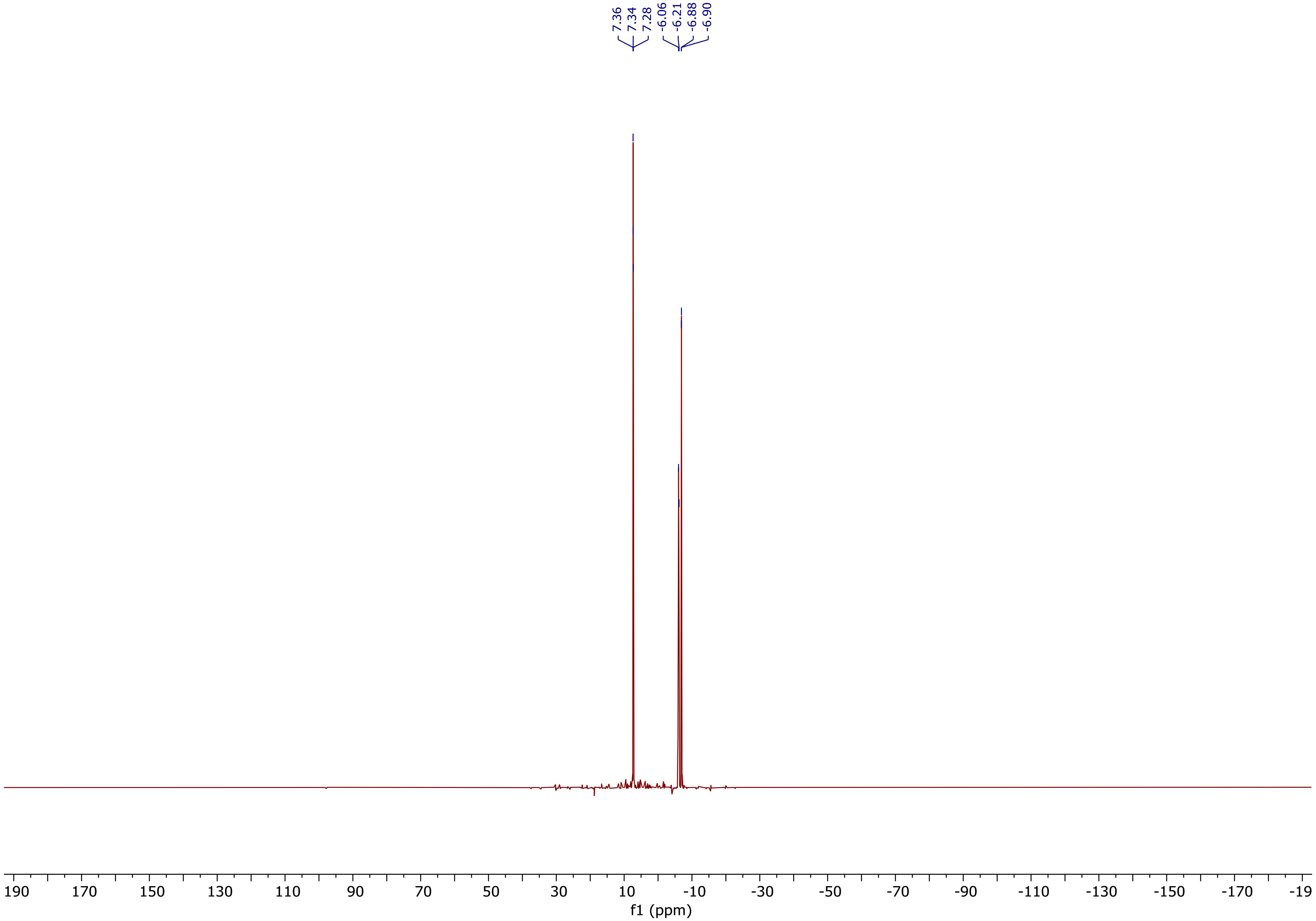

^{31}P-NMR (162 MHz, $CDCl_3$) of compound **9**.

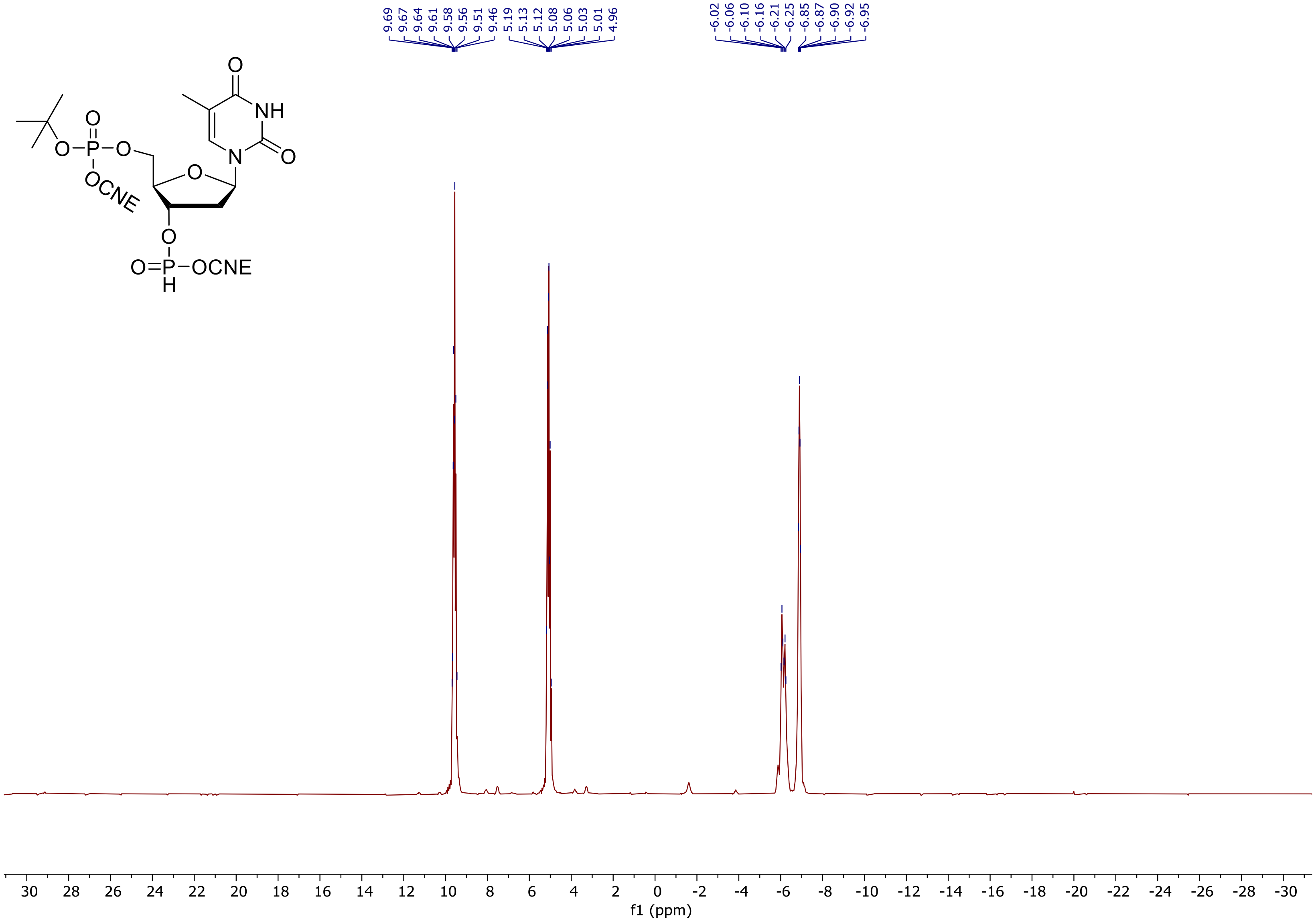

^{31}**P-NMR** (162 MHz, $CDCl_3$, proton coupled) of compound **9**.

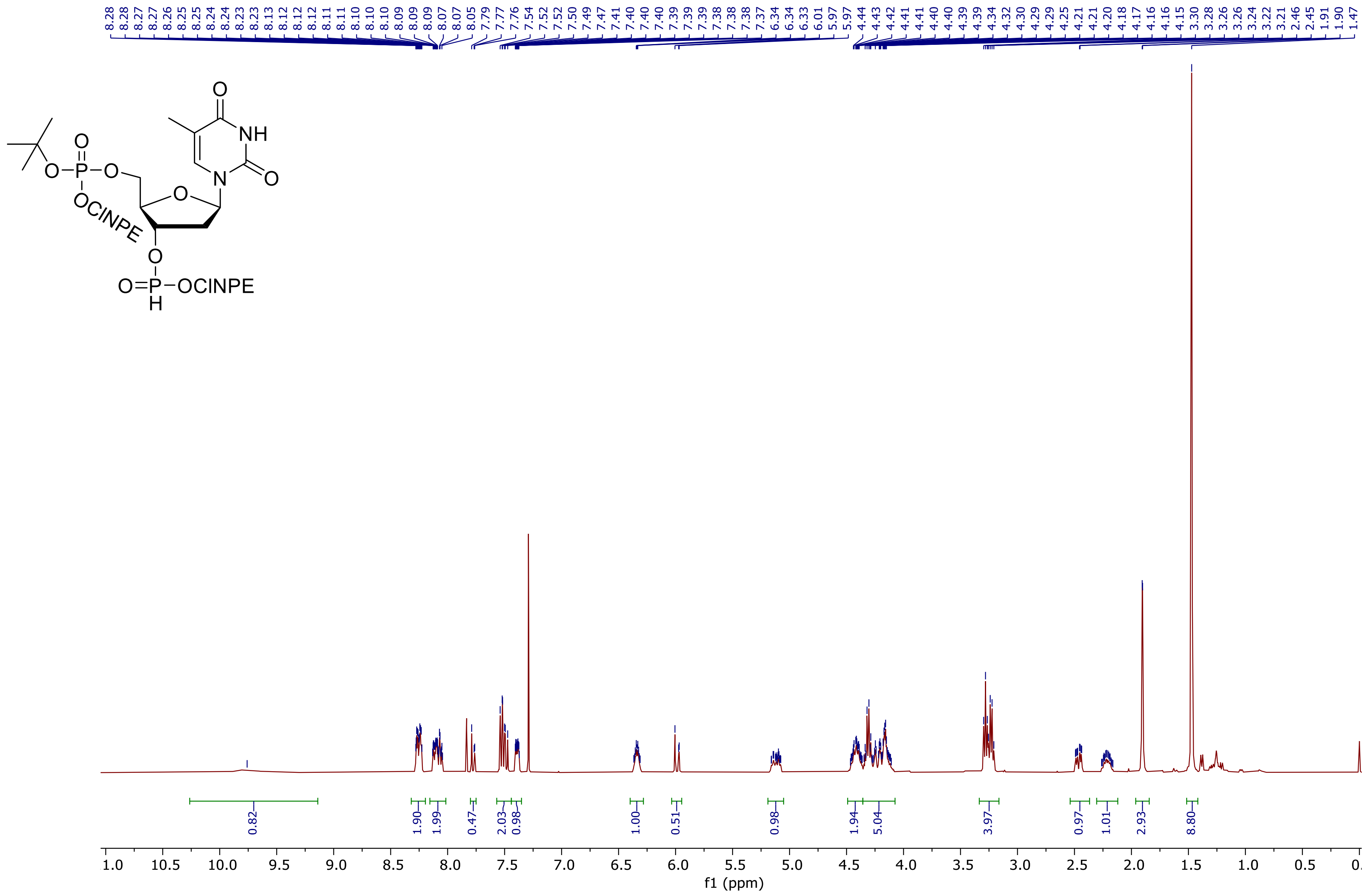

^{1}H-NMR (400 MHz, $CDCl_3$) of compound **10**. Solvent peak at 7.26 ppm.

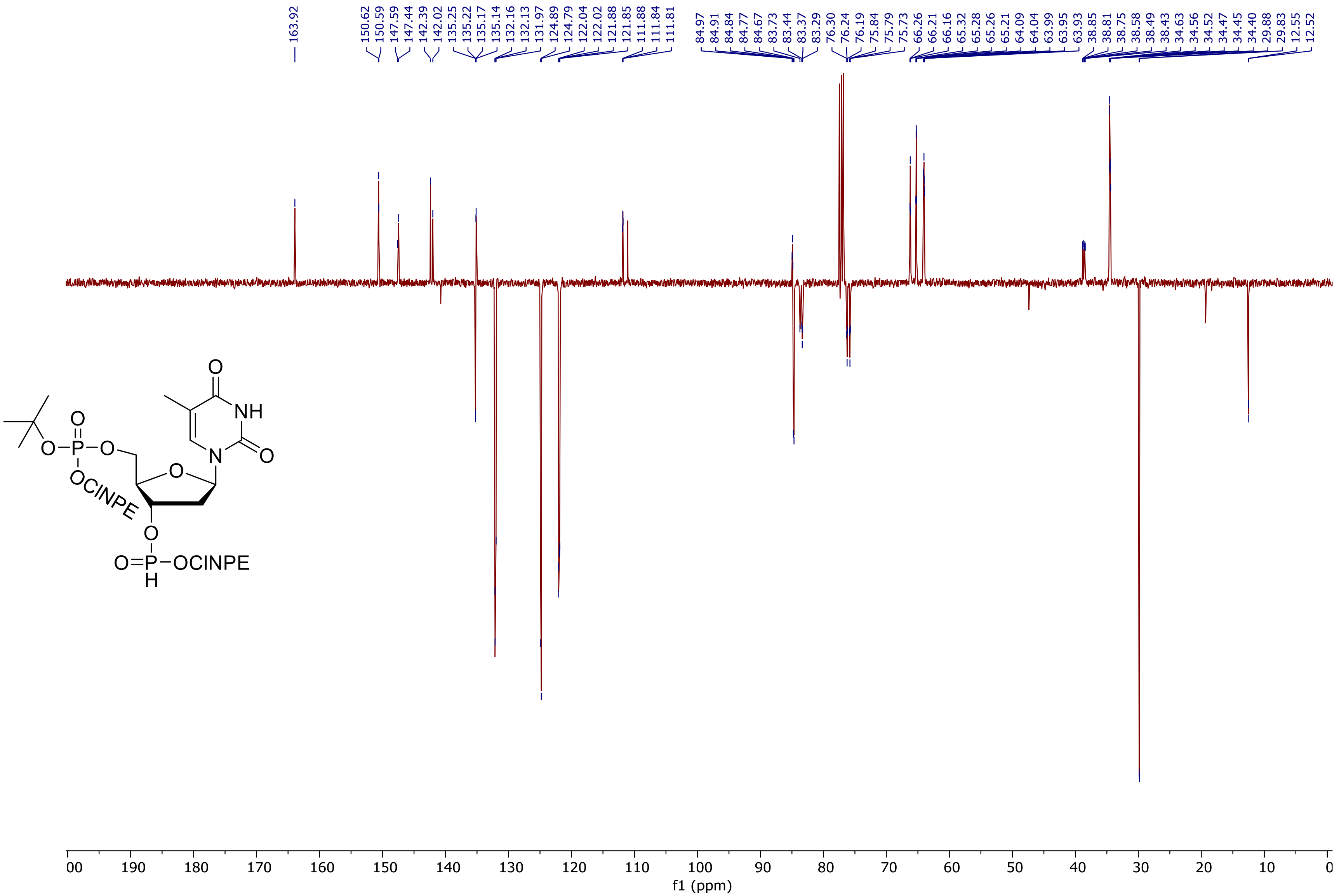

¹³C-NMR (101 MHz, $CDCl_3$) of compound **10**. Solvent peak at 77.16 ppm.

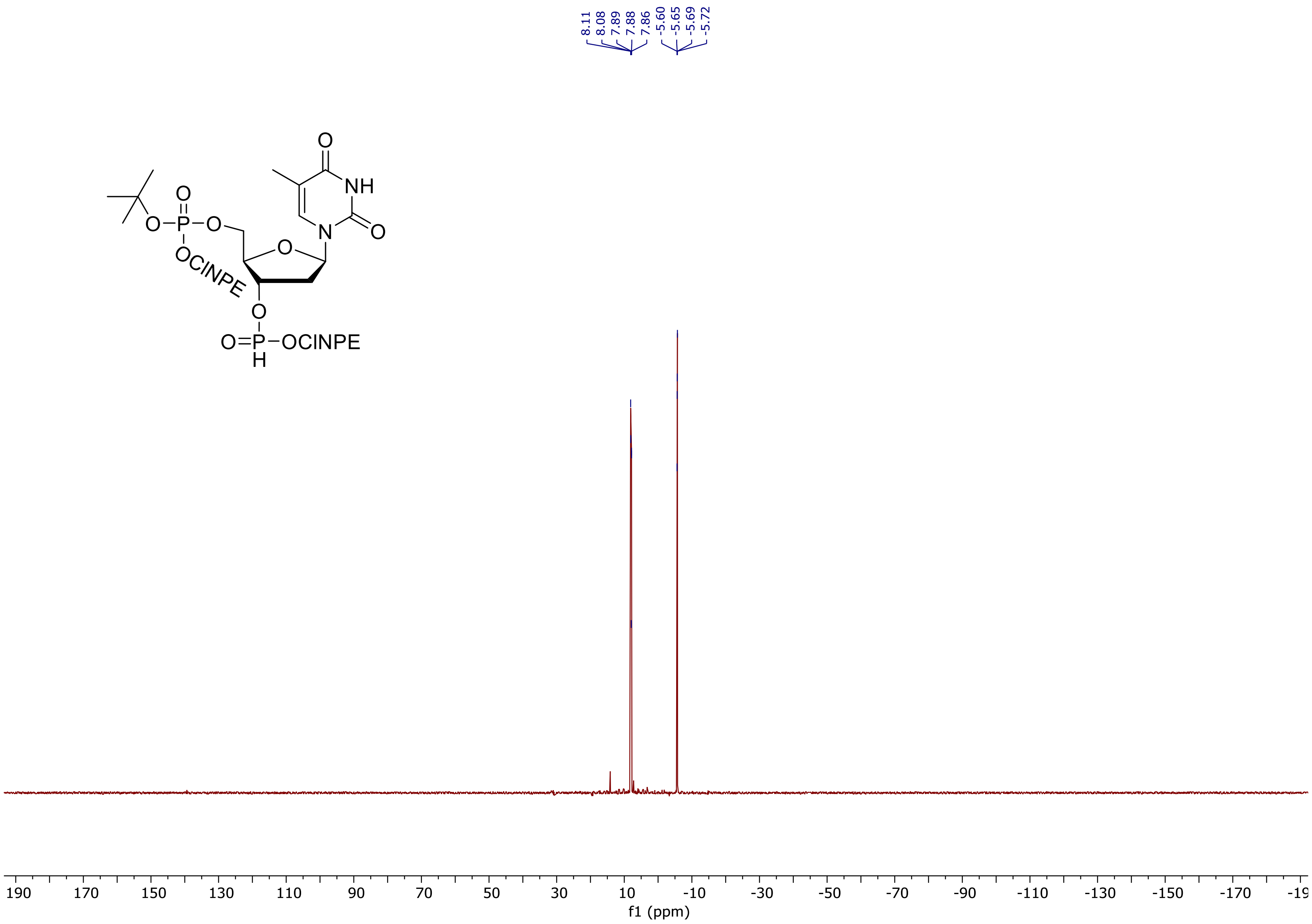

^{31}**P-NMR** (162 MHz, $CDCl_3$) of compound **10**.

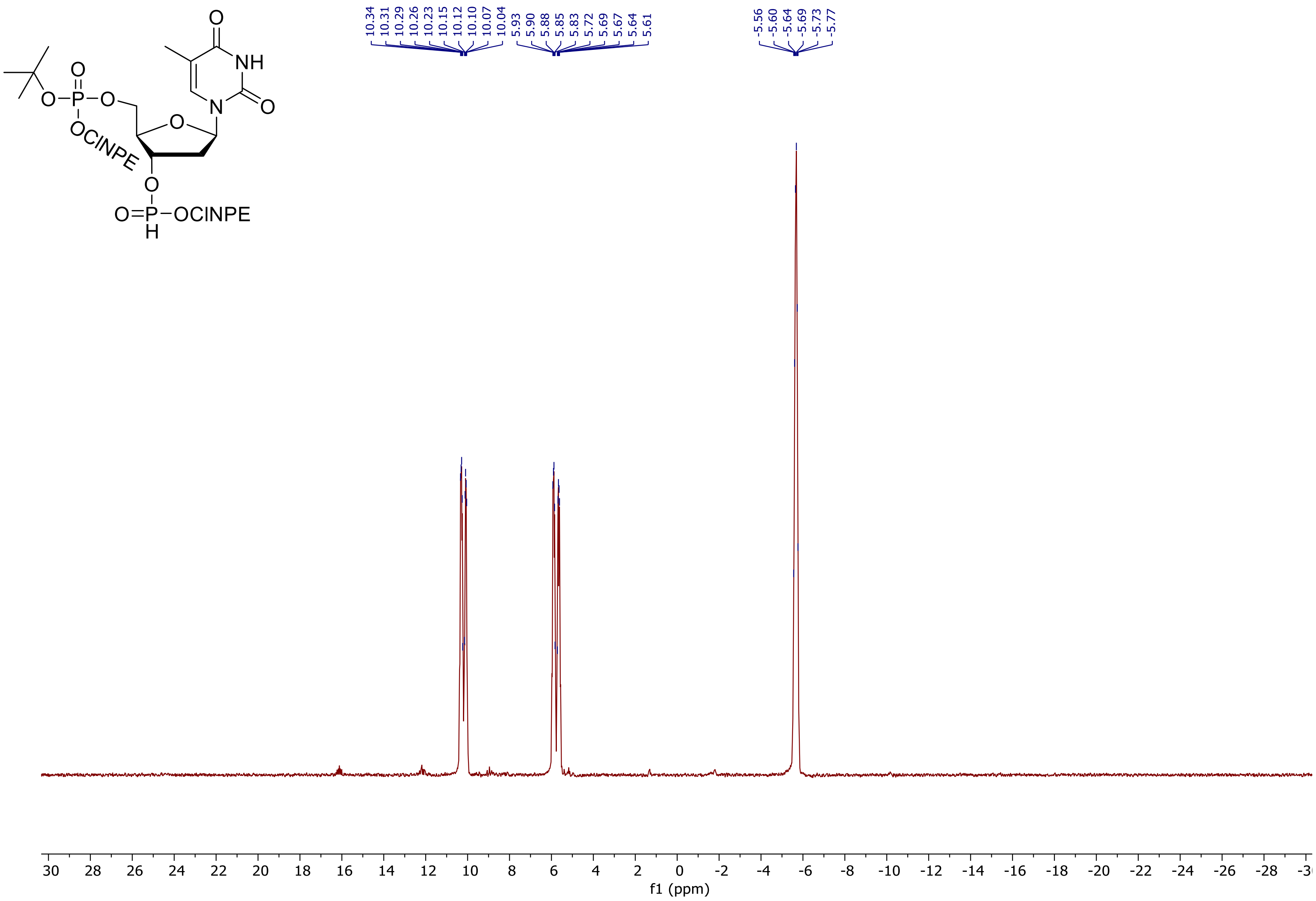

^{31}P-NMR (162 MHz, $CDCl_3$, proton coupled) of compound **10**.

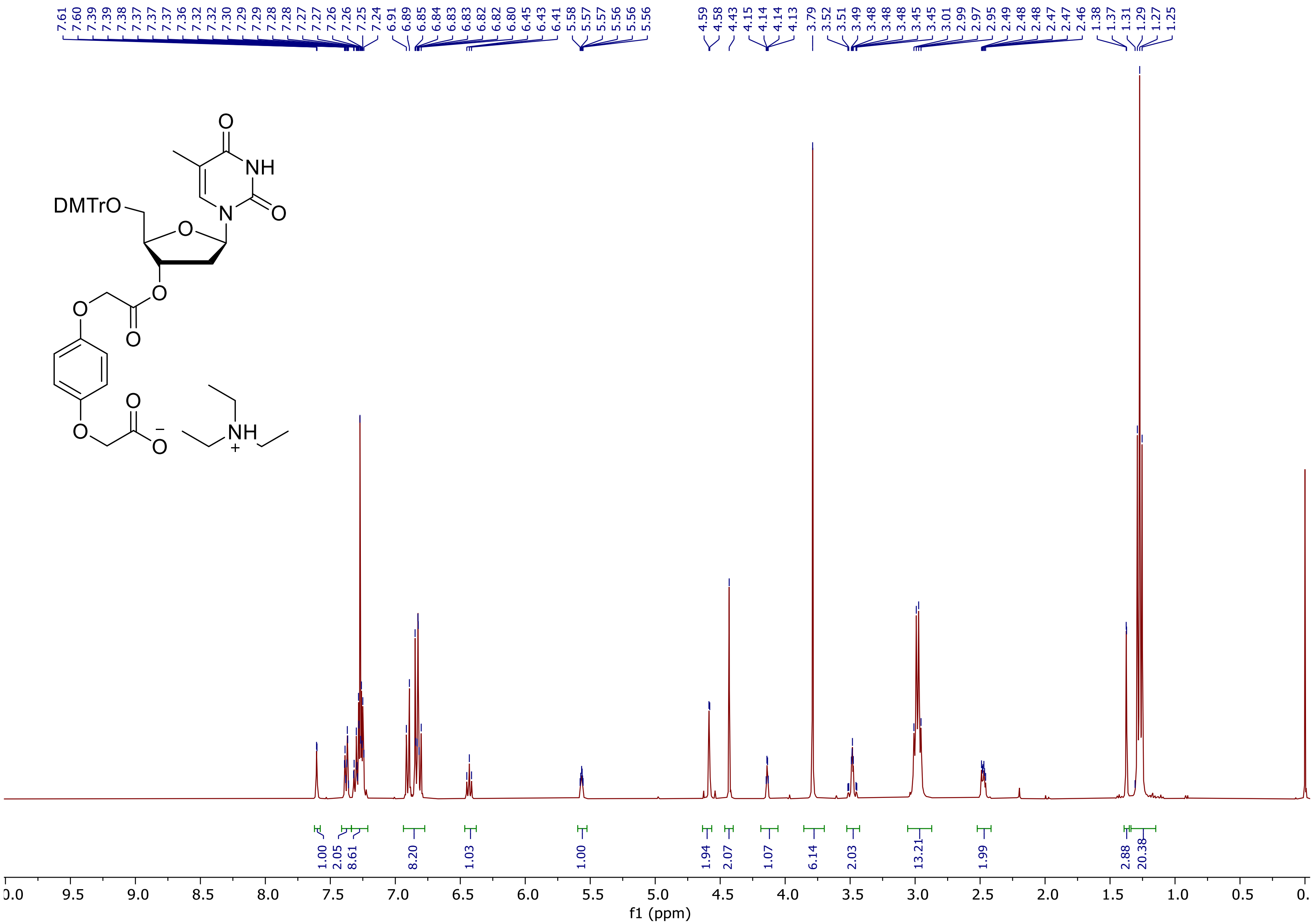

^{1}**H-NMR** (400 MHz, $CDCl_3$) of compound **65**. Solvent peak at 7.26 ppm.

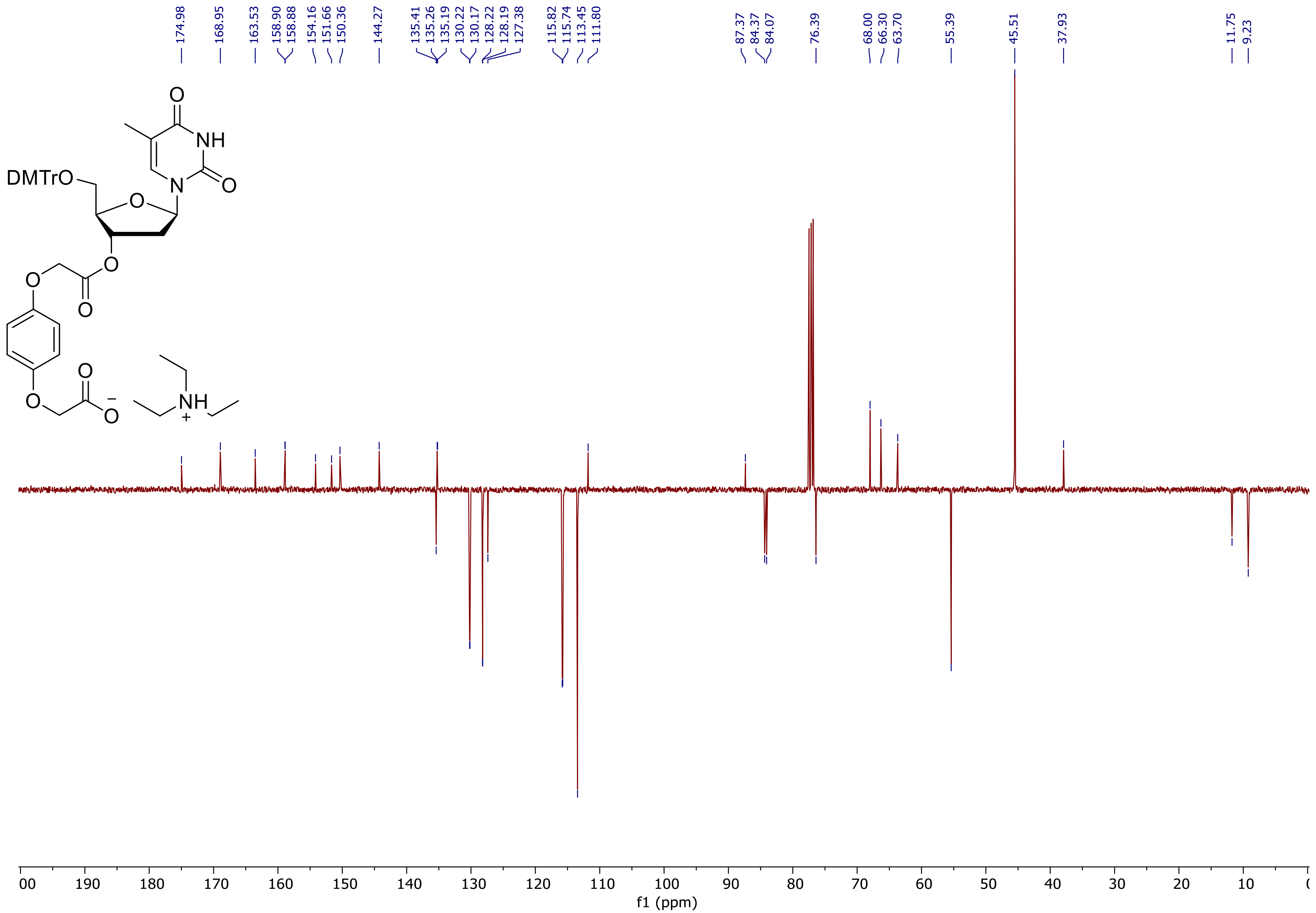

^{13}**C-NMR** (101 MHz, $CDCl_3$) of compound **65**. Solvent peak at 77.16 ppm.

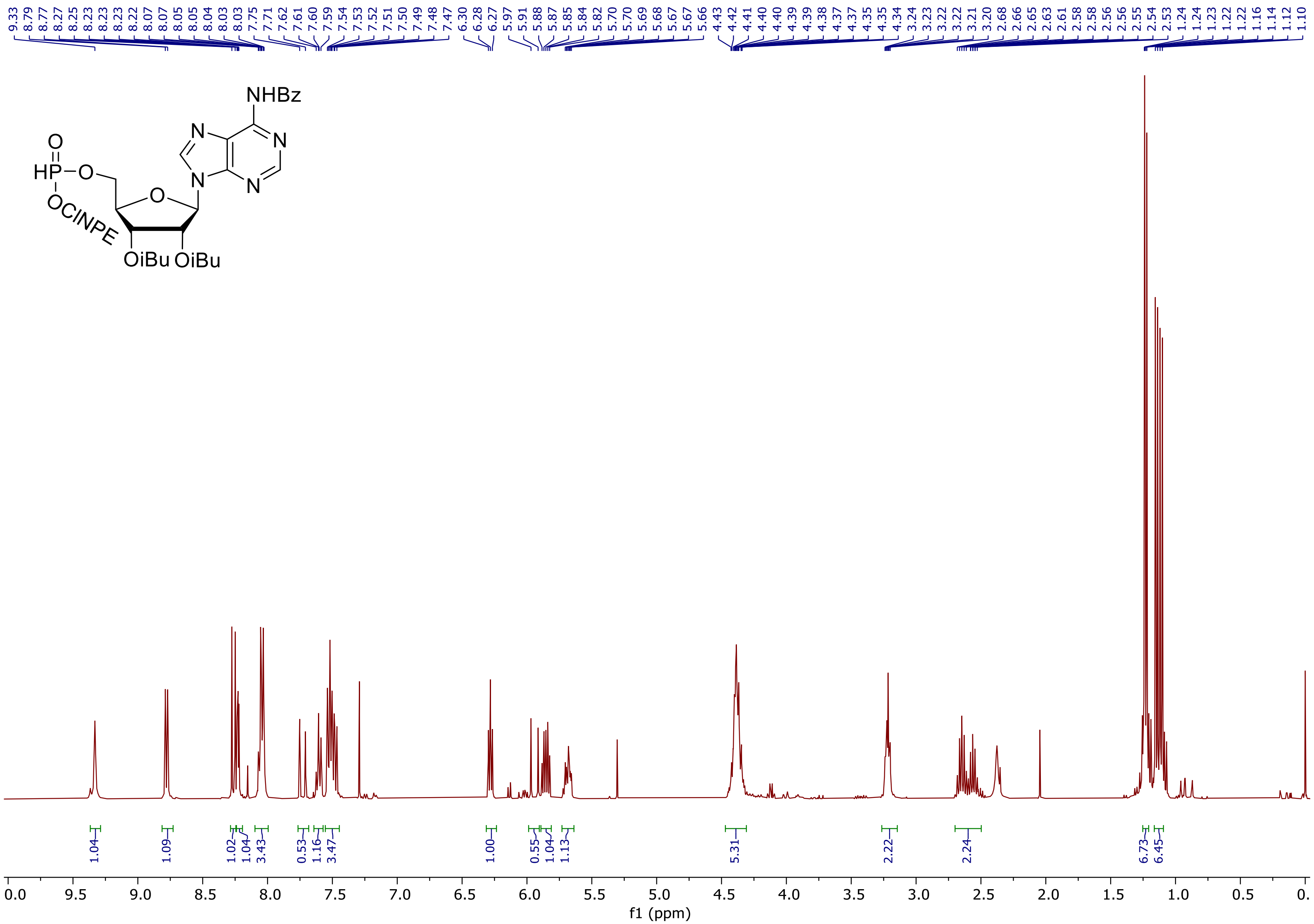

[1]**H-NMR** (400 MHz, $CDCl_3$) of compound **27**. Solvent peak at 7.26 ppm.

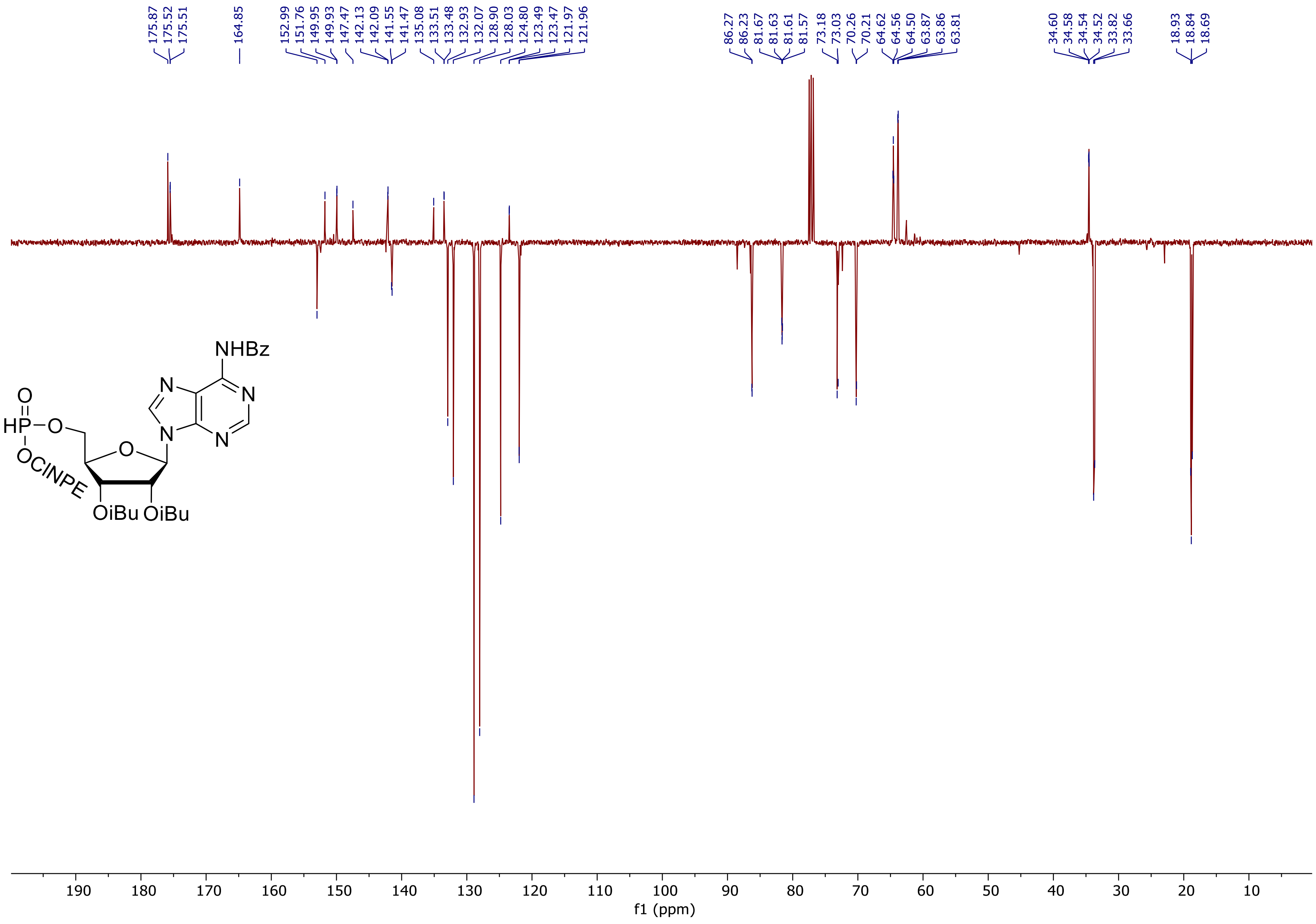

^{13}C-NMR (101 MHz, $CDCl_3$) of compound **27**. Solvent peak at 77.16 ppm.

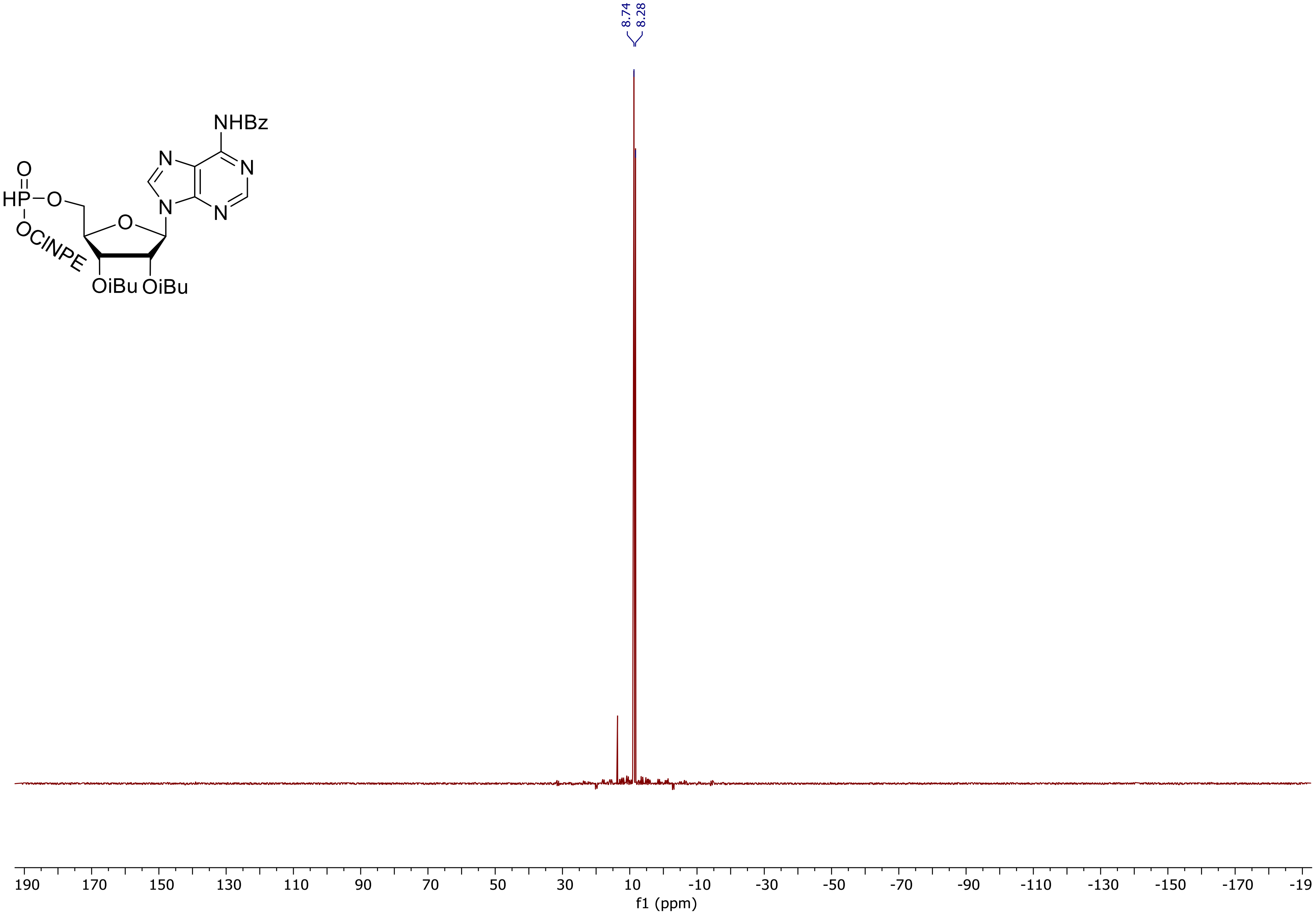

^{31}**P-NMR** (162 MHz, $CDCl_3$) of compound **27**.

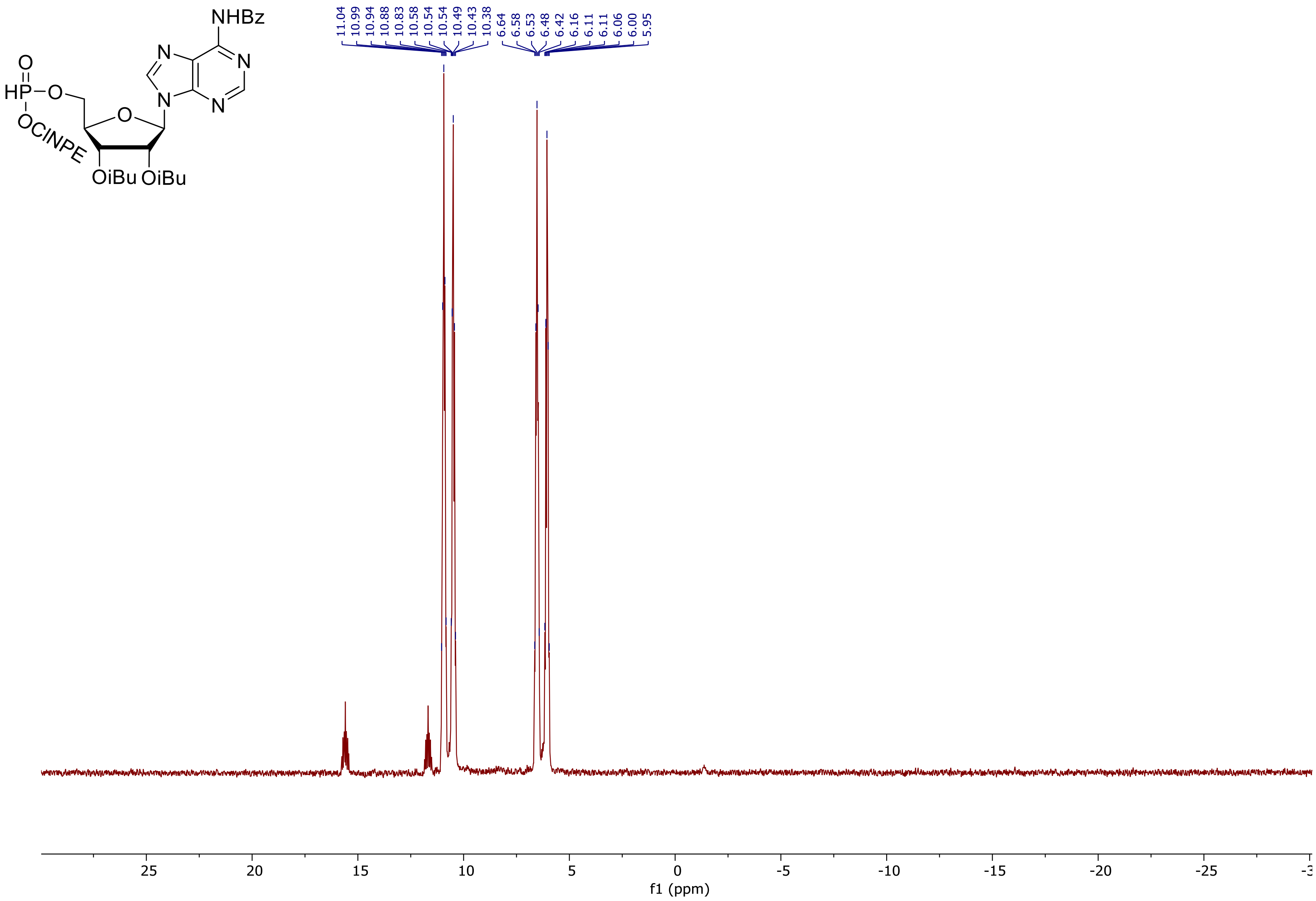

^{31}P-NMR (162 MHz, $CDCl_3$, proton coupled) of compound **27**.

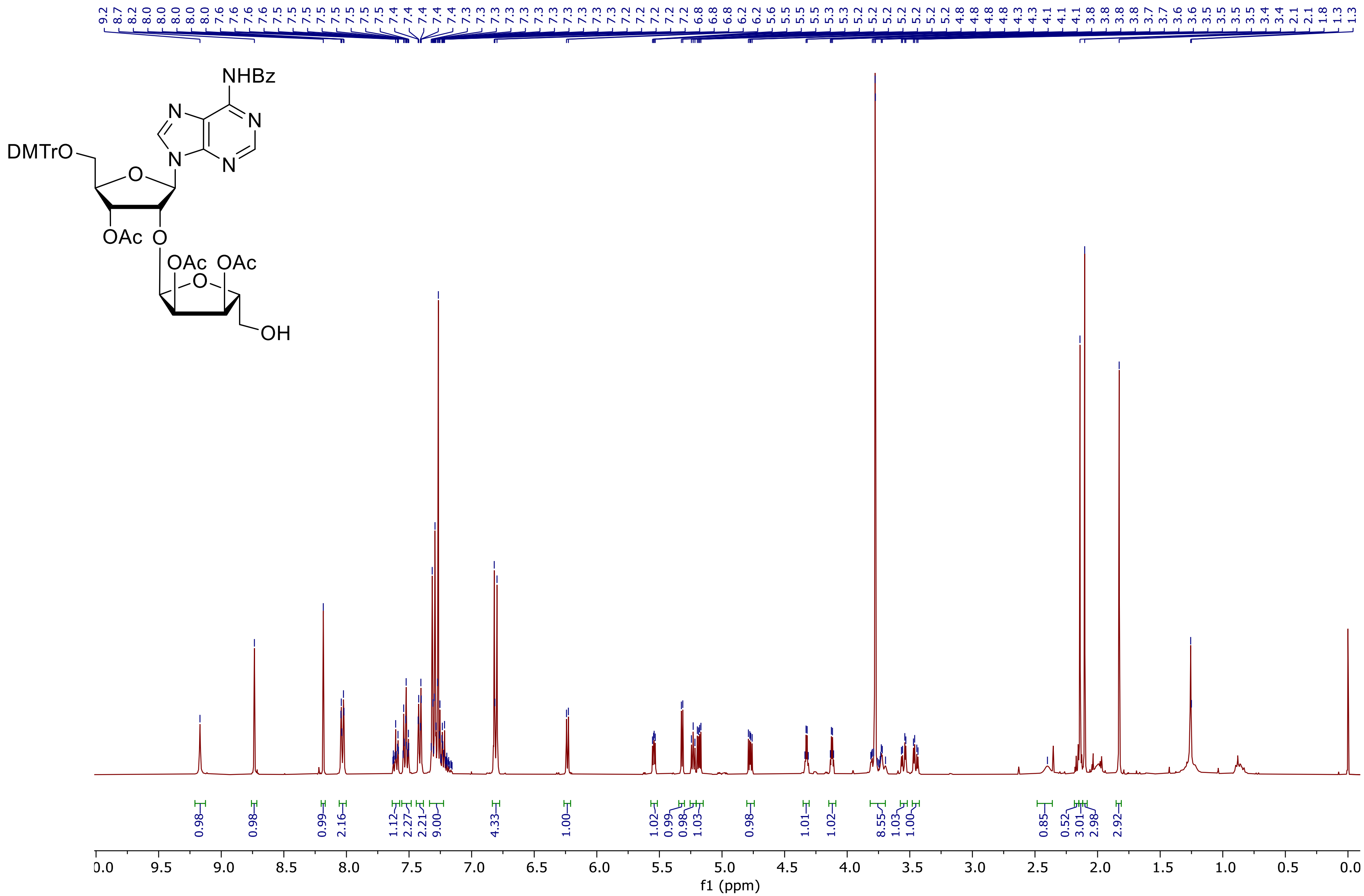

^{1}H-NMR (400 MHz, $CDCl_3$) of compound **32**. Solvent peak at 7.26 ppm.

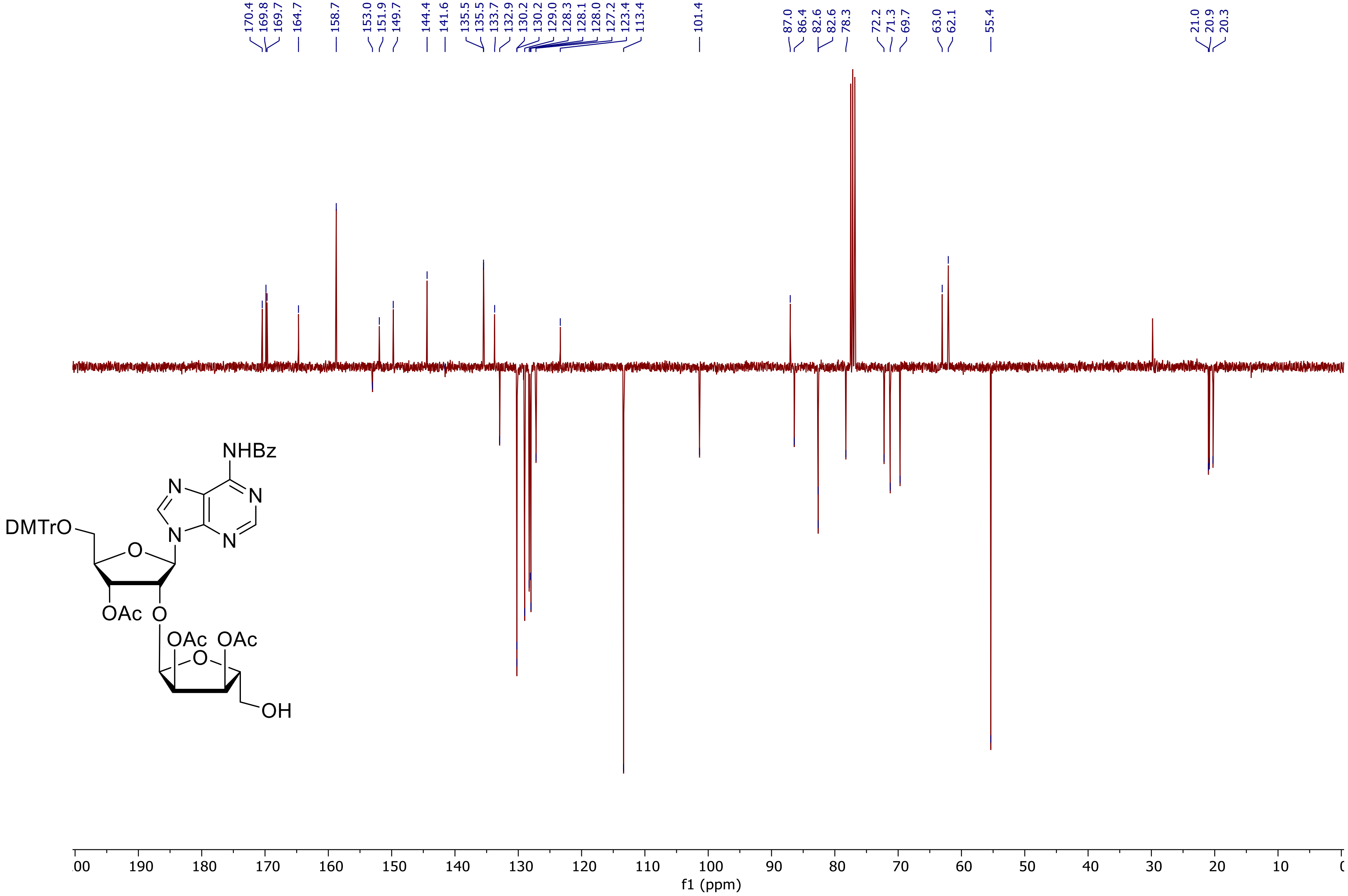

^{13}C-NMR (101 MHz, $CDCl_3$) of compound **32**. Solvent peak at 77.16 ppm.

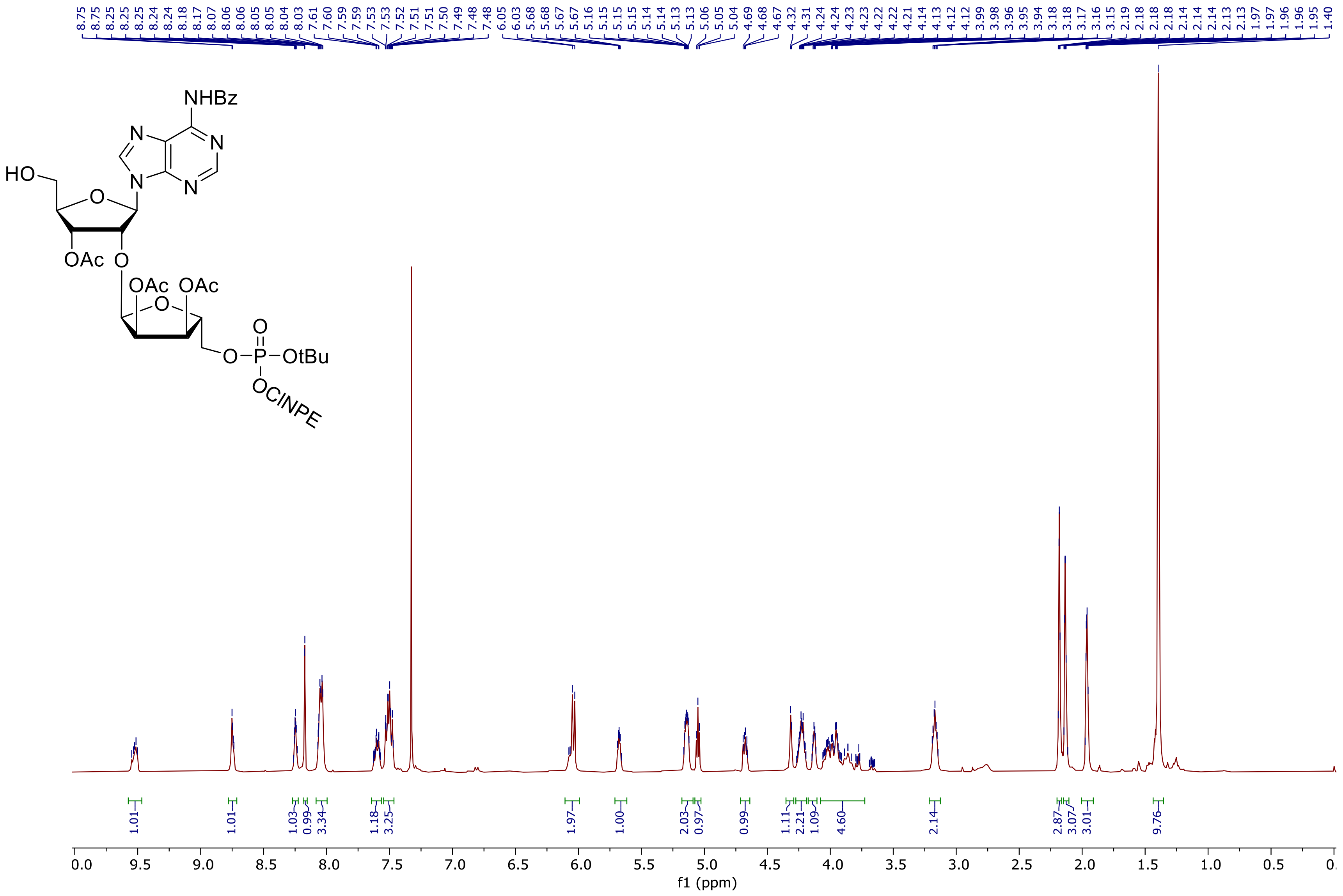

^{1}**H-NMR** (400 MHz, $CDCl_3$) of compound **33**. Solvent peak at 7.26 ppm.

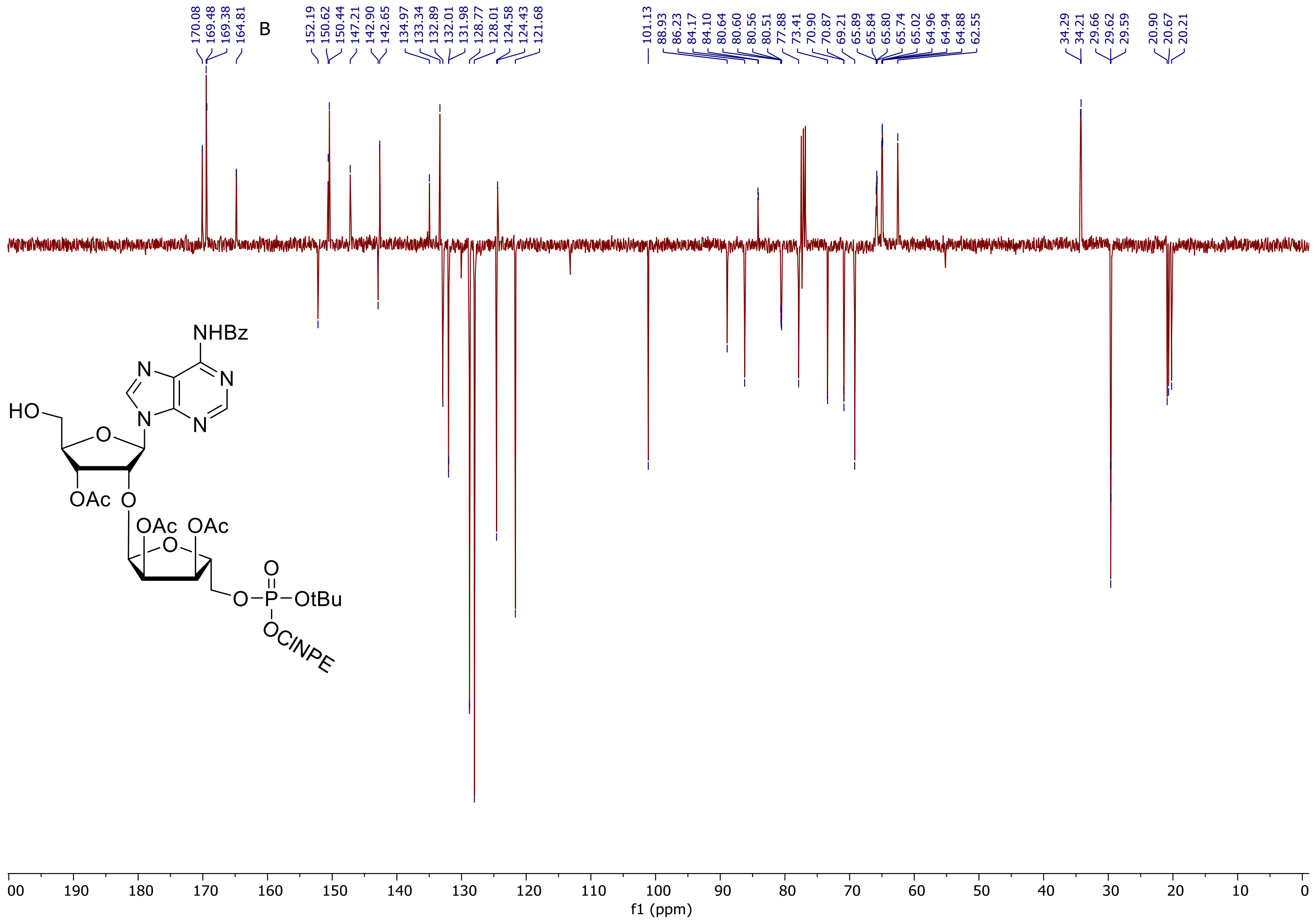

^{13}C-NMR (101 MHz, $CDCl_3$) of compound **33**. Solvent peak at 77.16 ppm.

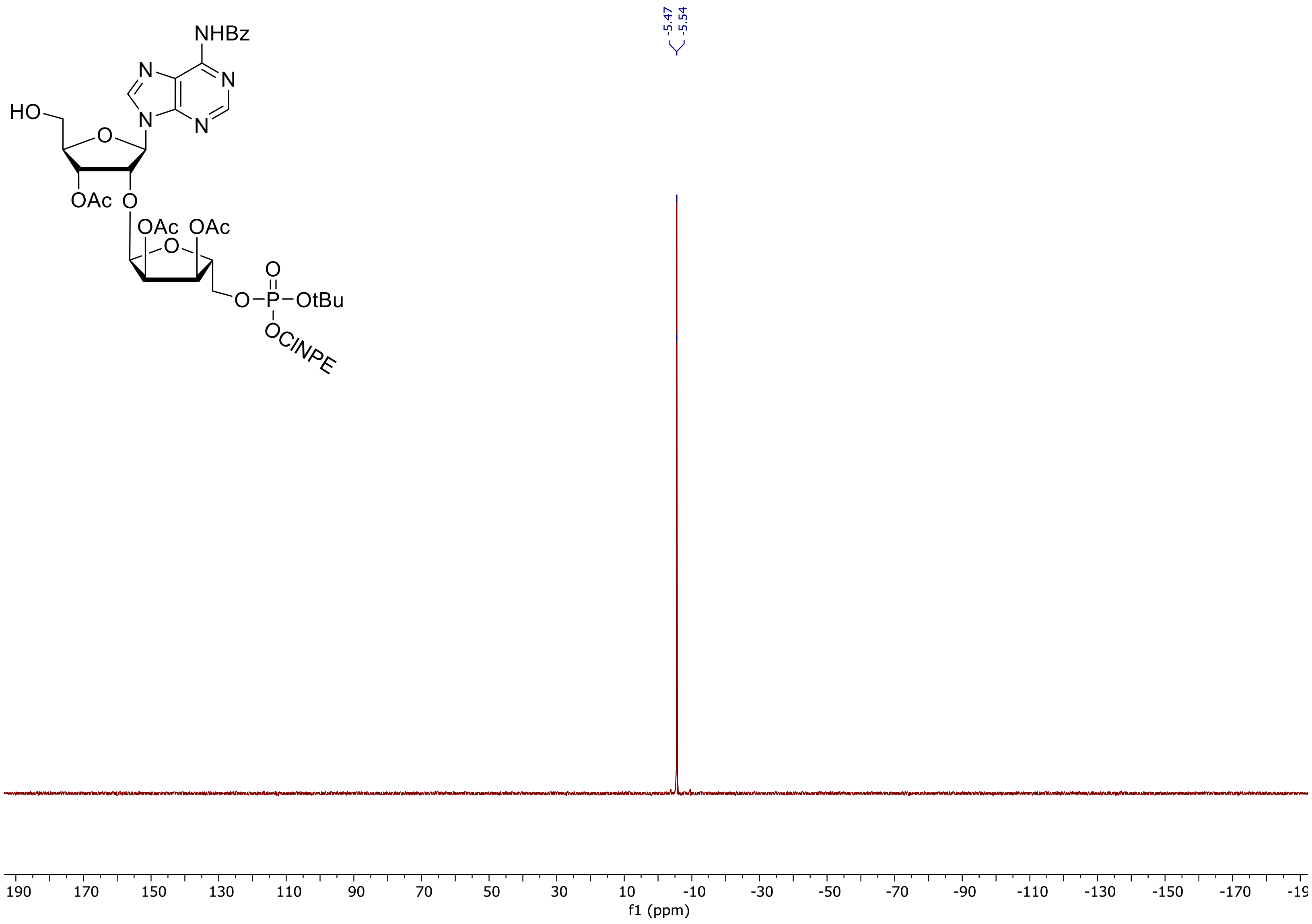

^{31}**P-NMR** (162 MHz, $CDCl_3$) of compound **33**.

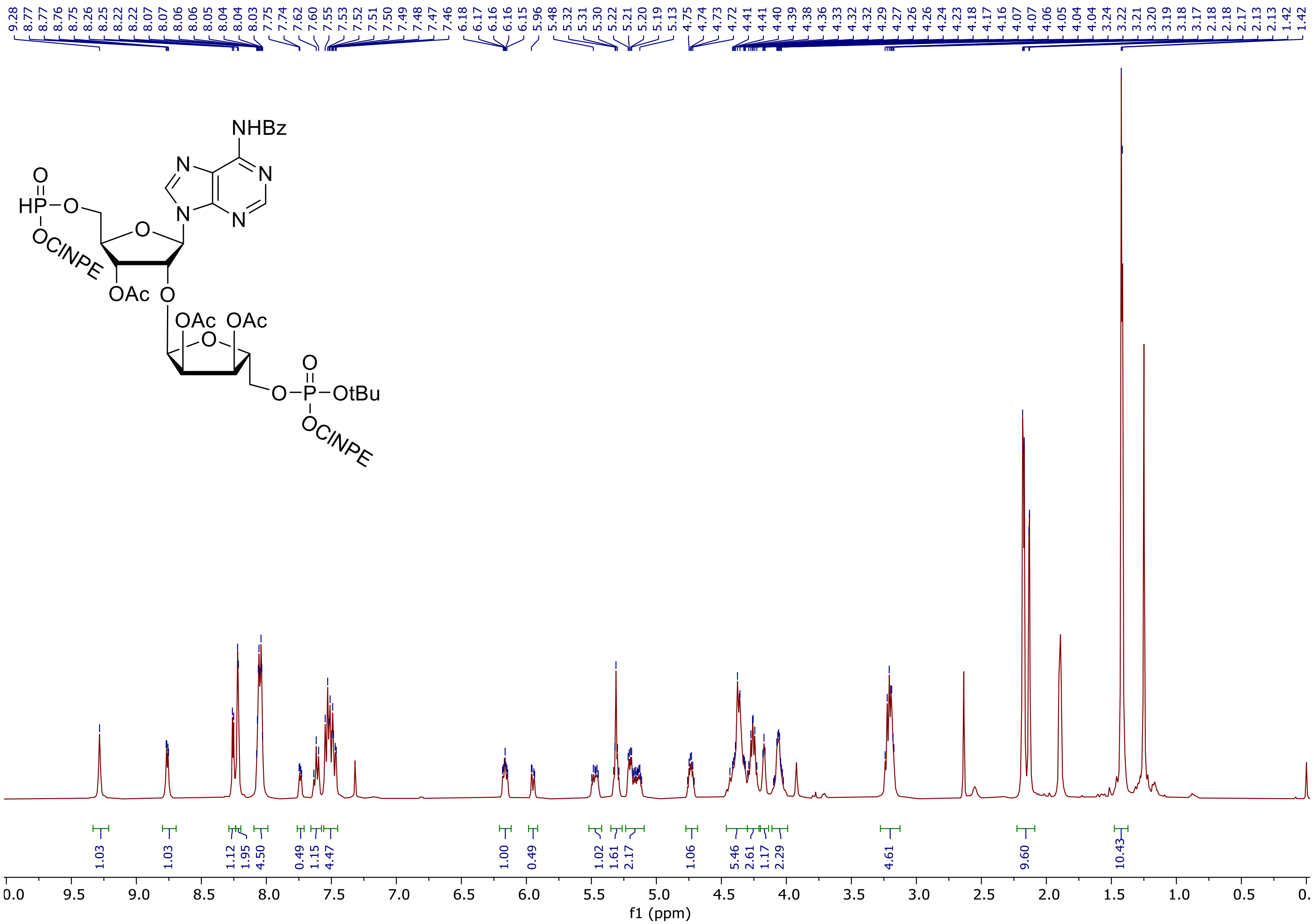

[1]**H-NMR** (400 MHz, $CDCl_3$) of compound **29**. Solvent peak at 7.26 ppm.

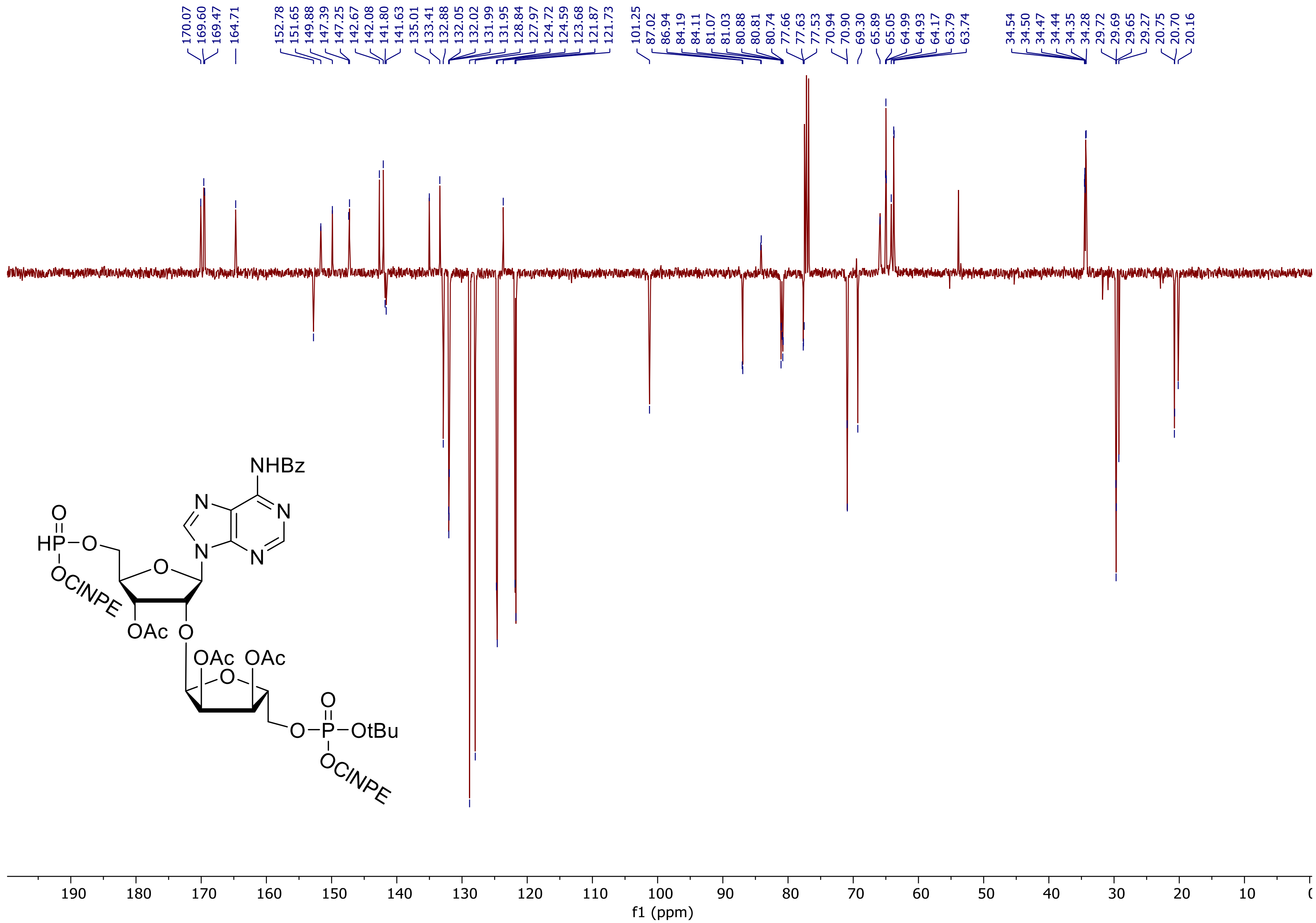

[13]**C-NMR** (101 MHz, $CDCl_3$) of compound **29**. Solvent peak at 77.16 ppm.

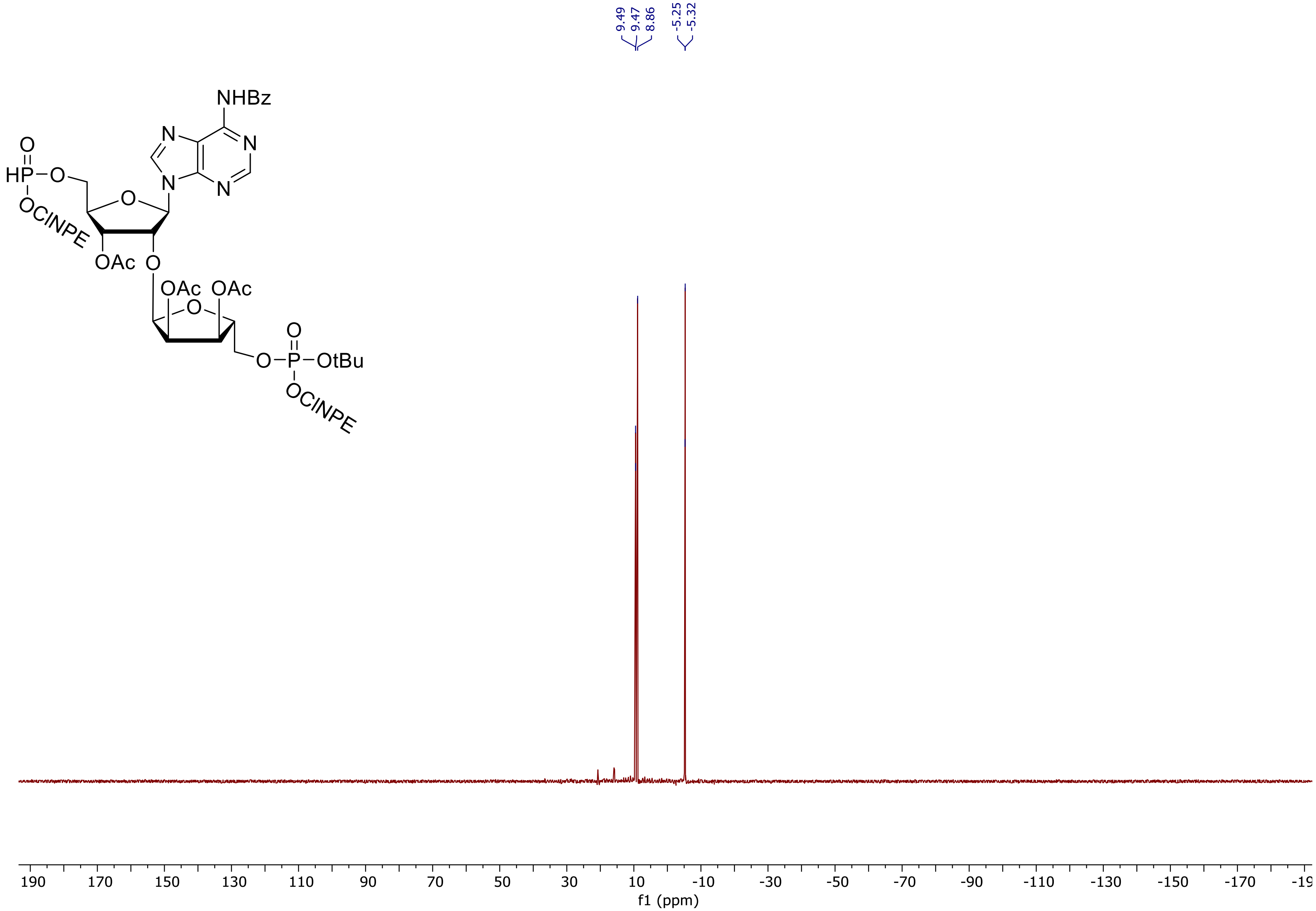

^{31}P-NMR (162 MHz, $CDCl_3$) of compound **27**.

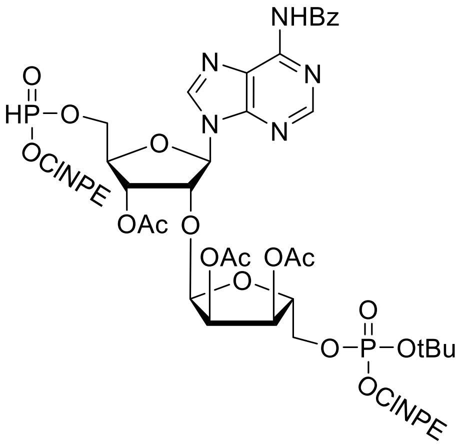

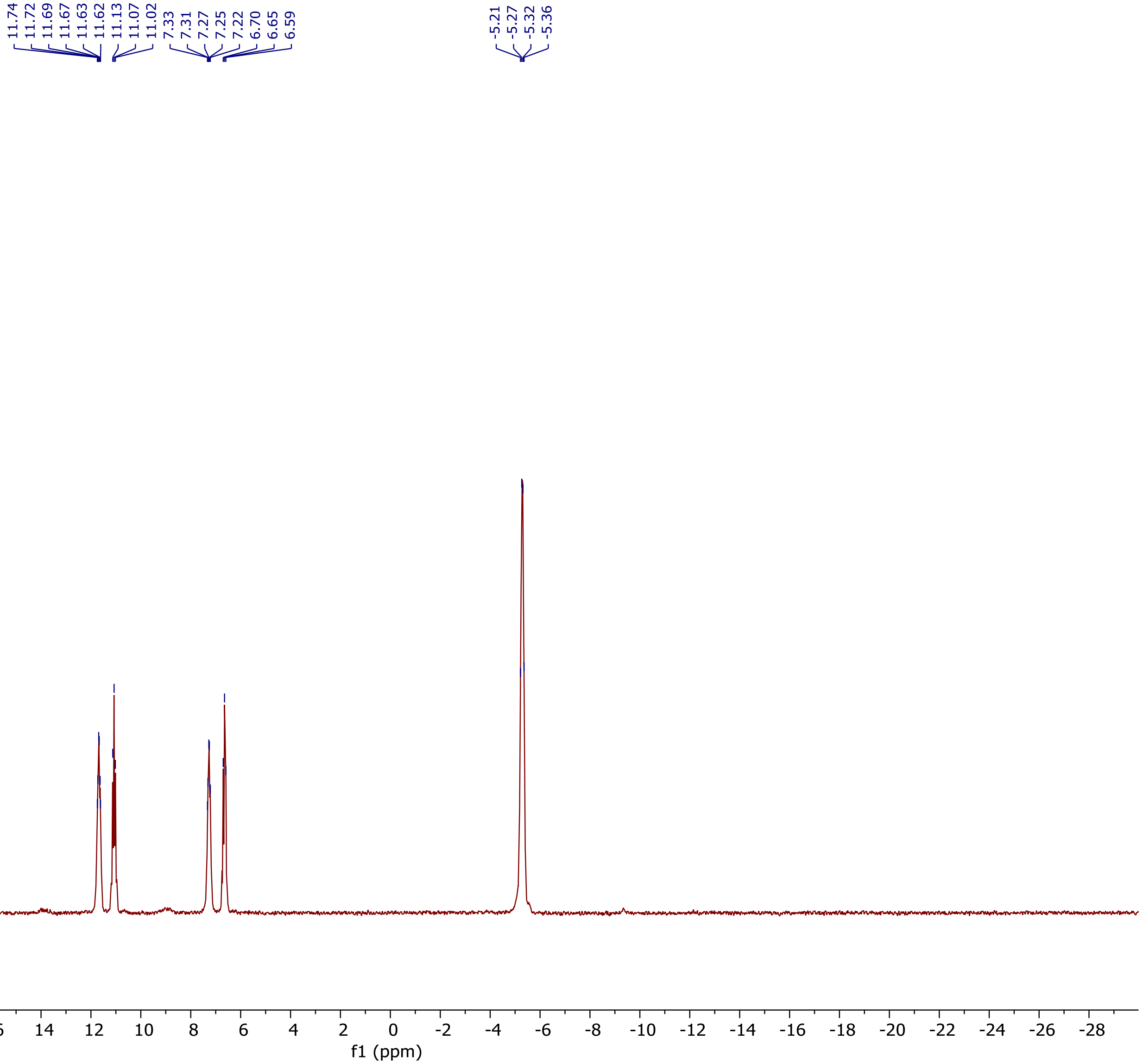

^{31}**P-NMR** (162 MHz, $CDCl_3$, proton coupled) of compound **29**.

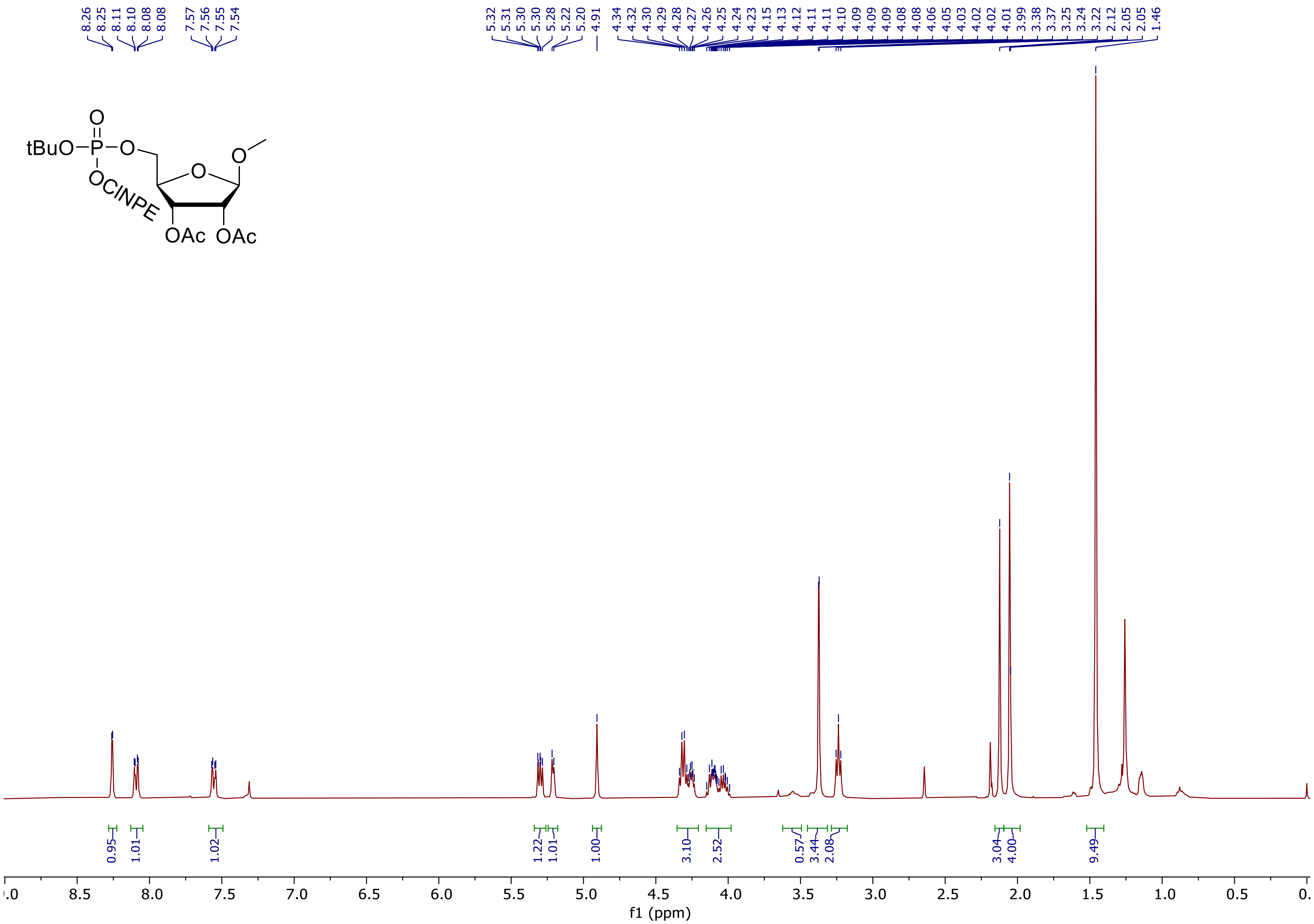

^{1}H-NMR (400 MHz, $CDCl_3$) of compound **46**. Solvent peak at 7.26 ppm.

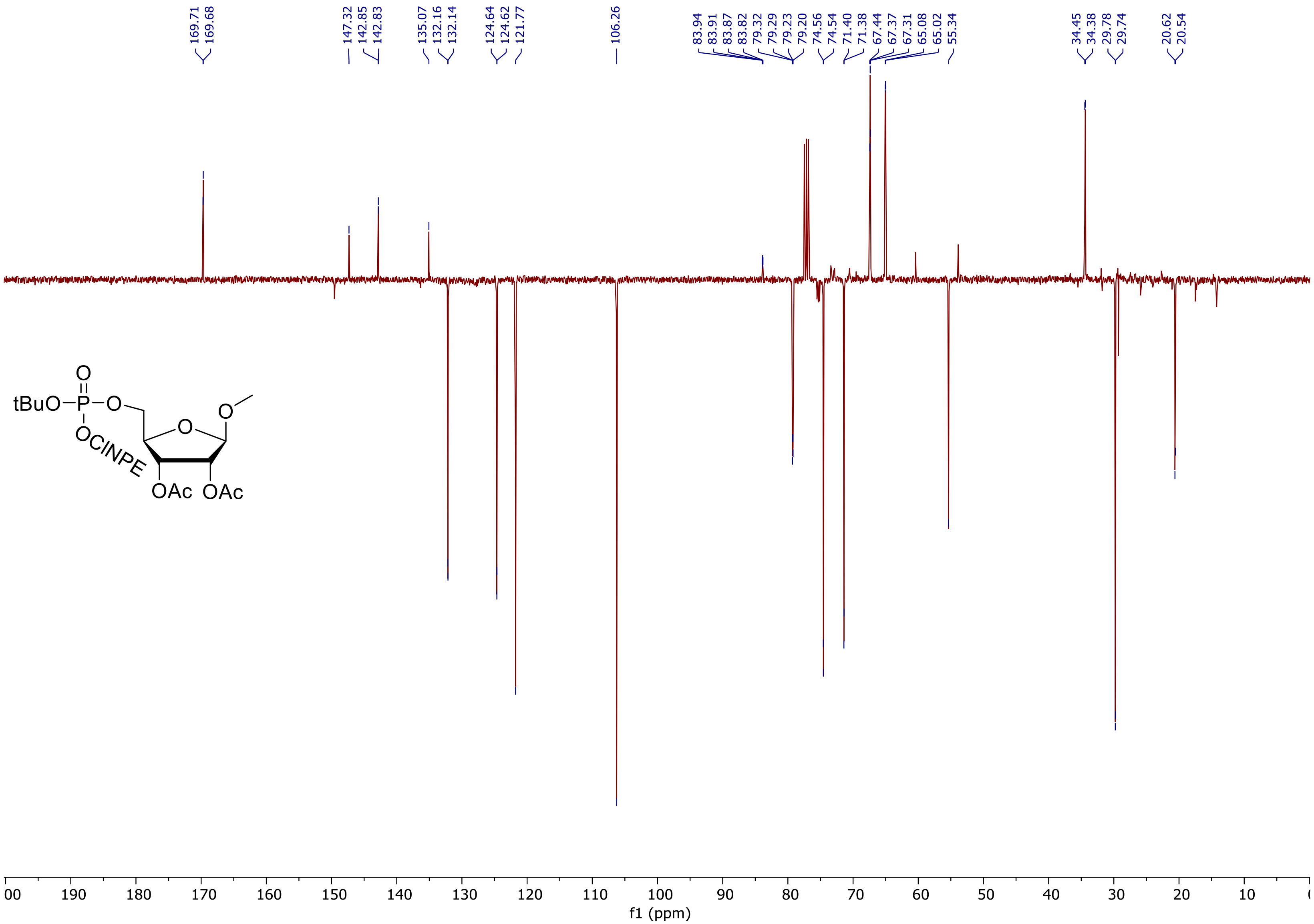

^{13}C-NMR (101 MHz, $CDCl_3$) of compound **46**. Solvent peak at 77.16 ppm.

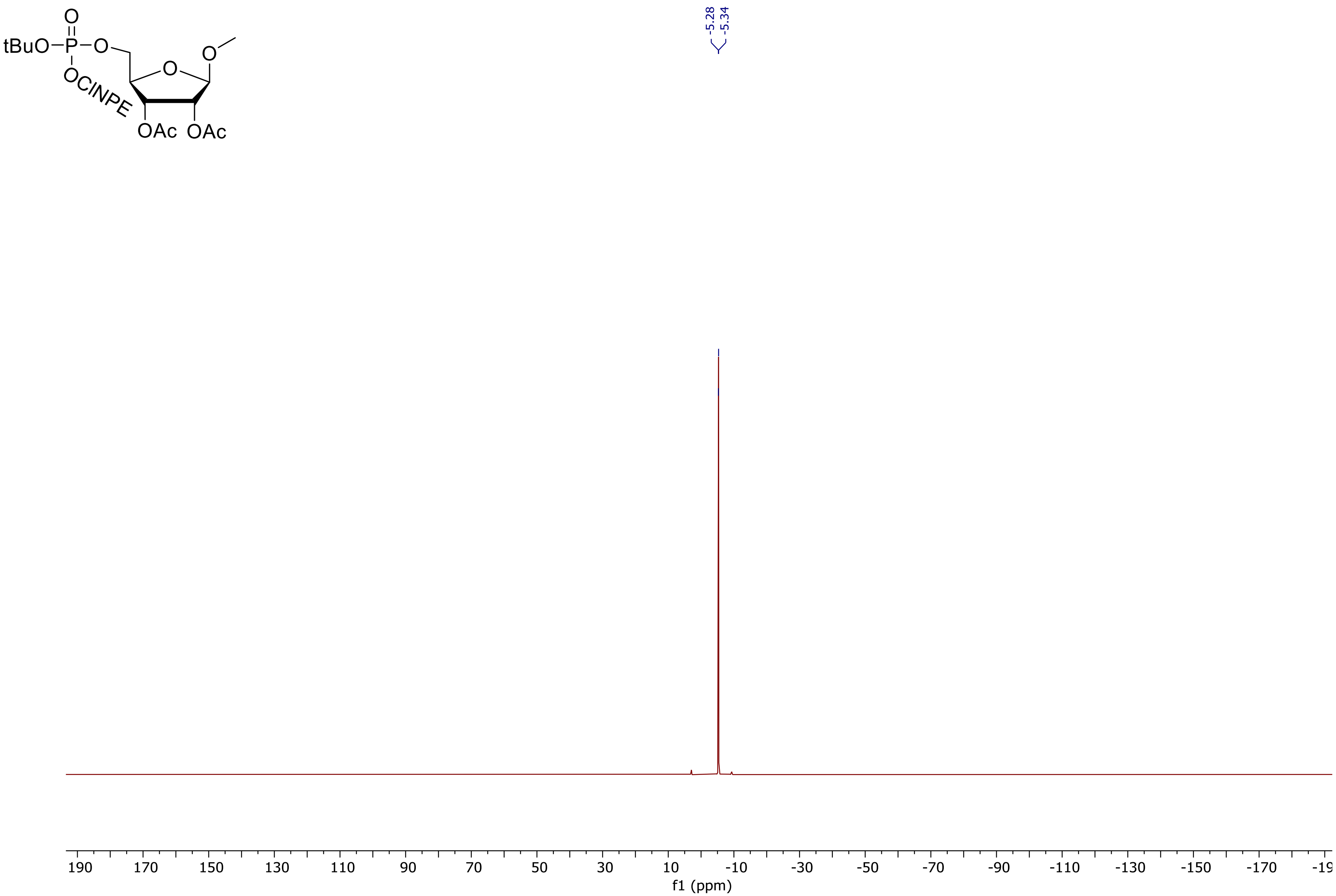

^{31}P-NMR (162 MHz, $CDCl_3$) of compound **46**.
