# Supplementary material for: Fully protected pyrophosphates via phosphorobromidates for the synthesis of biopolymers
Source: Chem Sci. 2026 Mar 9;17(17):8630–7. doi: 10.1039/d6sc01119e (PMC12970391; doi:10.1039/d6sc01119e)
Supplement: SC-017-D6SC01119E-s002 [file SC-017-D6SC01119E-s002.pdf]

Supplementary Information (SI) for Chemical Science.
This journal is © The Royal Society of Chemistry 2026

Fully protected pyrophosphates *via* phosphorobromidates for the synthesis of biopolymers

*Sven Wijngaarden, Femke L. A. M. van der Heijden, Koen J. Rijpkema, Bob van Puffelen, Nico J. Meeuwenoord, Danicia Ramcharan, Gijsbert A. van der Marel, Jeroen D. C. Codée, Herman S. Overkleeft and Dmitri V. Filippov**

Leiden Institute of Chemistry, Leiden University, Einsteinweg 55, 2333 CC, Leiden, The Netherlands E-mail: filippov@chem.leidenuniv.nl

Table of contents:

Supplementary Figures 3

Experimental Section 8

General synthetic procedures 8

Building block synthesis 9

Solution phase synthesis of protected pyrophosphates 20

Solid-phase synthesis 23

Optimization of protecting group strategy (Table 1). 24

Solid-phase synthesis of oligo-TDP oligomers 26

Atherton-Todd oxidation of H-phosphonates followed by ^{31}P-NMR 29

Supplementary Figures

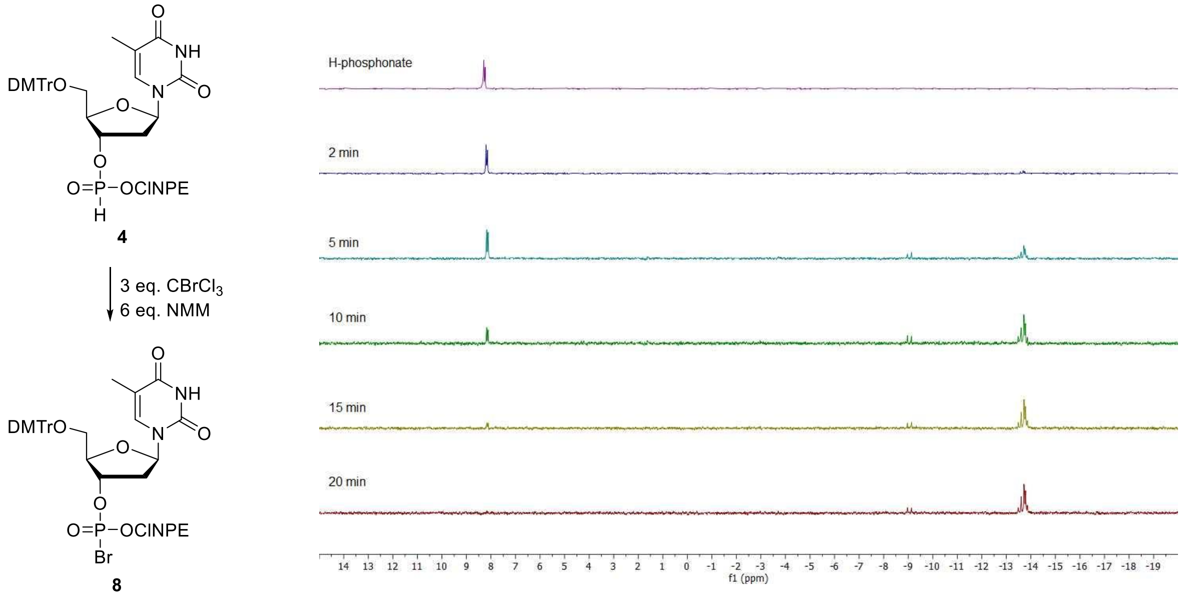

Figure S1. H-phosphonate activation of ClNPE protected H-phosphonate **4** by $CBrCl_3$ and NMM, measured over time by ^{31}P-NMR. Chemical shifts: H-phosphonate **4** (9 ppm), phosphobromidate **8** (-9 ppm), symmetric pyrophosphotetraester of type **VII** (-14 ppm).

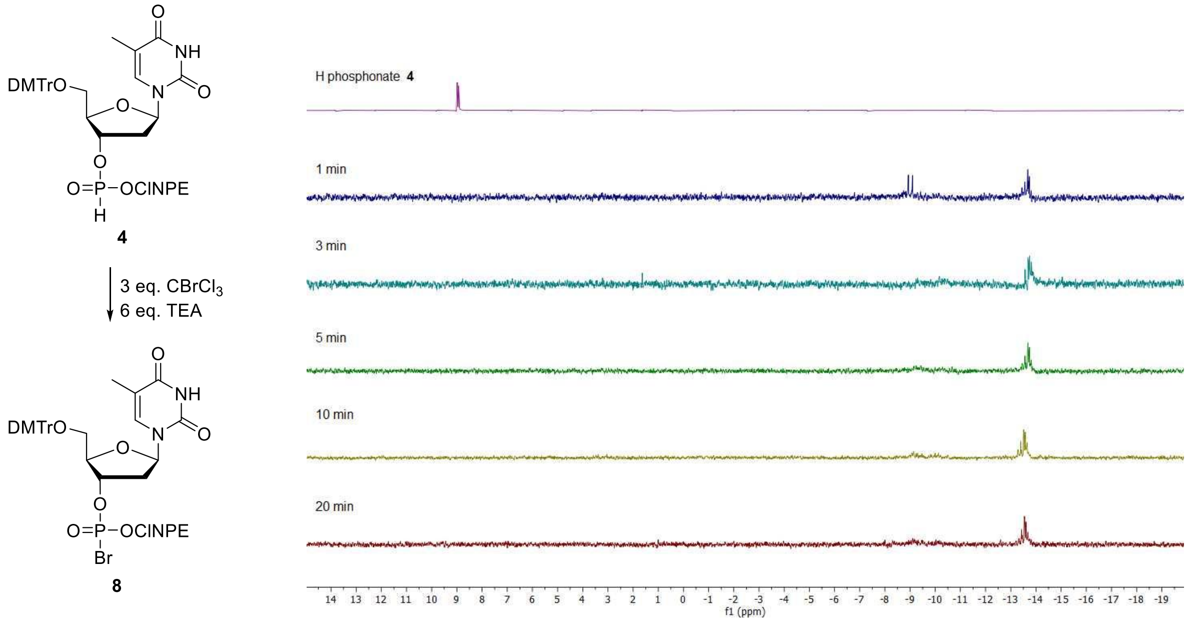

Figure S2. H-phosphonate activation of ClNPE protected H-phosphonate **4** by $CBrCl_3$ and TEA, measured over time by ^{31}P-NMR. Chemical shifts: H-phosphonate **4** (9 ppm), phosphobromidate **8** (-9 ppm), symmetric pyrophosphotetraester of type **VII** (-14 ppm).

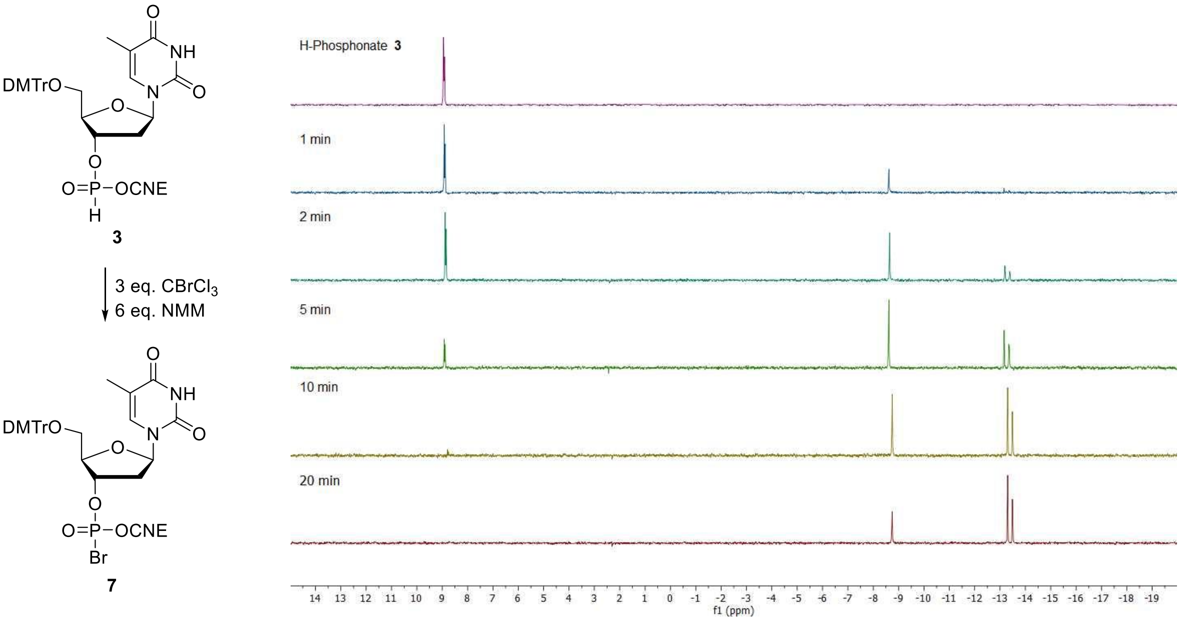

Figure S3. H-phosphonate activation of CNE protected H-phosphonate **3** measured over time, followed by ^{31}P-NMR. For the oxidation reaction, 2 eq. of $CBrCl_3$ as halogenating agent and 3 eq. of NMM as base were used. H-phosphonate **3** (9ppm), phosphor bromidate **7** (-9 ppm), symmetric pyrophosphotetraester **VII** (-14 ppm).

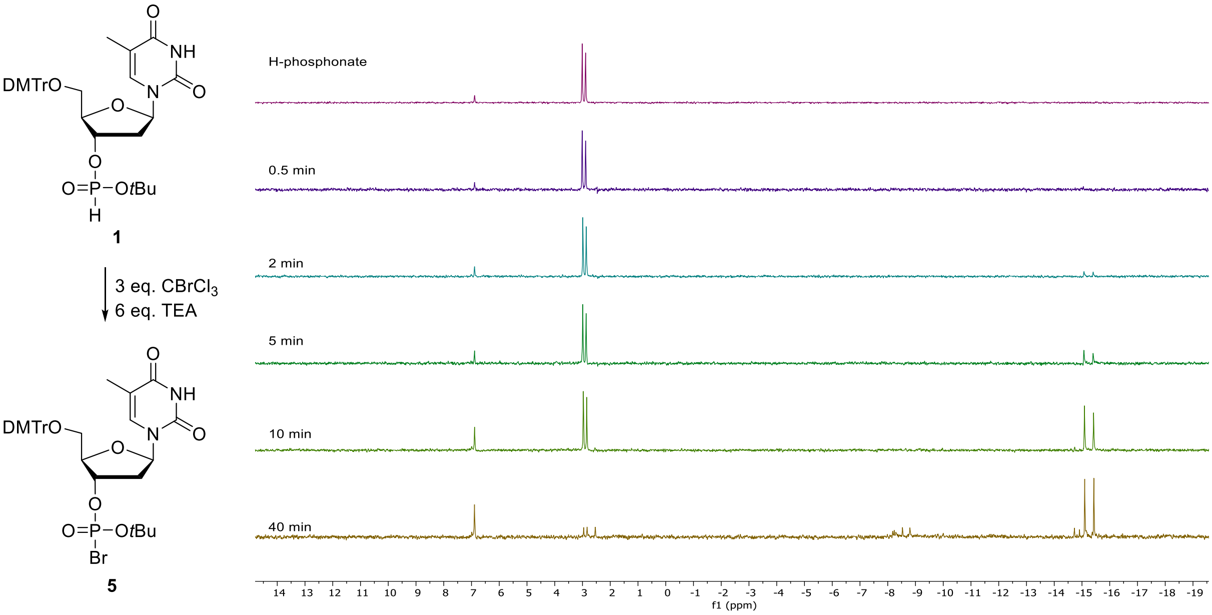

Figure S4. H-phosphonate activation of *t*Bu protected H-phosphonate **1** measured over time, followed by ^{31}P-NMR. For the oxidation reaction, 2 eq. of $CBrCl_3$ as halogenating agent and 3 eq. of TEA as base were used. H-phosphonate **1** (3 ppm), phosphor bromidate **5** (-15 ppm).

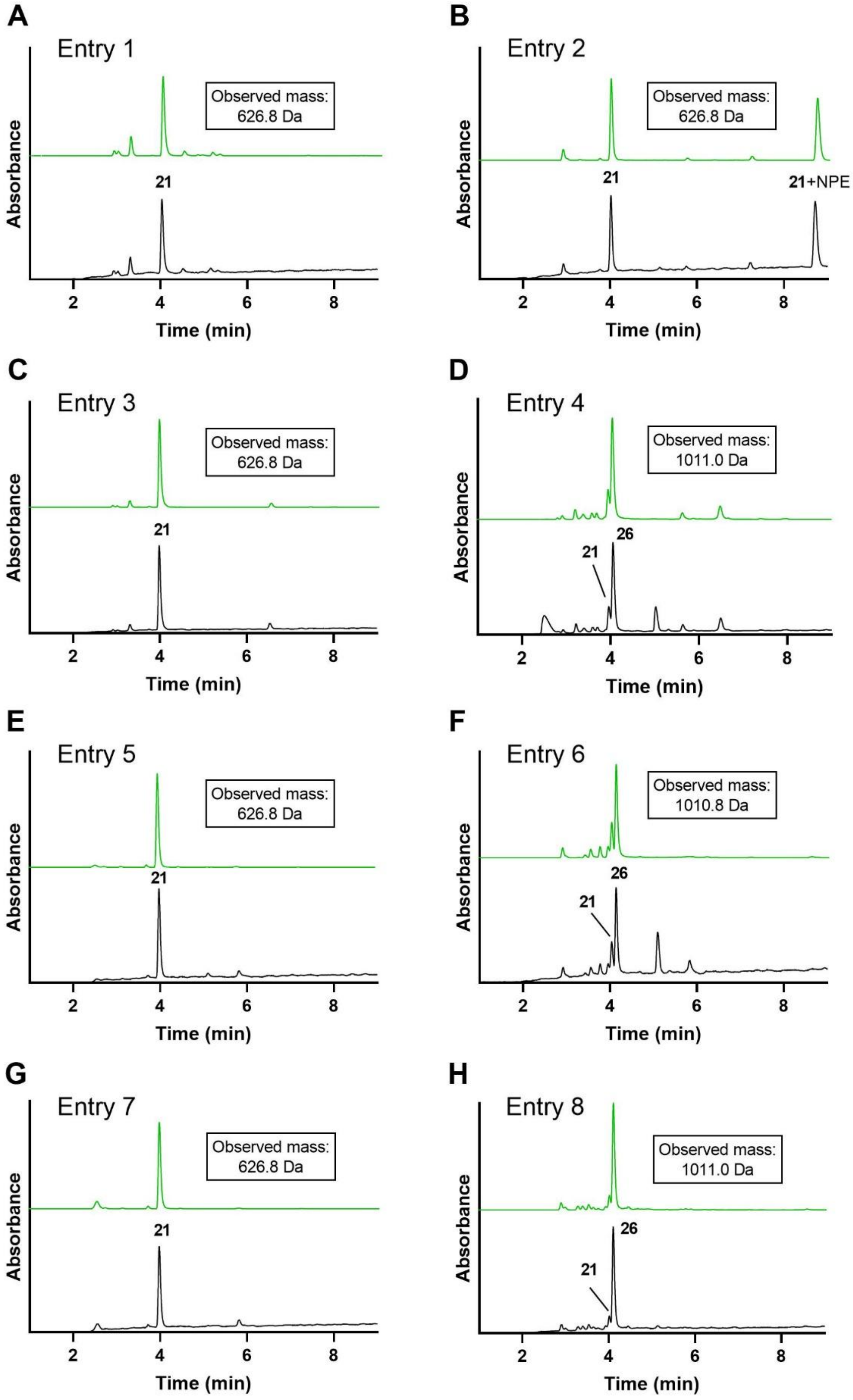
A
Entry 1
Observed mass: 626.8 Da
21
B
Entry 2
Observed mass: 626.8 Da
21
21+NPE
C
Entry 3
Observed mass: 626.8 Da
21
D
Entry 4
Observed mass: 1011.0 Da
21
26
E
Entry 5
Observed mass: 626.8 Da
21
F
Entry 6
Observed mass: 1010.8 Da
21
26
G
Entry 7
Observed mass: 626.8 Da
21
H
Entry 8
Observed mass: 1011.0 Da
21
26
Absorbance
Time (min)
2
4
6
8

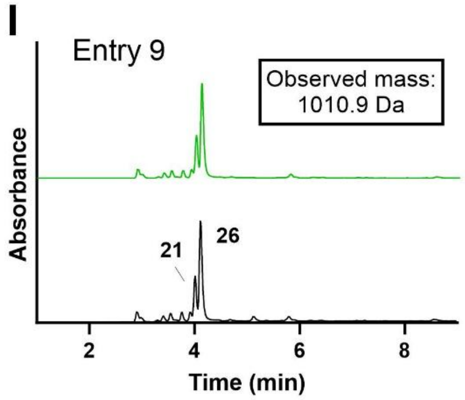

Figure S3. Analytical LCMS (linear gradient 0-20% ACN over 10 minutes) of crude reaction mixtures after the exploratory solid-phase syntheses in **Table 1**. Total scan chromatogram of UV absorption from 200-600 nm is given in black. UV absorption at 268 nm is given in green. Expected mass of dimer **21**: 626.1 Da and expected mass of trimer **26**: 1010.1 Da.

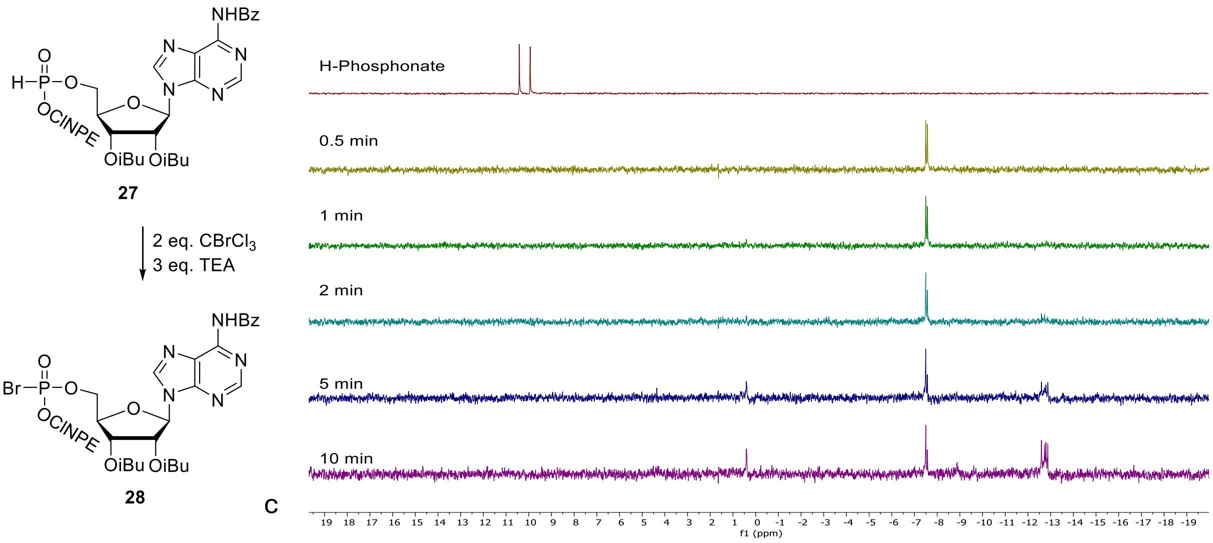

Figure S6. H-phosphonate activation of compound **27** by $CBrCl_3$ and TEA, measured over time by ^{31}P-NMR. General chemical shifts: H-phosphonate **27** (10 ppm), phosphobromidate **28** (-7 ppm), symmetric pyrophosphotetraester of type **VII** (-13 ppm).

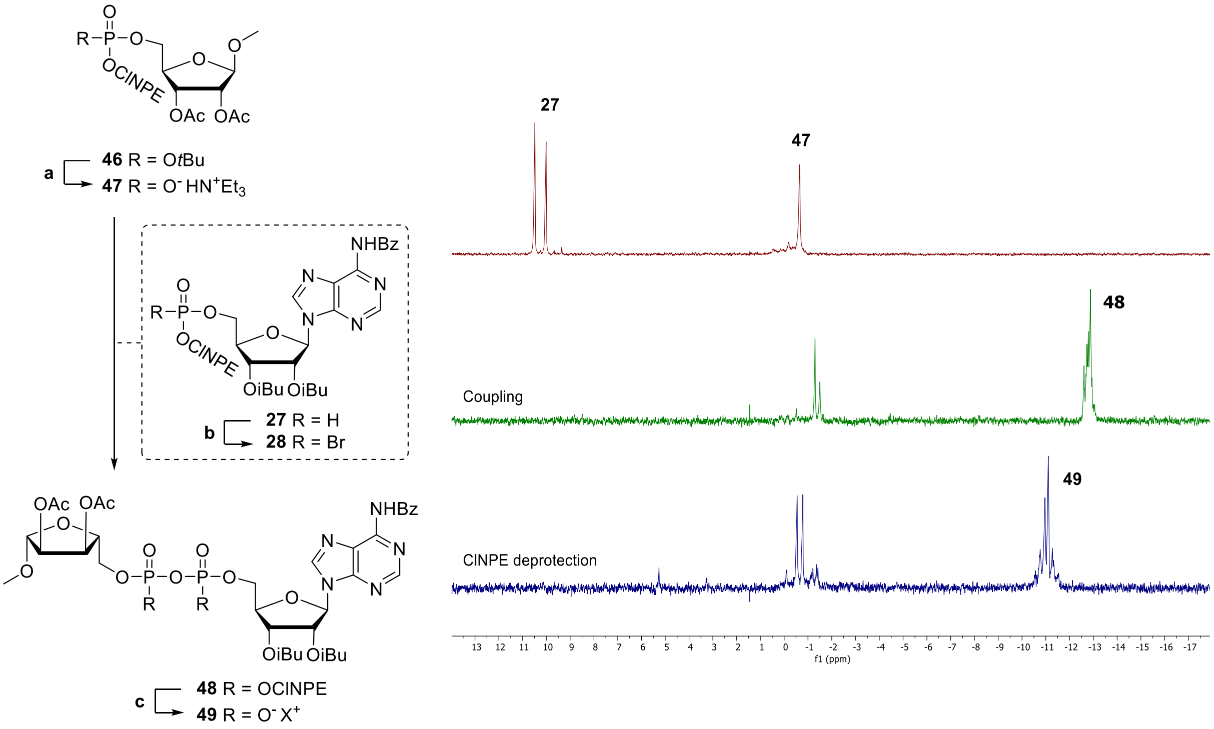

Figure S7. Synthesis of protected ADP-ribose **49** in solution via the P(V)-P(V) methodology. The reaction was analyzed by ^{31}P-NMR. Doublet at 10 ppm corresponds to H-phosphonate **27**. Peaks found at -1 ppm correspond to phosphodiesters. The peak found at -11 ppm corresponds to the pyrophosphate of mono-ADPr **49**. The peak found at -13 ppm corresponds to the pyrophosphotetraester intermediate **48**.

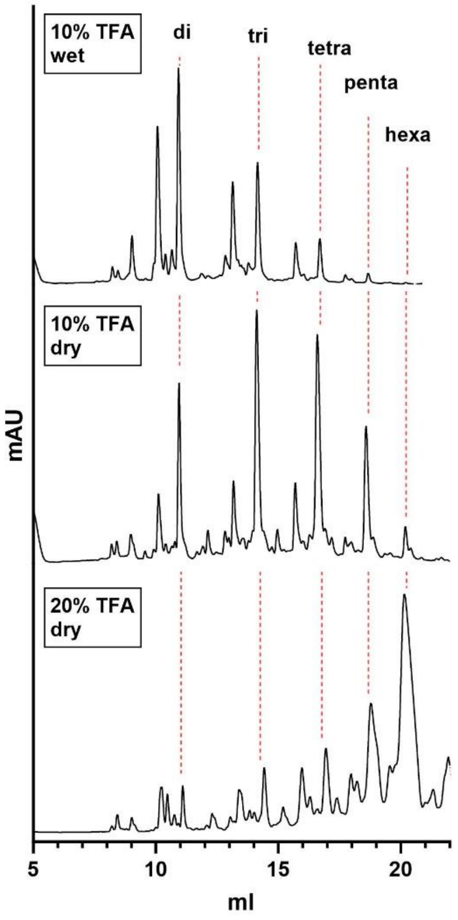

Figure S8. Analytical anion exchange chromatograms of crude syntheses of ADPr- hexamer **44**, obtained via the solid-phase synthetic strategy depicted in **Scheme 2**. The syntheses differ in the rigor of reagent drying prior to solid phase synthesis (top vs. middle/bottom) and the amount of TFA used during *t*Bu deprotection (top/middle vs. bottom). Truncation fragments are indicated with red lines. UV absorbance is measured at 260 nm.

Experimental Section

General synthetic procedures

All reagents were of commercial grade and used as received unless stated otherwise. ^{1}H- and ^{13}C-NMR spectra were recorded on a Bruker AV-400, AV-500 or AV-600 NMR. Chemical shifts (δ) are given in ppm relative to tetramethylsilane as internal standard. Coupling constants (J) are given in Hz. For pyrophosphate-containing compounds, a small amount of EDTA was added to the NMR sample to sharpen the peaks for ^{31}P-NMR. All given ^{13}C-APT spectra are proton decoupled and are presented with even signals (Cq. and CH_2) pointing upwards and odd signals (CH and CH3) pointing downwards. LC-MS analysis was performed on a Finnigan Surveyor HPLC system with a Nucleodur C18 Gravity 3 µm 50 x 4.60 mm column (detection at 200-600 nm) coupled to a Finnigan LCQ Advantage Max mass spectrometer with ESI or a Thermo Scientific Vanquish UHPLC coupled to a Thermo Scientific LCQ Fleet ion mass spectrometer with ESI. Buffers used were A= H_2O, B= MeCN and C= 1% TFA/H_2O. The methods used were 00→20% 13.5 min (0→0.5 min: 00% MeCN; 0.5→8.5 min: 00% to 20% MeCN; 8.5→11 min: 90% MeCN; 11→13.5 min: 00% MeCN) or 0→50% 13.5 min. HRMS was recorded on a Thermo Scientific Q Exactive HF Orbitrap mass

spectrometer equipped with an electrospray ion source. Ion exchange chromatography was performed on a Cytiva ÄKTA pure™. The buffer systems used were Buffer D=10 mM NaOAc, 10 mM NaCl. Buffer E=10 mM NaOAc, 0.5M NaCl. Analytical anion exchange chromatography spectra were measured using a DNAPac PA-100 column 4·250 mm. UV absorbance was measured at 260nm. Preparative Ion exchange chromatography was performed using a Resource Q 6 ml semi-preparative column, using buffers A and B. Fractions were collected based on UV absorbance at 260nm. Size exclusion chromatography was performed on a Cytiva ÄKTA explorer™. The column was prepared using HW-40 media from Toyopearl®. An isocratic elution with a buffer was used containing 0.15M NH_4OAc in 1:9 ACN:H_2O. Fractions were collected based on UV absorbance at 260 nm.

Building block synthesis

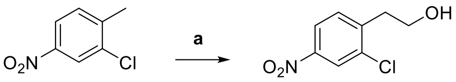

50

Scheme S1. Synthesis of 2-(2-chloro-4-nitrophenyl)ethanol required for the preparation of the ClNPE protecting group. Reagents and conditions: a) para-formaldehyde, DBU, DMF, 90°C, 1 h, 18% (**51**).

2-(2-chloro-4-nitrophenyl)ethan-1-ol (51)

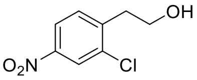

2-chloro-1-methyl-4-nitrobenzene (22.9 g, 134 mmol, 1 eq.) and para-formaldehyde (8.01 g, 267 mmol, 2 eq.) were dissolved in DMF (65 mL, 2 M). DBU (1.0 mL, 6.7 mmol, 0.05 eq.) was added, after which the reaction mixture was heated to 90 °C. After 1 hour, the reaction was quenched with 1 M HCl, and extracted twice with Et_2O, the combined organics were washed once with 1 M HCl, once with brine, dried with $MgSO_4$, filtered and concentrated *in vacuo.* Purification by flash column chromatography (0% -> 5% acetone/DCM) yielded the titled compound as a yellow solid (4.73 g, 23.5 mmol, 18%). **Rf** 0.37 (5% acetone/DCM) **^{1}H-NMR** (400 MHz, $CDCl_3$) δ 8.25 (d, *J* = 2.4 Hz, 1H, arom.), 8.08 (dd, *J* = 8.4, 2.4 Hz, 1H, arom.), 7.50 (d, *J* = 8.4 Hz, 1H, arom.), 3.95 (p, *J* = 3.2 Hz, 2H, CH_2), 3.11 (t, *J* = 6.4 Hz, 2H, CH_2), 1.71 - 1.62 (m, 1H, OH). **^{13}C-NMR** (101 MHz, $CDCl_3$) δ 147.15 (Cq. Arom.), 144.30, 135.18, 131.93 (arom.), 124.79, 121.79, 61.28 (CH_2), 36.87 (CH_2).

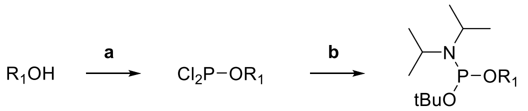

51 R_1= CNE
52 R_1= ClNPE
53 R_1= NPE

54 R_1= CNE
34 R_1= ClNPE
55 R_1= NPE

Scheme S2. Synthesis of asymmetric phosphoramidites **54**, **34** and **55**.

General procedure A: synthesis of phosphordichloridites.

PCl_3 (7 eq.) in ACN (11 M) was added to a flame dried flask under a nitrogen atmosphere. A solution of alcohol (1 eq.) in ACN (2.5 M) was added dropwise over 5 minutes so the final concentration of alcohol was 1M. The solution was stirred for 10 minutes at room temperature, after which it was concentrated *in vacuo.*[40]

2-cyanoethyl phosphordichloridite (51)

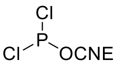

General procedure **A** using 3-hydroxypropionitrile (3.42 mL, 50 mmol, 1 eq.) in ACN (20 mL, 2.5 M) and PCl_3 (30 mL, 350 mmol, 7 eq.) in ACN (16 mL, 11 M) afforded the titled compound as a yellow liquid (8.6 g, 50 mmol, quant.) after concentration *in vacuo*. **^{1}H-NMR** (300 MHz, $CDCl_3$) δ 4.42 (dt, J = 7.7, 6.2 Hz, 2H, CH_2), 2.81 (td, J = 6.2, 0.7 Hz, 2H, CH_2). **^{31}P-NMR** (121 MHz, $CDCl_3$) δ 179.50.

2-(2-chloro-4-nitro-phenyl)ethyl phosphordichloridite (**52**)

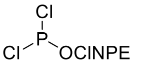

General procedure **A** using 2-(2-chloro-4-nitrophenyl)ethanol (2.02 g, 10 mmol, 1 eq.) in ACN (4 mL, 2.5 M) and PCl_3 (6 mL, 70 mmol, 7 eq.) in ACN (3.2 mL, 11 M) afforded the titled compound as a yellow liquid (3.02 g, 10 mmol, quant.) after concentration *in vacuo*. ^{1}**H-NMR** (400 MHz, $CDCl_3$) δ 8.27 (m, 1H, arom.), 8.09 (m, 1H, arom.), 7.48 (m, 1H, arom.), 4.51 (dtd, *J* = 8.0, 6.4, 1.5 Hz, 1.5H, CH_2), 4.34 (dtd, *J* = 8.0, 6.4, 1.4 Hz, 0.5H, CH_2), 3.40 - 3.17 (m, 2H, CH_2). ^{13}**C-NMR** (101 MHz, $CDCl_3$) δ 147.58 (Cq. Arom.), 142.26, 142.21, 135.29 (arom.), 132.20, 132.10, 132.00, 124.93, 124.89, 122.17, 121.97, 121.95, 65.95 (CH_2), 65.85, 63.35 (CH_2), 63.30, 34.71 (CH_2), 34.64, 34.25, 34.23. ^{31}**P-NMR** (162 MHz, $CDCl_3$) δ 179.21, 9.78 (H-phos.).

2-(4-nitro-phenyl)ethyl phosphordichloridite (**53**)

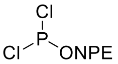

General procedure **A** using 2-(4-nitrophenyl)ethanol (1.67 g, 10 mmol, 1 eq.) in ACN (4 mL, 2.5 M) and PCl_3 (6 mL, 70 mmol, 7 eq.) in ACN (3.2 mL, 11 M) afforded the titled compound as an orange liquid (2.66 g, 9.9 mmol, 99%) after concentration *in vacuo*. ^{1}**H-NMR** (300 MHz, $CDCl_3$) δ 8.25-8.11 (m, 2H, arom.), 7.49 - 7.33 (m, 2H, arom.), 4.61 - 4.14 (m, 2H, CH_2), 3.29 - 3.01 (m, 2H, CH_2). ^{13}**C-NMR** (75 MHz, $CDCl_3$) δ 147.23 (Cq. arom.), 144.76, 129.99 (arom.), 123.90, 67.54 (CH_2), 67.40, 36.11 (CH_2), 36.07. ^{31}**P-NMR** (121 MHz, $CDCl_3$) δ 178.89, 8.28 (H-Phos).

General procedure B: synthesis of asymmetric phosphoramidites.

Phosphordichloridite Cl_2POR_1 (1 eq.) was added to a flame dried flask under a nitrogen atmosphere, after which it was dissolved in THF (1.3 M) and cooled to 0°C. DIPA (2.2 eq.) was added dropwise after which the solution was allowed to heat up to room temperature and was stirred for 30 minutes. A solution of *t*BuOH (1 eq.), DIPEA (2.4 eq.) in THF (5 M) was added dropwise and the solution was stirred overnight. The reaction was quenched with sat. aq. $NaHCO_3$ (1x) and extracted with EtOAc (2x). The combined organics were dried over $MgSO_4$, filtered and concentrated *in vacuo.*

***N,N*-diisopropylamino-*O*-*tert*-butyl-*O*-cyanoethyl phosphoramidite** (**54**)

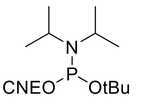

General procedure **B** was performed using 2-cyanoethyl phosphordichloridite **51** (3.78 g, 22 mmol, 1.1 eq.), DIPA (6.17 mL, 44 mmol, 2.2 eq.), *t*BuOH (5.21 mL, 20 mmol, 1.0 eq.) and DIPEA (8.36 mL, 48 mmol, 2.4 eq.) in THF (20 mL, 1.3 M). Purification by flash column chromatography (0% -> 5% Et_2O/Pen +1% TEA) afforded the titled compound as a yellow oil (4.71 g, 17.2 mmol, 86%). ^{1}**H-NMR** (300 MHz, $CDCl_3$) δ 3.86 – 3.67 (m, 2H), 3.67 – 3.52 (m, 2H), 2.63 (td, *J* = 6.6, 0.8 Hz, 2H), 1.36 (s, 9H), 1.17 (dd, *J* = 6.8, 3.0 Hz, 12H). ^{13}**C-NMR** (75 MHz, $CDCl_3$) δ 168.8, 118.0, 58.0, 57.8, 43.4, 43.2, 31.0, 30.9, 24.8, 24.7, 24.3, 24.2, 20.6, 20.5. ^{31}**P-NMR** (121 MHz, $CDCl_3$) δ 139.21.

N,N-diisopropylamino-O-tert-butyl-O-2-(2-chloro-4-nitro-phenyl)ethyl phosphoramidite (**34**)

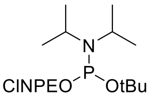

General procedure **B** was performed using 2-(2-chloro-4-nitro-phenyl)ethyl phosphordichloridite **52** (3.02 g, 10 mmol, 1.1 eq.), DIPA (2.8 mL, 20 mmol, 2.2 eq.), *t*BuOH (0.86 mL, 9.1 mmol, 1.0 eq.) and DIPEA (3.8 mL, 22 mmol, 2.4 eq.) in THF (16 mL, 1.3 M). Purification by flash column chromatography (0% -> 5% Et_2O/Pen +1% TEA) afforded the titled compound as a yellow oil (2.79 gr., 7.07 mmol, 78%).^{1}**H-NMR** (400 MHz, $CDCl_3$) δ 8.26 - 8.21 (m, 1H, arom.), 8.08 - 8.00 (m, 1H, arom.), 7.55 - 7.46 (m, 1H, arom.), 3.91 - 3.71 (m, 2H, CH_2ClNPE), 3.65 - 3.47 (m, 2H, CH DIPA), 3.13 (t, J = 6.4 Hz, 2H, CH_2ClNPE), 1.31 (s, 9H, CH_3 *t*Bu), 1.04-1.19 (m, 12H, CH_3 DIPA). ^{13}**C-NMR** (101 MHz, $CDCl_3$) δ 147.0 (Cq. arom.), 145.0, 135.1, 132.2 (arom.), 124.6, 121.5, 75.1 (Cq. *t*Bu), 75.0, 61.0 (CH_2 ClNPE), 60.8, 43.2 (CH DIPA), 43.1, 35.7 (CH_2 ClNPE), 35.7, 31.0 (CH_3 *t*Bu), 30.9, 24.7 (CH_3 DIPA), 24.7, 24.3, 24.2. ^{31}**P-NMR** (162 MHz, $CDCl_3$) δ 137.5.

***N,N*-diisopropylamino-*O-tert*-butyl-*O*-2-(4-nitro-phenyl)ethyl phosphoramidite (55)**

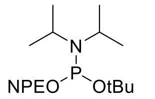

General procedure **B** was performed using 2-(4-nitro-phenyl)ethyl phosphordichloridite **53** (7.37 g, 27.5 mmol, 1.1 eq.), DIPA (7.71 mL, 55 mmol, 2.2 eq.), *t*BuOH (2.37 mL, 25 mmol, 1.0 eq.) and DIPEA (10.5 mL, 60 mmol, 2.4 eq.) in THF (20 mL, 1.3 M). Purification by flash column chromatography (0% -> 5% Et_2O/Pen +1% TEA) afforded the titled compound as a yellow oil (6.46 g, 23.6 mmol, 77%). **^{1}H-NMR** (400 MHz, $CDCl_3$) δ 8.19 - 8.10 (m, 2H, arom.), 7.44 - 7.36 (m, 2H, arom.), 3.89 - 3.68 (m, 2H , CH_2 NPE), 3.65 - 3.48 (m, 2H, CH DIPA), 3.01 (t, J = 6.5 Hz, 2H, CH_2 NPE), 1.30 (s, 9H, CH_3 *t*Bu), 1.08-1.16 (m, 12H, CH_3, DIPA). **^{13}C-NMR** (101 MHz, $CDCl_3$) δ 147.6 (Cq. arom.), 146.7, 130.1 (arom.), 123.6, 75.1 (Cq. *t*Bu), 62.9 (CH_2 NPE), 62.8, 43.2 (CH DIPA), 43.1, 37.9 (CH_2 NPE), 37.8, 31.0 (CH_3 *t*Bu), 30.9, 24.7 (CH_3 DIPA), 24.7, 24.3, 24.2. **^{31}P-NMR** (162 MHz, $CDCl_3$) δ 137.5.

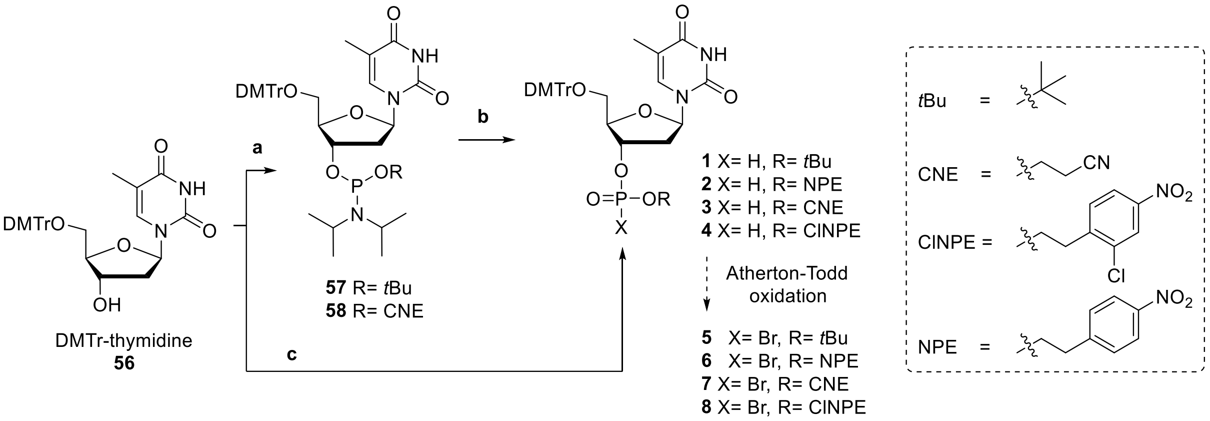

Scheme S3. Synthesis of chain-terminating H-phosphonate building blocks required for solid-phase synthesis of oligo-TDP fragments. Reagents and conditions: a) *t*BuOP(N(*i*Pr)$_2$)$_2$, pyridine·HCl, pyridine, RT, 30 min, 72% (**56**). b) pyridine·HCl, H_2O, ACN, RT, 2 h, 57% (**4**), 93% (**6**). c) (*i*) PCl_2OR (**53** R= NPE for **2**, **52** R= ClNPE for **4**), pyridine, triazole, THF, RT, 30 min, (*ii*) H_2O, RT, 5 min, 98% (**2**), 75% (**4**).

5'-*O*-dimethoxytrityl-3'-*O*-(*N,N*-diisopropylamino-*O-tert*-butyl)-phosphoramidite thymidine (57)

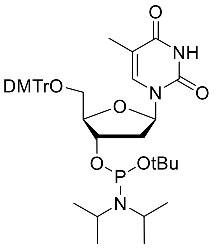

Pyridine·HCl (173 mg, 1.5 mmol, 1.5 eq.) was co-evaporated twice with pyridine under a nitrogen atmosphere. *tert*-butyl tetraisopropylphosphorodiamidite (457 mg, 1.5 mmol, 1.5 eq.) was added to the flask after which it was dissolved in pyridine (10 mL, 0.1 M). DMTr-thymidine **56** (545 mg, 1.0 mmol, 1 eq.) was added to the mixture, after which the reaction was allowed to stir for 20 minutes at room temperature. The reaction mixture was concentrated *in vacuo* and co-evaporated thrice with toluene. The crude mixture was diluted in DCM. washed once with sat. aq. $NaHCO_3$ and extracted twice with DCM. The combined organics were dried over $MgSO_4$, filtered and concentrated *in vacuo*. Purification by flash column chromatography over high purity grade silica (0% -> 5% acetone/DCM + 1% TEA) yielded the titled compound as a white foam (537 mg, 0.72 mmol, 72%, contaminated with H-Phosphonate of *tert*-butyl tetraisopropylphosphorodiamidite). **^{1}H-NMR** (400 MHz, $CDCl_3$) δ 7.82 (s, 0.5H, H-phos), 7.66 (dd, *J* = 11.6, 1.4 Hz, 1H, H-6), 7.44 - 7.38 (m, 2H, arom.), 7.33 - 7.23 (m, 7H, arom.), 6.87 - 6.79 (m, 4H ,arom.), 6.49 - 6.38 (m, 1H, H-1'), 6.28 (s, 0.5H, H-phos), 4.64 - 4.51 (m, 1H, H-3'), 4.22 - 4.14 (m, 1H, H-4'), 3.79 (s, 6H, DMTr OMe), 3.65 - 3.39 (m, 5H, CH DIPA + H-5'a), 3.39 - 3.27 (m, 1H, h-5'b), 2.57 - 2.46 (m, 1H, H-2'a), 2.29 (dt, *J* = 13.6, 7.1 Hz, 1H, H-2'b), 1.50 (s, 9H, CH_3 *t*Bu), 1.34 (s, 3H, CH_3T), 1.26 - 1.20 (m, 18H, CH_3 DIPA), 1.17 - 1.12 (m, 9H, CH_3 DIPA (H-Phos)).**^{13}C-NMR** (101 MHz, $CDCl_3$) δ 164.1 (C-4), 158.8 (Cq. arom.), 150.4 (C-2), 144.4 (Cq. arom.), 144.4, 135.9 (C-6), 135.9, 135.5 (Cq. arom), 135.4, 130.3, 130.2 (arom.), 130.2, 130.2, 128.3 (arom.), 128.3, 128.0 (arom.),

127.2 (arom.), 113.3 (arom.), 113.3, 111.2 (C-5), 111.1, 86.9 (Cq. *t*Bu), 86.0 (C-4'), 85.9, 85.7, 85.6, 84.9 (C-1'), 84.9, 73.1 (C-3'), 73.0, 72.6, 72.5, 63.6 (C-5'), 63.2, 55.3 (DMTr OMe), 45.2 (CH DIPA (H-Phos)), 45.2, 43.3 (CH DIPA), 43.2, 43.1, 43.1, 40.5 (C-2'), 40.4, 40.3, 40.2, 30.5 (CH_3 *t*Bu), 30.5, 24.7 (CH_3 DIPA + (H-Phos)), 24.7, 24.6, 24.6, 24.3, 24.2, 24.2, 24.1, 23.8, 23.7, 23.1, 23.1, 22.8, 22.7, 22.7, 22.6, 22.6, 11.8 (CH_3 T), 11.8. **^{31}P-NMR** (162 MHz, $CDCl_3$) δ 138.9 (amidite), 138.0, 7.0 (H-Phos), 6.2.

5'-*O*-dimethoxytrityl-3'-*O*-*tert*-butyl-*H*-phosphoryl thymidine (1)

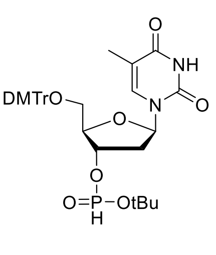

Amidite **56** (374 mg, 0.5 mmol, 1 eq.) was added to a flask under nitrogen atmosphere. pyridine·HCl (116 mg, 1.0 mmol, 2 eq.) was added after which the mixture was dissolved in ACN (5 mL, 0.1 M). Water (45 µL, 2.5 mmol, 5 eq.) was added after which the reaction was stirred for 20 minutes at room temperature. The reaction mixture was diluted with DCM, was with sat. aq. $NaHCO_3$ and extracted once with DCM. The combined organics were dried over $MgSO_4$, filtered and concentrated *in vacuo*. Purification by flash column chromatography over high purity grade silica (0% -> 30% acetone/DCM) yielded the titled compound as a white foam (188 mg, 0.28 mmol, 57%). **^{1}H-NMR** (400 MHz, $CDCl_3$) δ 9.81 (d, J = 3.0 Hz, 1H, H-3), 7.62 - 7.56 (m, 1H,H-6), 7.42 - 7.35 (m, 2H, arom.), 7.34 - 7.15 (m, 8H, arom.), 6.91 (d, $^{1}J_{PH}$ = 700 Hz, 0.5 H, H-Phos diastereoisomer a), 6.88 (d, $^{1}J_{PH}$ = 696 Hz, 0.5 H, H-Phos diastereoisomer b) , 6.88 - 6.78 (m, 4H, arom.), 6.54 - 6.45 (m, 1H, H-1'), 5.30 - 5.15 (m, 1H, H-3'), 4.28 (dt, J = 8.1, 2.4 Hz, 1H, H-4'), 3.78 (s, 6H, DMTr), 3.63 - 3.48 (m, 1H, H-5'a), 3.45 - 3.35 (m, 1H, H-5'b), 2.69 - 2.54 (m, 1H, H-2'a), 2.51 - 2.36 (m, 1H, H-2'b), 1.49 (d, J = 18.6 Hz, 9H, CH_3 *t*Bu), 1.40 (dd, J = 6.3, 1.2 Hz, 3H, CH_3 T). **^{13}C-NMR** (101 MHz, $CDCl_3$) δ 164.1 (C-4), 164.0, 158.8 (Cq. arom.), 150.7 (C-2), 150.6, 144.1 (Cq. arom.), 135.3 (C-6), 135.3, 135.1 (Cq. arom.), 135.0, 130.1 (arom.), 130.1, 128.1, 128.0, 127.2, 113.3 (arom.), 111.6 (C-5), 111.8, 87.2 (Cq. arom.), 84.9 (C-4'), 84.8, 84.7, 84.6, 84.3 (C-1'), 76.2 (C-3'), 76.2, 76.1, 76.0, 63.1 (C-5'), 63.1, 62.2 (Cq. *t*Bu), 55.2 (DMTr), 39.4 (C-2'), 39.4, 39.3, 39.2, 30.4 (CH_3 *t*Bu), 30.3, 30.3, 11.7 (CH_3 T), 11.7. **^{31}P-NMR** (162 MHz, $CDCl_3$) δ 7.6 (reagent), 2.3, 2.2. **HRMS** [$C_{35}H_{41}N_2O_9P$+ Na^+] found: 687.2448, calculated: 687.2442.

5'-*O*-dimethoxytrityl-3'-*O*-2-(4-nitro-phenyl)ethyl)-*H*-phosphoryl thymidine (2)

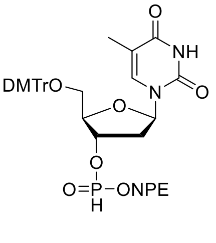

Dichlorophosphite **53** (PCl_2ONPE, 268 mg, 1 mmol, 2 eq.) was dissolved in THF (0.6 mL) under nitrogen atmosphere and cooled to 0 °C. A solution of DMTr-thymidine **56** (273 mg, 0.5 mmol, 1 eq.), 1,2,4-triazole (103 mg, 1.5 mmol, 3 eq.) and pyridine (0.24 mL, 3.0 mmol, 6 eq.) in THF (0.9 mL, final concentration 0.3 M) was added to the cooled dichlorophosphite dropwise over 10 minutes. The reaction was allowed to react room temperature and was stirred for 30 minutes, after which the solution was cooled to 0 °C and water (0.18 mL, 10 mmol, 20 eq.) was added. The reaction was stirred for 5 minutes after which it was diluted with H_2O and extracted twice with DCM. The combined organics were dried over $MgSO_4$, filtered and concentrated *in vacuo*. Purification by flash column chromatography (0% -> 100% acetone/DCM) yielded the titled compound as a white foam (370 mg, 0.49 mmol, 98%). **^{1}H-NMR** (400 MHz, $CDCl_3$) δ 10.08 (dd, J = 11.0, 4.7 Hz, 1H, H-3), 8.26 - 8.04 (m, 2H, arom.), 7.60 - 7.51 (m, 1H, H-6), 7.51 - 7.32 (m, 4H, arom.), 7.30 - 7.16 (m, 7H, arom.), 6.88 - 6.77 (m, 4H, arom.), 6.83 (d, $^{1}J_{PH}$ = 712 Hz, 0.5 H, H-Phos diastereoisomer a), 6.79 (d, $^{1}J_{PH}$ = 712 Hz, 0.5 H, H-Phos diastereoisomer b), 6.45 (ddd, J = 8.8, 5.5, 1.5 Hz, 1H, 1'), 5.31 - 5.17 (m, 1H, H-3'), 4.46 - 4.16 (m, 3H, H-4'+CH_2 NPE), 3.81 - 3.71 (m, 6H, DMTr), 3.50 (ddd, J = 10.6, 9.0, 3.0 Hz, 1H, H-5'a), 3.38 (dd, J = 10.8, 2.6 Hz, 1H, H-5'b), 3.11 (t, J = 6.5 Hz, 1H, CH_2a NPE), 3.04 (t, J = 6.6 Hz, 1H, CH_2b NPE), 2.60 - 2.48 (m, 1H, H-2'a), 2.49 - 2.35 (m, 1H, H-2'b), 1.45 - 1.40 (m, 3H, CH_3 T). **^{13}C-NMR** (101 MHz, $CDCl_3$) δ 164.0 (C-4), 158.7 (Cq. arom.), 158.7, 150.7 (C-2), 150.7, 146.9 (Cq. arom.), 144.6, 144.5, 144.0, 144.0, 135.1 (C-6), 135.0, 135.0 (Cq. arom.), 134.9, 130.0 (arom.), 130.0, 129.9, 129.8, 129.8, 128.0, 128.0, 127.2, 123.7, 123.7, 123.6, 113.3 (arom.), 111.7 (C-5), 87.2 (Cq. DMTr), 87.2, 84.7 (C-4'), 84.6, 84.4, 84.4, 84.2 (C-1'), 84.1, 76.8 (C-3'), 76.9, 76.8, 76.8, 65.4 (CH_2 NPE), 65.4, 65.3, 63.1 (C-

5'), 63.0, 55.2 (DMTr), 55.2, 39.3 (C-2'), 39.0, 39.0, 36.4 (CH_2 NPE), 36.4, 11.7 (CH_3 T), 11.7. **^{31}P-NMR** (162 MHz, $CDCl_3$) δ 7.2, 7.2. **HRMS** [$C_{39}H_{40}N_3O_{11}P$+ Na^+] found: 780.2297, calculated: 780.2293.

5'-*O*-dimethoxytrityl-3'-*O*-2-cyanoethyl-*H*-phosphoryl thymidine (3)

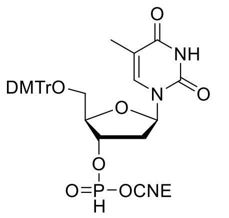

Amidite **58** (370 mg, 0.5 mmol, 1 eq.) was dissolved in DCM:ACN (2:1, 7.5 mL, 0.07 M) under a nitrogen atmosphere. ETT (118 mg, 1 mmol, 2 eq.) was added, followed by H_2O (0.18 mL, 10 mmol, 20 eq.). The reaction was stirred for 30 minutes, after which it was diluted with DCM, washed twice with sat. aq. $NaHCO_3$ and once with brine. The organic fraction was dried over $MgSO_4$, filtered and concentrated *in vacuo*. Purification by flash column chromatography (0% -> 5% MeOH/DCM) yielded the titled compound as a white foam (310 mg, 0.47 mmol, 93%). **Rf** 0.25 (20% acetone/DCM). **^{1}H-NMR** (500 MHz, $CDCl_3$) δ 10.05 (s, 1H, H-3), 7.54 (s, 1H, H-6), 7.39 (d, J = 7.5 Hz, 2H, arom.), 7.33 - 7.22 (m, 7H, arom.), 6.94 (d, $^1J_{PH}$ = 725 Hz, 0.5 H, H-Phos diastereoisomer a), 6.92 (d, $^1J_{PH}$ = 725 Hz, 0.5 H, H-Phos diastereoisomer b), 6.85 (d, J = 8.5 Hz, 4H, arom.), 6.45 (dd, J = 8.6, 5.6 Hz, 1H, H-1'), 5.32 - 5.24 (m, 1H, H-3'), 4.33 - 4.12 (m, 3H, H-4'+ CH_2 CNE), 3.78 (s, 6H, DMTr), 3.54 (dt, J = 11.0, 8.2, 3.0 Hz, 1H, H-5'a), 3.42 (dt, J = 10.9, 3.0 Hz, 1H, H-5'b), 2.76 (t, J = 6.1 Hz, 1H, CH_2a CNE), 2.69 - 2.58 (m, 2H, CH_2b CNE+H-2'a), 2.53 - 2.42 (m, 1H, H-2'b), 1.43 (s, 3H, CH_3 T). **^{13}C-NMR** (126 MHz, $CDCl_3$) δ 164.1 (C-4), 158.8 (Cq arom.), 150.8 (C-2), 150.8, 144.1 (Cq arom.), 135.2 (C-6), 135.2, 135.1 (Cq arom.), 130.2 (arom.), 130.1, 130.1, 128.2, 128.1, 128.1, 127.3, 116.6 (CN), 116.5, 113.4 (arom.), 111.7 (C-5), 87.3 (Cq DMTr), 87.2 (C-4'), 84.6, 84.5, 84.4, 84.3 (C-1'), 77.5 (C-3'), 77.3, 77.0, 76.9, 63.1 (C-5'), 63.0, 60.6 (CH_2 CNE), 60.5, 60.5, 60.4, 55.3 (DMTr), 39.4 (C-2'), 39.1, 19.9 (CH_2 CNE), 19.9, 19.8, 19.8, 11.8 (CH_3 T), 11.8. **^{31}P-NMR** (202 MHz, $CDCl_3$) δ 7.7, 7.7. **HRMS** [$C_{34}H_{36}N_3O_9P$+ Na^+] found: 684.2083, calculated: 684.2081.

5'-*O*-dimethoxytrityl-3'-*O*-2-(2-chloro-4-nitro-phenyl)ethyl-*H*-phosphoryl thymidine (4)

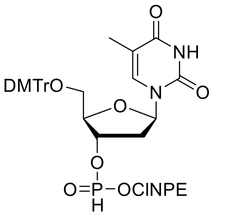

Dichlorophosphite **52** (PCl_2OClNPE, 590 mg, 2 mmol, 2 eq.) was dissolved in THF (2 ml) under nitrogen atmosphere and cooled to 0 °C. A solution of DMTr-thymidine **56** (544 mg, 1.0 mmol, 1.0 eq.), 1,2,4-triazole (210 mg, 3.0 mmol, 3 eq.) and pyridine (0.81 mL, 10 mmol, 10 eq.) in THF (4.0 mL, final concentration 0.15 M) was added to the cooled dichlorophosphite dropwise over 10 minutes. The reaction was reacted at room temperature and was stirred for 30 minutes, after which the solution was cooled to 0°C and water (0.36 mL, 20 mmol, 20 eq.) was added. The reaction was stirred for 15 minutes after which it was diluted with DCM and washed thrice with sat. aq. $NaHCO_3$. The combined organics were dried over $MgSO_4$, filtered and concentrated *in vacuo.* Purification by flash column chromatography (10% -> 50% acetone/DCM) yielded the titled compound as a white foam (588 mg, 0.75 mmol, 75%). **Rf** 0.50 (30% acetone/DCM). **^{1}H-NMR** (400 MHz, $CDCl_3$) δ 10.28 (d, J = 6.2 Hz, 1H, H-3), 8.63 (dt, J = 4.3, 1.8 Hz, 1H, arom.), 8.23 (dd, J = 7.0, 2.4 Hz, 1H, arom.), 8.05 (ddd, J = 15.8, 8.4, 2.4 Hz, 1H, arom.), 7.55 (dd, J = 4.1, 1.5 Hz, 1H, H-6), 7.47 (dd, J = 28.7, 8.4 Hz, 1H, arom.), 7.41 - 7.35 (m, 2H, arom.), 7.33 - 7.22 (m, 8H, arom.), 6.85 (d, $^1J_{PH}$ = 712 Hz, 0.5 H, H-Phos diastereoisomer a), 6.84 (dt, J = 9.2, 2.1 Hz, 4H, arom.), 6.82 (d, $^1J_{PH}$ = 716 Hz, 0.5 H, H-Phos diastereoisomer b), 6.49 - 6.43 (m, 1H, H-1'), 5.30 - 5.20 (m, 1H, H-3'), 4.45 - 4.19 (m, 3H. H-4'+CH_2 ClNPE), 3.79 - 3.74 (m, 6H, DMTr), 3.55 - 3.48 (m, 1H, H-5'a), 3.42 - 3.36 (m, 1H, H-5'b), 3.24 (t, J = 6.5 Hz, 1H, CH_2a ClNPE), 3.17 (t, J = 6.6 Hz, 1H, CH_2b ClNPE), 2.60 - 2.52 (m, 1H, H-2'a), 2.50 - 2.40 (m, 1H, H-2'b), 1.45 - 1.39 (m, 3H, CH_3 T). **^{13}C-NMR** (101 MHz, $CDCl_3$) δ 164.0 (C-4), 158.7 (Cq arom.), 158.7, 150.7 (C-2), 149.4 (arom.), 147.3 (Cq arom.), 144.0, 142.0, 141.9, 136.1 (arom.), 135.0 (C-6), 134.9 (Cq arom.), 132.0 (arom.), 131.9, 130.0, 130.0, 128.0, 128.0, 127.2, 124.6, 123.7, 121.8, 121.8, 113.2 (arom.), 111.6 (C-5), 87.2 (Cq DMTr), 84.7 (C-4'), 84.6, 84.4, 84.3, 84.1 (C-1'), 84.1, 77.0 (C-3'), 77.0, 76.9, 76.9, 63.7 (CH_2 ClNPE), 63.6, 63.6, 63.0 (C-5'), 55.2 (CH_3 DMTr), 39.3 (C-2'), 39.0, 39.0, 34.4 (CH_2 ClNPE),

34.4, 11.7 (CH_3 T), 11.6. ^{31}P NMR (162 MHz, $CDCl_3$) δ 7.77. **HRMS** [$C_{39}H_{39}ClN_3O_{11}P$+ Na^+] found: 814.1905, calculated: 814.1903.

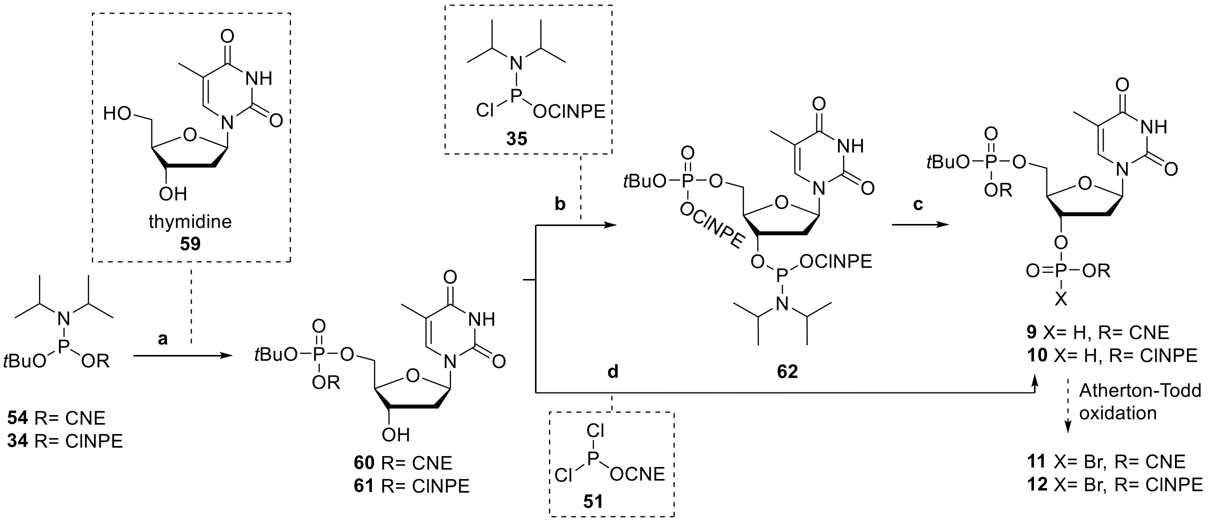

Scheme S4. Synthesis of H-phosphonate building blocks for solid-phase synthesis of oligo-TDP. Reagents and conditions: a) (*i*) thymidine **59**, pyridine·HCl, 3 Å molecular sieves, pyridine, RT, 30 min, (*ii*) *t*BuOOH, RT, 1.5 h, 59% (**60**), 45% (**61**). b) chlorophosphoramidite **35**, NMM, RT, DCM, 15 min, 44% (**62**). c) pyridine·HCl, H_2O, ACN, RT, 2 h, 60% (**10**). d) (*i*) PCl_2OCNE **51**, pyridine, triazole, THF, RT, 30 min, (*ii*) H_2O, RT, 5 min, 57% (**9**).

5'-*O*-(*O*-tert-butyl-*O*-2-cyanoethyl)-phosphoryl thymidine (**60**)

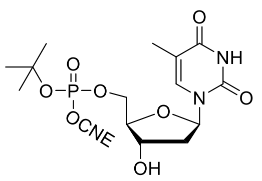

To a solution of thymidine **59** (1.82 g, 7.5 mmol, 1.25 eq.) and pyridine·HCl (1.39 g, 12 mmol, 2 eq.) in pyridine under a nitrogen atmosphere were added 3 Å molecular sieves and the solution was dried overnight. Amidite **54** (1.65 g, 6.0 mmol, 1 eq.) was added to the solution dropwise over 30 minutes and the reaction was allowed to stir for 10 minutes. The reaction mixture was cooled to 0 °C after which *t*BuOOH (5.5 mL, 30 mmol, 5 eq., 5.5 M solution in nonane) was added and the reaction was stirred for 1.5 hours. The reaction was quenched by the addition of sat. aq. $NaHCO_3$ and extracted thrice with DCM. The combined organics were dried over $MgSO_4$, filtered and concentrated *in vacuo.* Purification by flash column chromatography (0% -> 7% MeOH/DCM + 0.5% pyridine) and co-evaporation of the product with dioxane yielded the titled compound as a white foam (1.52 g, 3.5 mmol, 59%). **Rf** 0.24 (5% MeOH/DCM). **^{1}H-NMR** (400 MHz, $CDCl_3$) δ 9.09 (d, J = 19.2 Hz, 1H, H-3), 7.40 (dd, J = 12.9, 1.3 Hz, 1H, H-6), 6.33 (dd, J = 6.4 Hz, 1H, H-1'), 4.53 (dt, J = 7.8, 4.0 Hz, 1H, H-3'), 4.33 - 4.19 (m, 4H, H-5'+CH_2 CNE), 4.12 - 4.06 (m, 1H, H-4'), 2.84 - 2.73 (m, 2H, CH_2 CNE), 2.44 - 2.36 (m, 1H, H-2'a), 2.21 (ddd, J = 13.8, 7.0 Hz, 1H, H-2'b), 1.95 (dd, J = 2.2, 1.2 Hz, 3H, CH_3 T), 1.54 (s, 9H, CH_3 *t*Bu). **^{13}C-NMR** (101 MHz, $CDCl_3$) δ 163.9 (C-4), 150.6 (C-2), 135.8 (C-6), 135.7, 117.0 (CN), 117.0, 111.5 (C-5), 85.6 (Cq tBu), 85.6, 85.5, 85.0 (C-1'), 84.9, 84.8 (C-4'), 84.7, 84.7, 84.6, 71.0 (C-3'), 70.9, 66.9 (C-5'), 66.9, 66.8, 61.9 (CH_2 CNE), 61.8, 40.1 (C-2'), 40.1, 30.0 (CH_3 tBu), 30.0, 19.9 (CH_2 CNE), 19.9, 12.7 (CH_3 T). **^{31}P-NMR** (162 MHz, $CDCl_3$) δ -5.8, -6.3.

5'-*O*-(*O*-tert-butyl-*O*-2-(2-chloro-4-nitro-phenyl)ethyl)-phosphoryl thymidine (**61**)

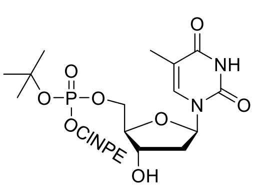

To a solution of thymidine **59** (0.38 g, 1.6 mmol, 1.25 eq.) and pyridine·HCl (0.29 g, 2.5 mmol, 2 eq.) in pyridine under a nitrogen atmosphere were added 3 Å molecular sieves and the solution was dried overnight. Amidite **34** (0.51 g, 1.3 mmol, 1 eq.) was added to the solution dropwise over 30 minutes and the reaction was allowed to stir for 10 minutes. The reaction

mixture was cooled to 0°C after which *t*BuOOH (1.2 mL, 6.25 mmol, 5 eq., 5.5 M solution in nonane) was added and the reaction was stirred for 30 minutes. The reaction was quenched by the addition of sat. aq. $NaHCO_3$ and extracted thrice with DCM. The combined organics were dried over $MgSO_4$, filtered and concentrated *in vacuo*. Purification by flash column chromatography (0% -> 7% MeOH/DCM) yielded the titled compound as a white foam (0.32 g, 0.57 mmol, 45%). **Rf** 0.35 (5% MeOH/DCM). **^{1}H-NMR** (400 MHz, $CDCl_3$) δ 10.30 (d, J = 6.4 Hz, 1H, H-3), 8.22 (dd, J = 5.2, 2.4 Hz, 1H, arom.), 8.06 (ddd, J = 8.2, 5.5, 2.4 Hz, 1H, arom.), 7.51 (t, J = 8.7 Hz, 1H, arom.), 7.43 (s, 1H, H-6), 6.37 (q, J = 7.4 Hz, 1H, H-1'), 4.50 - 4.44 (m, 1H, H-3'), 4.36 - 4.28 (m, 2H, CH_2 ClNPE), 4.22 - 4.16 (m, 2H, H-5'), 4.13 - 4.08 (m, 1H, H-4'), 3.23 (q, J = 6.5 Hz, 2H, CH_2 ClNPE), 2.45 - 2.37 (m, 1H, H-2'a), 2.19 - 2.09 (m, 1H, H-2'b), 1.89 (s, 3H, CH_3 T), 1.47 (s, 9H, CH_3 *t*Bu). **^{13}C-NMR** (101 MHz, $CDCl_3$) δ 164.3 (C-4), 150.9 (C-2), 147.3 (Cq arom.), 147.3, 142.5 (Cq arom.), 142.4, 135.7 (C-6), 135.0 (Cq arom.), 132.1 (arom.), 131.9, 124.6 (arom.), 121.8 (arom.), 121.8, 111.3 (C-5), 85.0 (C-4'), 84.9, 84.9 (C-1'), 84.8, 84.7 (Cq tBu), 84.6, 84.6, 84.5, 71.2 (C-3'), 71.0, 66.8 (C-5'), 65.2 (CH_2 ClNPE), 65.2, 65.1, 65.1, 40.1 (C-2'), 40.1, 34.4 (CH_2 ClNPE), 34.4, 34.3, 34.3, 29.8 (CH_3 tBu), 29.7, 12.5 (CH_3 T). **^{31}P-NMR** (162 MHz, $CDCl_3$) δ -5.2, -5.3.

5'-*O*-(*O*-tert-butyl-*O*-2-(2-chloro-4-nitro-phenyl)ethyl)-phosphoryl-3'-*O*-(*N*,*N*-diisopropylamino-*O*-2-(2-chloro-4-nitro-phenyl)ethyl)-phosphoramidite thymidine (**62**)

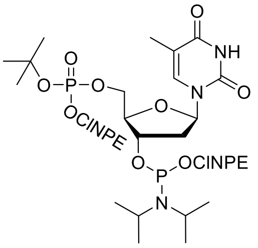

Dichlorophosphite **52** ($PCl_2OClNPE$, 1.2 g, 4.0 mmol, 1.75 eq.) was added to a flame dried flask under a nitrogen atmosphere, after which it was dissolved in DCM (4.4 mL, 0.1 M) and cooled to 0 °C. DIPA (1.1 mL, 8.0 mmol, 2 eq.) was added dropwise after which the reaction was allowed to reach room temperature and stirred for 60 minutes. NMM (0.75 mL, 6.6 mmol, 3 eq.) was added, followed by phosphoryl thymidine **61** (1.24 g, 2.2 mmol, 1 eq.) in DCM (4 mL, final concentration 0.25 M) after which the reaction was stirred for 10 minutes. The reaction was diluted with DCM, washed once with sat. aq. $NaHCO_3$ and once with brine. The combined organics were dried over $MgSO_4$, filtered and concentrated *in vacuo*. Purification by automated column chromatography (0% -> 20% acetone/DCM) yielded the titled compound as a white foam (0.86 g, 0.96 mmol, 44%). **Rf** 0.60 (30% acetone/DCM). **^{1}H-NMR** (300 MHz, $CDCl_3$) δ 9.49 (s, 1H, H-3), 8.27 - 8.20 (m, 2H, arom.), 8.11 - 8.04 (m, 2H, arom.), 7.55 - 7.47 (m, 2H, arom.), 7.45 - 7.41 (m, 1H, H-6), 6.40 - 6.30 (m, 1H, H-1'), 4.52 - 4.41 (m, 1H, H-3'), 4.34 - 4.24 (m, 2H, CH_2 ClNPE), 4.17 - 4.06 (m, 3H, H-4'+H-5'), 3.98 - 3.76 (m, 2H, CH_2 ClNPE), 3.62 - 3.46 (m, 2H, CH DIPA), 3.28 - 3.10 (m, 4H, CH_2 ClNPE), 2.49 - 2.31 (m, 1H, H-2'a), 2.14 - 1.99 (m, 1H, H-2'b), 1.94 - 1.89 (m, 3H, CH_3 T), 1.49 - 1.45 (m, 9H, CH_3 *t*Bu), 1.19 - 1.09 (m, 12H, CH_3 DIPA). **^{13}C-NMR** (75 MHz, $CDCl_3$) δ 163.9 (C-4), 150.5 (C-2), 147.4 (Cq arom.), 147.1, 144.1 (Cq arom.), 144.1, 142.4 (Cq arom.), 135.4 (C-6), 135.1 (Cq arom.), 135.0, 132.1 (arom.), 132.0, 131.9, 124.8 (arom.), 124.6, 124.6, 121.8 (arom.), 121.6, 111.5 (C-5), 111.5, 111.4, 84.9 (C-1'), 84.9, 84.4 (Cq tBu), 84.1 (C-4'), 73.0 (C-3'), 72.9, 72.7, 66.6 (C-5'), 66.5, 66.4, 65.1 (CH_2 ClNPE), 65.0, 65.0, 61.7 (CH_2 ClNPE), 61.7, 61.6, 61.5, 61.4, 43.2 (CH DIPA), 43.1, 39.4 (C-2'), 35.5 (CH_2 ClNPE), 35.4, 34.5 (CH_2 ClNPE), 29.9 (CH_3 tBu), 29.8, 24.6 (CH_3 DIPA), 24.5, 12.5 (CH_3 T). **^{31}P-NM**R (121 MHz, $CDCl_3$) δ 149.6, 149.5, 149.5, 149.4, 149.4, -4.1, -4.2, -4.3, -4.3.

5'-*O*-(*O*-tert-butyl-*O*-2-cyanoethyl)-phosphoryl-3'-*O*-2-cyanoethyl-*H*-phosphoryl thymidine (**9**)

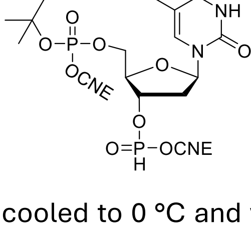

Dichlorophosphite **51** (PCl_2OCNE, 770 mg, 4.5 mmol, 3 eq.) was dissolved in THF (1 mL) under nitrogen atmosphere and cooled to 0 °C. A solution of phosphoryl thymidine **15** (650 mg, 1.5 mmol, 1.0 eq.), 1,2,4-triazole (310 mg, 4.5 mmol, 3 eq.) and pyridine (1.2 mL, 15 mmol, 10 eq.) in THF (2.0 mL, final concentration 1.0 M) was added to the cooled dichlorophosphite dropwise over 10 minutes. The reaction was allowed to react room temperature and was stirred for 30 minutes, after which the solution was cooled to 0 °C and water (0.54 mL, 30 mmol, 20 eq.) was added. The reaction was stirred for 5

minutes after which it was diluted with DCM and washed thrice with sat. aq. $NaHCO_3$. The combined organics were dried over $MgSO_4$, filtered and concentrated *in vacuo*. Purification by flash column chromatography (0% -> 100% acetone/DCM + 0.5% pyridine) yielded the titled compound as a white foam (470 mg, 0.85 mmol, 57%). **Rf** 0.58 (80% acetone/DCM). **^{1}H-NMR** (400 MHz, $CDCl_3$) δ 9.83 (d, J = 13.9 Hz, 1H, H-3), 7.40 (dd, J = 19.7, 3.4 Hz, 1H, H-6), 7.03 (d, $^1J_{PH}$ = 724 Hz, 0.5 H, H-Phos diastereoisomer a), 7.01 (d, $^1J_{PH}$ = 728 Hz, 0.5 H, H-Phos diastereoisomer b), 6.45 - 6.32 (m, 1H, H-1'), 5.34 - 5.25 (m, 1H, H-3'), 4.43 - 4.18 (m, 7H, H-4'+H-5'+CH_2 CNE), 2.90 - 2.70 (m, 4H, CH_2 CNE), 2.66 - 2.50 (m, 1H, H-2'a), 2.47 - 2.27 (m, 1H, H-2'b), 1.94 (s, 3H, CH_3 T), 1.54 (s, 9H, CH_3 *t*Bu). **^{13}C-NMR** (101 MHz, $CDCl_3$) δ 164.0 (C-4), 150.7 (C-2), 150.7, 135.4 (C-6), 135.3, 117.0 (CN), 116.8, 111.9 (C-5), 111.8, 85.6 (Cq tBu), 85.6, 84.8 (C-1'), 84.7, 84.6, 83.3 (C-4'), 83.2, 83.2, 76.1 (C-3'), 76.0, 76.0, 75.9, 75.8, 75.7, 75.6, 66.5 (C-5'), 66.5, 66.4, 66.3, 66.2, 66.1, 61.9 (CH_2 CNE), 61.9, 60.8 (CH_2 CNE), 60.8, 60.8, 60.8, 60.7, 38.6 (C-2'), 38.5, 29.9 (CH_3 *t*Bu), 29.9, 20.1 (CH_2 CNE), 20.0, 19.8 (CH_2 CNE), 19.7, 12.6 (CH_3 T), 12.5.**^{31}P-NMR** (162 MHz, $CDCl_3$) δ 7.4, 7.3, 7.23, -6.1, -6.2, -6.9, -6.9. **HRMS** [$C_{13}H_{18}N_3O_8P$+ Na^+] (**20** without *t*Bu and H-phosphonate) found: 398.0731, calculated: 398.0724.

5'-O-(O-tert-butyl-O-2-(2-chloro-4-nitro-phenyl)ethyl)-phosphoryl-3'-O-2-(2-chloro-4-nitro-phenyl)ethyl-H-phosphoryl thymidine (**10**)

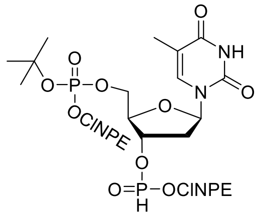

Amidite **62** (860 mg, 0.96 mmol, 1 eq.) was dissolved in DCM (10 mL, 0.1 M) under a nitrogen atmosphere. DCI (110 mg, 0.96 mmol, 1 eq.) was added, followed by H_2O (0.087 mL, 4.8 mmol, 5 eq.). The reaction was stirred for 4 hours, after which it was concentrated *in vacuo* diluted with $CHCl_3$ and washed thrice with sat. aq. $NaHCO_3$. The organic fraction was dried over $MgSO_4$, filtered and concentrated *in vacuo*. Purification by flash column chromatography (0% -> 6% MeOH/DCM) yielded the titled compound as a white foam (470 mg, 0.58 mmol, 60%). **Rf** 0.55 (50% acetone/DCM). **^{1}H-NMR** (400 MHz, $CDCl_3$) δ 9.76 (s, 1H, H-3), 8.30 - 8.20 (m, 2H, arom.), 8.15 - 8.04 (m, 2H, arom.), 7.55 - 7.46 (m, 2H, arom.), 7.42 - 7.36 (m, 1H, H-6), 6.90 (d, $^1J_{PH}$ = 712 Hz, 0.5 H, H-Phos diastereoisomer a), 6.87 (d, $^1J_{PH}$ = 720 Hz, 0.5 H, H-Phos diastereoisomer b), 6.38 - 6.30 (m, 1H, H-1'), 5.17 - 5.06 (m, 1H, H-3'), 4.50 - 4.36 (m, 2H, CH_2 ClNPE), 4.35 - 4.09 (m, 5H, H-4'+H-5'+CH_2 ClNPE), 3.31 - 3.19 (m, 4H, CH2 ClNPE), 2.50 - 2.41 (m, 1H, H-2'a), 2.27 - 2.16 (m, 1H, H-2'b), 1.90 (d, J = 1.4 Hz, 3H, CH_3 T), 1.47 (s, 9H, CH_3 *t*Bu). **^{13}C-NMR** (101 MHz, $CDCl_3$) δ 163.9 (C-4), 150.6 (C-2), 150.6, 147.6 (Cq arom.), 147.4, 142.4 (Cq arom.), 142.0, 135.3 (C-6), 135.2, 135.2 (Cq arom.), 135.1, 132.2 (arom.), 132.1, 132.0, 124.9 (arom.), 124.8, 122.0 (arom.), 122.0, 121.9, 121.9, 111.9 (C-5), 111.8, 111.8, 85.0 (Cq tBu), 84.9, 84.8, 84.8 (C-1'), 84.7, 83.7 (C-4'), 83.4, 83.4, 83.3, 76.3 (C-3'), 76.2, 76.2, 75.8, 75.8, 75.7, 66.3 (C-5'), 66.2, 66.2, 65.3 (CH_2 ClNPE), 65.3, 65.3, 65.2, 64.1 (CH_2 ClNPE), 64.0, 64.0, 64.0, 63.9, 38.9 (C-2'), 38.8, 38.8, 38.6, 38.5, 38.4, 34.6 (CH_2 ClNPE), 34.6, 34.5, 34.5, 34.4, 34.4, 29.9 (CH_3 tBu), 29.8, 12.6 (CH_3 T), 12.5. **^{31}P-NMR** (162 MHz, $CDCl_3$) δ 8.1, 8.1, 7.9, 7.9, 7.9, -5.6, -5.6, -5.7, -5.7. **HRMS** [$C_{18}H_{21}ClN_3O_{10}P$+ H^+] (**21** without *t*Bu and H-phosphonate) found: 506.0737, calculated: 506.0726.

5'-*O*-dimethoxytrityl-3'-*O*-hydroquinone-*O*,*O*'-diacetylhemiester-triethylammonium thymidine (**65**)

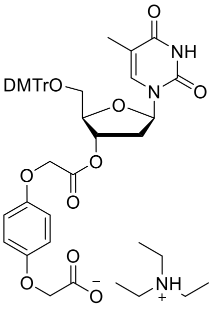

DMTr-thymidine **56** (1.32 g, 2.0 mmol, 1 eq.) was dissolved in pyridine (20 ml. 0.1 M) under a nitrogen atmosphere. To the solution was added DMAP (24 mg, 0.2 mmol, 0.1 eq.), EDC·HCl (420 mg, 2.2 mmol, 1.1 eq.), TEA (0.20 mL, 1.4 mmol, 0.7 eq.) and hydroquinone-*O*,*O*'-diacetic acid (540 mg, 2.4 mmol, 1.2 eq.) and the reaction was stirred overnight. The reaction mixture was concentrated in vacuo, co-evaporated thrice with toluene. Purification by flash column chromatography (0% -> 20% MeOH/DCM + 1% TEA) yielded the titled compound as a yellow foam (700 mg, 0.72 mmol, 36%). **Rf** 0.19 (10% MeOH/DCM). **^{1}H-NMR** (400 MHz, $CDCl_3$) δ 7.60 (d, J = 1.4 Hz, 1H, H-6), 7.40 -

7.36 (m, 2H, arom.), 7.32 - 7.23 (m, 8H, arom.), 6.92 - 6.78 (m, 8H, arom.), 6.43 (t, J = 7.3 Hz, 1H, H-1'), 5.57 (td, J = 3.7, 2.9, 1.7 Hz, 1H, H-3'), 4.58 (d, J = 2.5 Hz, 2H, CH_2 Q-linker), 4.43 (s, 2H, CH_2 Q-linker), 4.14 (q, J = 2.2 Hz, 1H, H-4'), 3.79 (s, 6H, DMTr), 3.53 - 3.43 (m, 2H, H-5'), 2.98 (q, J = 7.2 Hz, 13H, CH_2 TEA), 2.52 - 2.36 (m, 2H, H-2'), 1.37 (d, J = 1.1 Hz, 3H, CH_3 T), 1.27 (t, J = 7.3 Hz, 20H, CH_3 TEA). **^{13}C-NMR** (101 MHz, $CDCl_3$) δ 175.0 (COOH), 169.0 ($COOCH_2$), 163.5 (C-4), 158.9 (Cq arom.), 158.9, 154.2 (Cq arom.), 151.7 (Cq arom.), 150.4 (C-2), 144.3 (Cq arom.), 135.4 (C-6), 135.3 (Cq arom.), 135.2, 130.2 (arom.), 130.2, 128.2 (arom.), 128.2, 127.4 (arom.), 115.8 (arom.), 115.7, 113.5 (arom.), 111.8 (C-5), 87.4 (Cq DMTr), 84.4 (C-1'), 84.1 (C-4'), 76.4 (C-3'), 68.0 (CH_2 Q-linker), 66.3 (CH_2 Q-linker), 63.7 (C-5'), 55.4 (DMTr), 45.5 (CH_2 TEA), 37.9 (C-2'), 11.8 (CH_3 T), 9.2 (CH_3 TEA).

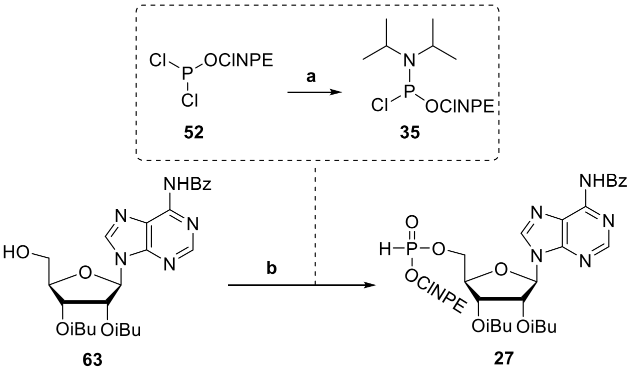

Scheme S5. Synthesis of chain terminating adenosine **4** required for solid-phase synthesis of ADPr oligomers. Reagents and conditions: a) **52**, DIPA, DCM, 0°C, 30 min. b) (*i*) **35**, NMM, DCM, RT, 30 min, (*ii*) pyridine·HCl, H_2O, ACN, RT, 1 h, 45% (**27**).

***N*^{6} -Benzoyl-9-[5'-*O*-2-(2-chloro-4-nitro-phenyl)ethyl-*H*-phosphoryl-2',3'-di-*O*-isobutyryl-β-D-ribofuranosyl]-adenine (27)**

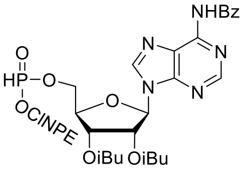

Dichlorophosphite **52** ($PCl_2OClNPE$, 0.44 g, 1.5 mmol, 3.0 eq.) was added to a flame dried flask under a nitrogen atmosphere, after which it was dissolved in DCM (3 mL, 0.5 M) and cooled to 0 °C. DIPA (0.43 mL, 3.1 mmol, 6.3 eq.) was added dropwise after which the reaction was allowed to reach room temperature and stirred for 30 minutes. NMM (0.32 mL, 3.0 mmol, 6.0 eq.) was added, followed by compound **63**[41] (0.25 g, 0.49 mmol, 1.0 eq.) in DCM (2 mL, final concentration 0.1 M) after which the reaction was stirred for 15 minutes. The reaction was diluted with EtOAc and washed once with sat. aq. $NaHCO_3$. The combined organics were dried over $MgSO_4$, filtered and concentrated *in vacuo*. The crude mixture was filtered over a short plug of high purity grade silica (0% -> 5% acetone/DCM). The fractions containing the amidite intermediate were collected and concentrated. The intermediate amidite together with pyridine·HCl (120 mg, 1.0 mmol, 2 eq.) was dissolved in ACN (5 mL, 0.1 M), after which H_2O (45 µL, 2.5 mmol, 5 eq.) was added. The reaction was stirred for 50 minutes, after which it was concentrated *in vacuo*. The crude mixture was diluted with $CHCl_3$ and sat. aq. $NaHCO_3$, extracted twice with $CHCl_3$, dried over $MgSO_4$, filtered and conczentrated *in vacuo*. Purification by flash column chromatography over high purity grade silica (0% -> 20% acetone/DCM) yielded the titled compound as a white foam (170 mg, 0.22 mmol, 45%). **Rf** 0.42 (25% acetone/DCM). **^{1}H-NMR** (400 MHz, $CDCl_3$) δ 9.33 (s, 1H, NH), 8.78 (d, J = 7.2 Hz, 1H, H-2), 8.26 (d, J = 10.0 Hz, 1H, H-8), 8.24 - 8.21 (m, 1H, arom.), 8.10 - 8.00 (m, 3H, arom.), 7.63 - 7.57 (m, 1H, arom.), 7.54 - 7.46 (m, 3H, arom.), 6.90 (d, $^1J_{PH}$ = 710 Hz, 0.5 H, H-Phos diastereoisomer a), 6.89 (d, $^1J_{PH}$ = 715 Hz, 0.5 H, H-Phos diastereoisomer b), 6.28 (t, J = 6.1 Hz, 1H, H-1'), 5.85 (dt, J = 11.7, 5.8 Hz, 1H, H-2'), 5.73 - 5.66 (m, 1H, H-3'), 4.45 - 4.31 (m, 5H, H-4'+CH_2 ClNPE), 3.25 - 3.18 (m, 2H, H-5'), 2.69 - 2.48 (m, 2H, CH *i*Bu), 1.23 (dd, J = 7.0, 0.7 Hz, 6H, CH_3 *i*Bu), 1.13 (dd, J = 14.6, 7.0 Hz, 6H CH_3 *i*Bu). **^{13}C-NMR** (101 MHz, $CDCl_3$) δ 175.9 (COO *i*Bu), 175.5, 175.5, 164.9 (COO Bz), 153.0 (C-2), 151.8 (C-4), 150.0 (C-6), 149.9, 147.5 (Cq arom.),

142.1 (Cq arom.), 142.1, 141.6 (C-8), 141.5, 135.1 (Cq arom.), 133.5 (Cq arom.), 133.5, 132.9 (arom.), 132.1 (arom.), 128.9 (arom.), 128.0 (arom.), 124.8 (arom.), 123.5 (C-5), 123.5, 122.0 (arom.), 122.0, 86.3 (C-1'), 86.2, 81.7 (C-4'), 81.6, 81.6, 81.6, 73.2 (C-2'), 73.0, 70.3 (C-3'), 70.2, 64.6 (CH_2 ClNPE), 64.6, 64.5, 63.9 (CH_2 ClNPE), 63.9, 63.8, 34.6 (C-5'), 34.6, 34.5, 34.5, 33.8 (CH *i*Bu), 33.7, 18.9 (CH_3 *i*Bu), 18.8, 18.7. **^{31}P-NMR** (162 MHz, $CDCl_3$) δ 8.7, 8.3. **HRMS** [$C_{33}H_{36}ClN_6O_{11}P$ + H^+] found: 759.1937, calculated: 759.1941.

N^6 -Benzoyl-9-[2-*O*-(2,3-di-*O*-acetyl-α-D-ribofuranosyl)-3-*O*-acetyl-5-*O*-dimethoxytrityl-β-D-ribofuranosyl]-adenine (32)

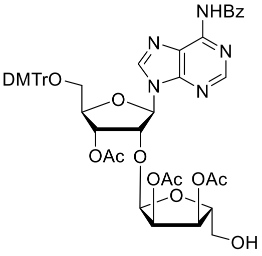

Compound **31** (0.12 g, 0.96 mmol) was co-evaporated with dioxane (2x) and dissolved in THF (10 mL, 0.1 M). TBAF (1.91 mL, 1.9 mmol, 2 eq., 1 M in THF) was added dropwise and the reaction was stirred overnight. After TLC analysis showed full conversion of the starting material, the reaction was quenched with water. The mixture was diluted with EtOAc, and washed with water (2x) and brine, after which the organic layer was dried over $MgSO_4$, filtered and concentrated *in vacuo*. Purification by flash column chromatography (0% -> 3% MeOH/DCM) yielded the titled compound as a white foam (670 mg, 0.72 mmol, 75%). **Rf** 0.52 (30% acetone/DCM). **^{1}H-NMR** (400 MHz, $CDCl_3$) δ 9.17 (s, 1H, NH), 8.74 (s, 1H, H-2), 8.18 (s, 1H, H-8), 8.09 - 7.93 (m, 2H, arom.), 7.63 - 7.57 (m, 1H, arom.), 7.57 - 7.50 (m, 2H ,arom.), 7.47 - 7.37 (m, 2H, arom.), 7.32 - 7.15 (m, 9H, arom.), 6.81 (d, J = 8.9 Hz, 4H, arom.), 6.23 (d, J = 6.0 Hz, 1H, H-1'), 5.54 (dd, J = 5.2, 3.5 Hz, 1H, H-3'), 5.32 (d, J = 4.5 Hz, 1H, H-1"), 5.23 (t, J = 5.6 Hz, 1H, H-2'), 5.18 (dd, J = 7.4, 4.0 Hz, 1H, H-3"), 4.77 (dd, J = 7.4, 4.5 Hz, 1H, H-2"), 4.32 (q, J = 3.6 Hz, 1H, H-4'), 4.12 (q, J = 3.4 Hz, 1H, H-4"), 3.81-3.68 (m, 8H, H-5"+DMTr), 3.55 (dd, J = 10.7, 3.4 Hz, 1H, H-5'a), 3.45 (dd, J = 10.7, 3.9 Hz, 1H, H-5'b), 2.44-2.34 (bs, 1H, OH-5"), 2.14 (s, 3H, CH_3 Ac), 2.10 (s, 3H, CH_3 Ac), 1.83 (s, 3H, CH_3 Ac). **^{13}C-NMR** (101 MHz, $CDCl_3$) δ 170.4 (COO Ac), 169.9 (COO Ac), 169.7 (COO Ac), 164.7 (COO Bz), 158.8 (Cq arom.), 153.0 (C-2), 151.9 (C-4), 149.8 (C-6), 144.4 (Cq arom.), 141.6 (C-8), 135.5 (Cq arom.), 135.5, 133.7 (Cq arom.), 132.9 (arom.), 130.2 (arom.), 130.2, 129.0 (arom.), 128.3 (arom.), 128.1, 128.0, 127.2 (arom.), 123.4 (C-5), 113.4 (arom.), 101.4 (C-1"), 87.0 (Cq DMTr), 86.4 (C-1'), 82.6 (C-4"), 82.6 (C-4'), 78.3 (C-2'), 72.2 (C-3'), 71.3 (C-2"), 69.7 (C-3"), 63.0 (C-5"), 62.1 (C-5'), 55.4 (DMTr), 21.0 (CH_3 Ac), 20.9 (CH_3 Ac), 20.3 (CH_3 Ac). Spectra were in accordance with Kistemaker *et al.*[15]

N^6-Benzoyl-9-[2-*O*-(2,3-di-*O*-acetyl-5-*O*-(*O*-tert-butyl-*O*-2-chloro-4-nitro-phenylethyl)-phosphoryl-α-D-ribofuranosyl)-3-*O*-acetyl-β-D-ribofuranosyl]-adenine (33)

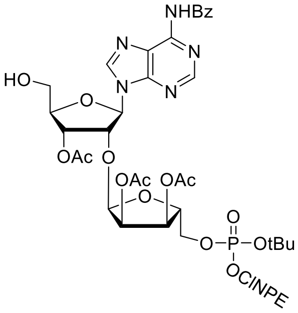

Compound **31** (2.9 g, 3.1 mmol) was co-evaporated (3x) with dioxane after which it was dissolved in 38 mL of a solution of 1-methyl-imidazole·HCl (3 eq., 0.3 M) and 1-methyl-imidazole (2 eq., 0.2 M) in DMF (dried over 3 Å molecular sieves). *Tert*-butyl-2-chloro-4-nitro-phenylethyl-*N*,*N*-diisopropylphosphoramidite **34** (1.4 g, 3.5 mmol, 1.1 eq.) was added and the mixture was stirred for 15 minutes. The solution was cooled to 0 °C after which *t*BuOOH (2.9 mL, 15.7 mmol, 5 eq., 5.5 M solution in decane) was added dropwise and stirred for 1 hour. The reaction was quenched with sat. aq. $NaHCO_3$ and extracted with EtOAc (2x). The combined organics were washed with brine, dried over $MgSO_4$, filtered, concentrated *in vacuo* and co-evaporated with toluene (3x). The crude mixture was dissolved in DCM (23 mL, 0.15 M) after which a solution of TFA (23 mL, 4% v/v in DCM) was added dropwise. The mixture was stirred for 30 minutes, after which TLC analysis showed full conversion of the starting material. The reaction was quenched with sat. aq. $NaHCO_3$ and extracted with DCM (3x). The combined organics were dried over $MgSO_4$, filtered and concentrated *in vacuo*. Purification by flash column chromatography over high purity grade silica (0% -> 10% MeOH/DCM) yielded the titled compound as a white foam (2.7 g, 2.8 mmol, 90%). **Rf** 0.41 (7% MeOH/DCM). **^{1}H-NMR** (400 MHz, $CDCl_3$) δ 9.58 - 9.46 (m, 1H, NH), 8.81 - 8.71 (m, 1H, H-2), 8.29 - 8.22 (m, 1H, arom.), 8.17 (d, J =

1.3 Hz, 1H, H-8), 8.08 - 8.01 (m, 3H, arom.), 7.63 - 7.57 (m, 1H, arom.), 7.54 - 7.46 (m, 3H, arom.), 6.09 - 6.01 (m, 2H, H-1'+OH-5'), 5.71 - 5.63 (m, 1H, H-3'), 5.17 - 5.11 (m, 2H, H-2'+H-3"), 5.05 (t, J = 4.8 Hz, 1H, H-1"), 4.70 - 4.65 (m, 1H, H-2"), 4.33 - 4.30 (m, 1H, H-4'), 4.28 - 4.18 (m, 2H, CH_2 ClNPE), 4.16 - 4.10 (m, 1H, H-4"), 4.07 - 3.63 (m, 4H, H-5'+H-5"), 3.20 - 3.14 (m, 2H, CH_2 ClNPE), 2.20 - 2.17 (m, 3H, CH_3 Ac), 2.16 - 2.11 (m, 3H, CH_3 Ac), 1.98 - 1.93 (m, 3H, CH_3 Ac), 1.40 (s, 9H, CH3 *t*Bu). **^{13}C-NMR** (101 MHz, $CDCl_3$) δ 170.1 (COO Ac), 169.5 (COO Ac), 169.4 (COO Ac), 164.8 (COO Bz), 152.2 (C-2), 150.6 (C-4), 150.4 (C-6), 147.2 (Cq arom.), 142.9 (C-8), 142.6 (Cq arom.), 135.0 (Cq arom.), 133.3 (Cq arom.), 132.9 (arom.), 132.0 (arom.), 132.0, 128.8 (arom.), 128.0 (arom.), 124.6 (arom.), 124.4 (C-5), 121.7 (arom.), 101.1 (C-1"), 88.9 (C-1'), 86.2 (C-4'), 84.2 (Cq *t*Bu), 84.1, 80.6 (C-4"), 80.6, 80.6, 80.5, 77.9 (C-2'), 73.4 (C-3'), 70.9 (C-2"), 70.9, 69.2 (C-3"), 65.9 (C-5"), 65.8, 65.8, 65.7, 65.0 (CH_2 ClNPE), 65.0, 64.9, 64.9, 62.6 (C-5'), 34.3 (CH_2 ClNPE), 34.2, 29.7 (CH_3 *t*Bu), 29.6, 29.6, 20.9 (CH_3 Ac), 20.7 (CH_3 Ac), 20.2 (CH_3 Ac). **^{31}P-NMR** (162 MHz, $CDCl_3$) δ -5.5, -5.5. **HRMS** [$C_{40}H_{46}ClN_6O_{17}P + H^+$] found: 949.2422, calculated: 949.2418.

***N*^{6}-Benzoyl-9-[2-*O*-(2,3-di-*O*-acetyl-5-*O*-(*tert*-butyl-2-chloro-4-nitro-phenylethyl)-*H*-phosphoryl-α-D-ribofuranosyl)-3-*O*-acetyl-5-*O*-(*O*-2-chloro-4-nitro-phenylethyl)-phosphoryl-(β-D-ribofuranosyl]-adenine** (**29**)

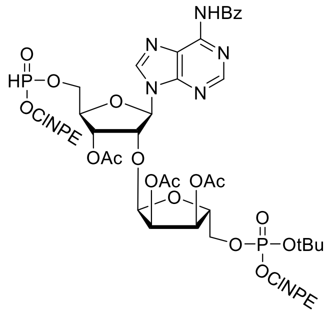

Dichlorophosphite **52** (PCl_2OClNPE, 1.7 g, 5.6 mmol, 2.0 eq.) was added to a flame dried flask under a nitrogen atmosphere, after which it was dissolved in DCM (11 mL, 0.5 M) and cooled to 0 °C. DIPA (1.6 mL, 11.6 mmol, 4.1 eq.) was added dropwise after which the reaction was allowed to reach room temperature and stirred for 30 minutes. NMM (1.9 mL, 16.9 mmol, 6.0 eq.) was added, followed by compound **33** (2.6 g, 2.8 mmol, 1.0 eq.) in DCM (15 mL, final concentration 0.1 M) after which the reaction was stirred for 15 minutes. The reaction was diluted with EtOAc and washed once with sat. aq. $NaHCO_3$. The combined organics were dried over $MgSO_4$, filtered and concentrated *in vacuo*. The crude mixture together with pyridine·HCl (650 mg, 5.6 mmol, 2 eq.) was dissolved in ACN (28 mL, 0.1 M), after which H_2O (0.25 mL, 14 mmol, 5 eq.) was added. The reaction was stirred for 50 minutes, after which it was concentrated *in vacuo*. The crude mixture was diluted with $CHCl_3$ and sat. aq. $NaHCO_3$, extracted twice with $CHCl_3$, dried with $MgSO_4$, filtered and concentrated *in vacuo*. Purification by flash column chromatography over high purity grade silica (0% -> 70% acetone/DCM) yielded the titled compound as a white foam (2.5 g, 2.1 mmol, 74%). **Rf** 0.49 (50% acetone/DCM). **^{1}H-NMR** (400 MHz, $CDCl_3$) δ 9.28 (s, 1H, NH), 8.76 (dd, J = 5.4, 1.7 Hz, 1H, H-2), 8.26 (d, J = 3.9 Hz, 1H, H-8), 8.24 - 8.20 (m, 2H, arom.), 8.09 - 8.01 (m, 4H, arom.), 7.65 - 7.57 (m, 1H, arom.), 7.57 - 7.44 (m, 4H, arom.), 6.80 (d, $^1J_{PH}$ = 716 Hz, 0.5 H, H-Phos diastereoisomer a), 6.78 (d, $^1J_{PH}$ = 716 Hz, 0.5 H, H-Phos diastereoisomer b), 6.16 (ddd, J = 8.0, 5.6, 2.4 Hz, 1H, H-1'), 5.51 - 5.43 (m, 1H, H-3'), 5.34 - 5.28 (m, 1H, H-1"), 5.23 - 5.10 (m, 2H, H-2'+H-3"), 4.73 (dq, J = 8.1, 4.2 Hz, 1H, H-2"), 4.46 - 4.30 (m, 5H, H-4'+H-5'+CH_2 ClNPE), 4.30 - 4.22 (m, 2H, CH_2 ClNPE), 4.20 - 4.15 (m, 1H, H-4"), 4.11 - 4.01 (m, 2H, H-5"), 3.26 - 3.15 (m, 4H, CH_2 ClNPE), 2.21 - 2.12 (m, 9H, CH_3 Ac), 1.42 (d, J = 3.4 Hz, 9H, CH_3 *t*Bu). **^{13}C-NMR** (101 MHz, $CDCl_3$) δ 170.1 (COO Ac), 169.6 (COO Ac), 169.5 (COO Ac), 164.7 (COO Bz), 152.8 (C-2), 151.6 (C-4), 149.9 (C-6), 147.4 (Cq arom.), 147.3 (Cq arom.), 142.7 (Cq arom.), 142.1 (Cq arom.), 141.8 (C-8), 141.6, 135.0 (Cq arom.), 133.4 (Cq arom.), 132.9 (arom.), 132.1 (arom.), 132.0, 132.0, 132.0, 128.8 (arom.), 128.0 (arom.), 124.7 (arom.), 124.6, 123.7 (C-5), 121.9 (arom.), 121.7, 101.3 (C-1"), 87.0 (C-1'), 86.9, 84.2 (Cq *t*Bu), 84.1, 81.1 (C-4'), 81.0, 80.9 (C-4"), 80.8, 80.7, 77.7 (C-2'), 77.6, 77.5, 70.9 (C-2'+C-3'), 70.9, 69.3 (C-3"), 65.9 (C-5'), 65.0 (CH_2 ClNPE), 65.0, 64.9, 64.2 (C-5'), 63.8 (CH_2 ClNPE), 63.7, 34.5 (CH_2 ClNPE), 34.5, 34.5, 34.4, 34.4 (CH_2 ClNPE), 34.3, 29.7 (CH_3 *t*Bu), 29.7, 29.6, 29.3, 20.8 (CH_3 Ac), 20.7 (CH_3 Ac), 20.2 (CH_3 Ac). **^{31}P-NMR** (162 MHz, $CDCl_3$) δ 9.5, 9.5, 8.9, -5.3, -5.3. **HRMS** [$C_{48}H_{53}Cl_2N_7O_{21}P_2 + H^+$] found: 1196.2224, calculated: 1196.2220.

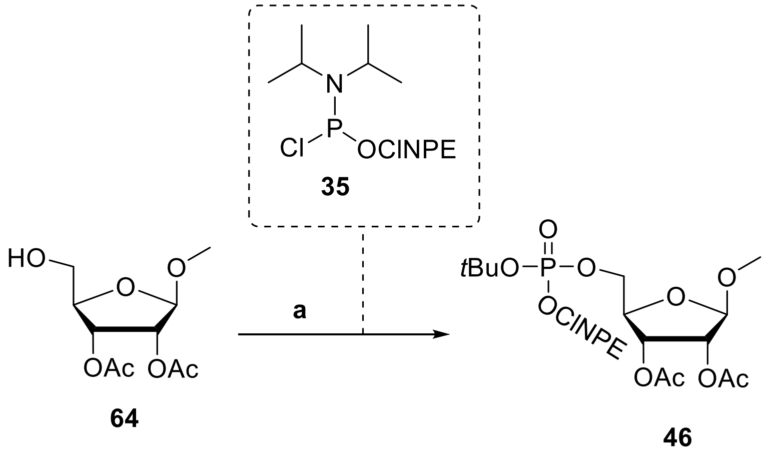

Scheme S6. Synthesis of asymmetric phosphotriester **46**. Reagents and conditions: a) (*i*) **35**, pyridine·HCl, pyridine, RT, 15 min, (*ii*) *t*BuOOH, 0°C, 30 min, quant. (**46**).

1-*O*-Methyl-2,3-di-*O*-acetyl-5-*O*-(*O*-tert-butyl-*O*-2-(2-chloro-4-nitro-phenyl)ethyl)-phosphoryl-β-D-ribofuranoside (**46**)

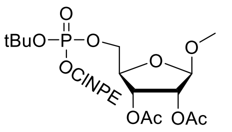

Compound **64**[15] (46 mg, 0.19 mmol, 1.0 eq.) and pyridine·HCl (42 mg, 0.37 mmol, 2 eq.) were co-evaporated (3x) with dioxane after which it was dissolved in pyridine (1.5 mL, 0.1 M). Amidite **35** (110 mg, 0.27 mmol, 1.5 eq.) was added and the mixture was stirred for 15 minutes. The solution was cooled to 0 °C after which *t*BuOOH (0.17 mL, 0.93 mmol, 5 eq., 5.5 M solution in decane) was added dropwise and stirred for 1 hour. The reaction was quenched with sat. aq. $NaHCO_3$ and extracted with EtOAc (2x). The combined organics were washed with brine, dried over $MgSO_4$, filtered, concentrated *in vacuo* and co-evaporated with toluene (3x). Purification by flash column chromatography over high purity grade silica (0% -> 10% acetone/DCM) yielded the titled compound as a white foam (81 mg, 0.19 mmol, quant). **Rf** 0.43 (10% acetone/DCM). **^{1}H-NMR** (400 MHz, $CDCl_3$) δ 8.26 (d, J = 2.4 Hz, 1H, arom.), 8.09 (dd, J = 8.5, 2.4 Hz, 1H, arom.), 7.56 (dd, J = 8.5, 2.9 Hz, 1H, arom.), 5.33 - 5.27 (m, 1H, H-3), 5.21 (d, J = 4.9 Hz, 1H, H-2), 4.91 (s, 1H, H-1), 4.36 - 4.21 (m, 3H, H-4+CH_2 ClNPE), 4.15 - 3.98 (m, 2H, H-5), 3.37 (d, J = 1.6 Hz, 1H, CH_3 OMe), 3.24 (t, J = 6.4 Hz, 2H, CH_2 ClNPE), 2.12 (s, 3H, CH_3 Ac), 2.07 - 2.04 (m, 3H, CH_3 Ac), 1.46 (s, 9H, CH_3 *t*Bu). **^{13}C-NMR** (101 MHz, $CDCl_3$) δ 169.7 (COO Ac), 169.7 (COO Ac), 147.3 (Cq arom.), 142.9 (Cq arom.), 142.8, 135.1 (Cq arom.), 132.2 (arom.), 132.1, 124.6 (arom.), 124.6, 121.8 (arom.), 106.3 (C-1), 83.9 (Cq *t*Bu), 83.9, 83.9, 83.8, 79.3 (C-4), 79.3, 79.2, 79.2, 74.6 (C-3), 74.5, 71.4 (C-2), 71.4, 67.4 (C-5), 67.4, 67.3, 65.1 (CH_2 ClNPE), 65.0, 55.3 (CH_3 OMe), 34.4 (CH_2 ClNPE), 34.4, 29.8 (CH_3 *t*Bu), 29.7, 20.6 (CH_3 Ac), 20.5 (CH_3 Ac). **^{31}P-NMR** (162 MHz, $CDCl_3$) δ -5.3, -5.3.

Solution phase synthesis of protected pyrophosphates

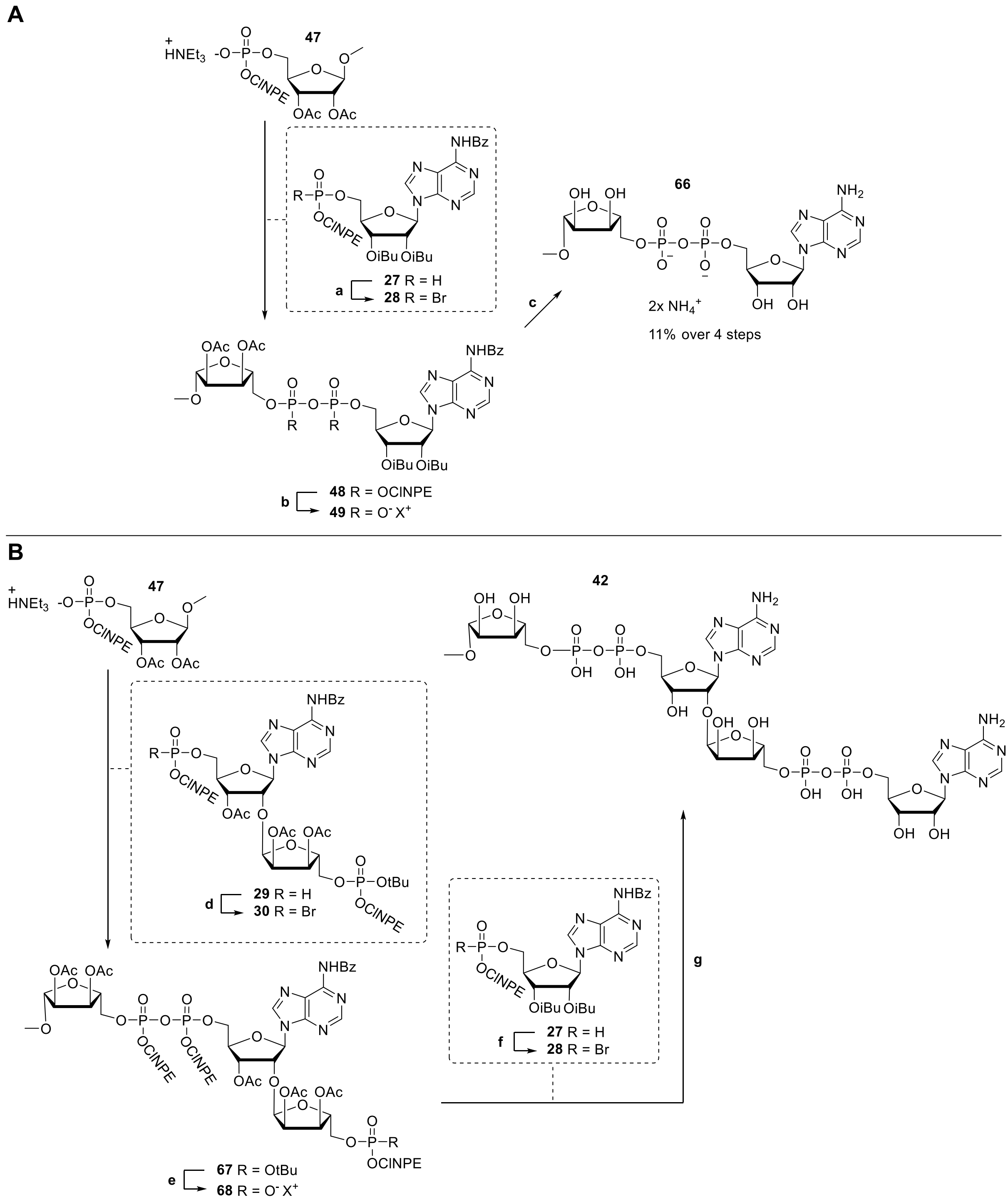

Scheme S7. Solution phase synthesis using protected pyrophosphates. Reagents and conditions. a) Cl_3CBr, NEt_3, ACN, 90 minutes; b) DBU, ACN, 90 minutes; c) NH_4OH, ACN, o.n., 11% (over 4 steps); d) Cl_3CBr, NEt_3, ACN, 2.5 hrs; e) TFA, DCM, 4 hrs; f) Cl_3CBr, NEt_3, ACN, 4 hrs; g) (i) DBU, ACN, 50 minutes, then NH_4OH, ACN, o.n.

Synthesis of 1”-β-O-methyl-ADPr 66 through a fully protected pyrophosphate intermediate

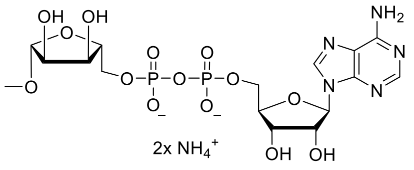

A mixture of compound **47** (51 mg, 0.10 mmol) and compound **27** (91 mg, 0.12 mmol, 1.2 eq.) was co-evaporated thrice with dried and distilled toluene after which the mixture was dissolved in freshly dried acetonitrile (1.0 mL). To this solution was added a stirring bar and the mixture was brought under a

nitrogen atmosphere before it was cooled to 0 °C using an icebath at which point it was stirred vigorously. In another flask, a stock solution of triethylamine in acetonitrile (0.3 M) was dried using freshly activated 3Å molecular sieves after which it was also cooled to 0 °C using an icebath. Then, some of the resulting stock solution (1.0 mL, 0.3 mmol of triethylamine, 3.0 eq.) was added to the vigorously stirring mixture of compounds **47** and **27**. To this mixture was then added Cl_3CBr (20 µL, 0.2 mmol, 2.0 eq.) after which the resulting clear and yellow solution was allowed to stir at 0 °C. TLC analysis indicated complete consumption of compound **27** after 90 minutes, in addition to the formation of a high running spot (Rf 0.8, 1/3 acetone/DCM) which was identified as the fully protected intermediate **48** via TLC-MS. At this point, DBU (0.60 mL, 4.0 mmol, 40 eq.) was added and the clear yellow solution almost immediately turned a deep amber red reminiscent of liquid bromine. The reaction mixture was allowed to warm up to room temperature while stirring. After 90 minutes of stirring at room temperature global deprotection was initiated via the addition of aq. NH_4OH solution (28% w/w)(3.6 mL). The flask was outfitted with a glass stopper and was stirred overnight. At this point, LCMS analysis indicated that no partially protected intermediates remained and the solution was washed thrice with diethyl ether, then thrice with DCM, then once more with diethyl ether. The aqueous layer was then concentrated *in vacuo* after which the crude product was purified using a C18AQ flash chromatography column cartridge (0% to 20% ACN in aq. $HNEt_3OAc$ (15 mM). Concentration of the fractions *in vacuo*, followed by de-salting through first NH_4 ion-exchange resin, size exclusion chromatography and subsequent lyophilization afforded title compound **66** as a white foam (6.8 mg, 11 µmol, 11%). All spectra are in accordance with literature. ^{1}H NMR (400 MHz, D_2O) δ 8.47 (s, 1H), 8.20 (s, 1H), 6.09 (d, J = 5.9 Hz, 1H), 4.81 (d, J = 1.3 Hz, 1H), 4.72 (t, J = 5.5 Hz, 1H), 4.49 (dd, J = 5.1, 3.6 Hz, 1H), 4.35 (dq, J = 4.8, 2.8 Hz, 1H), 4.22 – 4.14 (m, 3H), 4.10 – 4.00 (m, 2H), 3.95 (dd, J = 4.7, 1.3 Hz, 1H), 3.91 (q, J = 6.2 Hz, 1H), 3.29 (s, 3H). ^{13}C NMR (101 MHz, D2O) δ 155.55, 152.71, 149.11, 139.83, 107.80, 86.88, 83.87 (d, *J* = 8.6 Hz), 81.24 (d, *J* = 8.3 Hz), 74.29, 74.04, 70.68, 70.37, 66.47 (d, *J* = 4.5 Hz), 65.18 (d, *J* = 5.0 Hz), 55.08. ^{31}P NMR (162 MHz, D_2O) δ -11.33 (dd, J = 4.6, 2.3 Hz).

Part 2: Attempted isolation and purification of the fully protected pyrophosphate intermediate.

In tandem with the procedure described above, the same exact experiment was performed but this time stopping shy of pyrophosphate deprotection. Instead, after full consumption of the starting material **27** was observed, to the mixture was added 0.7 mL of dried and distilled toluene whereafter the resulting mixture was directly concentrated *in vacuo*. TLC analysis of the intermediate right after evaporation indicated only minimal degradation. However, attempts to purify the protected intermediate through column chromatography (ultrapure silica, neutralized, 5% to 40% acetone in DCM) were unsuccessful due to degradation on the column. Note: interestingly, TLC analysis of the protected pyrophosphate had been successful for neutralized, acidified (1% acetic acid) and basified (1% triethylamine) eluents, indicating a certain stability to these conditions, however brief.

Part 3: Attempted solution-phase synthesis of an ADP-ribose dimer.

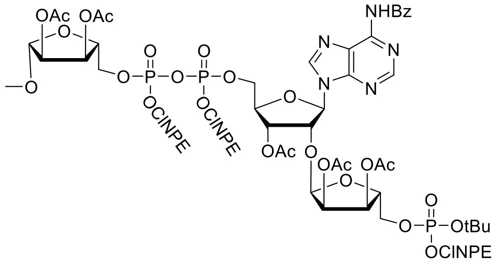

A mixture of compound **47** (51 mg, 0.10 mmol) and compound **29** (132 mg, 0.11 mmol, 1.1 eq.) was co-evaporated thrice with dried and distilled toluene after which the mixture was dissolved in freshly dried acetonitrile (1.0 mL). To this solution was added a stirring bar after which the mixture was brought under a nitrogen atmosphere. Having been cooled to 0 °C using an icebath, it was stirred vigorously. To it was then added a stock solution of triethylamine in acetonitrile (0.3 M) at 0 °C (1.0 mL, 0.3 mmol of triethylamine, 3.0 eq.) after which the reaction mixture was stirred vigorously. To this mixture was then added Cl_3CBr (20 µL, 0.2 mmol, 2.0 eq.) after which the resulting clear and yellow solution

was allowed to stir at 0 °C. TLC analysis indicated complete consumption of compound **29** after 150 minutes, in addition to the formation of a high running spot (Rf 0.9, 1/1 acetone/DCM) which was identified as the fully protected intermediate via TLC-MS. At this point, 1 mL of dried and distilled toluene was added to the mixture, whereafter the resulting solution was concentrated *in vacuo*.

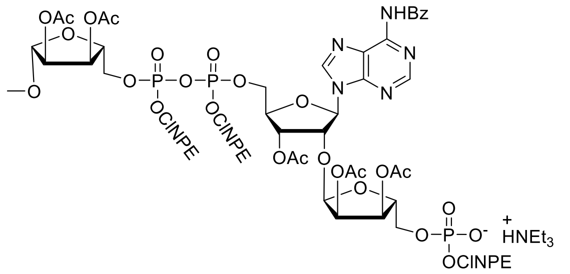

The crude product, an orange chunky oil, was then co-evaporated with dried and distilled toluene three times before being dissolved in dried DCM (3.0 mL, 0.03M). Stirring commenced, and to the stirring solution was added TFA (69 µL, 0.90 mmol, 9.0 eq.). The resulting deeply orange clear solution was allowed to stir at room temperature for four hours, at which point TLC analysis indicated complete consumption of the protected intermediate into a baseline spot consistent with the formation of the much more polar partially deprotected phosphate. To the reaction mixture was then added dried and distilled toluene (1.0 mL) and the reaction mixture was directly concentrated *in vacuo*. After this initial evaporation, the resulting reddish orange syrup was co-evaporated with dried and distilled toluene three more times. Then, to the mixture was added a mixture of dried and distilled toluene (1.5 mL) and triethylamine (130 µL). The reddish orange hue immediately made way for a more yellow orange one and the solution was once again concentrated *in vacuo*. This was followed by three additional co-evaporation cycles with dried and distilled toluene.

To the crude intermediate was then added compound **27** (91 mg, 0.12 mmol, 1.2 eq.) and the mixture was co-evaporated three times with dried and distilled toluene. After this, a stirring basr was added to the flask and the mixture of intermediates was dissolved in dried acetonitrile (1.0 mL, 0.10 M) to afford a clear and golden yellow solution. Stirring commenced and the flask was brought under a nitrogen atmosphere and cooled to 0 °C using an icebath. Once sufficiently cooled down, to the mixture were added first a stock solution of triethylamine in cooled and dried acetonitrile (1.0 mL, 0.3 mmol, 0.3 M) and then Cl_3CBr (20 µL, 0.20 mmol, 2.0 eq.). After stirring for four hours at 0 °C, TLC analysis indicated complete consumption of **27** but this time no higher running spot was observed in its stead. Nevertheless, DBU (0.60 mL, 4.0 mmol, 40 eq.) was then added whereafter the golden yellow and clear solution immediately turned the familiar liquid bromine-like colour. This mixture was then allowed to stir at room temperature for 50 minutes, whereafter aq. NH_4OH (28%, w/w) (10 mL) was added to it to facilitate global deprotection. The flask was then stoppered off with a glass topper and the mixture was stirred overnight at room temperature. The next morning, LCMS analysis indicated no presence of the product or its partially protected intermediates.

Solid-phase synthesis

General procedure C: resin preparation.

Resins containing succinyl linked thymidine were bought preloaded on Tentagel® N NH_2 resin. Loading of Q-linker containing thymidine **65** was performed manually, using fritted syringes (2, 5, 10 or 20 mL). Resins were loaded based on a scale of 100 µmol, based on resin loading supplied by the manufacturer, unless stated otherwise. Dry Tentagel® N NH_2 was swollen for 30 minutes prior to coupling. Q-linker containing thymidine **65** (3 eq.) was activated using DIC (3 eq.), ethyl-2-cyano-2-(hydroxyimino)acetate (ECHIA, Oxyma Pure) (0.1 eq.) and DIPEA (6 eq.) in ACN (0.15 M based on resin loading) for 3 minutes. The activated **65** was then added to the resin and shaken over night, after which the resin was washed with ACN. The resin was acetylated using a solution of Ac_2O (10% v/v) and pyridine (20% v/v) in ACN for 60 minutes. The resin was washed with ACN

and the acetylation was repeated once. The resin was washed with DCM, Et_2O and dried under reduced pressure, after which the loading was determined by trityl analysis (~150 µmol/g resin).

General procedure D: On-resin phosphorylation.

The DMTr ether was deprotected using 5% v/v TFA in DCM until no orange color could be observed, after which it was washed with DCM and dry ACN under a nitrogen atmosphere. A cocktail of imidazole (10 eq., 0.2 M) and 1-methyl imidazole·HCl (15 eq., 0.3 M) in DMF was added to the resin followed by amidite (**54**, **34** or **55**) (3 eq.), after which the reaction was shaken for 15 minutes. The resin was washed with ACN, after which *t*BuOOH (22 eq. 0.55 M in ACN) was added and the resin was shaken for 15 minutes. The resin was washed with ACN, DCM, Et_2O and dried under reduced pressure.

***t*Bu/NPE phosphoryl thymidine resin** (**14**)

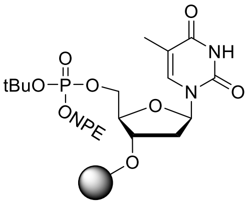

General procedure **C** was applied to 100 µmol Tentagel® N NH_2 resin using thymidine Q-linker **65**. The loading was determined by trityl analysis at 503 nm (150 µmol/g). General procedure **D** was applied to the resin using amidite **55** to give *t*Bu/NPE phosphorylated resin **14**.

***t*Bu/CNE phosphoryl thymidine resin** (**15**)

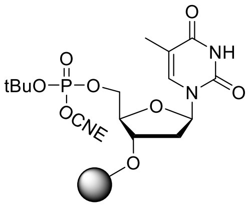

General procedure **C** was applied to 100 µmol Tentagel® N preloaded with thymidine (loading 180 µmol/g). General procedure **D** was applied to the resin using amidite **54** to give *t*Bu /CNE phosphorylated resin **15**.

***t*Bu/ClNPE phosphoryl thymidine resin** (**16**)

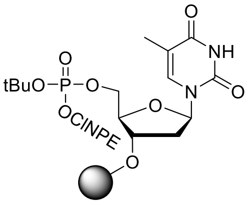

General procedure **C** was applied to 100 µmol Tentagel® N NH_2 resin using Thymidine Q-linker **56**. The loading was determined by trityl analysis at 503 nm (180 µmol/g). General procedure **D** was applied to the resin using amidite **34** to give *t*Bu/ClNPE phosphorylated resin **16**.

Optimization of protecting group strategy (Table 1).

Dimer 21 via *t*Bu pyrophosphate protection (**Table 1**, Entry 1)

2 µmol of resin **16** was loaded in a 2 mL fritted syringe and deprotected using 20% v/v DBU in ACN (0.1 mL/µmol) for 2x10 minutes. The resin was washed with ACN, NMM solution (0.1 M in ACN) and ACN. Building block **1** (3 eq., 0.1 M in ACN), TEA (9 eq., 0.3 M in ACN) and $CBrCl_3$ (6 eq., 0.2 M in ACN) were added consecutively. The resin was agitated for 30 minutes, washed with ACN, after which the coupling step was repeated. The pyrophosphate was deprotected using 3x5 minutes 40% TFA in DCM (0.1 mL/µmol). The resin was washed with ACN, after which a solution of NH_4OH (33% in H_2O, 1 mL) was added and the resin was shaken for 1 hour. The filtrate was collected and concentrated *in vacuo*, after which it was analyzed by LCMS.

Dimer 21 via NPE protection (**Table 1**, Entry 2)

2 µmol of resin **14** was loaded in a 2 mL fritted syringe and deprotected using 10% v/v TFA in DCM (0.1 mL/µmol) for 2x5 minutes. The resin was washed with ACN, NMM solution (0.1 M in ACN) and ACN. Building block **2** (3 eq., 0.1 M in ACN), TEA (9 eq., 0.3 M in ACN) and $CBrCl_3$ (6 eq., 0.2 M in

ACN) were added consecutively. The resin was agitated for 10 minutes, followed by a wash with ACN. The pyrophosphate was deprotected using 2x15 minutes of 15% DBU in ACN (0.1 mL/µmol). The resin was washed with ACN, after which it was treated with a solution of 10% v/v TFA in DCM until no orange color could be observed. NH_4OH (33% in H_2O, 1 mL) was added and the resin was shaken for 1 hour. The filtrate was collected and concentrated *in vacuo*, after which it was analyzed by LCMS.

Dimer 21 via CNE protection (**Table 1**, Entry 3)

2 µmol of resin **15** was loaded in a 2 mL fritted syringe and deprotected using 10% v/v TFA in DCM (0.1 mL/µmol) for 2x5 minutes. The resin was washed with ACN, NMM solution (0.1 M in ACN) and ACN. Building block **3** (3 eq., 0.1M in ACN), NMM (9 eq., 0.3M in ACN) and $CBrCl_3$ (6 eq., 0.2M in ACN) were added consecutively. The resin was agitated for 10 minutes, followed by a wash with ACN. The pyrophosphate was deprotected using 2x3 minutes of 5% v/v DBU in ACN (0.1 mL/µmol). The resin was washed with ACN, followed by treatment with a solution of 10% v/v TFA in DCM until no orange color could be observed. NH_4OH (33% in H_2O, 1 mL) was added and the resin was shaken for 1 hour. The filtrate was collected and concentrated *in vacuo*, after which it was analyzed by LCMS.

Trimer 26 via CNE protection (**Table 1**, Entry 4)

2 µmol of resin **15** was loaded in a 2 mL fritted syringe and deprotected using 10% v/v TFA in DCM (0.1 mL/µmol) for 2x5 minutes. The resin was washed with ACN, NMM solution (0.1 M in ACN) and ACN, after which building block **9** (3 eq., 0.1 M in ACN), NMM (9 eq., 0.3 M in ACN) and $CBrCl_3$ (6 eq., 0.2 M in ACN) were added consecutively. The resin was agitated for 5 minutes, after which the coupling step was repeated, followed by a wash with ACN. After coupling, the resin was deprotected using 10% v/v TFA in DCM (0.1 mL/µmol) for 2x5 minutes. The resin was washed with ACN, NMM solution (0.1 M in ACN) and ACN. Building block **3** (3 eq., 0.1 M in ACN), NMM (9 eq., 0.3 M in ACN) and $CBrCl_3$ (6 eq., 0.2 M in ACN) were added consecutively. The resin was agitated for 10 minutes and washed with ACN. The pyrophosphate was deprotected using 2x3 minutes of 5% v/v DBU in ACN (0.1 mL/µmol). The resin was washed with ACN, after which it was treated with a solution of 10% v/v TFA in DCM until no orange color could be observed. NH_4OH (33% in H_2O, 1 mL) was added and the resin was shaken for 1 hour. The filtrate was collected and concentrated *in vacuo*, after which it was analyzed by LCMS.

Dimer 21 via ClNPE protection (**Table 1**, Entry 5)

2 µmol of resin **16** was loaded in a 2 mL fritted syringe and deprotected using 10% v/v TFA in DCM (0.1 mL/µmol) for 2x5 minutes. The resin was washed with ACN, NMM solution (0.1M in ACN) and ACN, after which building block **4** (3 eq., 0.1M in ACN), NMM (9 eq., 0.3M in ACN) and $CBrCl_3$ (6 eq., 0.2M in ACN) were added consecutively. The resin was agitated for 10 minutes, followed by a wash with ACN. The coupling step was repeated, after which the pyrophosphate was deprotected using 2x5 minutes of 20% v/v DBU in ACN (0.1 mL/µmol). The resin was washed with ACN, followed by treatment with a solution of 10% v/v TFA in DCM until no orange color could be observed. NH_4OH (33% in H_2O, 1 mL) was added and the resin was shaken for 1 hour. The filtrate was collected and concentrated *in vacuo*, after which it was analyzed by LC-MS.

Trimer 26 via ClNPE protection (Table 1, Entry 6)

2 µmol of resin **16** was loaded in a 2 mL fritted syringe and deprotected using 10% v/v TFA in DCM (0.1 mL/µmol) for 2x5 minutes. The resin was washed with ACN, NMM solution (0.1 M in ACN) and ACN, after which building block **10** (3 eq., 0.1 M in ACN), NMM (9 eq., 0.3 M in ACN) and $CBrCl_3$ (6 eq., 0.2 M in ACN) were added consecutively. The resin was agitated for 15 minutes, after which the coupling step was repeated, followed by a wash with ACN. After coupling, the resin was deprotected using 10% v/v TFA in DCM (0.1 mL/µmol) for 2x5 minutes. The resin was washed with ACN, NMM solution (0.1 M in ACN) and ACN. Building block **4** (3 eq., 0.1 M in ACN), NMM (9 eq.,

0.3 M in ACN) and $CBrCl_3$ (6 eq., 0.2 M in ACN) were added consecutively, the resin was agitated for 15 minutes, after which the coupling step was repeated. The pyrophosphate was deprotected using 2x5 minutes of 20% v/v DBU in ACN (0.1 mL/µmol). The resin was washed with ACN, after which it was treated with a solution of 10% v/v TFA in DCM until no orange color could be observed. NH_4OH (33% in H_2O, 1 mL) was added and the resin was shaken for 1 hour. The filtrate was collected and concentrated *in vacuo*, after which it was analyzed by LCMS.

Dimer 21 via ClNPE protection (**Table 1**, Entry 7)

2 µmol of resin **16** was loaded in a 2 mL fritted syringe and deprotected using 10% v/v TFA in DCM (0.1 mL/µmol) for 2x5 minutes. The resin was washed with ACN, TEA solution (0.1 M in ACN) and ACN, after which building block **4** (3 eq., 0.1 M in ACN), TEA (9 eq., 0.3 M in ACN) and $CBrCl_3$ (6 eq., 0.2 M in ACN) were added consecutively. The resin was agitated for 10 minutes, followed by a wash with ACN. The coupling step was repeated, after which the pyrophosphate was deprotected using 2x5 minutes of 20% v/v DBU in ACN (0.1 mL/µmol). The resin was washed with ACN, followed by treatment with a solution of 10% v/v TFA in DCM until no orange color could be observed. NH_4OH (33% in H_2O, 1 mL) was added and the resin was shaken for 1 hour. The filtrate was collected and concentrated *in vacuo*, after which it was analyzed by LCMS.

Trimer 26 via ClNPE protection (**Table 1**, Entry 8)

2 µmol of resin **16** was loaded in a 2 mL fritted syringe and deprotected using 10% v/v TFA in DCM (0.1 mL/µmol) for 2x5 minutes. The resin was washed with ACN, TEA solution (0.1 M in ACN) and ACN, after which building block **10** (3 eq., 0.1 M in ACN), TEA (9 eq., 0.3 M in ACN) and $CBrCl_3$ (6 eq., 0.2 M in ACN) were added consecutively. The resin was agitated for 1 minute, after which the coupling step was repeated, followed by a wash with ACN. After coupling, the resin was deprotected using 10% v/v TFA in DCM (0.1 mL/µmol) for 2x5 minutes. The resin was washed with ACN, TEA solution (0.1 M in ACN) and ACN. Building block **4** (3 eq., 0.1 M in ACN), TEA (9 eq., 0.3 M in ACN) and $CBrCl_3$ (6 eq., 0.2 M in ACN) were added consecutively, the resin was agitated for 1 minute, after which the coupling step was repeated. The pyrophosphate was deprotected using 2x5 minutes of 20% v/v DBU in ACN (0.1 mL/µmol). The resin was washed with ACN, after which it was treated with a solution of 10% v/v TFA in DCM until no orange color could be observed. NH_4OH (33% in H_2O, 1 mL) was added and the resin was shaken for 1 hour. The filtrate was collected and concentrated *in vacuo*, after which it was analyzed by LCMS.

Solid-phase synthesis of oligo-TDP oligomers

Oligo-TDP trimer (**26**)

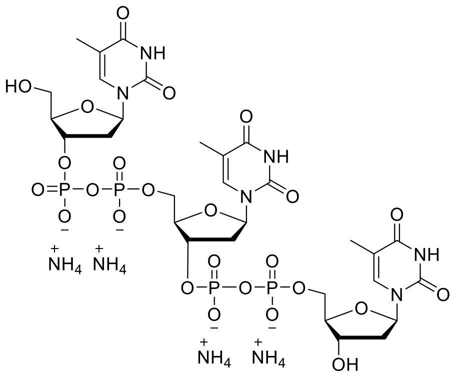

5 µmol of resin **16** was swollen in a reaction column of a Mermade 6 oligonucleotide synthesizer with ACN for 5 minutes, prior to solid-phase synthesis. Synthesis was performed under an argon atmosphere. The resin was treated thrice with a 5% v/v TFA/DCE, 0.1 mL/µmol) for 5 minutes per deprotection cycle. The resin was washed with ACN, after which it was washed once with TEA solution (0.3 M in ACN). Elongation of the oligo-TDP chain was performed with H-phosphonate **10** (3 eq., 0.1 M in ACN), TEA (9 eq., 0.3 M in ACN) and $CBrCl_3$ (6 eq., 0.2 M in ACN) which were added consecutively to the resin, after which the reaction was left to stand for 1 minute. The resin was washed with ACN after which the coupling step was repeated twice. After coupling, the resin was deprotected thrice with a 5% v/v solution of TFA/DCE (0.1 mL/µmol). The resin was washed with ACN, after which it was washed once with TEA solution (0.3 M in ACN). The oligo-TDP chain was terminated via coupling of building block **4** (3 eq., 0.1 M in ACN), TEA (9 eq., 0.3 M in ACN) and

$CBrCl_3$ (6 eq., 0.2 M in ACN) which were added consecutively to the resin, after which it was left to stand for 1 minute. The resin was washed with ACN after which the coupling was repeated twice. The resin was washed with ACN, after which a solution of 20% v/v DBU in ACN (0.1 mL/μmol) was added which was left to stand for 5 minutes. The resin was washed with ACN and the base deprotection was repeated once. The resin was treated with a solution of 5% v/v solution of TFA/DCE. The resin was washed with ACN, after which a solution of NH_4OH was added for 1 hour to cleave the oligo-TDP construct of the resin. The filtrate was collected and concentrated *in vacuo*, after which it was analyzed by LCMS and/or IEX. The crude oligomer was purified by preparative anion exchange chromatography using a gradient of 0-25% buffer B. The fractions containing trimer **26** were collected, lyophilized, and subjected to size exclusion chromatography. Oligomer **26** was lyophilized and isolated as a white powder (1.60 mg, 1.48 μmol, 30%) ^{1}**H-NMR** (400 MHz, D_2O) δ 7.72 - 7.59 (m, 3H, H-6), 6.32 - 6.19 (m, 3H, H-1'), 4.93 (t, *J* = 6.8 Hz, 1H, H-3'), 4.56 (q, *J* = 4.8 Hz, 1H, H-3'), 4.36 (t, *J* = 2.6 Hz, 1H, H-4'), 4.21 - 4.07 (m, 6H, H-4'+H-5'), 3.82 - 3.71 (m, 2H, H-5'), 2.57 - 2.48 (m, 2H, H-2'), 2.38 - 2.26 (m, 4H, H-2'), 1.91 - 1.80 (m, 9H, CH_3 T). ^{31}**P-NMR** (162 MHz, D_2O) δ -10.8, -10.9, -11.0, -11.2, -11.6, -11.7, -11.8. **HRMS** [$C_{30}H_{42}N_6O_{25}P_4 + Na^+$] found: 1033.10648, calculated: 1033.10423.

***t*Bu/ClNPE Phosphorylribose resin** (**36**)

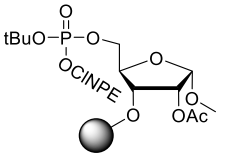

Tentagel® N NH_2 (100 μmol, 0.22 mmol/g resin) was swollen for 30 minutes in ACN. Q-linker containing ribose[16] (3 eq.) was activated using DIC (3 eq.), ethyl-2-cyano-2-(hydroxyimino)acetate (ECHIA, Oxyma Pure) (0.1 eq.) and DIPEA (6 eq.) in ACN (0.15 M based on resin loading) for 3 minutes. The activated ribose was then added to the resin and shaken over night, after which the resin was washed with ACN. The resin was acetylated with a solution of Ac_2O (10% v/v) and pyridine (20% v/v) in ACN for 60 minutes. The resin was washed with ACN and the acetylation step was repeated once. The resin was washed with DCM, Et_2O and dried under reduced pressure, after which the loading was determined by trityl analysis (80 μmol/g resin). The DMTr was deprotected using 5% v/v TFA in DCM until no orange color could be observed, after which it was washed with DCM and dry ACN under nitrogen atmosphere. A cocktail of imidazole (10 eq., 0.2 M) and 1-methyl imidazole·HCl (15 eq., 0.3 M) in DMF was added to the resin followed by amidite **34** (3 eq.), after which the reaction was shaken for 15 minutes. The resin was washed with ACN, after which *t*BuOOH (22 eq. 0.55 M in ACN) was added and the resin was shaken for another 15 minutes. The resin was washed with ACN, DCM, Et_2O and dried under reduced pressure to give *t*Bu/ClNPE phosphoribosylated resin **36**.

General procedure A: solid-phase synthesis of oligo-ADPr

Resin containing asymmetric phosphotriester **36** was swollen in a fritted syringe with ACN for 5 minutes, prior to solid-phase synthesis. The syringe was drained and washed twice with a 20% v/v solution of TFA in DCM, after which the *t*Bu was deprotected thrice for 4 minutes with 20% v/v TFA/DCM (0.1 mL/μmol resin). The resin was washed with ACN, after which it was washed once with a solution of NMM (0.1 M in ACN).

Elongation cycle: For the coupling of the elongating building block, H-phosphonate **29** (3 eq., 0.1 M in ACN), TEA (9 eq., 0.6 M in ACN) and $CBrCl_3$ (6 eq., 0.4 M in ACN) were added consecutively to the immobilized phosphodiester, after which the resin was agitated for 2 minutes. The resin was washed with ACN after which the coupling step was repeated. The resin was washed extensively with ACN, twice with a 20% v/v solution of TFA in DCM, after which the *t*Bu was deprotected thrice for 4 minutes with 20% v/v TFA/DCM (0.1 mL/μmol resin). The resin was washed extensively with ACN, after which it was washed once with a solution of NMM (0.1 M in ACN).

Chain termination cycle: For the coupling of the terminating building block, H-phosphonate **27** (3 eq., 0.1 M in ACN), TEA (9 eq., 0.6 M in ACN) and $CBrCl_3$ (6 eq., 0.4 M in ACN) were added consecutively to the immobilized phosphodiester, after which the resin was agitated for 2

minutes. The resin was washed with ACN after which the coupling step was repeated. The resin was washed extensively with ACN, after which a solution of DBU (0.1 mL/µmol resin) in ACN was added, followed by agitation of the resin for 5 minutes. The resin was washed with ACN and the deprotection was repeated once.

Resin cleavage and final deprotection: The resin was washed with ACN, after which a solution of NH_4OH (32% in H_2O, 0.5 mL/µmol) was added, followed by addition of Ethylenediaminetetraacetic acid (EDTA) (final concentration of 0.5 mM from a stock of 0.5 M, pH 8 in H_2O) and the resin was shaken overnight. The filtrate was collected and lyophilized, after which it was analyzed by LCMS and/or IEX.

Oligo-ADPr dimer (42)

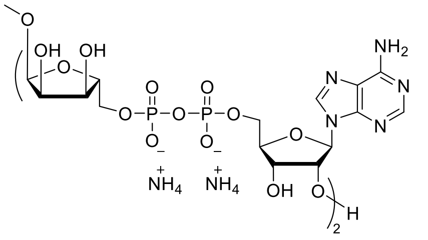

General procedure **A** was applied to 2 µmol of resin **36** to synthesize oligo-ADPr dimer **42**. The elongation cycle was repeated once using building block **29**, 2 minutes of coupling time and a single coupling procedure. The termination cycle was performed using building block **27**, 2 minutes of coupling time and a single coupling procedure. The crude oligomer was analyzed via LCMS and IEX. The crude dimer was purified by preparative anion exchange chromatography using a gradient of 0-40% buffer B. The fractions containing dimer **42** were collected, lyophilized, and subjected to size exclusion chromatography. Oligomer **42** was lyophilized repeatedly and isolated as a white powder (0.62 mg, 0.52 µmol, 27%) **^{1}H-NMR** (400 MHz, D_2O) δ 8.28 - 8.19 (m, 2H, H-2), 8.03 - 7.94 (m, 2H, H-8), 6.02 (d, *J* = 3.2 Hz, 1H, H-1'), 5.84 (d, *J* = 6.0 Hz, 1H, H-1'), 5.15 (d, *J* = 4.3 Hz, 1H, H-1") 3.31 (s, 3H, OMe). **^{31}P-NMR** (162 MHz, D_2O) δ -10.4, -10.5, -10.6, -10.6, -10.7, -10.8. Spectra were in full accordance with literature experimental data.[16] **HRMS** [$C_{31}H_{46}N_{10}O_{27}P_4$ + H^+] found: 1115.15667, calculated: 1115.15571.

Oligo-ADPr trimer (43)

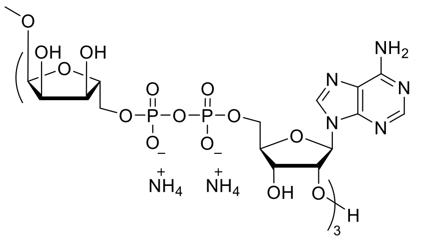

General procedure **A** was applied to 2 µmol of resin **36** to synthesize oligo-ADPr trimer **43**. The crude oligomer was analyzed via LCMS and IEX. The crude trimer was purified by preparative anion exchange chromatography using a gradient of 0-50% buffer B. The fractions containing trimer **43** were collected, lyophilized, and subjected to size exclusion chromatography. Oligomer **43** was lyophilized repeatedly and isolated as a white powder (1.07 mg, 0.61 µmol, 30%) **^{1}H-NMR** (400 MHz, D_2O) δ 8.38 - 8.34 (m, 2H, H-2), 8.29 (s, 1H, H-2), 8.10 (s, 1H, H-8), 8.07 (s, 1H, H-8), 8.04 (s, 1H, H-8), 6.12 (d, *J* = 2.9 Hz, 1H, H-1'), 5.97 (d, *J* = 2.2 Hz, 1H, H-1'), 5.94 (d, *J* = 6.0 Hz, 1H, H-1'), 5.30 (d, *J* = 4.2 Hz, 1H, H-1"), 5.22 (d, *J* = 4.2 Hz, 1H, H-1"), 3.33 (s, 3H, OMe). **^{31}P-NMR** (162 MHz, D_2O) δ -10.34, -10.46, -10.54, -10.59, -10.72, -11.00. Spectra were in full accordance with literature experimental data.[16] **HRMS** [$C_{46}H_{67}N_{15}O_{40}P_6$+ H^+] found: 1656.21965, calculated: 1656.21682.

Oligo-ADPr hexamer (44)

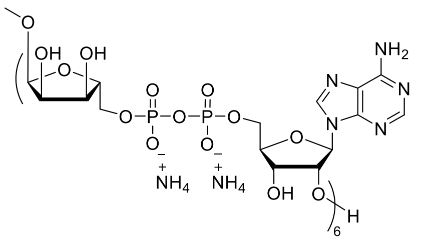

General procedure **A** was applied to 2 µmol of resin **36** to synthesize oligo-ADPr hexamer **44**. The crude oligomer was analyzed via LCMS and IEX. The crude hexamer was purified by preparative anion exchange chromatography using a gradient of 25-60% buffer B. The fractions containing hexamer **44** were collected, lyophilized, and subjected to size exclusion chromatography. Oligomer **44** was lyophilized repeatedly and isolated as a white powder (0.58 mg, 180 nmol, 8.8%) **^{1}H-NMR** (400 MHz, D_2O) δ 8.35 (s, 1H, H-2), 8.31 - 8.24 (m, 5H, H-2), 8.08 (s, 1H, H-8), 8.01 - 7.93 (m, 4H, H-8), 6.12 (d, *J* = 3.4 Hz, 1H, H-1'), 6.02 - 5.96 (m, 4H, H-1'), 5.91 (d, *J* = 5.8 Hz, 1H ,H-1'), 5.30 - 5.20 (m, 4H, H-1"), 3.32 (s, 3H, OMe).

^{31}**P-NMR** (162 MHz, D_2O) δ -10.4, -10.4, -10.5, -10.5, -10.6, -10.6, -10.6, -10.7, -10.7. **LCMS** (00% -> 20% MeCN) RT = 3.32 min. **HRMS** [$C_{91}H_{130}N_{30}O_{79}P_{12}$+ $2H^+$] found: 1640.7081, calculated: 1640.7051.

Oligo-ADPr decamer (45)

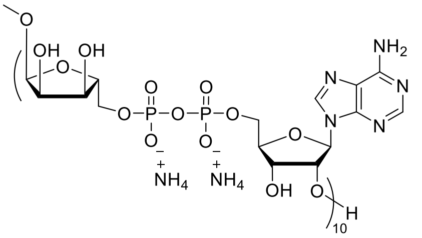

General procedure **A** was applied to 2 µmol of resin **36** to synthesize oligo-ADPr decamer **45**. The crude oligomer was analyzed via LC-MS and IEX. The crude decamer was purified by preparative anion exchange chromatography using a gradient of 50-90% buffer B. The fractions containing decamer **25** were collected, lyophilized, and subjected to size exclusion chromatography. Oligomer **45** was lyophilized repeatedly and isolated as a white powder (0.50 mg, 95 nmol, 4.8%) ^{1}**H-NMR** (400 MHz, D_2O) δ 8.23 (s, 10H, H-2), 7.98 - 7.80 (m, 10H, H-8), 6.08 - 5.79 (m, 10H, H-1'), 5.13 (d, *J* = 24.0 Hz, 10H, H-1"), 3.22 (s, 3H, OMe). ^{31}**P-NMR** (162 MHz, D_2O) δ -10.4, -10.4, -10.4, -10.5, -10.5, -10.6, -10.6, -10.67, -10.7, -10.8, -10.8. **LCMS** (00% -> 20% MeCN) RT = 3.51 min. **LRMS** calculated $[M+3H]^{3+}$ = 1815.21, $[M+4H]^{4+}$ = 1361.66; observed m/z 1815.58, 1362.08.

Atherton-Todd oxidation of H-phosphonates followed by ^{31}P-NMR

Oxidation of terminating H-phosphonates

All solutions were dried over 3 Å molecular sieves for one night prior to experiments. ^{31}P-NMR spectra were measured on a Bruker 300 AV NMR spectrometer. Spectra were referenced indirectly to H_3PO_4 (0.00 ppm) in D_2O. To an NMR tube was added H-phosphonate (1 eq, 60 µL 6 µmol, 0.1 M in ACN), $CBrCl_3$ (2 eq, 60 µL 12 µmol, 0.2 M in ACN) and ACN (270 µL). The NMR tube was fitted with a glass insert filled with deuterated acetone for signal locking. The NMR machine was set up and shimmed after which an ^{31}P-NMR spectrum was measured at t=0 min. The NMR tube was removed after which base was added (3 eq, 60 µL 18 µmol, 0.3 M in ACN) starting the Atherton-Todd oxidation. The sample was quickly homogenized using a vortex and measured using the previously obtained shimming parameters. The oxidation reaction was followed over time by ^{31}P NMR. The early time points (1-5 minutes) were averaged over 10 scans per time point, while the later time points (10-40 minutes) were averaged over 50 scans.

P(V)-P(V) coupling to protected ADP-ribose (Figure S7)

Asymmetrical phosphotriester **46** (8.4 mg, 15 µmol) was dissolved in DCM (15 mM) after which TFA (50 µL, 50 µmol, 3 eq.) was added. The solution was stirred for 30 minutes, after which the reaction was concentrated *in vacuo*, followed by co-evaporation with toluene (2x), TEA (1x) and toluene (3x). The solution was dissolved in ACN (150 µL, 0.1 M) after which H-phosphonate **27** was added (13.5 mg, 18 µmol, 1.2 eq., 0.1 M solution in ACN). To the solution was added 3 Å molecular sieves after which the solution was dried over night. The solution was loaded in an NMR tube which was fitted with a glass insert filled with deuterated acetone. A ^{31}P-NMR spectrum was measured. To the reaction mixture in the NMR tube was added TEA (3 eq, 150 µL, 45 µmol, 0.3 M in ACN) and $CBrCl_3$ (2 eq, 150 µL, 30 µmol, 0.2 M in ACN). The coupling was followed over time by ^{31}P-NMR. After completion of the coupling, the ClNPE pyrophosphate protecting groups were cleaved by addition of DBU (90 µL, 600 µmol, 40 eq., final concentration ~15% v/v). The deprotection was again followed by ^{31}P-NMR. The data was compiled in **Figure S7**.
